# AI Models for Predicting Acute Kidney Injury (AKI) and Post-AKI Mortality: Systematic Review and Meta-Analysis

**DOI:** 10.2196/104269

**Published:** 2026-08-20

**Authors:** Huiyu Xiao, Jingjing Liang, Gaoming Li, Yunhao Yang, Tianxin Li, Xin Chen, Qiangguo Ao, Yazhou Wu, Qiuyue Song

**Affiliations:** 1 Department of Health Statistics, Army Medical University, No. 30 Gaotanyan Street, Shapingba District, Chongqing, China, 86 13708344829; 2 Department of Neurology, Xinqiao Hospital and The Second Affiliated Hospital, Army Medical University, Chongqing, China; 3 Department of Nephrology, The Second Medical Center of Chinese People's Liberation Army General Hospital, Beijing, China

**Keywords:** acute kidney injury, artificial intelligence, prediction model, risk prediction, mortality

## Abstract

**Background:**

Machine learning (ML) models are increasingly used to predict acute kidney injury (AKI), but validation quality and clinical readiness remain uncertain.

**Objective:**

This systematic review and meta-analysis aimed to summarize discrimination performance and implementation-relevant gaps for AKI occurrence and post-AKI mortality prediction.

**Methods:**

We searched the Cochrane Library, Embase, PubMed, and Web of Science through January 23, 2025. Eligible studies developed or validated ML-based prediction models and reported the area under the receiver operating characteristic curve (AUC). Two reviewers screened studies, extracted data, and assessed risk of bias using the PROBAST+AI (Prediction Model Risk Of Bias Assessment Tool+Artificial Intelligence). Logit-transformed AUCs were pooled using restricted maximum likelihood random-effects meta-analysis with Hartung-Knapp-Sidik-Jonkman–adjusted inference.

**Results:**

We included 219 studies with 7,343,170 participants and 101 modeling approaches. Primary analyses included 188 AUC estimates for AKI occurrence and 31 for post-AKI mortality. Pooled AUCs were 0.834 (95% CI 0.821‐0.846) for AKI occurrence prediction and 0.830 (95% CI 0.807‐0.851) for post-AKI mortality prediction. For AKI occurrence, nonlinear approaches, especially deep learning and tree-based or ensemble methods, generally showed higher pooled AUC point estimates than linear or generalized linear models in exploratory subgroup analyses. At the study level, PROBAST+AI rated 129 (58.9%) studies as having low risk, 84 (38.4%) studies as having high risk, and 6 (2.7%) studies as having unclear risk. External validation was uncommon: it was reported in 30 (16.0%) AKI occurrence records and 9 (29.0%) post-AKI mortality records.

**Conclusions:**

ML models have shown high average discrimination for AKI occurrence and post-AKI mortality, supporting their potential value for AKI risk stratification and early warning. However, high levels of heterogeneity, limited external or prospective validation, and inconsistent reporting of model calibration and clinical utility mean that substantial barriers remain before routine clinical deployment. Pooled AUC estimates revealed that nonlinear models have considerable clinical translational potential. Further refinements to modeling frameworks are warranted to explore feasible strategies for real-world clinical implementation. Future studies should prioritize standardized definitions, robust validation, clinically meaningful thresholds, assessment of alert burden, and evidence that model-guided care improves kidney-protective management or patient outcomes.

## Introduction

Acute kidney injury (AKI) is a common and serious clinical syndrome characterized by an abrupt decline in kidney excretory function over hours to days. It occurs in a substantial proportion of hospitalized and critically ill patients, particularly in intensive care units (ICUs), and is associated with increased short- and long-term morbidity, mortality, longer hospital length of stay, and higher health care costs [1-3]. Early identification of patients at high risk of AKI may allow clinicians to implement preventive strategies, intensify monitoring, and allocate limited critical care resources more effectively [4,5].

However, timely prediction of AKI remains challenging. AKI is clinically heterogeneous, with diverse etiologies, complex pathophysiology, variable prediction windows, and often nonspecific early manifestations [6]. The increasing availability of electronic health record data has created new opportunities to develop prediction models that integrate comorbidities, laboratory results, medication exposure, vital signs, urine output, and other longitudinal clinical information. Machine learning (ML) methods can capture nonlinear relationships and high-order interactions that are difficult to model using conventional approaches, and several algorithms, including random forests, gradient boosting methods, support vector machines, and neural networks, have shown promising performance in AKI prediction [7-10].

Existing studies of ML-based AKI prediction differ substantially in patient population, AKI definition, prediction horizon, validation strategy, clinical setting, and model class. These differences are clinically important because AKI risk stratification at hospital admission, perioperative AKI monitoring, ICU real-time alerting, and specialty-specific prediction represent distinct decision-support tasks. Similarly, linear or generalized linear models, tree-based or ensemble methods, deep learning models, and hybrid approaches may differ in discrimination performance, interpretability, data requirements, and readiness for clinical use. Previous reviews have described AKI prediction models, but few have provided a large-scale quantitative synthesis that separates AKI occurrence from post-AKI mortality while simultaneously examining performance according to clinical application scenario and model class. Therefore, this systematic review and meta-analysis aimed to summarize the discrimination performance of ML-based models for predicting AKI occurrence and post-AKI mortality, and to explore whether performance differed across clinical application scenarios, model classes, validation characteristics, and risk of bias profiles.

## Methods

### Search Strategy

This systematic review was registered in PROSPERO (International Prospective Register of Systematic Reviews) in March 2026 (CRD420261333545). No separate review protocol was prepared or made publicly available beyond the PROSPERO registration record. No amendments to the registered methods were made after registration. This systematic review and meta-analysis was conducted and reported in accordance with PRISMA (Preferred Reporting Items for Systematic Reviews and Meta-Analyses) 2020 and guidance for systematic reviews and meta-analyses of prediction model performance [11]. The completed PRISMA 2020 checklist is provided in the Checklist 1. We searched the Cochrane Library, PubMed, Web of Science, and Embase from inception to January 23, 2025, using database-specific combinations of controlled vocabulary and free-text terms related to AKI, ML, and prediction models. No additional trial registers, websites, organizational sources, or reference lists were searched. The complete search strategies are provided in Table S1 in Multimedia Appendix 1.

### Eligibility Criteria and Study Selection

Two reviewers independently screened titles, abstracts, and full texts, with disagreements resolved by discussion or consultation with a third reviewer. Eligible studies developed or validated an AI- or ML-based multivariable prediction model for AKI occurrence or post-AKI mortality and reported AUC. We excluded reviews, editorials, guidelines, studies without full text, nonprediction model studies, studies without eligible outcomes or ML-based prediction models, and studies with fewer than 200 participants. Reference management software (EndNote 20) was used to remove duplicates and manage records.

### Data Extraction

Two reviewers independently extracted study characteristics, population and outcome definitions, data source, validation strategy, model type, sample size, number of outcome events, and model performance metrics using a standardized form. AKI occurrence and post-AKI mortality time horizons were extracted as reported in the original studies. Disagreements were resolved by discussion or consultation with a third reviewer. Study investigators were not contacted for additional information. When information was missing or unclear, data were recorded as not reported or unclear rather than imputed. AUC and its variance information were extracted when available. Other model performance information, when reported, was recorded descriptively. Because calibration measures, decision-curve analyses, operating thresholds, and clinical utility outcomes were not consistently available and were not included in the primary quantitative synthesis, the meta-analysis focused on AUC as the main discrimination metric. Details of extracted variables are provided in Table S2 in Multimedia Appendix 1. Clinical application scenarios were classified into four mutually exclusive categories. Detailed operational definitions are provided in Table S2 in Multimedia Appendix 1.

### Risk of Bias and Applicability Assessment

Risk of bias and applicability were assessed independently by 2 reviewers using PROBAST+AI (Prediction Model Risk Of Bias Assessment Tool+Artificial Intelligence), covering participants and data sources, predictors, outcomes, and analysis [12]. Disagreements were resolved by discussion or consultation with a third reviewer. Overall risk of bias was determined according to PROBAST+AI guidance. The PROBAST+AI signaling questions are listed in Tables S3 and S4 in Multimedia Appendix 1, and study-level assessments are provided in Table S6 in Multimedia Appendix 1.

### Definition of Analysis Units

We prespecified one representative AUC estimate per study and per outcome for the primary meta-analysis, selected according to the following validation hierarchy: external validation, internal validation, test cohort, training cohort, and other or unclear cohorts. When multiple eligible estimates were available within the same validation category, the estimate with the highest reported AUC was selected. A total of 219 studies were included for qualitative synthesis. Two records assessing AKI incidence were excluded from quantitative pooling owing to missing AUC values, leaving 217 studies with eligible and complete AUC data. As 2 studies reported both end points simultaneously, we ultimately obtained 219 outcome-specific analytical records, consisting of 188 records for AKI occurrence and 31 records for post-AKI mortality, all of which were incorporated into the quantitative meta-analysis. For complementary model-level analyses, the 1881 extracted model performance records were collapsed by article, outcome, and model or modeling approach, yielding 404 AUC records for AKI occurrence and 72 AUC records for post-AKI mortality. We identified 101 distinct modeling approaches to describe algorithmic diversity, but these were not treated as independent units in the primary meta-analysis. The 101 distinct modeling approaches were defined at the extracted algorithm-specific or study-reported model name level rather than at the level of the 4 broad model classes. For example, random forest, XGBoost (Extreme Gradient Boosting), LightGBM (Light Gradient Boosting Machine), support vector machine, multilayer perceptron, convolutional neural network, recurrent neural network, and study-specific hybrid pipelines were counted as distinct modeling approaches when reported as separate models by the original studies. These approaches were then mapped into 4 broad AI model classes—linear/generalized linear models, tree-based and ensemble methods, deep learning models, and hybrid models or others—for subgroup and meta-regression analyses.

### Statistical Analysis

Random-effects meta-analyses were performed using logit-transformed AUCs with restricted maximum likelihood estimation of between-study variance. Confidence intervals and statistical tests for pooled estimates, subgroup analyses, sensitivity analyses, and meta-regression were calculated using the Hartung-Knapp-Sidik-Jonkman (HKSJ) adjustment, and results were back-transformed to the AUC scale for presentation [13]. Standard errors were derived preferentially from reported 95% CIs, followed by the Hanley-McNeil approximation based on event and nonevent counts, and then reported SDs when available. AUC estimates without derivable variance information were excluded from quantitative pooling. The distribution of variance information sources used to derive standard errors is summarized in Table S10 in Multimedia Appendix 1. Subgroup differences were assessed using HKSJ-adjusted omnibus tests. In multivariable meta-regression, category-specific coefficients were assessed using HKSJ-adjusted *t* tests, global moderator effects were assessed using HKSJ-adjusted omnibus *F* tests, and complete cases were used for all included moderators. Small-study effects and funnel plot asymmetry were examined using funnel plots, Egger regression test, and trim-and-fill analysis when at least 10 effect sizes were available [14,15]. Sensitivity analyses were restricted to studies rated as low overall risk of bias.

Statistical analyses were performed using R (version 4.5.3; R Foundation for Statistical Computing) with the metafor package.

### Ethical Considerations

Not applicable because this study is a systematic review and meta-analysis of published studies.

## Results

### Basic Characteristics

**Figure 1.**
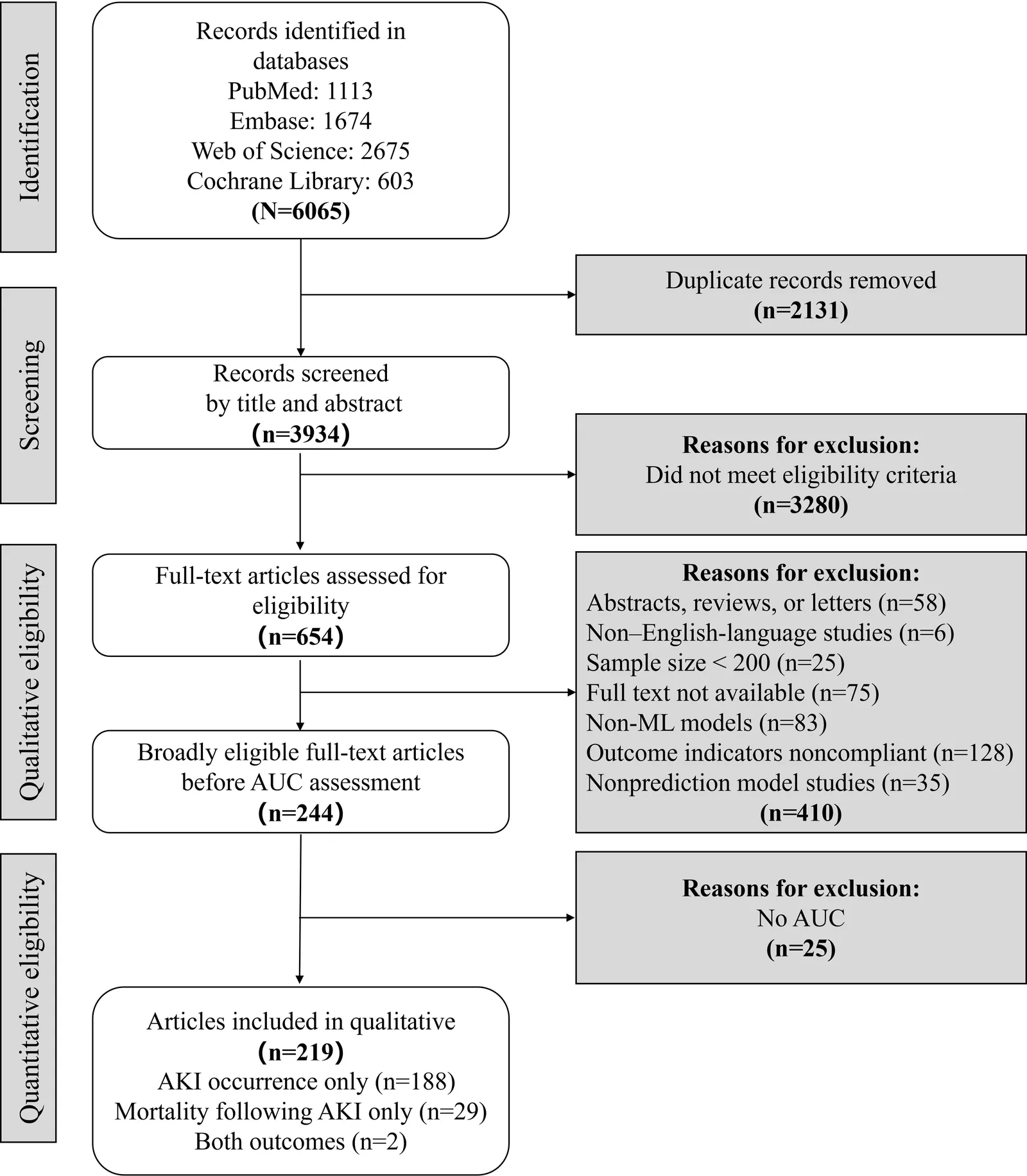
Flow diagram of study identification, screening, full-text assessment, and inclusion in the synthesis. Reasons for exclusion at the full-text stage are shown. AKI: acute kidney injury; AUC: area under the receiver operating characteristic curve; ML: machine learning.

Figure 1 shows the study selection flowchart according to the PRISMA statement. A total of 219 studies involving 7,343,170 patients were included in this review. The basic characteristics of each included study are detailed in Table S5 in Multimedia Appendix 1, with full references provided in Table S8 in Multimedia Appendix 1. Full references for excluded full-text articles and detailed reasons for exclusion are provided in Table S9 in Multimedia Appendix 1.

The characteristics of the included studies and prediction models are summarized in Table 1. This review included 219 studies and 101 distinct prediction models or modeling approaches. Overall, 188 outcome-specific model records addressed AKI occurrence prediction and 31 addressed post-AKI mortality prediction; 2 studies reported both outcomes.

**Table 1.** Characteristics of prediction model records for acute kidney injury occurrence and post–acute kidney injury mortality^a^.

Characteristic	All model records	Linear/generalized linear models	Tree-based and ensemble methods	Deep learning models	Hybrid models and others
**AKI**^**b**^ **occurrence, n (%)**	190 (100)	62 (32.6)	94 (49.5)	11 (5.8)	23 (12.1)
Sample size, n	7,335,791	3,051,811	3,745,480	323,664	214,836
Study type, n (%)					
Prospective cohort study	14 (7.4)	8 (57.1)	6 (42.9)	0 (0)	0 (0)
Retrospective cohort study	176 (92.6)	54 (30.7)	88 (50.0)	11 (6.3)	23 (13.1)
Data source, n (%)					
In-hospital	146 (76.8)	51 (34.9)	67 (45.9)	9 (6.2)	19 (13.0)
Public databases	44 (23.2)	11 (25)	27 (61.4)	2 (4.6)	4 (9.1)
Dataset, n (%)					
External validation cohort	31 (16.3)	3 (9.7)	21 (67.7)	5 (16.1)	2 (6.5)
Internal validation cohort	66 (34.7)	36 (54.6)	21 (31.8)	4 (6.1)	5 (7.6)
Other or unknown	31 (16.3)	14 (45.2)	12 (38.7)	0 (0)	5 (16.1)
Test cohort	41 (21.6)	4 (9.8)	26 (63.4)	2 (4.9)	9 (22.0)
Training cohort	21 (11.1)	5 (23.8)	14 (66.7)	0 (0)	2 (9.5)
External validation cohort, n (%)					
Yes	31 (16.3)	3 (9.7)	21 (67.7)	5 (16.1)	2 (6.5)
No	159 (83.7)	59 (37.1)	73 (45.9)	6 (3.8)	21 (13.2)
Internal validation cohort, n (%)					
Yes	87 (45.8)	38 (43.7)	34 (39.1)	9 (10.4)	6 (6.9)
No	103 (54.2)	24 (23.3)	60 (58.3)	2 (1.9)	17 (16.5)
Clinical application scenario, n (%)					
Early risk stratification	75 (39.5)	29 (38.7)	38 (50.7)	4 (5.3)	4 (5.3)
Perioperative monitoring	68 (35.8)	17 (25.0)	35 (51.5)	3 (4.4)	13 (19.1)
ICU^c^ real-time alert	15 (7.9)	6 (40.0)	8 (53.3)	1 (6.7)	0 (0)
Specialty-specific	32 (16.8)	10 (31.3)	13 (40.6)	3 (9.4)	6 (18.8)
Disease group, n (%)					
Cardiac and cardiac surgery–related	49 (25.8)	16 (32.7)	25 (51.0)	2 (4.1)	6 (12.3)
Sepsis- or infection-related	13 (6.8)	8 (61.5)	5 (38.5)	0 (0)	0 (0)
Severe acute pancreatitis	11 (5.8)	4 (36.4)	6 (54.6)	1 (9.1)	0 (0)
Liver disease–related	13 (6.8)	4 (30.8)	7 (53.9)	1 (7.7)	1 (7.7)
Nephrotoxic- or drug-related	17 (9.0)	6 (35.3)	6 (35.3)	2 (11.8)	3 (17.7)
Hypoperfusion- or shock-related	9 (4.7)	4 (44.4)	3 (33.3)	0 (0)	2 (22.2)
Trauma- or noncardiac surgery–related	18 (9.5)	3 (16.7)	10 (55.6)	1 (5.6)	4 (22.2)
Respiratory- or ARDS^d^-related	5 (2.6)	1 (20)	3 (60)	1 (20)	0 (0)
Hematologic- or malignancy-related	7 (3.7)	1 (14.3)	3 (42.9)	0 (0)	3 (42.9)
Cerebrovascular-related	4 (2.1)	2 (50)	2 (50)	0 (0)	0 (0)
Other or mixed	44 (23.2)	13 (29.6)	24 (54.6)	3 (6.8)	4 (9.1)
AKI frequency group, n (%)					
≤20%	75 (39.5)	25 (33.3)	36 (48.0)	5 (6.7)	9 (12.0)
>20% to ≤50%	85 (44.7)	26 (30.6)	42 (49.4)	6 (7.1)	11 (12.9)
>50%	23 (12.1)	9 (39.1)	12 (52.2)	0 (0)	2 (8.7)
Not reported	7 (3.7)	2 (28.6)	4 (57.1)	0 (0)	1 (14.3)
Data splitting and validation, n (%)					
Multicenter/external validation	44 (23.2)	10 (22.7)	25 (56.8)	6 (13.6)	3 (6.8)
Random split	102 (53.7)	30 (29.4)	53 (52.0)	5 (4.9)	14 (13.7)
Cross-validation	13 (6.8)	3 (23.1)	6 (46.2)	0 (0)	4 (30.8)
Other	15 (7.9)	6 (40)	7 (46.7)	0 (0)	2 (13.3)
Not reported	16 (8.4)	13 (81.3)	3 (18.8)	0 (0)	0 (0)
Overall risk of bias, n (%)					
Low	107 (56.3)	27 (25.2)	57 (53.3)	9 (8.4)	14 (13.1)
High	77 (40.5)	32 (41.6)	34 (44.2)	2 (2.6)	9 (11.7)
No information	6 (3.2)	3 (50)	3 (50)	0 (0)	0 (0)
**Post-AKI mortality, n (%)**	31 (100)	7 (22.6)	21 (67.7)	0 (0)	3 (9.7)
Sample size, n	417,999	18,755	260,841	0	138,403
Study type, n (%)					
Prospective cohort study	2 (6.5)	0 (0)	2 (100.0)	0 (0)	0 (0)
Retrospective cohort study	29 (93.5)	7 (24.1)	19 (65.5)	0 (0)	3 (10.4)
Data source, n (%)					
In-hospital	14 (45.2)	5 (35.7)	8 (57.1)	0 (0)	1 (7.1)
Public databases	17 (54.8)	2 (11.8)	13 (76.5)	0 (0)	2 (11.8)
Dataset, n (%)					
External validation cohort	9 (29.0)	1 (11.1)	6 (66.7)	0 (0)	2 (22.2)
Internal validation cohort	10 (32.3)	3 (30)	7 (70)	0 (0)	0 (0)
Other or unknown	4 (12.9)	1 (25)	3 (75)	0 (0)	0 (0)
Test cohort	4 (12.9)	1 (25)	2 (50)	0 (0)	1 (25)
Training cohort	4 (12.9)	1 (25)	3 (75)	0 (0)	0 (0)
External validation cohort, n (%)					
Yes	9 (29.0)	1 (11.1)	6 (66.7)	0 (0)	2 (22.2)
No	22 (71.0)	6 (27.3)	15 (68.2)	0 (0)	1 (4.6)
Internal validation cohort, n (%)					
Yes	15 (48.4)	3 (20)	10 (66.7)	0 (0)	2 (13.3)
No	16 (51.6)	4 (25)	11 (68.8)	0 (0)	1 (6.3)
Clinical application scenario, n (%)					
Early risk stratification	19 (61.3)	4 (21.1)	14 (73.7)	0 (0)	1 (5.3)
Perioperative monitoring	3 (9.7)	0 (0)	3 (100)	0 (0)	0 (0)
ICU real-time alert	7 (22.6)	3 (42.9)	3 (42.9)	0 (0)	1 (14.3)
Specialty-specific	2 (6.5)	0 (0)	1 (50)	0 (0)	1 (50)
Disease group, n (%)					
Cardiac and cardiac surgery–related	1 (3.2)	0 (0)	1 (100)	0 (0)	0 (0)
Sepsis- or infection–related	11 (35.5)	2 (18.2)	9 (81.8)	0 (0)	0 (0)
Severe acute pancreatitis	1 (3.2)	0 (0)	1 (100)	0 (0)	0 (0)
Liver disease–related	1 (3.2)	0 (0)	0 (0)	0 (0)	1 (100)
Hypoperfusion/shock-related	1 (3.2)	1 (100)	0 (0)	0 (0)	0 (0)
Trauma- or noncardiac surgery–related	2 (6.5)	0 (0)	2 (100)	0 (0)	0 (0)
Respiratory- or ARDS-related	1 (3.2)	0 (0)	1 (100)	0 (0)	0 (0)
Hematologic- or malignancy-related	1 (3.2)	0 (0)	1 (100)	0 (0)	0 (0)
Other or mixed	12 (38.7)	4 (33.3)	6 (50)	0 (0)	2 (16.7)
Post-AKI mortality frequency group, n (%)					
≤20%	11 (35.5)	2 (18.2)	8 (72.7)	0 (0)	1 (9.1)
>20% to ≤50%	15 (48.4)	4 (26.7)	10 (66.7)	0 (0)	1 (6.7)
>50%	4 (12.9)	1 (25)	2 (50)	0 (0)	1 (25)
Not reported	1 (3.2)	0 (0)	1 (100)	0 (0)	0 (0)
Data splitting and validation, n (%)					
Multicenter/external validation	13 (41.9)	3 (23.1)	8 (61.5)	0 (0)	2 (15.4)
Random split	15 (48.4)	3 (20)	11 (73.3)	0 (0)	1 (6.7)
Cross-validation	3 (9.7)	1 (33.3)	2 (66.7)	0 (0)	0 (0)
Overall risk of bias, n (%)					
Low	22 (71.0)	5 (22.7)	15 (68.2)	0 (0)	2 (9.1)
High	9 (29.0)	2 (22.2)	6 (66.7)	0 (0)	1 (11.1)

a Categorical variables are summarized as n (%), with percentages rounded to 1 decimal place. Sample size is reported as the cumulative number of participants across prediction models within each outcome category and AI model group. For categorical characteristics, percentages in the “All model records” column were calculated using the total number of model records for the corresponding outcome category as the denominator. Percentages in the AI model group columns were calculated using the total number of model records within each characteristic level as the denominator.

b AKI: acute kidney injury.

c ICU: intensive care unit.

d ARDS: acute respiratory distress syndrome.

For AKI occurrence prediction, tree-based and ensemble methods were the most commonly used approaches (94/190, 49.5%), followed by linear or generalized linear models (62/190, 32.6%), hybrid or other methods (23/190, 12.1%), and deep learning models (11/190, 5.8%). For post-AKI mortality prediction, tree-based and ensemble methods were also predominant (21/31, 67.7%), followed by linear or generalized linear models (7/31, 22.6%) and hybrid or other methods (3/31, 9.7%).

Most model records were based on retrospective cohorts, including 92.6% (176/190) of AKI occurrence records and 93.5% (29/31) of mortality records. AKI occurrence models mainly used in-hospital data (146/190, 76.8%), whereas mortality models more often used public databases (17/31, 54.8%). External validation was reported in 16.3% (31/190) of AKI occurrence records and 29.0% (9/31) of mortality records.

The cumulative sample size was 7,335,791 for AKI occurrence prediction and 417,999 for post-AKI mortality prediction. The main application scenarios for AKI occurrence prediction were early risk stratification (75/190, 39.5%) and perioperative monitoring (68/190, 35.8%), whereas mortality prediction models were mainly developed for early risk stratification (19/31, 61.3%).

Disease contexts were heterogeneous. AKI occurrence models were most often developed in cardiac or cardiac surgery-related populations, sepsis or infection-related cohorts, and trauma or noncardiac surgery settings, with smaller numbers addressing shock or hypoperfusion, respiratory failure or acute respiratory distress syndrome, liver disease, nephrotoxic exposures, severe acute pancreatitis, hematologic or malignancy-related conditions, cerebrovascular disease, and mixed populations. Mortality prediction models more commonly involved sepsis or infection-related cohorts and mixed clinical populations.

### Risk of Bias Assessment of Included Prediction Model Studies

Risk of bias assessment based on the PROBAST+AI tool showed that 58.9% (129/219) of included studies were rated as low overall risk of bias, 38.4% (84/219) as having high risk of bias, and 2.7% (6/219) as having unclear risk of bias. At the domain level, most studies were rated as low risk in participants and data sources, predictors, and outcomes. Potential bias was most frequent in the analysis domain, where 36.5% (80/219) of studies were rated as having high risk.

When stratified by outcome, 56.3% (107/190) of AKI occurrence prediction records were at low overall risk of bias, 40.5% (77/190) were at high risk, and 3.2% (6/190) had unclear or insufficient information. For post-AKI mortality prediction, 71.0% (22/31) of records were at low risk of bias, and 29.0% (9/31) were at high risk. Because 2 studies contributed to both outcome strata, their evaluation results were counted in both the AKI occurrence subgroup and the post-AKI mortality subgroup. Graphical summaries and traffic-light plots are provided in Figure S1 in Multimedia Appendix 1.

### Pooled AUC Performance Assessment and Subgroup Analysis

Using random-effects meta-analysis with HKSJ-adjusted inference, the pooled AUC for AKI occurrence prediction was 0.834 (95% CI 0.821‐0.846; *k*=188; *I*²=100.0%), with a 95% prediction interval (PI) of 0.602 to 0.943. AUC estimates from individual studies ranged from 0.654 to 0.995. The forest plot for the pooled AUC analysis of AKI occurrence prediction models is presented in Figure S2 in Multimedia Appendix 1. For post-AKI mortality prediction, the pooled AUC was 0.830 (95% CI 0.807‐0.851; *k*=31; *I*²=99.0%), with a 95% PI of 0.675 to 0.920. Individual study-level AUC estimates ranged from 0.720 to 0.943. The corresponding forest plot is shown in Figure S3 in Multimedia Appendix 1.

Substantial between-study heterogeneity was observed in both analyses. We therefore conducted subgroup analyses to compare pooled AUCs across predefined study-level and model-level characteristics and to explore potential sources of between-study heterogeneity.

We stratified discrimination performance according to AI model application type (Table 2). Using HKSJ-adjusted random-effects models, deep learning models had the highest pooled AUC point estimate for AKI occurrence prediction (0.867; 95% CI 0.797‐0.915), followed by tree-based and ensemble methods (0.851; 95% CI 0.833‐0.868), hybrid models and other approaches (0.827; 95% CI 0.788‐0.860), and linear or generalized linear models (0.797; 95% CI 0.776‐0.816). For post-AKI mortality prediction, hybrid models and other approaches had the highest pooled AUC point estimate (0.858; 95% CI 0.542‐0.969), followed by tree-based and ensemble methods (0.831; 95% CI 0.803‐0.855) and linear or generalized linear models (0.814; 95% CI 0.747‐0.866).

**Table 2.** Pooled area under the receiver operating characteristic curve by AI model application type using Hartung-Knapp-Sidik-Jonkman–adjusted inference^a^.

AI application group	AUC^b^ estimates, n	Pooled AUC	HKSJ^c^-adjusted 95% CI	95% prediction interval
AKI^d^ occurrence				
Linear/generalized linear models	62	0.797	0.776‐0.816	0.644‐0.894
Tree-based and ensemble methods	92	0.851	0.833‐0.868	0.612‐0.954
Deep learning models	11	0.867	0.797‐0.915	0.535‐0.974
Hybrid models and others	23	0.827	0.788‐0.860	0.583‐0.942
Post-AKI mortality				
Linear/generalized linear models	7	0.814	0.747‐0.866	0.618‐0.922
Tree-based and ensemble methods	21	0.831	0.803‐0.855	0.678‐0.920
Hybrid models and others	3	0.858	0.542‐0.969	0.194‐0.993

a The number of AUC estimates refers only to records eligible for quantitative pooling. For AKI occurrence prediction, 188 of 190 records were pooled because two lacked derivable variance estimates; all 31 post-AKI mortality records were included. Random-effects meta-analyses were performed on logit-transformed AUCs using restricted maximum likelihood estimation of between-study variance. Confidence intervals were calculated using the Hartung-Knapp-Sidik-Jonkman adjustment and back-transformed to the AUC scale. Prediction intervals were derived from the corresponding random-effects models and back-transformed to the AUC scale.

b AUC: area under the receiver operating characteristic curve.

c HKSJ: Hartung-Knapp-Sidik-Jonkman.

d AKI: acute kidney injury.

**Figure 2.**
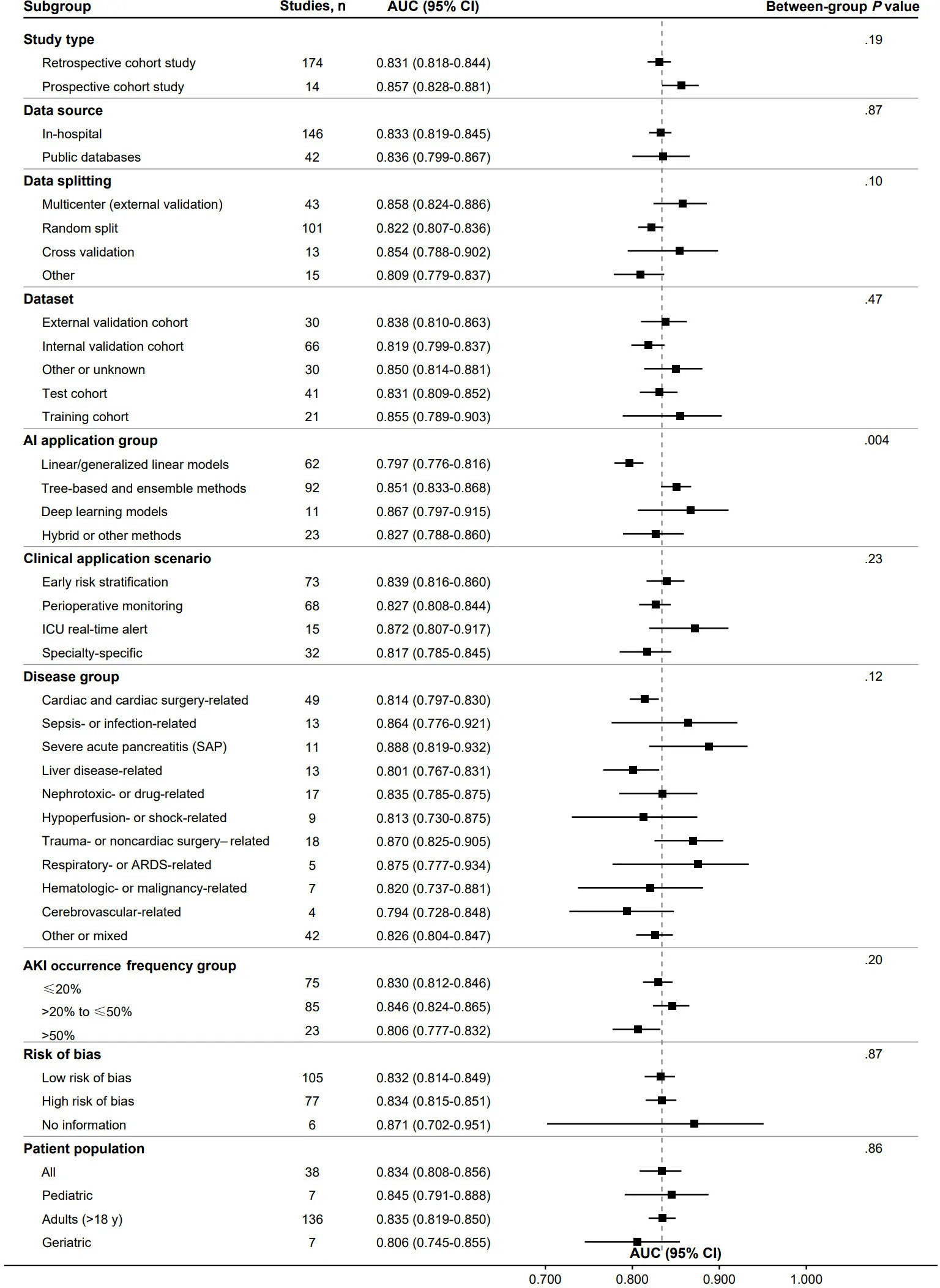
Subgroup analysis of pooled AUC for acute kidney injury occurrence prediction. Pooled AUCs and 95% CIs were estimated using random-effects models on the logit-transformed AUC scale with restricted maximum likelihood estimation and Hartung-Knapp-Sidik-Jonkman–adjusted inference. Between-group *P* values were based on Hartung-Knapp-Sidik-Jonkman–adjusted omnibus tests. AKI: acute kidney injury; ARDS: acute respiratory distress syndrome; AUC: area under the receiver operating characteristic curve; ICU: intensive care unit.

**Figure 3.**
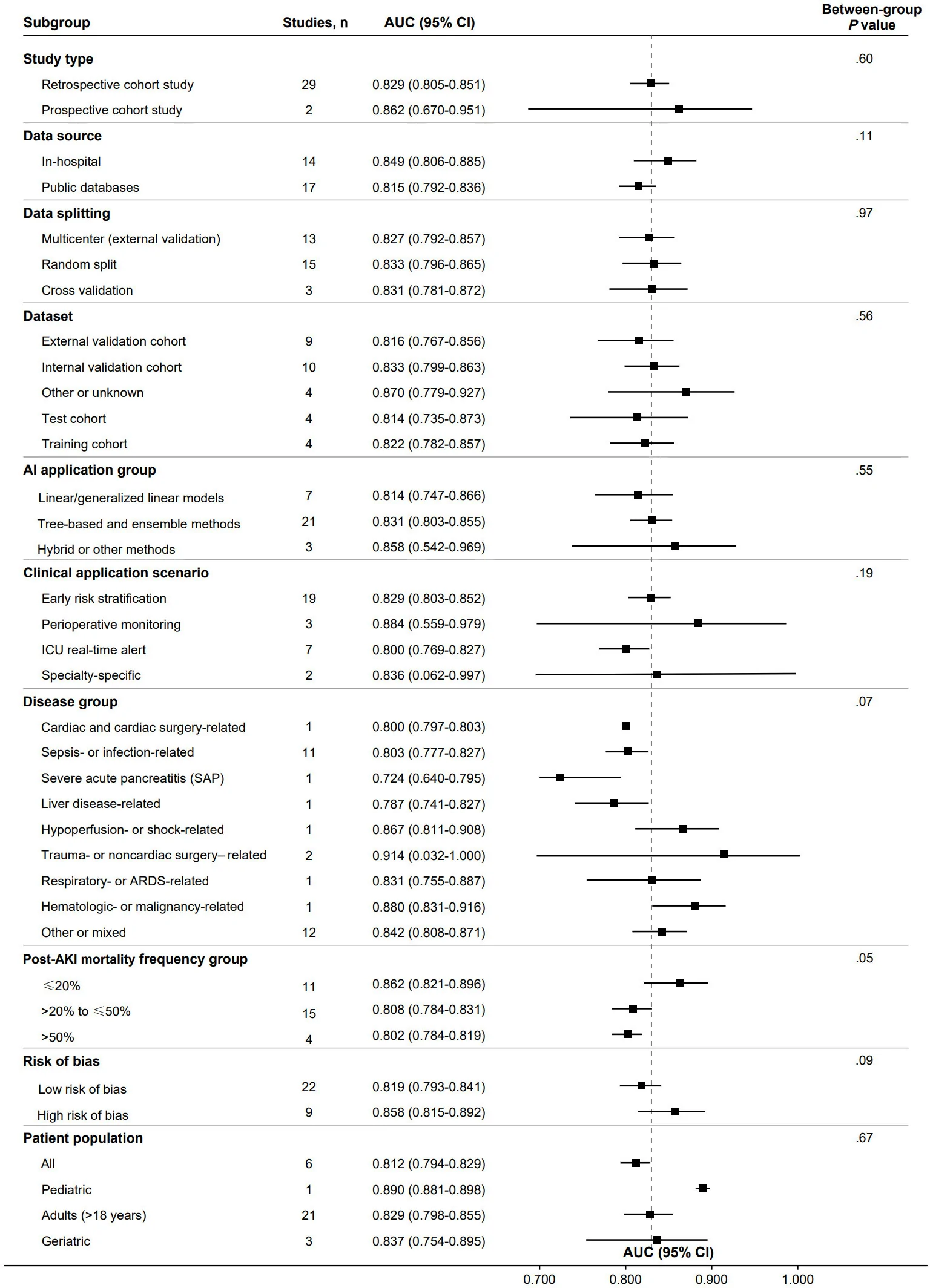
Subgroup analysis of pooled AUC for post-AKI mortality prediction. Pooled AUCs and 95% CIs were estimated using random-effects models on the logit-transformed AUC scale with restricted maximum likelihood estimation and Hartung-Knapp-Sidik-Jonkman–adjusted inference. Between-group *P* values were based on Hartung-Knapp-Sidik-Jonkman–adjusted omnibus tests. AKI: acute kidney injury; ARDS: acute respiratory distress syndrome; AUC: area under the receiver operating characteristic curve; ICU: intensive care unit.

Subgroup analyses of pooled AUC for AKI occurrence and post-AKI mortality prediction models are shown in Figures 2 and 3, respectively. AI model application type was associated with discrimination performance for AKI occurrence prediction (*F*_3,184_=4.6891; *P*=.004). Nonlinear approaches, particularly deep learning and tree-based and ensemble methods, generally showed higher pooled AUC point estimates than linear or generalized linear models. Subgroup differences by clinical application scenario, disease group, and AKI frequency group were not statistically significant for AKI occurrence prediction (*P*=.23, *P*=.12, and *P*=.20, respectively).

For post-AKI mortality prediction, subgroup differences by AI model application type were not statistically significant (*P*=.55). In-hospital data showed a numerically higher pooled AUC point estimate than public databases (0.849 vs 0.815), but the between-group difference by data source did not reach statistical significance (*P*=.11). Subgroup differences by clinical application scenario and disease group were also not statistically significant (*P*=.19 and *P*=.07, respectively). Only post-AKI mortality frequency group showed a borderline subgroup-level difference (*P*=.05). Complementary model-level analyses including all available model records yielded lower pooled AUCs than the representative-estimate analyses for both outcomes and are reported in Table S7 in Multimedia Appendix 1 and Figures S4 and S5 in Multimedia Appendix 1.

### Meta-Regression Analysis

In multivariable meta-regression using complete cases for all included moderators (Table 3), AI application group and disease group were associated with logit-transformed AUC for AKI occurrence prediction. The global test for AI application group was statistically significant (*P*=.02). Compared with deep learning models, linear or generalized linear models had lower logit-transformed AUCs (coefficient=−0.526; 95% CI −0.982 to −0.070; *P*=.02). Disease group was also associated with logit-transformed AUC in the global test (*P*=.04), with trauma- or noncardiac surgery–related models showing higher logit-transformed AUCs compared with the reference disease group (coefficient=0.598; 95% CI 0.187 to 1.010; *P*=.005). Data-splitting strategy showed a borderline global association (*P*=.05). Data source, dataset type, clinical application scenario, AKI frequency group, risk of bias, and patient population were not significantly associated with logit-transformed AUC in the global tests.

For post-AKI mortality prediction, no moderator showed a statistically significant global association in the multivariable meta-regression. Data-splitting strategy (*P*=.16), dataset (*P*=.30), AI application group (*P*=.21), clinical application scenario (*P*=.52), disease group (*P*=.26), AKI frequency group (*P*=.81), risk of bias (*P*=.45), and patient population (*P*=.44) were not significantly associated with logit-transformed AUC in the global tests. A forest plot summarizing all multivariable meta-regression coefficients with 95% CIs is provided in Figure S6 in Multimedia Appendix 1.

**Table 3.** Multivariable meta-regression analysis of factors associated with logit-transformed area under the receiver operating characteristic curve using Hartung-Knapp-Sidik-Jonkman–adjusted inference^a^.

Variables	AKI^b^ occurence		Post-AKI mortality	
	Coefficient (95% CI)	HKSJ^c^-adjusted *P* value	Coefficient (95% CI)	HKSJ-adjusted *P* value
Study type		.14		.30
Retrospective cohort study	−0.330 (−0.770 to 0.109)	.14	−0.462 (−1.548 to 0.624)	.30
Data source		.85		.65
Public databases	−0.029 (−0.317 to 0.260)	.85	0.198 (−0.910 to 1.305)	.65
Data splitting		.05		.16
Multicenter (external validation)	0.179 (−0.379 to 0.738)	.53	1.068 (−0.758 to 2.894)	.18
Other	−0.251 (−0.834 to 0.332)	.40	—^d^	—
Random split	−0.254 (−0.775 to 0.268)	.34	0.599 (−1.645 to 2.843)	.50
Dataset		.14		.30
Internal validation cohort	0.371 (−0.028 to 0.770)	.07	−0.060 (−0.927 to 0.807)	.86
Other or unknown	0.573 (0.032 to 1.115)	.04	1.046 (−0.498 to 2.589)	.13
Test cohort	0.453 (0.044 to 0.862)	.03	−0.074 (−1.071 to 0.922)	.85
Training cohort	0.557 (0.094 to 1.021)	.02	0.543 (−0.313 to 1.399)	.15
AI application group		.02		.21
Hybrid models and others	−0.228 (−0.721 to 0.265)	.36	—	—
Linear/generalized linear models	−0.526 (−0.982 to −0.070)	.02	−1.042 (−2.439 to 0.356)	.11
Tree-based and ensemble methods	−0.112 (−0.524 to 0.299)	.59	−1.020 (−2.814 to 0.774)	.19
Clinical application scenario		.64		.52
ICU^e^ real-time alert	−0.207 (−1.145 to 0.732)	.66	−0.441 (−1.736 to 0.854)	.40
Perioperative monitoring	−0.263 (−0.812 to 0.285)	.34	−0.093 (−2.242 to 2.056)	.91
Specialty-specific	−0.011 (−0.526 to 0.504)	.97	−1.152 (−4.311 to 2.008)	.37
Disease group		.04		.26
Cerebrovascular-related	−0.131 (−0.945 to 0.683)	.75	—	—
Hematologic- or malignancy-related	−0.266 (−0.870 to 0.339)	.39	2.587 (−2.955 to 8.130)	.27
Hypoperfusion- or shock-related	−0.041 (−0.741 to 0.659)	.91	1.658 (−0.835 to 4.151)	.14
Liver disease–related	−0.124 (−0.603 to 0.355)	.61	—	—
Nephrotoxic- or drug-related	−0.036 (−0.532 to 0.460)	.89	—	—
Other or mixed	−0.202 (−0.770 to 0.366)	.48	0.128 (−1.536 to 1.792)	.84
Respiratory- or ARDS^f^-related	0.470 (−0.411 to 1.352)	.29	0.317 (−2.393 to 3.027)	.76
Sepsis- or infection-related	0.396 (−0.272 to 1.064)	.24	−0.114 (−1.995 to 1.767)	.88
Severe acute pancreatitis	0.596 (−0.472 to 1.664)	.27	—	—
Trauma- or noncardiac surgery–related	0.598 (0.187 to 1.010)	.005	1.225 (−1.046 to 3.497)	.21
AKI frequency group		.34		.81
>20% to ≤50%	0.156 (−0.101 to 0.414)	.23	0.197 (−0.659 to 1.054)	.56
>50%	−0.023 (−0.426 to 0.380)	.91	0.299 (−1.193 to 1.790)	.61
Risk of bias		.54		.45
Low bias	0.093 (−0.143 to 0.328)	.44	0.305 (−0.702 to 1.312)	.45
No information	0.282 (−0.327 to 0.892)	.36	—	—
Patient population		.35		.44
All	−0.160 (−0.420 to 0.100)	.23	−0.361 (−1.547 to 0.824)	.45
Geriatric	−0.183 (−0.713 to 0.346)	.50	0.442 (−0.454 to 1.338)	.24
Pediatric	0.284 (−0.222 to 0.791)	.27	0.889 (−1.155 to 2.933)	.29

a Multivariable meta-regression was performed using logit-transformed area under the receiver operating characteristic curve as the dependent variable. Models were fitted using restricted maximum likelihood random-effects meta-regression. Confidence intervals and category-specific *P* values were calculated using Hartung-Knapp-Sidik-Jonkman adjusted *t* tests. Global moderator *P* values are shown on variable-heading rows and were calculated using HKSJ-adjusted omnibus *F* tests. Multivariable models used complete cases for all included moderators. Reference categories were prospective cohort study for study type, in-hospital data for data source, cross-validation for data splitting, external validation cohort for dataset type, deep learning models for AI application group in AKI occurrence, hybrid models and others for AI application group in post-AKI mortality, early risk stratification for clinical application scenario, cardiac/cardiac surgery–related disease group, AKI frequency ≤20%, high risk of bias, and adult population. Blank cells indicate reference categories or nonestimable categories due to sparse data or absence of that category in the corresponding outcome group.

b AKI: acute kidney injury.

c HKSJ: Hartung-Knapp-Sidik-Jonkman.

d Not available.

e ICU: intensive care unit.

f ARDS: acute respiratory distress syndrome.

### Sensitivity Analysis Restricted to Studies at Low Risk of Bias

In sensitivity analyses restricted to studies at low overall risk of bias, 105 AKI occurrence prediction records and 22 post-AKI mortality prediction records were included. The pooled AUCs were similar to the main analyses: 0.832 (95% CI 0.814‐0.849) for AKI occurrence prediction and 0.819 (95% CI 0.792‐0.843) for post-AKI mortality prediction. Heterogeneity remained high, with *I*² values of 100.0% and 97.4%, respectively. The corresponding forest plots are presented in Figure S7 in Multimedia Appendix 1.

### Assessment of Small-Study Effects and Funnel Plot Asymmetry

**Figure 4.**
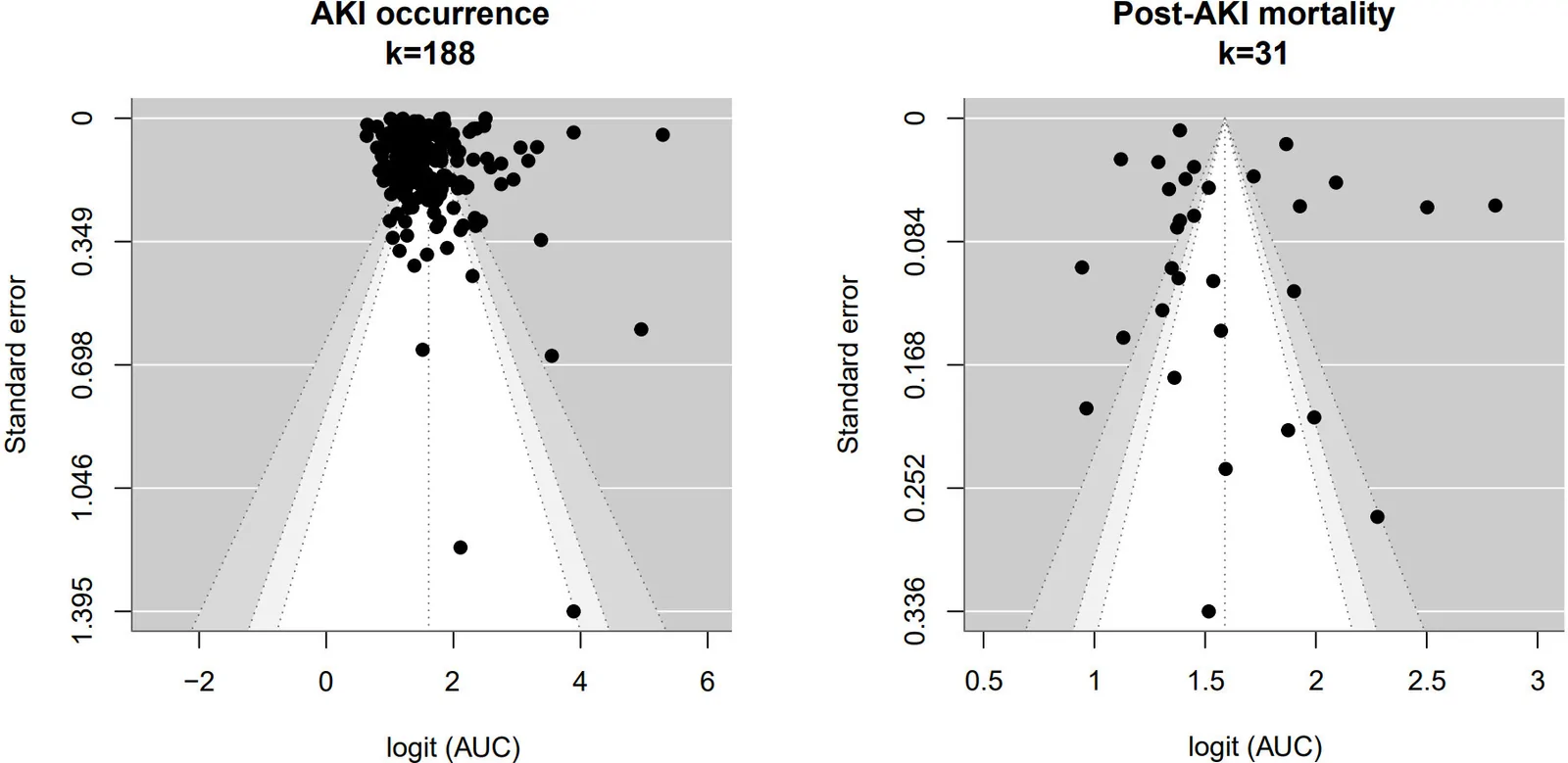
Funnel plots for assessing small-study effects in logit-transformed AUCs for AKI occurrence and post-AKI mortality prediction models.

Small-study effects and funnel plot asymmetry were examined using funnel plots, Egger regression test, and the trim-and-fill method (Figure 4). For AKI occurrence prediction models, visual inspection of the funnel plot suggested asymmetry, and Egger regression test was statistically significant among 188 effect sizes (test statistic=2.814; *P*=.005). However, the trim-and-fill method did not impute potentially missing studies. These findings suggest small-study effects or funnel plot asymmetry. For post-AKI mortality prediction models, there was no clear evidence of small-study effects: the funnel plot appeared broadly symmetrical, Egger regression test was not significant among 31 effect sizes (test statistic=0.062; *P*=.95), and trim-and-fill did not identify missing studies.

## Discussion

### Principal Findings

In this large-scale systematic review and meta-analysis of ML models for AKI prediction, analyses using logit-transformed AUCs, restricted maximum likelihood random-effects models, and HKSJ-adjusted inference showed high average discrimination for both AKI occurrence and post-AKI mortality. The pooled AUCs were 0.834 for AKI occurrence prediction and 0.830 for post-AKI mortality prediction. However, substantial heterogeneity and several sparse subgroup categories mean that between-group findings should be interpreted cautiously and as exploratory rather than confirmatory. These results support the potential value of ML-based approaches for AKI risk stratification and prognostic assessment, while also highlighting the need to interpret performance within specific clinical settings, validation designs, and implementation contexts.

Although the pooled AUCs were high, they should be interpreted as estimates of average discrimination across a clinically and methodologically diverse evidence base, rather than as guaranteed performance in any single institution or patient population. This interpretation is consistent with guidance for meta-analyses and external validation of prediction models, in which differences in case mix, outcome definitions, prediction horizons, and validation designs may strongly influence apparent model performance [16,17]. Future studies should therefore report discrimination together with calibration, clinically meaningful thresholds, decision-analytic measures, and transparent reporting according to contemporary prediction model guidance [18-20].

For AKI prediction, clinical usefulness depends on whether risk estimates are early enough, reliable enough, and actionable enough to change care [4,21]. A clinically useful model should be linked to predefined kidney-protective actions, such as reassessing nephrotoxic medications, optimizing hemodynamics and fluid balance, increasing creatinine and urine output monitoring, reviewing contrast exposure, or prompting earlier nephrology consultation. Without such links to workflow and action, even a well-discriminating model may remain a retrospective ranking tool rather than an AKI clinical decision-support intervention.

For AKI occurrence prediction, AI model class was associated with discrimination performance in both subgroup analysis and multivariable meta-regression using HKSJ-adjusted inference. Linear or generalized linear models showed lower logit-transformed AUCs than deep learning models in multivariable meta-regression, while nonlinear approaches generally showed higher pooled AUC point estimates in subgroup analyses. This pattern is biologically and clinically plausible, given the multifactorial nature of AKI. However, wide PIs, uneven subgroup sizes, and high residual heterogeneity indicate that these findings should not be interpreted as evidence that any model family is universally superior across clinical settings. For AKI clinical decision support, better discrimination alone is not enough to justify the adoption of more complex models unless they provide calibrated, timely, interpretable, and actionable predictions across institutions.

Subgroup analyses did not show statistically significant differences in pooled AUC across the 4 clinical application scenarios. However, this should not be interpreted as evidence of equivalent clinical usefulness. Early risk stratification, perioperative monitoring, ICU real-time alerting, and specialty-specific prediction differ in prediction timing, data requirements, intervention opportunities, and consequences of false-positive or false-negative alerts. This nonsignificant result may arise because AUC has limitations in evaluating clinical application and model calibration; it cannot effectively indicate performance differences among ML models under different clinical conditions. Thus, clinical application scenario remains important when evaluating implementation readiness.

For post-AKI mortality prediction, moderator findings were less stable than those for AKI occurrence prediction. In subgroup analyses, disease context was not statistically associated with pooled AUC, whereas AKI frequency group showed only a borderline subgroup-level difference. In multivariable meta-regression, no moderator showed a statistically significant global association with logit-transformed AUC for post-AKI mortality prediction. Given the small number of eligible mortality estimates and several sparse categories, these findings should be interpreted cautiously and considered exploratory rather than confirmatory. Mortality models may capture broader illness severity rather than AKI-specific prognosis alone, which may further contribute to heterogeneity in post-AKI mortality prediction.

The complementary model-level analysis yielded lower pooled AUCs than the representative-estimate analysis for both AKI occurrence and post-AKI mortality prediction. This difference may reflect the inclusion of lower-performing models reported within the same studies. Therefore, the representative-estimate analysis should be interpreted as study-level prioritized performance, whereas the model-level analysis provides a broader but more conservative summary of all reported model estimates.

Sensitivity analyses restricted to studies at low overall risk of bias yielded pooled AUCs similar to those in the main analysis, supporting the stability of the average discrimination estimates after excluding high-risk-of-bias studies. However, heterogeneity remained substantial, indicating that risk of bias was not the only source of between-study variability. Differences in model type, study population, data source, validation method, prediction horizon, and AKI definition may still have influenced the pooled results.

Evidence of small-study effects or funnel plot asymmetry was observed for AKI occurrence prediction but not for post-AKI mortality prediction. These findings should not be interpreted as direct evidence of publication bias. Publication bias represents only one possible explanation for small-study effects; other explanations include clinical and methodological heterogeneity, differences in validation strategies, outcome definitions, model-development practices, selective reporting, and chance.

Alert fatigue is an important consideration for the clinical implementation of ML-based AKI prediction models. Patients at AKI risk often have complex clinical profiles that already trigger frequent electronic health record alerts. Models with suboptimal specificity, calibration, or overly sensitive thresholds may produce excessive false-positive alerts, causing clinician desensitization and reduced responses to genuine AKI risks. Therefore, model performance should not be evaluated solely by AUROC-based discrimination. Future studies must additionally assess calibration, false-alert burden, clinical use, and workflow impact. To reduce alert fatigue, AKI prediction models require rigorous external validation, optimized thresholds, and integrated, actionable clinical workflows rather than standalone risk alerts.

### Strengths and Limitations

This review has several strengths. First, it provides one of the largest quantitative syntheses of ML models for AKI prediction to date [22-24]. Second, it analyzed AKI occurrence and post-AKI mortality separately, avoiding the combination of clinically distinct prediction tasks. Third, it examined discrimination performance according to both clinical application scenario and model class, which improves clinical interpretability. Fourth, it combined quantitative performance synthesis with PROBAST+AI-based risk of bias assessment and validation characteristics.

Several limitations should be acknowledged. First, substantial heterogeneity was present across included studies. This was expected given differences in AKI definitions, prediction horizons, clinical settings, patient populations, data sources, validation strategies, and model classes. Therefore, pooled AUCs should be interpreted as average discrimination across a broad evidence base rather than as setting-specific expected performance.

Second, many models were developed and evaluated using retrospective data, whereas external or prospective validation was relatively limited. This limits confidence in their transportability across different institutions, health care systems, and patient populations. Therefore, further external and prospective validation is needed before these models can be considered for broader clinical implementation.

Third, most studies primarily reported discrimination, commonly using AUC, whereas other important aspects of model performance, such as calibration and clinical usefulness, were less consistently reported. These are not only limitations of this review but also important findings about the current AKI prediction literature. The inconsistent reporting of calibration, thresholds, decision-analytic evaluation, and clinical impact prevented quantitative synthesis of implementation outcomes and highlights the need for future studies to move beyond discrimination-centered reporting.

Fourth, our representative AUC selection rule may have introduced optimistic selection bias. To reduce within-study dependence, we selected one AUC per study and outcome according to a prespecified validation hierarchy. When several eligible AUCs were available within the same validation tier, the highest value was used because many studies did not clearly identify a primary model, prediction horizon, or clinically intended final model. This approach may have favored better-performing models and inflated the pooled AUCs. Accordingly, these estimates should be interpreted as study-level summaries rather than unbiased averages of all reported model results. The complementary model-level analysis, which included all retrievable AUCs, yielded lower pooled estimates but did not change the overall conclusions.

### Conclusions

ML models showed high average discrimination for both AKI occurrence and post-AKI mortality in this large-scale systematic review and meta-analysis. Performance patterns differed by model class and clinical prediction task, with nonlinear approaches showing relatively higher pooled AUC point estimates in exploratory analyses for AKI occurrence, although between-group findings should be interpreted cautiously. These findings support the potential value of ML for AKI risk stratification and prognostic assessment. However, heterogeneity across studies and limited reporting of external validation, calibration, thresholds, alert burden, and clinical utility indicate that future research should move from discrimination-centered model development toward validated, calibrated, and clinically actionable AKI decision-support tools.

## Supplementary material

10.2196/104269 Multimedia Appendix 1 Tables and figures required to interpret the primary study results.

10.2196/104269 Checklist 1 PRISMA 2020 checklist.

## Abbreviations

AKI: acute kidney injury
AUC: area under the receiver operating characteristic curve
HKSJ: Hartung-Knapp-Sidik-Jonkman
ICU: intensive care unit
LightGBM: Light Gradient Boosting Machine
ML: machine learning
PI: prediction interval
PRISMA: Preferred Reporting Items for Systematic Reviews and Meta-Analyses
PROBAST+AI: Prediction Model Risk of Bias Assessment Tool+Artificial Intelligence
PROSPERO: International Prospective Register of Systematic Reviews
XGBoost: Extreme Gradient Boosting
