# Supplementary material for: AI Models for Predicting Acute Kidney Injury (AKI) and Post-AKI Mortality: Systematic Review and Meta-Analysis
Source: J Med Internet Res. 2026 Aug 20;28:e104269. doi: 10.2196/104269 (PMC13492631; doi:10.2196/104269)

Contents

Supplementary appendix	2
Supplementary Table S8. Full references of studies included in qualitative synthesis	3
Supplementary Table S9. Full references of excluded full-text articles and reasons for exclusion	12
Supplementary Table S1. Literature Search Strategy	37
Supplementary Table S2. Details of Extracted Variables	38
Supplementary Table S3 and S4. PROBAST+AI Signaling Questions	40
Supplementary Table S5. Study-Level Characteristics of Included Studies and AKI Prediction Models	42
Supplementary Table S6. Study-Level PROBAST+AI Assessment of Risk of Bias, Reporting Quality, and Applicability	73
Supplementary Table S7. Complementary Model-Level Meta-analysis of Pooled AUCs by AI Model Application Type	87
Supplementary Table S10. Sources of variance information used for AUC estimates included in quantitative pooling	89
Supplementary Figure S1. Risk-of-Bias Assessment Results Using the PROBAST+AI Tool	90
Supplementary Figure S2. Forest Plot for Meta-analysis of AUCs of AKI Occurrence Prediction Models	102
Supplementary Figure S3. Forest Plot for Meta-analysis of AUCs of Mortality Following AKI Prediction Models	108
Supplementary Figure S4. Complementary Model-Level Subgroup Meta-analysis for AKI Occurrence	109
Supplementary Figure S5. Complementary Model-Level Subgroup Meta-analysis for Mortality Following AKI	110
Supplementary Figure S6. Multivariable Meta-regression Coefficients	111
Supplementary Figure S7. Low Risk-of-Bias Sensitivity Analyses	112

Supplementary Materials

Inclusion Criteria

Participants: Our study population includes older adults (aged 65 years and above), children, and adults with various diseases that may lead to acute kidney injury (AKI) or undergoing certain procedures that may induce AKI.

Intervention: Machine learning; Artificial intelligence.

Comparator: Not applicable in our study selection criteria. There is no universally accepted prognostic model for AKI outcomes.

Outcomes: The outcomes of interest were either the development of AKI (diagnosed according to KDIGO, RIFLE, AKIN, or other study-defined criteria) or all-cause mortality following AKI.

Explanation of Machine Learning and Deep Learning architectures:

Machine learning models were categorized into four mutually exclusive groups:

(1) Linear/generalized linear models (e.g., logistic regression, lasso, elastic net);

(2) Tree-based and ensemble methods, defined as models relying on decision trees as base learners, including both single trees and ensembles (bagging, boosting, stacking, voting) where all base learners are tree-based (e.g., random forest, XGBoost, LightGBM, gradient boosting);

(3) Deep learning models, defined as neural networks with at least one hidden layer, including convolutional, recurrent, and attention-based architectures (e.g., CNN, RNN, LSTM, MLP);

(4) Hybrid models and others, encompassing (a) models combining heterogeneous paradigms (e.g., CNN-LSTM, VAE with classifiers), (b) non-tree, non-deep algorithms (e.g., support vector machines, k-nearest neighbors, Bayesian classifiers), (c) generative models, graph neural networks, meta-learning, automated machine learning pipelines, and any clinically interactive or custom architectures not falling into the previous three categories.

Supplementary Table S8. Full references of studies included in qualitative synthesis (n = 219)

Note. References are presented in JMIR/AMA style. PubMed identifiers, PubMed Central identifiers, DOIs, and journal URLs are provided when available.

Record Number	Full reference in JMIR/AMA style	PMID	DOI or URL
4	Yue S, Li S, Huang X, et al. Machine learning for the prediction of acute kidney injury in patients with sepsis. J Transl Med. 2022;20(1):215.	35562803	doi:10.1186/s12967-022-03364-0
8	Fan Z, Jiang J, Xiao C, et al. Construction and validation of prognostic models in critically Ill patients with sepsis-associated acute kidney injury: interpretable machine learning approach. J Transl Med. 2023;21(1):406.	37349774	doi:10.1186/s12967-023-04205-4
9	Kate RJ, Pearce N, Mazumdar D, Nilakantan V. A continual prediction model for inpatient acute kidney injury. Comput Biol Med. 2020;116:103580.	32001013	doi:10.1016/j.compbiomed.2019.103580
11	Zhang W, Chang Y, Cheng C, et al. A machine learning model for predicting acute kidney injury secondary to severe acute pancreatitis. Chin Med J (Engl). 2024;137(5):619-621.	38317516	doi:10.1097/cm9.0000000000003027
12	Tseng PY, Chen YT, Wang CH, et al. Prediction of the development of acute kidney injury following cardiac surgery by machine learning. Crit Care. 2020;24(1):478.	32736589	doi:10.1186/s13054-020-03179-9
14	Luo XQ, Kang YX, Duan SB, et al. Machine Learning-Based Prediction of Acute Kidney Injury Following Pediatric Cardiac Surgery: Model Development and Validation Study. J Med Internet Res. 2023;25:e41142.	36603200	doi:10.2196/41142
15	Chen Q, Zhang Y, Zhang M, Li Z, Liu J. Application of Machine Learning Algorithms to Predict Acute Kidney Injury in Elderly Orthopedic Postoperative Patients. Clin Interv Aging. 2022;17:317-330.	35386749	doi:10.2147/cia.S349978
18	Cheng Y, Yang J, Wu Q, et al. Machine learning for the prediction of acute kidney injury in patients with acute pancreatitis admitted to the intensive care unit. Chin Med J (Engl). 2022;135(23):2886-2887.	36728600	doi:10.1097/cm9.0000000000002531
26	Gao T, Nong Z, Luo Y, et al. Machine learning-based prediction of in-hospital mortality for critically ill patients with sepsis-associated acute kidney injury. Ren Fail. 2024;46(1):2316267.	38369749	doi:10.1080/0886022x.2024.2316267
31	Lin S, Lu W, Wang T, et al. Predictive model of acute kidney injury in critically ill patients with acute pancreatitis: a machine learning approach using the MIMIC-IV database. Ren Fail. 2024;46(1):2303395.	38264967	doi:10.1080/0886022x.2024.2303395
48	Wei S, Zhang Y, Dong H, et al. Machine learning-based prediction model of acute kidney injury in patients with acute respiratory distress syndrome. BMC Pulm Med. 2023;23(1):370.	37789305	doi:10.1186/s12890-023-02663-6
77	Zhang R, Yin M, Jiang A, Zhang S, Xu X, Liu L. Automated machine learning for early prediction of acute kidney injury in acute pancreatitis. BMC Med Inform Decis Mak. 2024;24(1):16.	38212745	doi:10.1186/s12911-024-02414-5
78	Li X, Wang P, Zhu Y, Zhao W, Pan H, Wang D. Interpretable machine learning model for predicting acute kidney injury in critically ill patients. BMC Med Inform Decis Mak. 2024;24(1):148.	38822285	doi:10.1186/s12911-024-02537-9
79	She S, Shen Y, Luo K, Zhang X, Luo C. Prediction of Acute Kidney Injury in Intracerebral Hemorrhage Patients Using Machine Learning. Neuropsychiatr Dis Treat. 2023;19:2765-2773.	38106359	doi:10.2147/ndt.S439549
83	Shi J, Han H, Chen S, Liu W, Li Y. Machine learning for prediction of acute kidney injury in patients diagnosed with sepsis in critical care. PLoS One. 2024;19(4):e0301014.	38603693	doi:10.1371/journal.pone.0301014
87	Wang Y, Sun X, Lu J, Zhong L, Yang Z. Construction and evaluation of a mortality prediction model for patients with acute kidney injury undergoing continuous renal replacement therapy based on machine learning algorithms. Ann Med. 2024;56(1):2388709.	39155811	doi:10.1080/07853890.2024.2388709
90	Ryan CT, Zeng Z, Chatterjee S, et al. Machine learning for dynamic and early prediction of acute kidney injury after cardiac surgery. J Thorac Cardiovasc Surg. 2023;166(6):e551-e564.	36347651	doi:10.1016/j.jtcvs.2022.09.045
96	Kalisnik JM, Bauer A, Vogt FA, et al. Artificial intelligence-based early detection of acute kidney injury after cardiac surgery. Eur J Cardiothorac Surg. 2022;62(5).	35521994	doi:10.1093/ejcts/ezac289
98	Wang J, Yu C, Zhang Y, Huang Y. A Prediction Model for Acute Kidney Injury After Pericardiectomy: An Observational Study. Front Cardiovasc Med. 2022;9:790044.	35224038	doi:10.3389/fcvm.2022.790044
99	Kuno T, Mikami T, Sahashi Y, et al. Machine learning prediction model of acute kidney injury after percutaneous coronary intervention. Sci Rep. 2022;12(1):749.	35031637	doi:10.1038/s41598-021-04372-8
101	Cui H, Shu S, Li Y, et al. Plasma Metabolites-Based Prediction in Cardiac Surgery-Associated Acute Kidney Injury. J Am Heart Assoc. 2021;10(22):e021825.	34719239	doi:10.1161/jaha.121.021825
102	Zhou H, Liu L, Zhao Q, et al. Machine learning for the prediction of all-cause mortality in patients with sepsis-associated acute kidney injury during hospitalization. Front Immunol. 2023;14:1140755.	37077912	doi:10.3389/fimmu.2023.1140755
109	Ejmalian A, Aghaei A, Nabavi S, et al. Prediction of Acute Kidney Injury After Cardiac Surgery Using Interpretable Machine Learning. Anesth Pain Med. 2022;12(4):e127140.	36937087	doi:10.5812/aapm-127140

Record Number	Full reference in JMIR/AMA style	PMID	DOI or URL
136	Zhang L, Wang Z, Zhou Z, et al. Developing an ensemble machine learning model for early prediction of sepsis-associated acute kidney injury. iScience. 2022;25(9):104932.	36060071	doi:10.1016/j.isci.2022.104932
137	Hu C, Tan Q, Zhang Q, et al. Application of interpretable machine learning for early prediction of prognosis in acute kidney injury. Comput Struct Biotechnol J. 2022;20:2861-2870.	35765651	doi:10.1016/j.csbj.2022.06.003
140	Gong K, Lee HK, Yu K, Xie X, Li J. A prediction and interpretation framework of acute kidney injury in critical care. J Biomed Inform. 2021;113:103653.	33338667	doi:10.1016/j.jbi.2020.103653
141	Liu Y, Zhu X, Xue J, Maimaitituerxun R, Chen W, Dai W. Machine learning models for mortality prediction in critically ill patients with acute pancreatitis-associated acute kidney injury. Clin Kidney J. 2024;17(10):sfae284.	39385947	doi:10.1093/ckj/sfae284
153	Jiang J, Liu X, Cheng Z, Liu Q, Xing W. Interpretable machine learning models for early prediction of acute kidney injury after cardiac surgery. BMC Nephrol. 2023;24(1):326.	37936067	doi:10.1186/s12882-023-03324-w
157	Liu J, Xu L, Zhu E, Han C, Ai Z. Prediction of acute kidney injury in patients with femoral neck fracture utilizing machine learning. Front Surg. 2022;9:928750.	35959132	doi:10.3389/fsurg.2022.928750
158	Yan Y, Gong H, Hu J, et al. Perioperative parameters-based prediction model for acute kidney injury in Chinese population following valvular surgery. Front Cardiovasc Med. 2023;10:1094997.	36960471	doi:10.3389/fcvm.2023.1094997
163	Jia T, Xu K, Bai Y, et al. Machine-learning predictions for acute kidney injuries after coronary artery bypass grafting: a real-life muticenter retrospective cohort study. BMC Med Inform Decis Mak. 2023;23(1):270.	37996844	doi:10.1186/s12911-023-02376-0
164	Hu Y, Liu K, Ho K, et al. A Simpler Machine Learning Model for Acute Kidney Injury Risk Stratification in Hospitalized Patients. J Clin Med. 2022;11(19).	36233556	doi:10.3390/jcm11195688
169	Filiberto AC, Ozrazgat-Baslanti T, Loftus TJ, et al. Optimizing predictive strategies for acute kidney injury after major vascular surgery. Surgery. 2021;170(1):298-303.	33648766	doi:10.1016/j.surg.2021.01.030
170	Peng X, Li L, Wang X, Zhang H. A Machine Learning-Based Prediction Model for Acute Kidney Injury in Patients With Congestive Heart Failure. Front Cardiovasc Med. 2022;9:842873.	35310995	doi:10.3389/fcvm.2022.842873
173	Gao Y, Wang C, Dong W, et al. An Explainable Machine Learning Model to Predict Acute Kidney Injury After Cardiac Surgery: A Retrospective Cohort Study. Clin Epidemiol. 2023;15:1145-1157.	38076638	doi:10.2147/clep.S404580
174	Sun R, Li S, Wei Y, et al. Development of interpretable machine learning models for prediction of acute kidney injury after noncardiac surgery: a retrospective cohort study. Int J Surg. 2024;110(5):2950-2962.	38445452	doi:10.1097/js9.0000000000001237
175	Dong L, Liu P, Qi Z, Lin J, Duan M. Development and validation of a machine-learning model for predicting the risk of death in sepsis patients with acute kidney injury. Heliyon. 2024;10(9):e29985.	38699001	doi:10.1016/j.heliyon.2024.e29985
176	Zahr RS, Mohammed A, Naik S, et al. Machine Learning Predicts Acute Kidney Injury in Hospitalized Patients with Sickle Cell Disease. Am J Nephrol. 2024;55(1):18-24.	37906980	doi:10.1159/000534864
178	Ko S, Jo C, Chang CB, et al. A web-based machine-learning algorithm predicting postoperative acute kidney injury after total knee arthroplasty. Knee Surg Sports Traumatol Arthrosc. 2022;30(2):545-554.	32880677	doi:10.1007/s00167-020-06258-0
181	Song Y, Zhai W, Ma S, et al. Machine learning-based prediction of off-pump coronary artery bypass grafting-associated acute kidney injury. J Thorac Dis. 2024;16(7):4535-4542.	39144311	doi:10.21037/jtd-24-711
182	Pierre-Paul S, Wang XS, Mere C, Rungkitwattanakul D. Creating a Machine Learning Tool to Predict Acute Kidney Injury in African American Hospitalized Patients. Pharmacy (Basel). 2022;10(4).	35893706	doi:10.3390/pharmacy10040068
183	Tang J, Huang J, He X, et al. The prediction of in-hospital mortality in elderly patients with sepsis-associated acute kidney injury utilizing machine learning models. Heliyon. 2024;10(4):e26570.	38420451	doi:10.1016/j.heliyon.2024.e26570
185	Li X, Wu R, Zhao W, et al. Machine learning algorithm to predict mortality in critically ill patients with sepsis-associated acute kidney injury. Sci Rep. 2023;13(1):5223.	36997585	doi:10.1038/s41598-023-32160-z
186	Petrosyan Y, Mesana TG, Sun LY. Prediction of acute kidney injury risk after cardiac surgery: using a hybrid machine learning algorithm. BMC Med Inform Decis Mak. 2022;22(1):137.	35585624	doi:10.1186/s12911-022-01859-w
187	Qu C, Gao L, Yu XQ, et al. Machine Learning Models of Acute Kidney Injury Prediction in Acute Pancreatitis Patients. Gastroenterol Res Pract. 2020;2020:3431290.	33061958	doi:10.1155/2020/3431290
188	Yuan L, Ji M, Wang S, et al. Early prediction of acute pancreatitis with acute kidney injury using abdominal contrast-enhanced CT features. iScience. 2024;27(10):111058.	39435145	doi:10.1016/j.isci.2024.111058
189	Tong Y, Niu X, Liu F. Prediction of Acute Kidney Injury after Extracorporeal Cardiac Surgery (CSA-AKI) by Machine Learning Algorithms. Heart Surg Forum. 2023;26(5):E537-e551.	37920093	doi:10.59958/hsf.5673
192	Li Q, Lv H, Chen Y, Shen J, Shi J, Zhou C. Development and Validation of a Machine Learning Predictive Model for Cardiac Surgery-Associated Acute Kidney Injury. J Clin Med. 2023;12(3).	36769813	doi:10.3390/jcm12031166
199	Schwager E, Ghosh E, Eshelman L, Pasupathy KS, Barreto EF, Kashani K. Accurate and interpretable prediction of ICU-acquired AKI. J Crit Care. 2023;75:154278.	36774817	doi:10.1016/j.jcrc.2023.154278

Record Number	Full reference in JMIR/AMA style	PMID	DOI or URL
201	Lei G, Wang G, Zhang C, Chen Y, Yang X. Using Machine Learning to Predict Acute Kidney Injury After Aortic Arch Surgery. J Cardiothorac Vasc Anesth. 2020;34(12):3321-3328.	32636105	doi:10.1053/j.jvca.2020.06.007
207	Du ZX, Chang FQ, Wang ZJ, Zhou DM, Li Y, Yang JH. A risk prediction model for acute kidney injury in patients with pulmonary tuberculosis during anti-tuberculosis treatment. Ren Fail. 2022;44(1):625-635.	35373713	doi:10.1080/0886022x.2022.2058405
211	Fan R, Qin W, Zhang H, et al. Machine learning in the prediction of cardiac surgery associated acute kidney injury with early postoperative biomarkers. Front Surg. 2023;10:1048431.	36824496	doi:10.3389/fsurg.2023.1048431
212	Chiofolo C, Chbat N, Ghosh E, Eshelman L, Kashani K. Automated Continuous Acute Kidney Injury Prediction and Surveillance: A Random Forest Model. Mayo Clin Proc. 2019;94(5):783-792.	31054606	doi:10.1016/j.mayocp.2019.02.009
216	Thongprayoon C, Pattharanitima P, Kattah AG, et al. Explainable Preoperative Automated Machine Learning Prediction Model for Cardiac Surgery-Associated Acute Kidney Injury. J Clin Med. 2022;11(21).	36362493	doi:10.3390/jcm11216264
217	Lee Y, Ryu J, Kang MW, et al. Machine learning-based prediction of acute kidney injury after nephrectomy in patients with renal cell carcinoma. Sci Rep. 2021;11(1):15704.	34344909	doi:10.1038/s41598-021-95019-1
219	Zeng X, Shi S, Sun Y, et al. A time-aware attention model for prediction of acute kidney injury after pediatric cardiac surgery. J Am Med Inform Assoc. 2022;30(1):94-102.	36287639	doi:10.1093/jamia/ocac202
220	Yang Y, Xiao W, Liu X, Zhang Y, Jin X, Li X. Machine Learning-Assisted Ensemble Analysis for the Prediction of Acute Pancreatitis with Acute Kidney Injury. Int J Gen Med. 2022;15:5061-5072.	35607360	doi:10.2147/ijgm.S361330
222	Liu WT, Liu XQ, Jiang TT, et al. Using a machine learning model to predict the development of acute kidney injury in patients with heart failure. Front Cardiovasc Med. 2022;9:911987.	36176988	doi:10.3389/fcvm.2022.911987
226	Zhou F, Lu Y, Xu Y, et al. Correlation between neutrophil-to-lymphocyte ratio and contrast-induced acute kidney injury and the establishment of machine-learning-based predictive models. Ren Fail. 2023;45(2):2258983.	37755332	doi:10.1080/0886022x.2023.2258983
227	Fan T, Wang J, Li L, Kang J, Wang W, Zhang C. Predicting the risk factors of diabetic ketoacidosis-associated acute kidney injury: A machine learning approach using XGBoost. Front Public Health. 2023;11:1087297.	37089510	doi:10.3389/fpubh.2023.1087297
230	Ma M, Wan X, Chen Y, et al. A novel explainable online calculator for contrast-induced AKI in diabetics: a multi-centre validation and prospective evaluation study. J Transl Med. 2023;21(1):517.	37525240	doi:10.1186/s12967-023-04387-x
232	Kamel Rahimi A, Ghadimi M, van der Vegt AH, et al. Machine learning clinical prediction models for acute kidney injury: the impact of baseline creatinine on prediction efficacy. BMC Med Inform Decis Mak. 2023;23(1):207.	37814311	doi:10.1186/s12911-023-02306-0
233	Zhang X, Chen S, Lai K, Chen Z, Wan J, Xu Y. Machine learning for the prediction of acute kidney injury in critical care patients with acute cerebrovascular disease. Ren Fail. 2022;44(1):43-53.	35166177	doi:10.1080/0886022x.2022.2036619
236	Huang CT, Wang TJ, Kuo LK, et al. Federated machine learning for predicting acute kidney injury in critically ill patients: a multicenter study in Taiwan. Health Inf Sci Syst. 2023;11(1):48.	37822805	doi:10.1007/s13755-023-00248-5
237	Penny-Dimri JC, Bergmeir C, Reid CM, Williams-Spence J, Cochrane AD, Smith JA. Machine Learning Algorithms for Predicting and Risk Profiling of Cardiac Surgery-Associated Acute Kidney Injury. Semin Thorac Cardiovasc Surg. 2021;33(3):735-745.	32979479	doi:10.1053/j.semtcvs.2020.09.028
239	Luo Y, Ye W, Sun Y, Bao H, Liu H. Development and Comparative Analysis of an Early Prediction Model for Acute Kidney Injury within 72-Hours Post-ICU Admission Using Evidence from the MIMIC-III Database. Discov Med. 2023;35(177):623-631.	37553314	doi:10.24976/Discov.Med.202335177.61
240	Okawa T, Mizuno T, Hanabusa S, et al. Prediction model of acute kidney injury induced by cisplatin in older adults using a machine learning algorithm. PLoS One. 2022;17(1):e0262021.	35041690	doi:10.1371/journal.pone.0262021
241	Shawwa K, Ghosh E, Lanius S, Schwager E, Eshelman L, Kashani KB. Predicting acute kidney injury in critically ill patients using comorbid conditions utilizing machine learning. Clin Kidney J. 2021;14(5):1428-1435.	33959271	doi:10.1093/ckj/sfaa145
245	Chang HH, Chiang JH, Wang CS, et al. Predicting Mortality Using Machine Learning Algorithms in Patients Who Require Renal Replacement Therapy in the Critical Care Unit. J Clin Med. 2022;11(18).	36142936	doi:10.3390/jcm11185289
246	Cai D, Xiao T, Zou A, et al. Predicting acute kidney injury risk in acute myocardial infarction patients: An artificial intelligence model using medical information mart for intensive care databases. Front Cardiovasc Med. 2022;9:964894.	36158815	doi:10.3389/fcvm.2022.964894
250	Zamirpour S, Hubbard AE, Feng J, Butte AJ, Pirracchio R, Bishara A. Development of a Machine Learning Model of Postoperative Acute Kidney Injury Using Non-Invasive Time-Sensitive Intraoperative Predictors. Bioengineering (Basel). 2023;10(8).	37627817	doi:10.3390/bioengineering10080932
253	Sun L, Zhu W, Chen X, et al. Machine Learning to Predict Contrast-Induced Acute Kidney Injury in Patients With Acute Myocardial Infarction. Front Med (Lausanne). 2020;7:592007.	33282893	doi:10.3389/fmed.2020.592007
256	Lazebnik T, Bahouth Z, Bunimovich-Mendrazitsky S, Halachmi S. Predicting acute kidney injury following open partial nephrectomy treatment using SAT-pruned explainable machine learning model. BMC Med Inform Decis Mak. 2022;22(1):133.	35578278	doi:10.1186/s12911-022-01877-8
259	Liu J, Wu J, Liu S, Li M, Hu K, Li K. Predicting mortality of patients with acute kidney injury in the ICU using XGBoost model. PLoS One. 2021;16(2):e0246306.	33539390	doi:10.1371/journal.pone.0246306

Record Number	Full reference in JMIR/AMA style	PMID	DOI or URL
262	Zhang Y, Zhang X, Xi X, Dong W, Zhao Z, Chen S. Development and validation of AKI prediction model in postoperative critically ill patients: a multicenter cohort study. Am J Transl Res. 2022;14(8):5883-5895.	36105045	Not available
263	Shi H, Shen Y, Li L. Early prediction of acute kidney injury in patients with gastrointestinal bleeding admitted to the intensive care unit based on extreme gradient boosting. Front Med (Lausanne). 2023;10:1221602.	37720504	doi:10.3389/fmed.2023.1221602
264	Wei J, Cai D, Xiao T, et al. Artificial intelligence algorithms permits rapid acute kidney injury risk classification of patients with acute myocardial infarction. Heliyon. 2024;10(16):e36051.	39224361	doi:10.1016/j.heliyon.2024.e36051
272	Wu M, Jiang X, Du K, Xu Y, Zhang W. Ensemble machine learning algorithm for predicting acute kidney injury in patients admitted to the neurointensive care unit following brain surgery. Sci Rep. 2023;13(1):6705.	37185782	doi:10.1038/s41598-023-33930-5
283	Hou S, Zhang L, Ji H, et al. Development and evaluation of the model for acute kidney injury in patients with cardiac arrest after successful resuscitation. BMC Cardiovasc Disord. 2024;24(1):440.	39180000	doi:10.1186/s12872-024-04110-8
284	Liu HX, Wang X, Xu MM, et al. A new prediction model for acute kidney injury following liver transplantation using grafts from donors after cardiac death. Front Med (Lausanne). 2024;11:1389695.	38873211	doi:10.3389/fmed.2024.1389695
285	He ZL, Zhou JB, Liu ZK, et al. Application of machine learning models for predicting acute kidney injury following donation after cardiac death liver transplantation. Hepatobiliary Pancreat Dis Int. 2021;20(3):222-231.	33726966	doi:10.1016/j.hbpd.2021.02.001
286	Xinsai L, Zhengye W, Xuan H, et al. Prediction model of acute kidney injury after different types of acute aortic dissection based on machine learning. Front Cardiovasc Med. 2022;9:984772.	36211563	doi:10.3389/fcvm.2022.984772
289	Zhang Y, Yang D, Liu Z, et al. An explainable supervised machine learning predictor of acute kidney injury after adult deceased donor liver transplantation. J Transl Med. 2021;19(1):321.	34321016	doi:10.1186/s12967-021-02990-4
290	Li L, Guan J, Peng X, et al. Machine learning for the prediction of 1-year mortality in patients with sepsis-associated acute kidney injury. BMC Med Inform Decis Mak. 2024;24(1):208.	39054463	doi:10.1186/s12911-024-02583-3
295	Yang J, Peng H, Luo Y, Zhu T, Xie L. Explainable ensemble machine learning model for prediction of 28-day mortality risk in patients with sepsis-associated acute kidney injury. Front Med (Lausanne). 2023;10:1165129.	37275353	doi:10.3389/fmed.2023.1165129
300	Shao J, Liu F, Ji S, et al. Development, External Validation, and Visualization of Machine Learning Models for Predicting Occurrence of Acute Kidney Injury after Cardiac Surgery. Rev Cardiovasc Med. 2023;24(8):229.	39076716	doi:10.31083/j.rcm2408229
302	Lee HC, Yoon SB, Yang SM, et al. Prediction of Acute Kidney Injury after Liver Transplantation: Machine Learning Approaches vs. Logistic Regression Model. J Clin Med. 2018;7(11).	30413107	doi:10.3390/jcm7110428
303	Han C, Soh S, Kim HI, Song JW, Yoon D. Machine Learning with Clinical and Intraoperative Biosignal Data for Predicting Cardiac Surgery-Associated Acute Kidney Injury. Stud Health Technol Inform. 2024;316:286-290.	39176729	doi:10.3233/shti240400
307	Chi S, Zhou T, Zhu W, Li X, Li J. A Knowledge-based and Data-driven Approach for Predicting Acute Kidney Injury in Patients with Heart Failure(). Annu Int Conf IEEE Eng Med Biol Soc. 2023;2023:46026.	38083608	doi:10.1109/embc40787.2023.10340347
308	Yang D, Zhao L, Kang J, et al. Development and validation of a predictive model for acute kidney injury in patients with moderately severe and severe acute pancreatitis. Clin Exp Nephrol. 2022;26(8):770-787.	35430680	doi:10.1007/s10157-022-02219-8
309	Dai A, Zhou Z, Jiang F, et al. Incorporating intraoperative blood pressure time-series variables to assist in prediction of acute kidney injury after type a acute aortic dissection repair: an interpretable machine learning model. Ann Med. 2023;55(2):2266458.	37813109	doi:10.1080/07853890.2023.2266458
311	Luo XQ, Yan P, Duan SB, et al. Development and Validation of Machine Learning Models for Real-Time Mortality Prediction in Critically Ill Patients With Sepsis-Associated Acute Kidney Injury. Front Med (Lausanne). 2022;9:853102.	35783603	doi:10.3389/fmed.2022.853102
321	Chen YY, Liu CF, Shen YT, et al. Development of real-time individualized risk prediction models for contrast associated acute kidney injury and 30-day dialysis after contrast enhanced computed tomography. Eur J Radiol. 2023;167:111034.	37591134	doi:10.1016/j.ejrad.2023.111034
332	Pan L, Deng Y, Dai S, et al. Development and internal validation of a prediction model for acute kidney injury following cardiac valve replacement surgery. Int J Cardiol. 2023;370:345-350.	36306946	doi:10.1016/j.ijcard.2022.10.147
336	Tian Y, Diao X, Wang Y, et al. Prediction Scores for Any-Stage and Stage-3 Acute Kidney Injury After Adult Cardiac Surgery in a Chinese Population. J Cardiothorac Vasc Anesth. 2021;35(10):3001-3009.	33810934	doi:10.1053/j.jvca.2021.02.047
341	Xu L, Jiang S, Li C, et al. Acute kidney disease in hospitalized pediatric patients: risk prediction based on an artificial intelligence approach. Ren Fail. 2024;46(2):2438858.	39668464	doi:10.1080/0886022x.2024.2438858
344	Lee MY, Heo KN, Lee S, Ah YM, Shin J, Lee JY. Development and validation of a medication-based risk prediction model for acute kidney injury in older outpatients. Arch Gerontol Geriatr. 2024;120:105332.	38382232	doi:10.1016/j.archger.2024.105332
347	Lee HC, Yoon HK, Nam K, et al. Derivation and Validation of Machine Learning Approaches to Predict Acute Kidney Injury after Cardiac Surgery. J Clin Med. 2018;7(10).	30282956	doi:10.3390/jcm7100322
351	Parreco J, Soe-Lin H, Parks JJ, et al. Comparing Machine Learning Algorithms for Predicting Acute Kidney Injury. Am Surg. 2019;85(7):725-729.	31405416	Not available
352	Zhang W, Niu H, Yuan F, et al. Development of machine learning prediction model for AKI after craniotomy and evacuation of hematoma in craniocerebral trauma. Medicine (Baltimore). 2024;103(44):e39735.	39496042	doi:10.1097/md.0000000000039735

Record Number	Full reference in JMIR/AMA style	PMID	DOI or URL
362	Hsu CN, Liu CL, Tain YL, Kuo CY, Lin YC. Machine Learning Model for Risk Prediction of Community-Acquired Acute Kidney Injury Hospitalization From Electronic Health Records: Development and Validation Study. J Med Internet Res. 2020;22(8):e16903.	32749223	doi:10.2196/16903
363	Ma M, Chen C, Chen D, et al. A Machine Learning-Based Prediction Model for Acute Kidney Injury in Patients With Community-Acquired Pneumonia: Multicenter Validation Study. J Med Internet Res. 2024;26:e51255.	39699941	doi:10.2196/51255
366	Peng X, Zhu T, Chen Q, et al. A simple machine learning model for the prediction of acute kidney injury following noncardiac surgery in geriatric patients: a prospective cohort study. BMC Geriatr. 2024;24(1):549.	38918723	doi:10.1186/s12877-024-05148-1
371	Yu X, Wu R, Ji Y, Huang M, Feng Z. Identifying Patients at Risk of Acute Kidney Injury among Patients Receiving Immune Checkpoint Inhibitors: A Machine Learning Approach. Diagnostics (Basel). 2022;12(12).	36553164	doi:10.3390/diagnostics12123157
379	Lu JY, Zhu J, Zhu J, Duong TQ. Long-short-term memory machine learning of longitudinal clinical data accurately predicts acute kidney injury onset in COVID-19: a two-center study. Int J Infect Dis. 2022;122:802-810.	35872094	doi:10.1016/j.ijid.2022.07.034
384	Lei L, Wang Y, Xue Q, Tong J, Zhou CM, Yang JJ. A comparative study of machine learning algorithms for predicting acute kidney injury after liver cancer resection. PeerJ. 2020;8:e8583.	32140301	doi:10.7717/peerj.8583
394	Scanlon LA, O'Hara C, Garbett A, Barker-Hewitt M, Barriuso J. Developing an Agnostic Risk Prediction Model for Early AKI Detection in Cancer Patients. Cancers (Basel). 2021;13(16).	34439336	doi:10.3390/cancers13164182
395	Li L, Wan X, Zhang Y, Zhao M, Ren J, Zhang Y. Analysis of factors associated with postoperative acute kidney injury in patients with colorectal cancer and the development of a risk prediction model: a retrospective study. Updates Surg. 2023;75(5):1171-1178.	36892811	doi:10.1007/s13304-023-01481-z
398	Tian J, Cui R, Song H, Zhao Y, Zhou T. Prediction of acute kidney injury in patients with liver cirrhosis using machine learning models: evidence from the MIMIC-III and MIMIC-IV. Int Urol Nephrol. 2024;56(1):237-247.	37256426	doi:10.1007/s11255-023-03646-6
405	Liu HH, Wang YT, Yang MH, Lin WK, Oyang YJ. Exploiting Machine Learning Technologies to Study the Compound Effects of Serum Creatinine and Electrolytes on the Risk of Acute Kidney Injury in Intensive Care Units. Diagnostics (Basel). 2023;13(15).	37568914	doi:10.3390/diagnostics13152551
407	Lu X, Chen Y, Zhang G, Zeng X, Lai L, Qu C. Application of interpretable machine learning algorithms to predict acute kidney injury in patients with cerebral infarction in ICU. J Stroke Cerebrovasc Dis. 2024;33(7):107729.	38657830	doi:10.1016/j.jstrokecerebrovasdis.2024.1077 29
408	Chi C, Song X, Ma Y, Wang C, Zhu J. Establishment and Diagnostic Value of an Early Prediction Model for Acute Pancreatitis Complicated With Acute Kidney Injury. Pancreas. 2024;53(7):e547-e552.	38986076	doi:10.1097/mpa.0000000000002325
409	Yang M, Liu S, Hao T, et al. Development and validation of a deep interpretable network for continuous acute kidney injury prediction in critically ill patients. Artif Intell Med. 2024;149:102785.	38462285	doi:10.1016/j.artmed.2024.102785
411	Yun D, Cho S, Kim YC, et al. Use of Deep Learning to Predict Acute Kidney Injury After Intravenous Contrast Media Administration: Prediction Model Development Study. JMIR Med Inform. 2021;9(10):e27177.	34596574	doi:10.2196/27177
415	Zhang Y, Wang Y, Xu J, et al. Comparison of Prediction Models for Acute Kidney Injury Among Patients with Hepatobiliary Malignancies Based on XGBoost and LASSO-Logistic Algorithms. Int J Gen Med. 2021;14:1325-1335.	33889012	doi:10.2147/ijgm.S302795
417	Li J, Wu J, Lei L, et al. Combining cardiac and renal biomarkers to establish a clinical early prediction model for cardiac surgery-associated acute kidney injury: a prospective observational study. J Thorac Dis. 2024;16(12):8399-8416.	39831226	doi:10.21037/jtd-24-1185
421	Kim K, Yang H, Yi J, et al. Real-Time Clinical Decision Support Based on Recurrent Neural Networks for In-Hospital Acute Kidney Injury: External Validation and Model Interpretation. J Med Internet Res. 2021;23(4):e24120.	33861200	doi:10.2196/24120
424	Gu M, Liu Y, Sun H, et al. Using machine learning to predict the risk of short-term and long-term death in acute kidney injury patients after commencing CRRT. BMC Nephrol. 2024;25(1):245.	39080581	doi:10.1186/s12882-024-03676-x
427	Liu X, Ai S, Yu R, Zhang C, Miao Q. Development and evaluation of a machine learning model for post-surgical acute kidney injury in active infective endocarditis. Front Cardiovasc Med. 2024;11:1425275.	39713217	doi:10.3389/fcvm.2024.1425275
430	Hou X, Zhang K, Liu T, et al. Prediction of Acute Kidney Injury Following Isolated Coronary Artery Bypass Grafting in Heart Failure Patients with Preserved Ejection Fraction Using Machine Leaning with a Novel Nomogram. Rev Cardiovasc Med. 2024;25(2):43.	39077338	doi:10.31083/j.rcm2502043
435	Du Y, Wang XZ, Wu WD, et al. Predicting the Risk of Acute Kidney Injury in Patients After Percutaneous Coronary Intervention (PCI) or Cardiopulmonary Bypass (CPB) Surgery: Development and Assessment of a Nomogram Prediction Model. Med Sci Monit. 2021;27:e929791.	33895770	doi:10.12659/msm.929791
443	Sanchez-Pinto LN, Khemani RG. Development of a Prediction Model of Early Acute Kidney Injury in Critically Ill Children Using Electronic Health Record Data. Pediatr Crit Care Med. 2016;17(6):508-15.	27124567	doi:10.1097/pcc.0000000000000750
446	Lee SW, Jang J, Seo WY, Lee D, Kim SH. Internal and External Validation of Machine Learning Models for Predicting Acute Kidney Injury Following Non-Cardiac Surgery Using Open Datasets. J Pers Med. 2024;14(6).	38929808	doi:10.3390/jpm14060587
458	Tran P, Knecht S, Tamine L, et al. Risk prediction of kalaemia disturbance and acute kidney injury after total knee arthroplasty: use of a machine learning algorithm. Orthop Traumatol Surg Res. 2024:103958.	39047862	doi:10.1016/j.otsr.2024.103958
465	Zhang L, Li M, Wang C, Zhang C, Wu H. Prediction of acute kidney injury in intensive care unit patients based on interpretable machine learning. Digit Health. 2025;11:20552076241311173.	39777058	doi:10.1177/20552076241311173

Record Number	Full reference in JMIR/AMA style	PMID	DOI or URL
470	Jeon N, Staley B, Henriksen C, Lipori GP, Winterstein AG. Development and validation of an automated algorithm for identifying patients at higher risk for drug-induced acute kidney injury. Am J Health Syst Pharm. 2019;76(10):654-666.	31361856	doi:10.1093/ajhp/zxz043
473	Wang Q, Tang Y, Zhou J, Qin W. A prospective study of acute kidney injury in the intensive care unit: development and validation of a risk prediction model. J Transl Med. 2019;17(1):359.	31690326	doi:10.1186/s12967-019-2118-6
480	Zhang X, Xiong Y, Liu H, Liu Q, Chen S. Prediction of Acute Kidney Injury for Critically Ill Cardiogenic Shock Patients with Machine Learning Algorithms. Int J Gen Med. 2025;18:33-42.	39801924	doi:10.2147/ijgm.S489362
485	Zhang Y, Xu D, Gao J, et al. Development and validation of a real-time prediction model for acute kidney injury in hospitalized patients. Nat Commun. 2025;16(1):68.	39747882	doi:10.1038/s41467-024-55629-5
490	Ye M, Liu C, Yang D, Gao H. Development and validation of a risk prediction model for acute kidney injury in coronary artery disease. BMC Cardiovasc Disord. 2025;25(1):12.	39794721	doi:10.1186/s12872-024-04466-x
503	Lin C, Lin S, Zheng M, et al. Development and validation of an early acute kidney injury risk prediction model for patients with sepsis in emergency departments. Ren Fail. 2024;46(2):2419523.	39477816	doi:10.1080/0886022x.2024.2419523
514	Ambe K, Aoki Y, Murashima M, et al. Prediction of Cisplatin-Induced Acute Kidney Injury Using an Interpretable Machine Learning Model and Electronic Medical Record Information. Clin Transl Sci. 2025;18(1):e70115.	39760531	doi:10.1111/cts.70115
515	Kate RJ, Perez RM, Mazumdar D, Pasupathy KS, Nilakantan V. Prediction and detection models for acute kidney injury in hospitalized older adults. BMC Med Inform Decis Mak. 2016;16:39.	27025458	doi:10.1186/s12911-016-0277-4
517	Zha D, Yang X, Zhang H, et al. Development and validation of prediction model using nursing notes on sentiment scores for prognosis of patients with severe acute kidney injury receiving continuous renal replacement therapy based on computational intelligence algorithms. Ann Transl Med. 2022;10(20):1110.	36388821	doi:10.21037/atm-22-4403
518	Lin K, Hu Y, Kong G. Predicting in-hospital mortality of patients with acute kidney injury in the ICU using random forest model. Int J Med Inform. 2019;125:55-61.	30914181	doi:10.1016/j.ijmedinf.2019.02.002
519	Zhou Y, Feng J, Mei S, et al. MACHINE LEARNING MODELS FOR PREDICTING ACUTE KIDNEY INJURY IN PATIENTS WITH SEPSIS-ASSOCIATED ACUTE RESPIRATORY DISTRESS SYNDROME. Shock. 2023;59(3):352-359.	36625493	doi:10.1097/shk.0000000000002065
524	Jing H, Liao M, Tang S, et al. Predicting the risk of acute kidney injury after cardiopulmonary bypass: development and assessment of a new predictive nomogram. BMC Anesthesiol. 2022;22(1):379.	36476178	doi:10.1186/s12871-022-01925-w
525	Li Y, Chan TM, Feng J, et al. A pattern-discovery-based outcome predictive tool integrated with clinical data repository: design and a case study on contrast related acute kidney injury. BMC Med Inform Decis Mak. 2022;22(1):103.	35428291	doi:10.1186/s12911-022-01841-6
526	Akimoto H, Hayakawa T, Nagashima T, Minagawa K, Takahashi Y, Asai S. Detection of potential drug-drug interactions for risk of acute kidney injury: a population-based case-control study using interpretable machine-learning models. Front Pharmacol. 2023;14:1176096.	37288110	doi:10.3389/fphar.2023.1176096
533	Griffin BR, Mudireddy A, Horne BD, et al. Predicting Nephrotoxic Acute Kidney Injury in Hospitalized Adults: A Machine Learning Algorithm. Kidney Med. 2024;6(12):100918.	39634332	doi:10.1016/j.xkme.2024.100918
545	He J, Hu Y, Zhang X, Wu L, Waitman LR, Liu M. Multi-perspective predictive modeling for acute kidney injury in general hospital populations using electronic medical records. JAMIA Open. 2019;2(1):115-122.	30976758	doi:10.1093/jamiaopen/ooy043
548	Li Y, Hou XJ, Liu TS, et al. Risk factors for acute kidney injury following coronary artery bypass graft surgery in a Chinese population and development of a prediction model. J Geriatr Cardiol. 2021;18(9):711-719.	34659377	doi:10.11909/j.issn.1671-5411.2021.09.011
556	Lu J, Qi Z, Liu J, et al. Nomogram Prediction Model of Serum Chloride and Sodium Ions on the Risk of Acute Kidney Injury in Critically Ill Patients. Infect Drug Resist. 2022;15:4785-4798.	36045875	doi:10.2147/idr.S376168
557	Li Y, Xu J, Wang Y, et al. A novel machine learning algorithm, Bayesian networks model, to predict the high-risk patients with cardiac surgery-associated acute kidney injury. Clin Cardiol. 2020;43(7):752-761.	32400109	doi:10.1002/clc.23377
562	Zheng L, Lin Y, Fang K, Wu J, Zheng M. Derivation and validation of a risk score to predict acute kidney injury in critically ill cirrhotic patients. Hepatol Res. 2023;53(8):701-712.	37041695	doi:10.1111/hepr.13907
563	Wu L, Li Y, Zhang X, et al. Prediction differences and implications of acute kidney injury with and without urine output criteria in adult critically ill patients. Nephrol Dial Transplant. 2023;38(10):2368-2378.	37019835	doi:10.1093/ndt/gfad065
568	Martin-Cleary C, Molinero-Casares LM, Ortiz A, Arce-Obieta JM. Development and internal validation of a prediction model for hospital-acquired acute kidney injury. Clin Kidney J. 2021;14(1):309-316.	33564433	doi:10.1093/ckj/sfz139
577	Tu H, Su J, Gong K, et al. A dynamic model to predict early occurrence of acute kidney injury in ICU hospitalized cirrhotic patients: a MIMIC database analysis. BMC Gastroenterol. 2024;24(1):290.	39192202	doi:10.1186/s12876-024-03369-7
578	Ma B, Jin G, Mao F, et al. Development of a nomogram to predict the incidence of acute kidney injury among ischemic stroke individuals during ICU hospitalization. Heliyon. 2024;10(3):e25566.	38352771	doi:10.1016/j.heliyon.2024.e25566
590	Wang M, Yan P, Zhang NY, et al. Prediction of Mortality Risk After Ischemic Acute Kidney Injury With a Novel Prognostic Model: A Multivariable Prediction Model Development and Validation Study. Front Med (Lausanne). 2022;9:892473.	36045922	doi:10.3389/fmed.2022.892473

Record Number	Full reference in JMIR/AMA style	PMID	DOI or URL
598	Qian Q, Wu J, Wang J, Sun H, Yang L. Prediction Models for AKI in ICU: A Comparative Study. Int J Gen Med. 2021;14:623-632.	33664585	doi:10.2147/ijgm.S289671
601	Huang H, Liu Y, Wu M, Gao Y, Yu X. Development and validation of a risk stratification model for predicting the mortality of acute kidney injury in critical care patients. Ann Transl Med. 2021;9(4):323.	33708950	doi:10.21037/atm-20-5723
603	Behnoush AH, Shariatnia MM, Khalaji A, et al. Predictive modeling for acute kidney injury after percutaneous coronary intervention in patients with acute coronary syndrome: a machine learning approach. Eur J Med Res. 2024;29(1):76.	38268045	doi:10.1186/s40001-024-01675-0
608	Jiang D, Shen M, Yuan X, et al. Serum heart-type fatty acid-binding protein as a predictor for the development of sepsis-associated acute kidney injury. Expert Rev Mol Diagn. 2019;19(8):757-765.	31288580	doi:10.1080/14737159.2019.1642750
618	Adhikari L, Ozrazgat-Baslanti T, Ruppert M, et al. Improved predictive models for acute kidney injury with IDEA: Intraoperative Data Embedded Analytics. PLoS One. 2019;14(4):e0214904.	30947282	doi:10.1371/journal.pone.0214904
619	Sakuragi M, Uchino E, Sato N, et al. Interpretable machine learning-based individual analysis of acute kidney injury in immune checkpoint inhibitor therapy. PLoS One. 2024;19(3):e0298673.	38502665	doi:10.1371/journal.pone.0298673
623	Li Y, Chen X, Shen Z, et al. Prediction models for acute kidney injury in patients with gastrointestinal cancers: a real-world study based on Bayesian networks. Ren Fail. 2020;42(1):869-876.	32838613	doi:10.1080/0886022x.2020.1810068
626	Gao W, Wang J, Zhou L, et al. Prediction of acute kidney injury in ICU with gradient boosting decision tree algorithms. Comput Biol Med. 2022;140:105097.	34864304	doi:10.1016/j.compbiomed.2021.105097
656	Liu K, Yuan B, Zhang X, et al. Characterizing the temporal changes in association between modifiable risk factors and acute kidney injury with multi-view analysis. Int J Med Inform. 2022;163:104785.	35504130	doi:10.1016/j.ijmedinf.2022.104785
660	Wu S, Zhou Q, Cai Y, Duan X. Development and validation of a prediction model for the early occurrence of acute kidney injury in patients with acute pancreatitis. Ren Fail. 2023;45(1):2194436.	36999227	doi:10.1080/0886022x.2023.2194436
661	Shi S, Xiong C, Bie D, Li Y, Wang J. Development and Validation of a Nomogram for Predicting Acute Kidney Injury in Pediatric Patients Undergoing Cardiac Surgery. Pediatr Cardiol. 2024.	38217691	doi:10.1007/s00246-023-03392-7
665	Ponce D, de Andrade LGM, Claure-Del Granado R, Ferreiro-Fuentes A, Lombardi R. Development of a prediction score for in-hospital mortality in COVID-19 patients with acute kidney injury: a machine learning approach. Sci Rep. 2021;11(1):24439.	34952908	doi:10.1038/s41598-021-03894-5
682	Sun T, Yue X, Zhang G, et al. AKIML(pred): An interpretable machine learning model for predicting acute kidney injury within seven days in critically ill patients based on a prospective cohort study. Clin Chim Acta. 2024;559:119705.	38702035	doi:10.1016/j.cca.2024.119705
687	Nikkinen O, Kolehmainen T, Aaltonen T, Jämsä E, Alahuhta S, Vakkala M. Developing a supervised machine learning model for predicting perioperative acute kidney injury in arthroplasty patients. Comput Biol Med. 2022;144:105351.	35286890	doi:10.1016/j.compbiomed.2022.105351
688	Huang C, Murugiah K, Mahajan S, et al. Enhancing the prediction of acute kidney injury risk after percutaneous coronary intervention using machine learning techniques: A retrospective cohort study. PLoS Med. 2018;15(11):e1002703.	30481186	doi:10.1371/journal.pmed.1002703
694	Wang J, Dong Y, Zhao B, Liu K. Preoperative NT-proBNP and LVEF for the prediction of acute kidney injury after noncardiac surgery: a single-centre retrospective study. BMC Anesthesiol. 2022;22(1):196.	35751021	doi:10.1186/s12871-022-01727-0
706	Heo KY, Rajan PV, Khawaja S, Barber LA, Yoon ST. Machine learning approach to predict acute kidney injury among patients undergoing multi-level spinal posterior instrumented fusion. J Spine Surg. 2024;10(3):362-371.	39399076	doi:10.21037/jss-24-15
721	Cao X, Liang Y, Feng H, Chen L, Liu S. Construction and evaluation of a risk prediction model for pulmonary infection-associated acute kidney injury in intensive care units. Clin Transl Sci. 2023;16(10):1923-1934.	37488744	doi:10.1111/cts.13599
724	Zheng L, Yang J, Zhao L, et al. Development and validation of the PHM-CPA model to predict in-hospital mortality for cirrhotic patients with acute kidney injury. Dig Liver Dis. 2024.	39379230	doi:10.1016/j.dld.2024.09.012
728	Shen J, Xue B, Kannampallil T, Lu C, Abraham J. A novel generative multi-task representation learning approach for predicting postoperative complications in cardiac surgery patients. J Am Med Inform Assoc. 2024.	39731515	doi:10.1093/jamia/ocae316
736	Pan P, Liu Y, Xie F, et al. Significance of platelets in the early warning of new-onset AKI in the ICU by using supervise learning: a retrospective analysis. Ren Fail. 2023;45(1):2194433.	37013397	doi:10.1080/0886022x.2023.2194433
741	Wang L, McGregor TL, Jones DP, et al. Electronic health record-based predictive models for acute kidney injury screening in pediatric inpatients. Pediatr Res. 2017;82(3):465-473.	28486440	doi:10.1038/pr.2017.116
750	Peng W, Yang B, Qiao H, Liu Y, Lin Y. Prediction of acute kidney injury following coronary artery bypass graft surgery in elderly Chinese population. J Cardiothorac Surg. 2023;18(1):287.	37817194	doi:10.1186/s13019-023-02372-5
754	Xin Q, Xie T, Chen R, et al. Construction and validation of an early warning model for predicting the acute kidney injury in elderly patients with sepsis. Aging Clin Exp Res. 2022;34(12):2993-3004.	36053443	doi:10.1007/s40520-022-02236-3
762	Goriki Y, Tanaka A, Nishihira K, Kuriyama N, Shibata Y, Node K. A Novel Prediction Model of Acute Kidney Injury Based on Combined Blood Variables in STEMI. JACC Asia. 2021;1(3):372-381.	36341223	doi:10.1016/j.jacasi.2021.07.013
794	Choi H, Lee JY, Sul Y, et al. Comparing machine learning and logistic regression for acute kidney injury prediction in trauma patients: A retrospective observational study at a single tertiary medical center. Medicine (Baltimore). 2023;102(33):e34847.	37603521	doi:10.1097/md.0000000000034847

Record Number	Full reference in JMIR/AMA style	PMID	DOI or URL
835	Chen D, Zhao J, Ma M, Jiang L, Tan Y, Wan X. Dynamic nomogram for predicting acute kidney injury in patients with community-acquired pneumonia. BMJ Open Respir Res. 2023;10(1).	37739457	doi:10.1136/bmjresp-2022-001495
852	Wang R, Zhang J, He M, Xu J. Classification and Regression Tree Predictive Model for Acute Kidney Injury in Traumatic Brain Injury Patients. Ther Clin Risk Manag. 2024;20:139-149.	38410117	doi:10.2147/tcrm.S435281
853	Kuno T, Numasawa Y, Mikami T, et al. Association of decreasing hemoglobin levels with the incidence of acute kidney injury after percutaneous coronary intervention: a prospective multi-center study. Heart Vessels. 2021;36(3):330-336.	33034713	doi:10.1007/s00380-020-01706-w
873	Li X, Li Y, Fan CJ, et al. A nomogram for predicting 28-day mortality in elderly patients with acute kidney injury receiving continuous renal replacement therapy: a secondary analysis based on a retrospective cohort study. BMC Nephrol. 2024;25(1):195.	38862887	doi:10.1186/s12882-024-03628-5
874	Jiang Z, An X, Li Y, Xu C, Meng H, Qu Y. Construction and validation of a risk assessment model for acute kidney injury in patients with acute pancreatitis in the intensive care unit. BMC Nephrol. 2023;24(1):315.	37884898	doi:10.1186/s12882-023-03369-x
894	Hofer IS, Kupina M, Laddaran L, Halperin E. Integration of feature vectors from raw laboratory, medication and procedure names improves the precision and recall of models to predict postoperative mortality and acute kidney injury. Sci Rep. 2022;12(1):10254.	35715454	doi:10.1038/s41598-022-13879-7
898	Zhang H, Wang Z, Tang Y, et al. Prediction of acute kidney injury after cardiac surgery: model development using a Chinese electronic health record dataset. J Transl Med. 2022;20(1):166.	35397573	doi:10.1186/s12967-022-03351-5
905	Yue S, Li S, Huang X, et al. Construction and Validation of a Risk Prediction Model for Acute Kidney Injury in Patients Suffering from Septic Shock. Dis Markers. 2022;2022:9367873.	35035614	doi:10.1155/2022/9367873
920	Yue X, Li Z, Wang L, et al. [Development and validation of a nomogram for predicting 3-month mortality risk in patients with sepsis-associated acute kidney injury]. Zhonghua Wei Zhong Bing Ji Jiu Yi Xue. 2024;36(5):465-470.	38845491	doi:10.3760/cma.j.cn121430-20231218-01091
924	Krzanowska K, Batko K, Niezabitowska K, et al. Predicting acute kidney injury onset with a random forest algorithm using electronic medical records of COVID-19 patients: the CRACoV-AKI model. Pol Arch Intern Med. 2024;134(5).	38483266	doi:10.20452/pamw.16697
932	Yan P, Duan SB, Luo XQ, Zhang NY, Deng YH. Development and validation of a deep neural network-based model to predict acute kidney injury following intravenous administration of iodinated contrast media in hospitalized patients with chronic kidney disease: a multicohort analysis. Nephrol Dial Transplant. 2023;38(2):352-361.	35218197	doi:10.1093/ndt/gfac049
947	Wang C, Sun H, Li X, et al. Development and validation of a nomogram for the early prediction of acute kidney injury in hospitalized COVID-19 patients. Front Public Health. 2022;10:1047073.	36505004	doi:10.3389/fpubh.2022.1047073
949	Xin Q, Xie T, Chen R, et al. A Predictive Model Based on Inflammatory and Coagulation Indicators for Sepsis-Induced Acute Kidney Injury. J Inflamm Res. 2022;15:4561-4571.	35979508	doi:10.2147/jir.S372246
991	Wang X, Fu X. Predicting AKI in patients with AMI: Development and assessment of a new predictive nomogram. Medicine (Baltimore). 2023;102(24):e33991.	37327276	doi:10.1097/md.0000000000033991
992	Peng JC, Wu T, Wu X, et al. Development of mortality prediction model in the elderly hospitalized AKI patients. Sci Rep. 2021;11(1):15157.	34312443	doi:10.1038/s41598-021-94271-9
1027	Hofer IS, Lee C, Gabel E, Baldi P, Cannesson M. Development and validation of a deep neural network model to predict postoperative mortality, acute kidney injury, and reintubation using a single feature set. NPJ Digit Med. 2020;3:58.	32352036	doi:10.1038/s41746-020-0248-0
1069	Liu K, Zhang X, Chen W, et al. Development and Validation of a Personalized Model With Transfer Learning for Acute Kidney Injury Risk Estimation Using Electronic Health Records. JAMA Netw Open. 2022;5(7):e2219776.	35796212	doi:10.1001/jamanetworkopen.2022.19776
1108	Li C, Zhao K, Ren Q, et al. Development and validation of a model for predicting in-hospital mortality in patients with sepsis-associated kidney injury receiving renal replacement therapy: a retrospective cohort study based on the MIMIC-IV database. Front Cell Infect Microbiol. 2024;14:1488505.	39559702	doi:10.3389/fcimb.2024.1488505
2814	Jiang Y, Zhang J, Ainiwaer A, et al. Development and validation of a predictive model for acute kidney injury in patients with ureterolithiasis. RENAL FAILURE. 2024;46(2):2394634.	Not available	doi:10.1080/0886022X.2024.2394634
2905	Li S, Li Z, Li Y. Identification of risk factors and establishment of prediction models for mortality risk in patients with acute kidney injury: A retrospective cohort study. PLOS ONE. 2024;19(10):e0312482.	Not available	doi:10.1371/journal.pone.0312482
2956	Sun T, Yue X, Chen X, et al. A novel real-time model for predicting acute kidney injury in critically ill patients within 12 hours. NEPHROLOGY DIALYSIS TRANSPLANTATION. 2024.	Not available	doi:10.1093/ndt/gfae168
3072	Li X, Li X, Zhao W, Wang D. Development and validation of a nomogram for predicting in-hospital death in cirrhotic patients with acute kidney injury. BMC NEPHROLOGY. 2024;25(1):175.	Not available	doi:10.1186/s12882-024-03609-8
3198	Hu J, Xu J, Li M, et al. Identification and validation of an explainable prediction model of acute kidney injury with prognostic implications in ill children: a multicenter cohort. ECLINICALMEDICINE. 2024;68:102409.	Not available	doi:10.1016/j.eclinm.2023.102409
3222	Cai EZ. Enriching an Acute Kidney Injury Prediction Model Among Percutaneous Coronary Intervention Patients: Leveraging Electronic Health Record Data. Journal not available. 2024.	Not available	Not available
3415	Tian Y, Zhang Y, He J, et al. Predictive model of acute kidney injury after spontaneous intracerebral hemorrhage: A multicenter retrospective study. EUROPEAN STROKE JOURNAL. 2023;8(3):747-755.	Not available	doi:10.1177/23969873231184667
3417	Wu C, Zhang Y, Nie S, et al. Predicting in-hospital outcomes of patients with acute kidney injury. NATURE COMMUNICATIONS. 2023;14(1):3739.	Not available	doi:10.1038/s41467-023-39474-6

Record Number	Full reference in JMIR/AMA style	PMID	DOI or URL
3533	L.-H. Feng, Lu Y, Ren S, Liang H, Wei L, Jiang J. Development and validation of a dynamic online nomogram for predicting acute kidney injury in cirrhotic patients upon ICU admission. FRONTIERS IN MEDICINE. 2023;10:1055137.	Not available	doi:10.3389/fmed.2023.1055137
3595	Zhao H, Miao R, Lin F, Zhao G. Risk Score for Prediction of Acute Kidney Injury in Patients with Acute ST-Segment Elevation Myocardial Infarction. Disease Markers. 2022;2022:7493690-Article No.: 7493690.	Not available	doi:10.1155/2022/7493690
3652	Hu Y, Wang X, Xiao S, et al. A Clinical Nomogram Based on the Triglyceride-Glucose Index to Predict Contrast-Induced Acute Kidney Injury after Percutaneous Intervention in Patients with Acute Coronary Syndrome with Diabetes Mellitus. CARDIOVASCULAR THERAPEUTICS. 2022;2022:5443880.	Not available	doi:10.1155/2022/5443880
3695	Zhang Y, Lan Y, Chen T, Chen Q, Guo Z, Jiang N. Prediction of Acute Kidney Injury for Acute Type A Aortic Dissection Patients Who Underwent Sun's Procedure by a Perioperative Nomogram. CARDIORENAL MEDICINE. 2022;12(3):117-130.	Not available	doi:10.1159/000524907
3942	Ma K, Li J, Shen G, et al. Development and Validation of a Risk Nomogram Model for Predicting Contrast-Induced Acute Kidney Injury in Patients with Non-ST-Elevation Acute Coronary Syndrome Undergoing Primary Percutaneous Coronary Intervention. CLINICAL INTERVENTIONS IN AGING. 2022;17:65-77.	Not available	doi:10.2147/CIA.S349159
4047	Nusca A, Mangiacapra F, Sticchi A, et al. Usefulness of Adding Pre-procedural Glycemia to the Mehran Score to Enhance Its Ability to Predict Contrast-induced Kidney Injury in Patients Undergoing Percutaneous Coronary Intervention Development and Validation of a Predictive Model. AMERICAN JOURNAL OF CARDIOLOGY. 2021;155:16-22.	Not available	doi:10.1016/j.amjcard.2021.06.014
4098	Chen Z, Chen M, Sun X, et al. Analysis of the Impact of Medical Features and Risk Prediction of Acute Kidney Injury for Critical Patients Using Temporal Electronic Health Record Data With Attention-Based Neural Network. FRONTIERS IN MEDICINE. 2021;8:658665.	Not available	doi:10.3389/fmed.2021.658665
4105	Wang J, Zhu B, Liu P, et al. Screening Key Indicators for Acute Kidney Injury Prediction Using Machine Learning. INTERNATIONAL JOURNAL OF COMPUTERS COMMUNICATIONS & CONTROL. 2021;16(3):4180.	Not available	doi:10.15837/ijccc.2021.3.4180
4116	Ma J, Deng Y, Lao H, et al. A nomogram incorporating functional and tubular damage biomarkers to predict the risk of acute kidney injury for septic patients. BMC NEPHROLOGY. 2021;22(1):176.	Not available	doi:10.1186/s12882-021-02388-w
4160	Fan T, Wang H, Wang J, Wang W, Guan H, Zhang C. Nomogram to predict the risk of acute kidney injury in patients with diabetic ketoacidosis: an analysis of the MIMIC-III database. BMC ENDOCRINE DISORDERS. 2021;21(1):37.	Not available	doi:10.1186/s12902-021-00696-8
4228	Wang X, Wang Y, Hui L, et al. Early prediction of acute kidney injury after liver transplantation by scoring system and decision tree. RENAL FAILURE. 2021;43(1):1137-1145.	Not available	doi:10.1080/0886022X.2021.1945462
4244	Du H, Pan Z, Ngiam KY, Wang F, Shum P, Feng M. Self-Correcting Recurrent Neural Network for Acute Kidney Injury Prediction in Critical Care. Health data science. 2021;2021:9808426-9808426.	38487505	doi:10.34133/2021/9808426
4277	Rank N, Pfahringer B, Kempfert J, et al. Deep-learning-based real-time prediction of acute kidney injury outperforms human predictive performance. NPJ DIGITAL MEDICINE. 2020;3(1):139.	Not available	doi:10.1038/s41746-020-00346-8
4352	Li Y, Chen X, Wang Y, Hu J, Shen Z, Ding X. Application of group LASSO regression based Bayesian networks in risk factors exploration and disease prediction for acute kidney injury in hospitalized patients with hematologic malignancies. BMC NEPHROLOGY. 2020;21(1):162.	Not available	doi:10.1186/s12882-020-01786-w
4827	Deng Y, Chi R, Chen S, et al. Evaluation of clinically available renal biomarkers in critically ill adults: a prospective multicenter observational study. CRITICAL CARE. 2017;21:46.	Not available	doi:10.1186/s13054-017-1626-0
5064	Ng SY, Sanagou M, Wolfe R, Cochrane A, Smith JA, Reid CM. Prediction of acute kidney injury within 30 days of cardiac surgery. JOURNAL OF THORACIC AND CARDIOVASCULAR SURGERY. 2014;147(6):1875-U225.	Not available	doi:10.1016/j.jtcvs.2013.06.049
5103	Kim WH, Lee SM, Choi JW, et al. Simplified Clinical Risk Score to Predict Acute Kidney Injury After Aortic Surgery. JOURNAL OF CARDIOTHORACIC AND VASCULAR ANESTHESIA. 2013;27(6):1158-1166.	Not available	doi:10.1053/j.jvca.2013.04.007

Supplementary Table S9. Full references of excluded full-text articles and reasons for exclusion (n = 435)

Note. Supplementary Table S9 order: Abstract, reviews, letter; Not English studies; Sample size <200; Full text not available; Non-ML models; Outcome indicators non-compliant; Non-predictive model studies; and No AUC. References follow a JMIR/AMA-style bibliographic structure using the available source fields: authors,title, journal, year, volume, issue, pages or article number, identifier, DOI, and URL. The uploaded source column labelled " PMID" contained PMCID-format values for most nonempty identifiers; these values are thereforereported as PMCID rather than converted to PMID. Missing fields are marked as "Not available". No external bibliographic lookup was performed.

1. Abstract, reviews, letter (n=58)

Record Number	Full reference in JMIR/AMA style	PMID	DOI or URL	Reason for exclusion
10	Song X, Liu X, Liu F, Wang C. Comparison of machine learning and logistic regression models in predicting acute kidney injury: A systematic review and meta-analysis. Int J Med Inform. 2021;151:104484.	33991886	doi:10.1016/j.ijmedinf.2021.104484	Abstract, reviews, letter
49	Huang CY, Grandas FG, Flechet M, Meyfroidt G. Clinical prediction models for acute kidney injury. Rev Bras Ter Intensiva. 2020;32(1):123-132.	32401985	doi:10.5935/0103-507x.20200018	Abstract, reviews, letter
50	Kallenberger SM, Schmidt C. Forecasting the development of acute kidney injury using a recurrent neural network. Cardiovasc Res. 2019;115(14):e155-e157.	31697360	doi:10.1093/cvr/cvz279	Abstract, reviews, letter
66	Taib BG, Karwath A, Wensley K, Minku L, Gkoutos GV, Moiemen N. Artificial intelligence in the management and treatment of burns: A systematic review and meta-analyses. J Plast Reconstr Aesthet Surg. 2023;77:133-161.	36571960	doi:10.1016/j.bjps.2022.11.049	Abstract, reviews, letter
92	Haredasht FN, Vanhoutte L, Vens C, Pottel H, Viaene L, De Corte W. Validated risk prediction models for outcomes of acute kidney injury: a systematic review. BMC Nephrol. 2023;24(1):133.	37161365	doi:10.1186/s12882-023-03150-0	Abstract, reviews, letter
106	Song Z, Yang Z, Hou M, Shi X. Machine learning in predicting cardiac surgery-associated acute kidney injury: A systemic review and meta-analysis. Front Cardiovasc Med. 2022;9:951881.	36186995	doi:10.3389/fcvm.2022.951881	Abstract, reviews, letter
159	Raina R, Shah R, Nemer P, et al. Using artificial intelligence to predict mortality in AKI patients: a systematic review/meta-analysis. Clin Kidney J. 2024;17(6):sfae150.	38903953	doi:10.1093/ckj/sfae150	Abstract, reviews, letter
167	Zhang H, Wang AY, Wu S, et al. Artificial intelligence for the prediction of acute kidney injury during the perioperative period: systematic review and Meta-analysis of diagnostic test accuracy. BMC Nephrol. 2022;23(1):405.	36536317	doi:10.1186/s12882-022-03025-w	Abstract, reviews, letter
197	Huang J, Chen J, Yang J, et al. Prediction models for acute kidney injury following liver transplantation: A systematic review and critical appraisal. Intensive Crit Care Nurs. 2025;86:103808.	39208611	doi:10.1016/j.iccn.2024.103808	Abstract, reviews, letter
229	Wainstein M, Flanagan E, Johnson DW, Shrapnel S. Systematic review of externally validated machine learning models for predicting acute kidney injury in general hospital patients. Front Nephrol. 2023;3:1220214.	37675372	doi:10.3389/fneph.2023.1220214	Abstract, reviews, letter
231	Du YH, Guan CJ, Li LY, Gan P. Predictive value of machine learning for the risk of acute kidney injury (AKI) in hospital intensive care units (ICU) patients: a systematic review and meta-analysis. PeerJ. 2023;11:e16405.	38034868	doi:10.7717/peerj.16405	Abstract, reviews, letter
247	Lee TH, Chen JJ, Cheng CT, Chang CH. Does Artificial Intelligence Make Clinical Decision Better? A Review of Artificial Intelligence and Machine Learning in Acute Kidney Injury Prediction. Healthcare (Basel). 2021;9(12).	34946388	doi:10.3390/healthcare9121662	Abstract, reviews, letter
340	Wu CC, Poly TN, Weng YC, Lin MC, Islam MM. Machine Learning Models for Predicting Mortality in Critically Ill Patients with Sepsis-Associated Acute Kidney Injury: A Systematic Review. Diagnostics (Basel). 2024;14(15).	39125470	doi:10.3390/diagnostics14151594	Abstract, reviews, letter
353	Lin Y, Shi T, Kong G. Acute Kidney Injury Prognosis Prediction Using Machine Learning Methods: A Systematic Review. Kidney Med. 2025;7(1):100936.	39758155	doi:10.1016/j.xkme.2024.100936	Abstract, reviews, letter
378	Lv X, Liu D, Chen X, et al. Machine learning for the prediction of mortality in patients with sepsis-associated acute kidney injury: a systematic review and meta-analysis. BMC Infect Dis. 2024;24(1):1454.	39709376	doi:10.1186/s12879-024-10380-6	Abstract, reviews, letter
402	Hou J, Cao G, Liu J, Cai L, Zhao L, Li X. Risk factors for acute renal injury caused by contrast media after percutaneous coronary intervention and coronary angiography: A protocol for systematic review and meta-analysis. Medicine (Baltimore). 2022;101(7):e28897.	35363209	doi:10.1097/md.0000000000028897	Abstract, reviews, letter
425	Chongo G, Soldera J. Use of machine learning models for the prognostication of liver transplantation: A systematic review. World J Transplant. 2024;14(1):88891.	38576762	doi:10.5500/wjt.v14.i1.88891	Abstract, reviews, letter
546	Wang D, Zhang W, Luo J, Fang H, Jing S, Mei Z. Prediction models for acute kidney injury in critically ill patients: a protocol for systematic review and critical appraisal. BMJ Open. 2021;11(5):e046274.	34011595	doi:10.1136/bmjopen-2020-046274	Abstract, reviews, letter
727	Najdaghi S, Davani DN, Shafie D, Alizadehasl A. Predictive performance of machine learning models for kidney complications following coronary interventions: a systematic review and meta-analysis. Int Urol Nephrol. 2024.	39477885	doi:10.1007/s11255-024-04257-5	Abstract, reviews, letter
780	Feng Y, Wang AY, Jun M, et al. Characterization of Risk Prediction Models for Acute Kidney Injury: A Systematic Review and Meta-analysis. JAMA Netw Open. 2023;6(5):e2313359.	37184837	doi:10.1001/jamanetworkopen.2023.13359	Abstract, reviews, letter
834	Allen DW, Ma B, Leung KC, et al. Risk Prediction Models for Contrast-Induced Acute Kidney Injury Accompanying Cardiac Catheterization: Systematic Review and Meta-analysis. Can J Cardiol. 2017;33(6):724-736.	28545621	doi:10.1016/j.cjca.2017.01.018	Abstract, reviews, letter

Record Number	Full reference in JMIR/AMA style	PMID	DOI or URL	Reason for exclusion
887	Doyle MP, Woldendorp K, Ng M, et al. Minimally-invasive versus transcatheter aortic valve implantation: systematic review with meta-analysis of propensity-matched studies. J Thorac Dis. 2021;13(3):1671-1683.	33841958	doi:10.21037/jtd-20-2233	Abstract, reviews, letter
1037	Choksi H, Singla A, Yoon P, et al. Outcomes of endovascular, open surgical and autotransplantation techniques for renal artery aneurysm repair: a systematic review and meta-analysis. ANZ J Surg. 2023;93(10):2303-2313.	37522385	doi:10.1111/ans.18628	Abstract, reviews, letter
1098	Judge C, Murphy R, Reddin C, et al. Trends in Adaptive Design Methods in Dialysis Clinical Trials: A Systematic Review. Kidney Med. 2021;3(6):925-941.	34939002	doi:10.1016/j.xkme.2021.08.001	Abstract, reviews, letter
1128	Elkoumi O, Elkoumi A, Elbairy MK, Habib OK, Abdulgadir A. IMPACT OF DEXMEDETOMIDINE ON REDUCING ACUTE KIDNEY INJURY RISK FOLLOWING CORONARY ARTERY BYPASS GRAFTING: A SYSTEMATIC REVIEW AND META-ANALYSIS. 2025;10:57.	Not available	doi:10.1016/j.ekir.2024.11.162	Abstract, reviews, letter
1181	Jawabreh B. Impact of Using Automated Electronic Alert on Acute Kidney Injury Outcomes Systematic Review and meta-analysis. 2025;10:69.	Not available	doi:10.1016/j.ekir.2024.11.184	Abstract, reviews, letter
1241	C C, Kant RAVI, Kandari S, et al. COMPARISON OF CLINICAL PROFILE AND OUTCOMES OF ACUTE KIDNEY INJURY IN DIABETIC AND NON-DIABETIC PATIENTS: A SYSTEMATIC REVIEW AND META-ANALYSIS. 2025;10:609-610.	Not available	doi:10.1016/j.ekir.2024.11.1084	Abstract, reviews, letter
1247	Lucena L, Cavalcante D, Zamora F, et al. IMPACT OF AMINO ACID/PROTEIN THERAPY ON RENAL OUTCOMES AND RECOVERY IN CARDIAC SURGERY PATIENTS: A SYSTEMATIC REVIEW AND META-ANALYSIS. 2025;10:48-49.	Not available	doi:10.1016/j.ekir.2024.11.149	Abstract, reviews, letter
1276	Franca Gois PH, Gois PF, Simpson D, Hedfi S, Shrapnel S, Pole JD. Global Geographic and Socio-Economic Disparities in COVID-Associated Acute Kidney Injury: A Systematic Review and Meta-analysis. 2025;10:611.	Not available	doi:10.1016/j.ekir.2024.11.1085	Abstract, reviews, letter
1285	Franca Gois PH, Gois PF, Simpson D, Hedfi S, Shrapnel S, Pole JD. Prevalence of Acute Kidney Injury throughout COVID-19: A Systematic review and meta-analysis. 2025;10:609.	Not available	doi:10.1016/j.ekir.2024.11.1083	Abstract, reviews, letter
1303	Behera S, Das R. CHRONIC KIDNEY DISEASE AS A PUBLIC HEALTH CHALLENGE IN SOUTH ASIA: A LITERATURE REVIEW. 2025;10:701-702.	Not available	doi:10.1016/j.ekir.2024.11.1245	Abstract, reviews, letter
1304	Majeed MW, Ruelas MG, Finnegan E, et al. Effectiveness of Amino Acid Supplementation in Preventing Acute Kidney Injury Following Cardiac Surgery: A Systematic Review and Meta-Analysis of Randomized Controlled Trials. 2025;10:30-31.	Not available	doi:10.1016/j.ekir.2024.11.125	Abstract, reviews, letter
1564	Wang C, Liu X, Zhang C, Yan R, Li Y, Peng X. The challenges for developing prognostic prediction models for acute kidney injury in hospitalized children: A systematic review. Pediatric Investigation. 2024.	Not available	doi:10.1002/ped4.12458	Abstract, reviews, letter
1591	Cama-Olivares A, Braun CG, Takeuchi T, et al. Artificial Intelligence/Machine Learning Externally Validated Models for AKI Risk-Classification: A Systematic Review and Meta-Analysis. Journal of the American Society of Nephrology. 2024;35:98.	Not available	doi:10.1681/asn.0000000000000223	Abstract, reviews, letter
1848	Raina R, Shah R, Sethi SK, Koyner JL, Neyra JA. Using Artificial Intelligence (AI) to Predict Mortality in AKI Patients: A Systematic Review and Meta-Analysis. Journal of the American Society of Nephrology. 2023;34:98-99.	Not available	https://www.embase.com/search/results?subaction=viewrecord&id=L642701932&from=export	Abstract, reviews, letter
2186	Judge C, Murphy R, Reddin C, et al. Adaptive design methods in dialysis clinical trials-a systematic review. Nephrology Dialysis Transplantation. 2021;36(SUPPL 1):i469.	Not available	doi:10.1093/ndt/gfab098.0032	Abstract, reviews, letter
2789	Saad M, Waqas SA, Aamir J, et al. Fasting Versus Nonfasting Before Cardiac Catheterization: A Systematic Review and Meta-Analysis of Randomized Controlled Trials. AMERICAN JOURNAL OF CARDIOLOGY. 2025;238:40-46.	Not available	doi:10.1016/j.amjcard.2024.11.030	Abstract, reviews, letter
2797	Ghasemi A, Ghasemi M, Rashidian M, Bastan F, Baghaei A. Efficacy of melatonin on drug- or contrast-induced acute kidney injury: a systematic review and GRADE-assessed meta-analysis of experimental and clinical studies. INTERNATIONAL UROLOGY AND NEPHROLOGY. 2025.	Not available	doi:10.1007/s11255-024-04333-w	Abstract, reviews, letter
2972	Chen JJ, Lee TH, Chan MJ, et al. Electronic Alert Systems for Patients With Acute Kidney Injury A Systematic Review and Meta-Analysis. JAMA NETWORK OPEN. 2024;7(8):e2430401.	Not available	doi:10.1001/jamanetworkopen.2024.30401	Abstract, reviews, letter
3039	Usman MS, Bhatt DL, Hameed I, et al. Effect of SGLT2 inhibitors on heart failure outcomes and cardiovascular death across the cardiometabolic disease spectrum: a systematic review and meta-analysis. LANCET DIABETES & ENDOCRINOLOGY. 2024;12(7):447-461.	Not available	doi:10.1016/S2213-8587(24)00102-5	Abstract, reviews, letter
3085	Ceban F, Yan E, Pivetta B, et al. Perioperative adverse events in adult patients with obstructive sleep apnea undergoing ambulatory surgery: An updated systematic review and meta-analysis. JOURNAL OF CLINICAL ANESTHESIA. 2024;96:111464.	Not available	doi:10.1016/j.jclinane.2024.111464	Abstract, reviews, letter
3101	Wilnes B, Castello-Branco B, Branco BC, Sanglard A, de Castro PASV, Simoes-e-Silva AC. Urinary L-FABP as an Early Biomarker for Pediatric Acute Kidney Injury Following Cardiac Surgery with Cardiopulmonary Bypass: A Systematic Review and Meta-Analysis. INTERNATIONAL JOURNAL OF MOLECULAR SCIENCES. 2024;25(9):4912.	Not available	doi:10.3390/ijms25094912	Abstract, reviews, letter
3399	Tan E, Liu D, Perry L, et al. Cell-free DNA as a potential biomarker for acute myocardial infarction: A systematic review and meta-analysis. International journal of cardiology. Heart & vasculature. 2023;47:101246-101246.	37560328	doi:10.1016/j.ijcha.2023.101246	Abstract, reviews, letter
3471	Chi HC, Liu Y, Tan CC, Zhang YC, Tan L, Xu W. Adult Renal Dysfunction and Risk of Dementia or Cognitive Decline: Brain-Kidney Axis Hypothesis Based on a Systematic Review and Meta-Analysis. JPAD-JOURNAL OF PREVENTION OF ALZHEIMERS DISEASE. 2023;10(3):443-452.	Not available	doi:10.14283/jpad.2023.35	Abstract, reviews, letter
3472	Lapp L, Roper M, Kavanagh K, Bouamrane MM, Schraag S. Dynamic Prediction of Patient Outcomes in the Intensive Care Unit: A Scoping Review of the State-of-the-Art. JOURNAL OF INTENSIVE CARE MEDICINE. 2023;38(7):575-591.	Not available	doi:10.1177/08850666231166349	Abstract, reviews, letter
3505	Meena J, Thomas CC, Kumar J, Mathew G, Bagga A. Biomarkers for prediction of acute kidney injury in pediatric patients: a systematic review and meta-analysis of diagnostic test accuracy studies. PEDIATRIC NEPHROLOGY. 2023;38(10):3241-3251.	Not available	doi:10.1007/s00467-023-05891-4	Abstract, reviews, letter

Record Number	Full reference in JMIR/AMA style	PMID	DOI or URL	Reason for exclusion
3610	Huang Y, Huang S, Zhuo X, Lin M. Predictive value of suPAR in AKI: a systematic review and meta-analysis. CLINICAL AND EXPERIMENTAL NEPHROLOGY. 2022;46030.	Not available	doi:10.1007/s10157-022-02300-2	Abstract, reviews, letter
3812	Sharrod-Cole H, Fenn J, Gama R, Ford C, Giri R, Luckraz H. Utility of plasma NGAL for the diagnosis of AKI following cardiac surgery requiring cardiopulmonary bypass: a systematic review and meta-analysis. SCIENTIFIC REPORTS. 2022;12(1):6436.	Not available	doi:10.1038/s41598-022-10477-5	Abstract, reviews, letter
3938	Brazzelli M, Aucott L, Aceves-Martins M, et al. Biomarkers for assessing acute kidney injury for people who are being considered for admission to critical care: a systematic review and cost-effectiveness analysis. HEALTH TECHNOLOGY ASSESSMENT. 2022;26(7):1-285.	Not available	doi:10.3310/UGEZ4120	Abstract, reviews, letter
4023	Mukhopadhyay K, Chakrabarty S, Chatterjee C, Misra SC. Mortality and complications of scrub typhus in the paediatric population: a systematic review and meta-analysis. TRANSACTIONS OF THE ROYAL SOCIETY OF TROPICAL MEDICINE AND HYGIENE. 2021;115(11):1234-1246.	Not available	doi:10.1093/trstmh/trab143	Abstract, reviews, letter
4257	Albert C, Zapf A, Haase M, et al. Neutrophil Gelatinase-Associated Lipocalin Measured on Clinical Laboratory Platforms for the Prediction of Acute Kidney Injury and the Associated Need for Dialysis Therapy: A Systematic Review and Meta-analysis. AMERICAN JOURNAL OF KIDNEY DISEASES. 2020;76(6):826-+.	Not available	doi:10.1053/j.ajkd.2020.05.015	Abstract, reviews, letter
4284	Barton AL, Williams SBM, Dickinson SJ, Parry RG, Pollard A. Acute Kidney Injury in Primary Care: A Review of Patient Follow-Up, Mortality, and Hospital Admissions following the Introduction of an AKI Alert System. NEPHRON. 2020;144(10):498-505.	Not available	doi:10.1159/000509855	Abstract, reviews, letter
4709	Pozzoli S, Simonini M, Manunta P. Predicting acute kidney injury: current status and future challenges. JOURNAL OF NEPHROLOGY. 2018;31(2):209-223.	Not available	doi:10.1007/s40620-017-0416-8	Abstract, reviews, letter
4732	Neyra JA, Leaf DE. Risk Prediction Models for Acute Kidney Injury in Critically Ill Patients: Opus in Progressu. NEPHRON. 2018;140(2):99-104.	Not available	doi:10.1159/000490119	Abstract, reviews, letter
4791	Chatterjee S, Kundu A, Mukherjee D, et al. Risk of contrast-induced acute kidney injury in ST-elevation myocardial infarction patients undergoing multi-vessel intervention-meta-analysis of randomized trials and risk prediction modeling study using observational data. CATHETERIZATION AND CARDIOVASCULAR INTERVENTIONS. 2017;90(2):205-212.	Not available	doi:10.1002/ccd.26928	Abstract, reviews, letter
4863	Alavijeh OS, Bansal J, Hadfield K, Laing C, Dawnay A. Implementation of an Automated Primary Care Acute Kidney Injury Warning System: A Quantitative and Qualitative Review of 2 Years of Experience. NEPHRON. 2017;135(3):189-195.	Not available	doi:10.1159/000452928	Abstract, reviews, letter
5164	Heung M, Chawla LS. Predicting progression to chronic kidney disease after recovery from acute kidney injury. CURRENT OPINION IN NEPHROLOGY AND HYPERTENSION. 2012;21(6):628-634.	Not available	doi:10.1097/MNH.0b013e3283588f24	Abstract, reviews, letter
5274	Haase M, Bellomo R, Devarajan P, Schlattmann P, Haase-Fielitz A, NMAI Grp. Accuracy of Neutrophil Gelatinase-Associated Lipocalin (NGAL) in Diagnosis and Prognosis in Acute Kidney Injury: A Systematic Review and Meta-analysis. AMERICAN JOURNAL OF KIDNEY DISEASES. 2009;54(6):1012-1024.	Not available	doi:10.1053/j.ajkd.2009.07.020	Abstract, reviews, letter

2. Not English studies (n=6)

Record Number	Full reference in JMIR/AMA style	PMID	DOI or URL	Reason for exclusion
481	Tang CQ, Li JQ, Xu DY, et al. [Comparison of machine learning method and logistic regression model in prediction of acute kidney injury in severely burned patients]. Zhonghua Shao Shang Za Zhi. 2018;34(6):343-348.	29961290	doi:10.3760/cma.j.issn.1009-2587.2018.06.006	Not English studies
1008	Lin K, Xie JQ, Hu YH, Kong GL. [Application of support vector machine in predicting in-hospital mortality risk of patients with acute kidney injury in ICU]. Beijing Da Xue Xue Bao Yi Xue Ban. 2018;50(2):239-244.	29643521	Not available	Not English studies
4324	Li Y, Chen XH, Wang YM, et al. Application of LASSO regression and Bayesian networks in exploring related factors of acute kidney injury (AKI) in cancer patients. Fudan Xuebao (Yixueban). 2020;47(4):521-530.	Not available	doi:10.3969/j.issn.1672-8467.2020.04.009	Not English studies
4336	Chi R, Liang M, Zou Q, Li C, Zhou H, Jian Z. [Construction and validation of a decision tree based on biomarkers for predicting severe acute kidney injury in critically ill patients]. Zhonghua wei zhong bing ji jiu yi xue. 2020;32(6):721-725.	32684220	doi:10.3760/cma.j.cn121430-20200509-00371	Not English studies
4419	Mironova OI, Staroverov II, Sivakova OA, Fomin VV. Contrast-induced acute kidney injury in patients with stable coronary artery disease: the most important risk factors and prevalence. TERAPEVTICHESKII ARKHIV. 2020;92(9):44-48.	Not available	doi:10.26442/00403660.2020.09.000751	Not English studies
5942	Dong Z, Shi L, Ye L, Xu Z, Zhou L. Risk factors analysis of renal replacement therapy after liver transplantation and prognosis effect of initial treatment time. Zhonghua wei zhong bing ji jiu yi xue. 2018;30(11):1056-1060.	Not available	doi:10.3760/cma.j.issn.2095-4352.2018.011.009	Not English studies

3. Sample size < 200 (n=25)

Record Number	Full reference in JMIR/AMA style	PMID	DOI or URL	Reason for exclusion
145	Xue X, Liu Z, Xue T, Chen W, Chen X. Machine learning for the prediction of acute kidney injury in patients after cardiac surgery. Front Surg. 2022;9:946610.	36157418	doi:10.3389/fsurg.2022.946610	Sample size < 200
150	Yang C, Yang C, Lin SP, et al. A Prediction Model for Acute Kidney Injury in Adult Patients With Minimal Change Disease. Front Med (Lausanne). 2022;9:862160.	35685412	doi:10.3389/fmed.2022.862160	Sample size < 200
156	Lofgren L, Silverton N, Kuck K. Combining Machine Learning and Urine Oximetry: Towards an Intraoperative AKI Risk Prediction Algorithm. J Clin Med. 2023;12(17).	37685632	doi:10.3390/jcm12175567	Sample size < 200
202	Imamura Y, Kiyota N, Tahara M, et al. Risk prediction model for cisplatin-induced acute kidney injury in patients with head and neck cancer receiving chemoradiotherapy: A re-analysis of a phase II/III JCOG1008 trial. Oral Oncol. 2024;154:106868.	38820889	doi:10.1016/j.oraloncology.2024.106868	Sample size < 200
265	Kong X, Zhao L, Pan Z, Li H, Wei G, Wang Q. Acute renal injury after aortic arch reconstruction with cardiopulmonary bypass for children: prediction models by machine learning of a retrospective cohort study. Eur J Med Res. 2023;28(1):499.	37941080	doi:10.1186/s40001-023-01455-2	Sample size < 200
274	Zhu K, Song H, Zhang Z, et al. Acute kidney injury in solitary kidney patients after partial nephrectomy: incidence, risk factors and prediction. Transl Androl Urol. 2020;9(3):1232-1243.	32676406	doi:10.21037/tau.2020.03.45	Sample size < 200
355	Susianti H, Asmoro AA, Sujarwoto, et al. Acute Kidney Injury Prediction Model Using Cystatin-C, Beta-2 Microglobulin, and Neutrophil Gelatinase-Associated Lipocalin Biomarker in Sepsis Patients. Int J Nephrol Renovasc Dis. 2024;17:105-112.	38562530	doi:10.2147/ijnrd.S450901	Sample size < 200
388	Musiał K, Stojanowski J, Augustynowicz M, Miśkiewicz-Migoń I, Kałwak K, Ussowicz M. Assessment of Risk Factors for Acute Kidney Injury with Machine Learning Tools in Children Undergoing Hematopoietic Stem Cell Transplantation. J Clin Med. 2024;13(8).	38673539	doi:10.3390/jcm13082266	Sample size < 200
574	Yao X, Zhang L, Huang L, Chen X, Geng L, Xu X. Development of a Nomogram Model for Predicting the Risk of In-Hospital Death in Patients with Acute Kidney Injury. Risk Manag Healthc Policy. 2021;14:4457-4468.	34754252	doi:10.2147/rmhp.S321399	Sample size < 200
599	Yang X, Tian X, Liu J, et al. [Establishment of a prognostic nomogram model for predicting acute renal injury in patients with moderate and severe burns]. Zhonghua Wei Zhong Bing Ji Jiu Yi Xue. 2023;35(7):736-740.	37545452	doi:10.3760/cma.j.cn121430-20220616-0057 7	Sample size < 200
655	Kim JY, Kim KY, Yee J, Gwak HS. Risk Scoring System for Vancomycin-Associated Acute Kidney Injury. Front Pharmacol. 2022;13:815188.	35330832	doi:10.3389/fphar.2022.815188	Sample size < 200
690	Zhu L, Cai J, Fang J, et al. Nomogram Model for Cardiac Surgery-Associated Acute Kidney Injury Based on Clinical Characteristics Combined with Plasma suPAR. Int J Gen Med. 2024;17:3181-3192.	39049830	doi:10.2147/ijgm.S464904	Sample size < 200
768	Wu Z, Wang Y, He L, et al. Development of a nomogram for the prediction of acute kidney injury after liver transplantation: a model based on clinical parameters and postoperative cystatin C level. Ann Med. 2023;55(2):2259410.	37734410	doi:10.1080/07853890.2023.2259410	Sample size < 200
854	Bredt LC, Peres LAB, Risso M, Barros L. Risk factors and prediction of acute kidney injury after liver transplantation: Logistic regression and artificial neural network approaches. World J Hepatol. 2022;14(3):570-582.	35582300	doi:10.4254/wjh.v14.i3.570	Sample size < 200
877	Sun Y, Xiao Z, Zhao H, An Y. Urinary dickkopf-3 as a predictor for postoperative acute kidney injury in the intensive care unit. Am J Med Sci. 2024.	39561890	doi:10.1016/j.amjms.2024.11.003	Sample size < 200
3535	Zeng J, Li Q, Wu Q, et al. A Novel Online Calculator Predicting Acute Kidney Injury After Liver Transplantation: A Retrospective Study. TRANSPLANT INTERNATIONAL. 2023;36:10887.	Not available	doi:10.3389/ti.2023.10887	Sample size < 200
4705	Borasino S, Wall KM, Crawford JH, et al. Furosemide Response Predicts Acute Kidney Injury After Cardiac Surgery in Infants and Neonates. PEDIATRIC CRITICAL CARE MEDICINE. 2018;19(4):310-317.	Not available	doi:10.1097/PCC.0000000000001478	Sample size < 200
4744	Gameiro J, Agapito Fonseca J, Monteiro Dias J, et al. Prediction of acute kidney injury in cirrhotic patients: a new score combining renal, liver and inflammatory markers. International journal of nephrology and renovascular disease. 2018;11:149-154.	29731657	doi:10.2147/IJNRD.S163602	Sample size < 200
4928	Martensson J, Jonsson N, Glassford NJ, et al. Plasma endostatin may improve acute kidney injury risk prediction in critically ill patients. ANNALS OF INTENSIVE CARE. 2016;6:6.	Not available	doi:10.1186/s13613-016-0108-x	Sample size < 200
4981	Mizuno A, Ohde S, Nishizaki Y, Komatsu Y, Niwa K. Additional value of the red blood cell distribution width to the Mehran risk score for predicting contrast-induced acute kidney injury in patients with ST-elevation acute myocardial infarction. JOURNAL OF CARDIOLOGY. 2015;66(46024):41-45.	Not available	doi:10.1016/j.jjcc.2014.09.006	Sample size < 200
5065	Kidher E, Harling L, Ashrafian H, et al. Pulse wave velocity and neutrophil gelatinase-associated lipocalin as predictors of acute kidney injury following aortic valve replacement. JOURNAL OF CARDIOTHORACIC SURGERY. 2014;9:89.	Not available	doi:10.1186/1749-8090-9-89	Sample size < 200
5098	Rodriguez E, Soler MJ, Rap O, Barrios C, Orfila MA, Pascual J. Risk Factors for Acute Kidney Injury in Severe Rhabdomyolysis. PLOS ONE. 2013;8(12):e82992.	Not available	doi:10.1371/journal.pone.0082992	Sample size < 200
5130	Romano TG, Schmidtbauer I, de Queiroz Silva FM, Pompilio CE, Carneiro D'Albuquerque LA, Macedo E. Role of MELD Score and Serum Creatinine as Prognostic Tools for the Development of Acute Kidney Injury after Liver Transplantation. PLOS ONE. 2013;8(5):e64089.	Not available	doi:10.1371/journal.pone.0064089	Sample size < 200
5190	Katagiri D, Doi K, Honda K, et al. Combination of Two Urinary Biomarkers Predicts Acute Kidney Injury After Adult Cardiac Surgery. ANNALS OF THORACIC SURGERY. 2012;93(2):577-583.	Not available	doi:10.1016/j.athoracsur.2011.10.048	Sample size < 200

Record Number	Full reference in JMIR/AMA style	PMID	DOI or URL	Reason for exclusion
5940	Goldenberg I, Chonchol M, Guetta V. Reversible acute kidney injury following contrast exposure and the risk of long-term mortality. American journal of nephrology. 2009;29(2):136-144.	Not available	doi:10.1159/000151772	Sample size < 200

4. Full text not available (n=75)

Record Number	Full reference in JMIR/AMA style	PMID	DOI or URL	Reason for exclusion
17	Kurokawa T, Tsurita G, Tanimoto T, Yamada T, Ferrone S. Risk Prediction Model for Cisplatin-Associated Acute Kidney Injury. J Clin Oncol. 2018;36(23):2453-2454.	29958036	doi:10.1200/jco.2018.77.8761	Full text not available
64	Zhuo XY, Lei SH, Zhao BC. Preoperative risk prediction models for acute kidney injury after noncardiac surgery: are all models wrong, or can some be recalibrated and become useful? Response to Br J Anaesth 2024;133: 658-659. Br J Anaesth. 2024;133(3):660-661.	38965011	doi:10.1016/j.bja.2024.06.020	Full text not available
100	Hylton AE, Stewart KE, Tanaka KA, Butt AL. Preoperative risk prediction models for acute kidney injury after noncardiac surgery: are all models wrong, or can some be recalibrated and become useful? Comment on Br J Anaesth 2024;133: 508-518. Br J Anaesth. 2024;133(3):658-659.	38821751	doi:10.1016/j.bja.2024.05.002	Full text not available
242	Vagliano I, Byrne Salsas C, Wünn T, Schut MC. External Validation and Transportability of Models to Predict Acute Kidney Injury in the Intensive Care Unit. Stud Health Technol Inform. 2022;295:148-151.	35773829	doi:10.3233/shti220683	Full text not available
255	Wu W, Zhang Y, Zhang Y, Qu X, Zhang Z, Zhang R. Machine-learning based prediction model for acute kidney injury induced by multiple wasp stings. Toxicon. 2024;250:108112.	39349067	doi:10.1016/j.toxicon.2024.108112	Full text not available
280	Tangri N, Ferguson TW. Role of artificial intelligence in the diagnosis and management of kidney disease: applications to chronic kidney disease and acute kidney injury. Curr Opin Nephrol Hypertens. 2022;31(3):283-287.	35190505	doi:10.1097/mnh.0000000000000787	Full text not available
349	Thongprayoon C, Vaitla P, Nissaisorakarn V, et al. Clinically Distinct Subtypes of Acute Kidney Injury on Hospital Admission Identified by Machine Learning Consensus Clustering. Med Sci (Basel). 2021;9(4).	34698185	doi:10.3390/medsci9040060	Full text not available
441	Hong H, Chen Y, Zhou L, Bao J, Ma J. Risk factors analysis and construction of predictive models for acute kidney injury in overweight patients receiving vancomycin treatment. Expert Opin Drug Saf. 2024;46032.	39140731	doi:10.1080/14740338.2024.2393285	Full text not available
449	Itenov TS, Berthelsen RE, Jensen JU, et al. Predicting recovery from acute kidney injury in critically ill patients: development and validation of a prediction model. Crit Care Resusc. 2018;20(1):54-60.	29458322	Not available	Full text not available
471	Gaytán-Muñoz GA, Villarreal-Ríos E, Vargas-Daza ER, Martínez-González L, Galicia-Rodríguez L. [Risk factors for developing acute kidney injury in elderly patients]. Rev Med Inst Mex Seguro Soc. 2019;57(1):15-20.	31071250	Not available	Full text not available
822	Zhang Y, Li X, Zhang S, et al. Clinical Features and Predictive Nomogram of Acute Kidney Injury in Aging Population Infected with SARS-CoV-2 Omicron Variant. J Inflamm Res. 2023;16:2967-2978.	37484995	doi:10.2147/jir.S413318	Full text not available
1058	Zhang J, Qi M, Ma L, Zhang K, Liu D, Liu D. [Construction and validation of a risk nomogram for sepsis-associated acute kidney injury in intensive care unit]. Zhonghua Wei Zhong Bing Ji Jiu Yi Xue. 2024;36(8):801-807.	39238403	doi:10.3760/cma.j.cn121430-20240221-00150	Full text not available
1687	Li C, Jia L, Zang J, et al. Risk factors analysis and prediction nomogram establishment of acute kidney injury in hip fracture patients with severe underlying diseases. Chinese Journal of Orthopaedics. 2023;43(16):1094-1103.	Not available	doi:10.3760/cma.j.cn121113-20230326-00163	Full text not available
2078	Vasquez-Rios G, Oh W, Lee S, et al. Machine Learning Models Uncover Subphenotypes of AKI With Unique Signatures That Associate With Differing Clinical Outcomes. Journal of the American Society of Nephrology. 2022;33:3.	Not available	https://www.embase.com/search/results?subaction=viewrecord&id=L639545491&from=export	Full text not available
2293	Yan P, Duan S, Duan X, Wu X, Tang C. Outcomes and risk prediction of acute kidney disease after acute kidney injury in hospitalized critically ill and noncritically ill patients. Nephrology. 2020;25(SUPPL 1):46338.	Not available	doi:10.1111/nep.13772	Full text not available
2313	Filiberto AC, Ozrazgat-Baslanti T, Peng YC, et al. Perioperative Acute Kidney Injury in Vascular Surgery Patients: Prevalence and Predictive Strategies. Journal of Vascular Surgery. 2020;72(1):e253-e254.	Not available	doi:10.1016/j.jvs.2020.04.420	Full text not available
2323	Kim HE, Yang H, Kim S, Kim K. Real-time clinical decision support to predict and manage in-hospital acute kidney injury utilizingrecurrent neural network. Nephrology Dialysis Transplantation. 2020;35(SUPPL 3):iii241.	Not available	doi:10.1093/ndt/gfaa139.P0616	Full text not available
2341	Safadi S, Hommos MS, Enders FT, Lieske JC, Kashani KB. Risk Factors for Acute Kidney Injury in Hospitalized Non–Critically Ill Patients: A Population-Based Study. Mayo Clinic Proceedings. 2020;95(3):459-467.	Not available	doi:10.1016/j.mayocp.2019.06.011	Full text not available
2365	Calvert J, Allen AO, Le SH, et al. Development and validation of a convolutional neural network model for intensive care unit AKI prediction. Journal of the American Society of Nephrology. 2020;31:16.	Not available	https://www.embase.com/search/results?subaction=viewrecord&id=L633697840&from=export	Full text not available
2366	Singh G, Hu Y, Riviello D, et al. Early prediction of hospital-acquired AKI from electronic health records using machine learning. Journal of the American Society of Nephrology. 2020;31:62.	Not available	https://www.embase.com/search/results?subaction=viewrecord&id=L633698021&from=export	Full text not available
2372	Zhang Q, Guo S, Liang X, Chen Y, Wu Y, Fu L. Risk prediction and key influence factors analysis of acute kidney injury in inpatients. Chinese Journal of Biomedical Engineering. 2019;38(6):702-710.	Not available	doi:10.3969/j.issn.0258-8021.2019.06.008	Full text not available
2380	Golato M, Matera S, Impicciatore G. Acute kidney injury (AKI) biomarkers. Part two: Traditional, new and potential biomarkers of AKI. Rivista Italiana della Medicina di Laboratorio. 2019;15(4):263-276.	Not available	doi:10.23736/S1825-859X.19.00041-0	Full text not available
2388	Rothstein WB, Navarrete SB, Scott MJ. Predictive Hemodynamic Monitoring in Cardiac Surgery: An Observational Validation Study of the Hypotension Prediction Index. Journal of the American College of Surgeons. 2019;229(4):S48.	Not available	doi:10.1016/j.jamcollsurg.2019.08.119	Full text not available

Record Number	Full reference in JMIR/AMA style	PMID	DOI or URL	Reason for exclusion
2389	Tsutsui R, Johnston J, Felix C, et al. TCT-615 A Supervised Machine Learning Approach for Predicting Acute Kidney Injury Following Percutaneous Coronary Intervention. Journal of the American College of Cardiology. 2019;74(13):B604.	Not available	doi:10.1016/j.jacc.2019.08.730	Full text not available
2440	Rakocz N, Hill BL, Sankararaman S, Halperin E, Hofer I. Deep learning to predict postoperative acute kidney injury. Anesthesia and Analgesia. 2019;128(4):67.	Not available	doi:10.1213/01.ane.0000554596.75851.9b	Full text not available
2459	Dong J, Thapa-Chhetry B, Cho BG, Newth C. A real-time pediatric acute kidney injury prediction algorithm. Critical Care Medicine. 2019;47(1).	Not available	https://www.embase.com/search/results?subaction=viewrecord&id=L629630461&from=export	Full text not available
2466	Sato N, Uchino E, Kojima R, Hiragi S, Yanagita M, Okuno Y. Prediction of AKI in ICU using routinely collected data by machine-learning algorithms and its visualization. Journal of the American Society of Nephrology. 2019;30:127.	Not available	doi:10.1681/asn.2018050531	Full text not available
2468	Uchino E, Kume K, Iwamoto K, et al. Development of machine learning models for predicting AKI onset using electronic medical records. Journal of the American Society of Nephrology. 2019;30:47.	Not available	https://www.embase.com/search/results?subaction=viewrecord&id=L633768941&from=export	Full text not available
2471	Neyra JA, Smith TD, Ortiz-Soriano VM, et al. Identification and performance evaluation of AKI trajectory subtypes associated with mortality and kidney recovery in critically ill patients. Journal of the American Society of Nephrology. 2019;30:805.	Not available	https://www.embase.com/search/results?subaction=viewrecord&id=L633770888&from=export	Full text not available
2487	Kalisvaart M, Schlegel A, Umbro I, et al. The AKI-Predict-Score: a new prediction model for acute kidney injury after liver transplantation. HPB. 2018;20:S174-S175.	Not available	doi:10.1016/j.hpb.2018.06.006	Full text not available
2507	Bishara A, Kothari R, Bokoch M, Niemann C, Adelmann D. Predicting acute kidney injury after orthotopic liver transplantation using a recurrent neural network. American Journal of Transplantation. 2018;18:960.	Not available	doi:10.1111/ajt.14918	Full text not available
2559	Han L, Luo Q, Zhou F, et al. The ITRAQ technology identifies biomarkers for the early diagnosis of contrast-induced AKI. Journal of the American Society of Nephrology. 2017;28:430.	Not available	https://www.embase.com/search/results?subaction=viewrecord&id=L633699346&from=export	Full text not available
2561	Zhou F, Luo Q, Shen G, et al. Identification of urine apolipoprotein A-I as a biomarker for early diagnosis of AKI following percutaneous coronary intervention by itraq-based quantitative proteomics. Journal of the American Society of Nephrology. 2017;28:697.	Not available	https://www.embase.com/search/results?subaction=viewrecord&id=L633700224&from=export	Full text not available
2564	Lee S, Park S, Lee A, Chin HJ, Na KY, Kim S. Risk factors and long-term prognosis of post-operative AKI under non-general anesthesia. Journal of the American Society of Nephrology. 2017;28:692.	Not available	https://www.embase.com/search/results?subaction=viewrecord&id=L633701942&from=export	Full text not available
2588	Wu VC, Wang JJ, Wu KD. Urinary biomarkers predicts advanced acute kidney injury after cardiovascular surgery. Nephrology Dialysis Transplantation. 2017;32:iii173.	Not available	doi:10.1093/ndt/gfx143	Full text not available
2594	Kroner PT, Jirapinyo P, Abougergi MS, Thompson CC. A nationwide trend analysis of patients admitted for acute pancreatitis: Ten year evaluation of in hospital mortality, clinical outcomes, and the impact of acute kidney injury. Gastroenterology. 2017;152(5):S485.	Not available	doi:10.1016/s0016-5085(17)31807-3	Full text not available
2612	Jonsson AJ, Lund SH, Palsson R, Indridason OS, Kristjansdottir I. Computerized Algorithms Compared to a Nephrologists Diagnosis of Acute Kidney Injury. Journal of the American Society of Nephrology. 2016;27:571A.	Not available	https://www.embase.com/search/results?subaction=viewrecord&id=L641131036&from=export	Full text not available
2615	Bhalla A, Leonberg-Yoo AK, Rao M. Risk Prediction Model for Kidney Injury in Two Stage Revision Arthroplasty with Antibiotic Loaded Spacers for Prosthetic Joint Infections. Journal of the American Society of Nephrology. 2016;27:258A.	Not available	https://www.embase.com/search/results?subaction=viewrecord&id=L641134828&from=export	Full text not available
2616	Park S, Kim DK, Joo KW, Kim YS, Lee H. Prediction Model for Postoperative Acute Kidney Injury after Noncardiac Major Surgery Using Machine Learning. Journal of the American Society of Nephrology. 2016;27:248A.	Not available	https://www.embase.com/search/results?subaction=viewrecord&id=L641135106&from=export	Full text not available
2617	Nangalia V, Connell A, Brown ST, et al. Prediction of Death and Renal Replacement Therapy in Hospitalised Patients with Acute Kidney Injury. Journal of the American Society of Nephrology. 2016;27:251A.	Not available	https://www.embase.com/search/results?subaction=viewrecord&id=L641135270&from=export	Full text not available
2618	Hessey E, Perreault S, Roy L, et al. Acute Kidney Injury Is Associated with Long-Term Renal Outcomes Using Administrative Data in Children. Journal of the American Society of Nephrology. 2016;27:253A.	Not available	https://www.embase.com/search/results?subaction=viewrecord&id=L641135364&from=export	Full text not available
2620	Wybraniec M, Chudek J, Bozentowicz-Wikarek M, Mizia-Stec K. Analysis of the significance of ultrasonographic renal blood flow parameters for the prediction of contrast-induced acute kidney injury after coronary angiography. Kardiologia Polska. 2016;74:258-259.	Not available	https://www.embase.com/search/results?subaction=viewrecord&id=L614983350&from=export	Full text not available
2624	Wybraniec M, Chudek J, Bozentowicz-Wikarek M, Mizia-Stec K. Intra-renal ultrasonographic blood flow parameters as early diagnostic predictors of contrast-induced acute kidney injury following coronary angiography. European Heart Journal. 2016;37:58.	Not available	doi:10.1093/eurheartj/ehw431	Full text not available

Record Number	Full reference in JMIR/AMA style	PMID	DOI or URL	Reason for exclusion
2654	Semanik MG, Hingorani SR. Identification of a Pediatric Inpatient Cohort at Risk for Developing Acute Kidney Injury Using Machine Learning Methods. Journal of the American Society of Nephrology. 2015;26:101A-102A.	Not available	https://www.embase.com/search/results?subaction=viewrecord&id=L641102763&from=export	Full text not available
2687	Seelhammer TG, Engoren M, Maile M, Jewell E, Heung M, Haft J. Estimated GFR and AKI outcome prediction model for post cardiac surgery patients. Anesthesia and Analgesia. 2014;118(5):S94.	Not available	doi:10.1213/01.ane.0000453015.43124.d0	Full text not available
2701	Pike F, Murugan R, Keener C, Wen X, Elder M, Kellum J. Biomarkers augment prediction of renal recovery and mortality in critically ill patients on dialysis. Critical Care Medicine. 2013;41(12):A10.	Not available	doi:10.1097/01.ccm.0000439220.90672.57	Full text not available
2703	Bihorac A, Korenkevych D, Baslanti TO, et al. Database communication enables machine learning classifiers to predict postoperative AKI in ICU. Critical Care Medicine. 2013;41(12):A176.	Not available	doi:10.1097/01.ccm.0000439952.88773.77	Full text not available
2719	Wang YN, Cheng H, Chen YP. Development and validation of a prognostic risk score foracute renal injury in patients hospitalized for acute decompensated heart failure. Nephrology Dialysis Transplantation. 2013;28:i335.	Not available	doi:10.1093/ndt/gft129	Full text not available
2739	Tsai TT, Patel UD, Chang TI, et al. A validated contemporary risk model of acute kidney injury in patients undergoing percutaneous coronary interventions: Insights from the NCDR Cath-PCI registry. Circulation: Cardiovascular Quality and Outcomes. 2012;5(3).	Not available	https://www.embase.com/search/results?subaction=viewrecord&id=L71256121&from=export	Full text not available
3212	Lofgren LR, Kuck K, MacLeod RS, Dorval AD, Hitchcock RW, Silverton NAZ. A Novel Monitoring Method for Early Detection and Intervention for Acute Kidney Injury. 2024.	Not available	Not available	Full text not available
3221	Cao JZ. Development and Validation of Transportable, Clinically Applicable and Scalable Machine Learning Models for Acute Kidney Injury. 2024.	Not available	Not available	Full text not available
3642	Chen S, Pan G, Yang J, Wang L. Risk of early acute kidney injury after cardiopulmonary bypass supporting cardiac surgery predicted by platelet lymphocyte ratio combined with neutrophil lymphocyte ratio. Wuhan Daxue Xuebao (Yixue Ban). 2022;43(6):957-961,992.	Not available	doi:10.14188/j.1671.8852.2022.0404	Full text not available
3906	da Silva DADZ. Digital Tools in Preventing Acute Kidney Injury. 2022.	Not available	Not available	Full text not available
3907	Karanam LZ. Continuous Anticipation of Acute Kidney Injury in the ICU. 2022.	Not available	Not available	Full text not available
4372	Saraiva IE, Ortiz-Soriano VM, Mei X, et al. Continuous renal replacement therapy in critically ill patients with acute on chronic liver failure and acute kidney injury: A retrospective cohort study. CLINICAL NEPHROLOGY. 2020;93(4):187-194.	Not available	doi:10.5414/CN109983	Full text not available
4494	Tsutsui R, Johnston J, Felix C, et al. A Supervised Machine Learning Approach for Predicting Acute Kidney Injury Following Percutaneous Coronary Intervention. JOURNAL OF THE AMERICAN COLLEGE OF CARDIOLOGY. 2019;74(13):B604-B604.	Not available	doi:10.1016/j.jacc.2019.08.730	Full text not available
4641	Butt K, Dsouza J, Nguyen M, Maqsood A, Jayakumaran J, Abusaada K. The Role of Neutrophil to Lymphocyte Ratio and Platelet to Lymphocyte Ratio in Predicting Contrast Induced Acute Kidney Injury in Patients Presenting With Acute Coronary Syndrome. CIRCULATION. 2018;138.	Not available	Not available	Full text not available
4851	Mao Y, Qin ZH. Risk factors of acute kidney injury and dialysis among patients attending intensive care units in China. INTERNATIONAL JOURNAL OF CLINICAL AND EXPERIMENTAL MEDICINE. 2017;10(8):12056-12067.	Not available	Not available	Full text not available
4859	Wang S, Li S, Sang G, et al. Long-term outcomes of hospital- and community-acquired acute kidney injury. INTERNATIONAL JOURNAL OF CLINICAL AND EXPERIMENTAL MEDICINE. 2017;10(6):9482-9487.	Not available	Not available	Full text not available
4996	Metzger J, Mullen W, Mischak H, Klingele M. VALIDATION OF A PREVIOUSLY ESTABLISHED URINARY PEPTIDE MARKER MODEL FOR EARLY AND ACCURATE PREDICTION OF ACUTE KIDNEY INJURY IN PATIENTS AFTER CARDIAC SURGERY. NEPHROLOGY DIALYSIS TRANSPLANTATION. 2015;30.	Not available	doi:10.1093/ndt/gfv172.19	Full text not available
5003	Bucholz EM, Whitlock RP, Zappitelli M, et al. Cardiac Biomarkers and Acute Kidney Injury After Cardiac Surgery. PEDIATRICS. 2015;135(4):E945-E956.	Not available	doi:10.1542/peds.2014-2949	Full text not available
5118	Chao CT, Lin YF, Tsai HB, Wu VC, Ko WJ. Acute Kidney Injury Network Staging in Geriatric Postoperative Acute Kidney Injury Patients: Shortcomings and Improvements. JOURNAL OF THE AMERICAN COLLEGE OF SURGEONS. 2013;217(2):240-250.	Not available	doi:10.1016/j.jamcollsurg.2013.03.024	Full text not available
5191	Parolari A, Pesce LL, Pacini D, et al. Risk Factors for Perioperative Acute Kidney Injury After Adult Cardiac Surgery: Role of Perioperative Management. ANNALS OF THORACIC SURGERY. 2012;93(2):584-591.	Not available	doi:10.1016/j.athoracsur.2011.09.073	Full text not available
5502	Grimm JC, Lui C, Kilic A, et al. A risk score to predict acute renal failure in adult patients after lung transplantation. Annals of thoracic surgery. 2015;99(1):251-257.	Not available	doi:10.1016/j.athoracsur.2014.07.073	Full text not available
5523	Bhatraju P, Russell J, Walley K, Liles W, Himmelfarb J, Wurfel M. Acute kidney injury subphenotypes respond differently to vasopressin therapy in septic shock. Critical care medicine. 2019;47(1).	Not available	https://www.cochranelibrary.com/central/doi/10.1002/central/CN-01995824/full	Full text not available
5598	Prowle JR, Calzavacca P, Licari E, et al. Combination of biomarkers for diagnosis of acute kidney injury after cardiopulmonary bypass. Renal failure. 2015;37(3):408-416.	Not available	doi:10.3109/0886022X.2014.1001303	Full text not available
5639	Wilson FP, Biswas A, Parikh CR, et al. Development and Validation of a Model to Improve Targeting of Electronic Alerts for Hospitalized Patients with Acute Kidney Injury. Journal of the American Society of Nephrology : JASN. 2016;27:2B.	Not available	https://www.cochranelibrary.com/central/doi/10.1002/central/CN-02557663/full	Full text not available
5640	Singh K, Zhang X, Yin M, et al. Development and validation of a model to predict in-hospital AKI among hospitalized us veterans. Journal of the American Society of Nephrology : JASN. 2020;31:62.	Not available	https://www.cochranelibrary.com/central/doi/10.1002/central/CN-02214967/full	Full text not available

Record Number	Full reference in JMIR/AMA style	PMID	DOI or URL	Reason for exclusion
5704	Chawla LS, Seneff MG, Nelson DR, et al. Elevated plasma concentrations of IL-6 and elevated APACHE II score predict acute kidney injury in patients with severe sepsis. Clinical journal of the American Society of Nephrology. 2007;2(1):22-30.	Not available	doi:10.2215/CJN.02510706	Full text not available
5721	Bokoch MP, Adelmann D, Kothari RP, et al. Feasibility and safety of the mild hypothermia and acute kidney injury in liver transplantation (MHALT) trial. Transplantation. 2019;103(8):71.	Not available	doi:10.1097/01.tp.0000580472.17422.db	Full text not available
5877	Nct. Prediction and Management of Acute Kidney Injury With Explainable Artificial Intelligence. https://clinicaltrials.gov/ct2/show/NCT05937451. 2023.	Not available	https://www.cochranelibrary.com/central/doi/10.1002/central/CN-02579248/full	Full text not available
5879	Pankratz DVS, Argyropoulos C, Myers O, Unruh ML. Prediction of Mortality or the Need for Continued Renal Replacement Therapy following Acute Kidney Injury Requiring. Journal of the American Society of Nephrology : JASN. 2016;27:257A.	Not available	https://www.cochranelibrary.com/central/doi/10.1002/central/CN-02596464/full	Full text not available
5925	Wong F, Boyer TD, Sanyal AJ, Pappas SC, Escalante S, Jamil K. Reduction in acute kidney injury stage predicts survival in patients with type-1 hepatorenal syndrome. Nephrology, dialysis, transplantation. 2020;35(9):1554-1561.	Not available	doi:10.1093/ndt/gfz048	Full text not available
5947	Ghani AA, Tohamy KY. Risk score for contrast induced nephropathy following percutaneous coronary intervention. Saudi journal of kidney diseases and transplantation. 2009;20(2):240-245.	Not available	https://www.cochranelibrary.com/central/doi/10.1002/central/CN-00684462/full	Full text not available
5948	Chang HC, Sung SH. Risk stratification in patients hospitalized for acute heart failure in asian population. European heart journal. 2018;39:1396-1397.	Not available	doi:10.1093/eurheartj/ehy566.P6543	Full text not available

5. Non-ML models (n=83)

Record Number	Full reference in JMIR/AMA style	PMID	DOI or URL	Reason for exclusion
52	Tao F, Yang H, Wang W, et al. Acute kidney injury prediction model utility in premature myocardial infarction. iScience. 2024;27(3):109153.	38390493	doi:10.1016/j.isci.2024.109153	Non-ML models
98	Wang J, Yu C, Zhang Y, Huang Y. A Prediction Model for Acute Kidney Injury After Pericardiectomy: An Observational Study. Front Cardiovasc Med. 2022;9:790044.	35224038	doi:10.3389/fcvm.2022.790044	Non-ML models
114	Wang S, Yang L, Zhou J, et al. A prediction model for acute kidney injury in adult patients with hemophagocytic lymphohistiocytosis. Front Immunol. 2022;13:987916.	36203572	doi:10.3389/fimmu.2022.987916	Non-ML models
138	Vagliano I, Lvova O, Schut MC. Interpretable and Continuous Prediction of Acute Kidney Injury in the Intensive Care. Stud Health Technol Inform. 2021;281:103-107.	34042714	doi:10.3233/shti210129	Non-ML models
205	Park N, Kang E, Park M, et al. Predicting acute kidney injury in cancer patients using heterogeneous and irregular data. PLoS One. 2018;13(7):e0199839.	30024918	doi:10.1371/journal.pone.0199839	Non-ML models
275	Peng M, Deng F, Qi D. Development of a nomogram model for the early prediction of sepsis-associated acute kidney injury in critically ill patients. Sci Rep. 2023;13(1):15200.	37709806	doi:10.1038/s41598-023-41965-x	Non-ML models
291	Davis SE, Lasko TA, Chen G, Siew ED, Matheny ME. Calibration drift in regression and machine learning models for acute kidney injury. J Am Med Inform Assoc. 2017;24(6):1052-1061.	28379439	doi:10.1093/jamia/ocx030	Non-ML models
294	Wu Y, Chen J, Luo C, Chen L, Huang B. Predicting the risk of postoperative acute kidney injury: development and assessment of a novel predictive nomogram. J Int Med Res. 2021;49(8):3000605211032838.	34382465	doi:10.1177/03000605211032838	Non-ML models
298	Zhou C, Wang R, Jiang W, et al. Machine learning for the prediction of acute kidney injury and paraplegia after thoracoabdominal aortic aneurysm repair. J Card Surg. 2020;35(1):89-99.	31765025	doi:10.1111/jocs.14317	Non-ML models
338	Wong WEJ, Chan SP, Yong JK, et al. Assessment of acute kidney injury risk using a machine-learning guided generalized structural equation model: a cohort study. BMC Nephrol. 2021;22(1):63.	33618695	doi:10.1186/s12882-021-02238-9	Non-ML models
374	Lv Y, Huang Z. Account of Deep Learning-Based Ultrasonic Image Feature in the Diagnosis of Severe Sepsis Complicated with Acute Kidney Injury. Comput Math Methods Med. 2022;2022:8158634.	35140807	doi:10.1155/2022/8158634	Non-ML models
382	Che M, Wang X, Liu S, et al. A Clinical Score to Predict Severe Acute Kidney Injury in Chinese Patients after Cardiac Surgery. Nephron. 2019;142(4):291-300.	30897580	doi:10.1159/000499345	Non-ML models
451	Jonsson AJ, Kristjansdottir I, Lund SH, Palsson R, Indridason OS. Computerized algorithms compared with a nephrologist's diagnosis of acute kidney injury in the emergency department. Eur J Intern Med. 2019;60:78-82.	30545589	doi:10.1016/j.ejim.2018.11.013	Non-ML models
453	Thottakkara P, Ozrazgat-Baslanti T, Hupf BB, et al. Application of Machine Learning Techniques to High-Dimensional Clinical Data to Forecast Postoperative Complications. PLoS One. 2016;11(5):e0155705.	27232332	doi:10.1371/journal.pone.0155705	Non-ML models
460	Koyner JL, Adhikari R, Edelson DP, Churpek MM. Development of a Multicenter Ward-Based AKI Prediction Model. Clin J Am Soc Nephrol. 2016;11(11):1935-1943.	27633727	doi:10.2215/cjn.00280116	Non-ML models
693	Barron J, Ng C, Aspin L, Robinson LJ, Smith G. Reflex testing to define action limits for community-based requests. Ann Clin Biochem. 2012;49(Pt 4):337-40.	22688736	doi:10.1258/acb.2012.011103	Non-ML models
699	Porter CJ, Juurlink I, Bisset LH, Bavakunji R, Mehta RL, Devonald MA. A real-time electronic alert to improve detection of acute kidney injury in a large teaching hospital. Nephrol Dial Transplant. 2014;29(10):1888-93.	24744280	doi:10.1093/ndt/gfu082	Non-ML models
751	Yu C, Guo D, Yao C, Zhu Y, Liu S, Kong X. Development and Validation of a Nomogram for Predicting Drug-Induced Acute Kidney Injury in Hospitalized Patients: A Case-Control Study Based on Propensity-Score Matching. Front Pharmacol. 2021;12:657853.	34194322	doi:10.3389/fphar.2021.657853	Non-ML models
820	Chen Z, Jia W, Guo C, Wu Y, Liu J, Song C. A Two-Center Study of a Prognostic Model Related to Acute Kidney Injury After Allogeneic Hematopoietic Stem Cell Transplantation in Children: Development of a New Predictive Dynamic Nomogram. Pediatr Blood Cancer. 2024;e31482.	39690795	doi:10.1002/pbc.31482	Non-ML models
921	Wu L, Hu Y, Yuan B, et al. Which risk predictors are more likely to indicate severe AKI in hospitalized patients?. Int J Med Inform. 2020;143:104270.	32961504	doi:10.1016/j.ijmedinf.2020.104270	Non-ML models
945	Lin H, Hou J, Tang H, et al. A novel nomogram to predict perioperative acute kidney injury following isolated coronary artery bypass grafting surgery with impaired left ventricular ejection fraction. BMC Cardiovasc Disord. 2020;20(1):517.	33302875	doi:10.1186/s12872-020-01799-1	Non-ML models
1484	Del Angel ES, Rivero Martínez JA. WCN24-2142 COMPUTATIONAL METHODOLOGY TO CHARACTERIZE AND IDENTIFY DAMAGE PATTERNS IN ACUTE KIDNEY INJURY PATIENTS. Kidney International Reports. 2024;9(4):S50-S51.	Not available	doi:10.1016/j.ekir.2024.02.1409	Non-ML models
2360	Huang CY, Güiza Grandas F, Schetz M, et al. Development and internal validation of a model to predict acute kidney injury recovery at hospital discharge. Critical Care. 2020;24.	Not available	doi:10.1186/s13054-020-2772-3	Non-ML models
2399	Huang CY, Guiza Grandas F, Schetz M, et al. Clinical prediction model for acute kidney injury recovery in the intensive care unit. Intensive Care Medicine Experimental. 2019;7.	Not available	doi:10.1186/s40635-019-0265-y	Non-ML models

Record Number	Full reference in JMIR/AMA style	PMID	DOI or URL	Reason for exclusion
2415	Abdul Muiz Dzihni W, Nor Azmi A, Ilyas C, Lotia S, Sivanandan I. Acute kidney injury in major abdominal nonelective surgery: A retrospective observational analysis. Anaesthesia. 2019;74:14.	Not available	doi:10.1111/anae.14740	Non-ML models
2439	Farmer C. Predicting acute kidney injury and clinical deterioration in hospital inpatients. European Journal of Clinical Investigation. 2019;49:40.	Not available	doi:10.1111/eci.13107	Non-ML models
2483	Falini S, Flechet M, Scharlaeken I, Güiza F, Van Den Berghe G, Meyfroidt G. Systolic blood pressure and lactate levels can predict severe pediatric AKI after cardiac surgery: A machine learning approach. Intensive Care Medicine Experimental. 2018;6.	Not available	doi:10.1186/s40635-018-0201-6	Non-ML models
2536	Scharlaeken I, Flechet M, Vlasselaers D, et al. A clinical prediction model for severe AKI after pediatric cardiac surgery. Critical Care. 2018;22.	Not available	doi:10.1186/s13054-018-1973-5	Non-ML models
2572	Not available. Predicting postoperative acute kidney injury. Colorectal Disease. 2017;19:73.	Not available	doi:10.1111/codi.13798	Non-ML models
2573	Marouli D, Papadakis E, Malliotakis P, et al. Preoperative albuminuria as a predictive marker of perioperative acute kidney injury (AKI) in non-cardiac major surgery. Intensive Care Medicine Experimental. 2017;5(2).	Not available	doi:10.1186/s40635-017-0151-4	Non-ML models
2575	Avila R, Carrizo N, Filippi M, Fernandez A, Reichert V. Acute kidney injury in critical trauma patients: Incidence and clinical outcomes. Intensive Care Medicine Experimental. 2017;5(2).	Not available	doi:10.1186/s40635-017-0151-4	Non-ML models
2576	Weisenthal S, Weisenthal SJ, Quill C, et al. A machine learning pipeline to predict acute kidney injury (AKI) in patients without AKI in their most recent hospitalization. Journal of Clinical and Translational Science. 2017;1:17-18.	Not available	doi:10.1017/cts.2017.287	Non-ML models
2661	Vanmassenhove J, Lameire N, Dhondt A, Vanholder R, Van Biesen W. Prognostic robustness of serum creatinine based AKI definitions in patients with sepsis: A prospective cohort study Epidemiology and Health Outcomes. BMC Nephrology. 2015;16(1).	Not available	doi:10.1186/s12882-015-0107-4	Non-ML models
4089	Sawhney S, Tan Z, Black C, et al. Validation of Risk Prediction Models to Inform Clinical Decisions After Acute Kidney Injury. AMERICAN JOURNAL OF KIDNEY DISEASES. 2021;78(1):28-37.	Not available	doi:10.1053/j.ajkd.2020.12.008	Non-ML models
4150	Lei L, He Y, Guo Z, et al. A Simple Nomogram to Predict Contrast-Induced Acute Kidney Injury in Patients with Congestive Heart Failure Undergoing Coronary Angiography. CARDIOLOGY RESEARCH AND PRACTICE. 2021;2021:9614953.	Not available	doi:10.1155/2021/9614953	Non-ML models
4380	Hsu CY, Chinchilli VM, Coca S, et al. Post-Acute Kidney Injury Proteinuria and Subsequent Kidney Disease Progression The Assessment, Serial Evaluation, and Subsequent Sequelae in Acute Kidney Injury (ASSESS-AKI) Study. JAMA INTERNAL MEDICINE. 2020;180(3):402-410.	Not available	doi:10.1001/jamainternmed.2019.6390	Non-ML models
4445	Tabata T, Sugiyama N, Otsuki Y, Kondo Y. Interleukin-24 is a novel diagnostic biomarker for the severity of acute kidney injury. MEDICAL MOLECULAR MORPHOLOGY. 2020;53(2):115-123.	Not available	doi:10.1007/s00795-019-00239-0	Non-ML models
4478	Ferrari F, Romero-Gonzalez G, Rizo Topete L, et al. Routine Adoption of Urinary [IGFBP7].[TIMP-2] to Assess Acute Kidney Injury at Any Stage 12 hours After Intensive Care Unit Admission: a Prospective Cohort Study. SCIENTIFIC REPORTS. 2019;9:16484.	Not available	doi:10.1038/s41598-019-52790-6	Non-ML models
4480	Regolisti G, Maggiore U, Greco P, et al. Electrocardiographic T wave alterations and prediction of hyperkalemia in patients with acute kidney injury. INTERNAL AND EMERGENCY MEDICINE. 2020;15(3):463-472.	Not available	doi:10.1007/s11739-019-02217-x	Non-ML models
4495	Jin J, Chang SC, Xu S, et al. Early Postoperative Serum Creatinine Adjusted for Fluid Balance Precisely Predicts Subsequent Acute Kidney Injury After Cardiac Surgery. JOURNAL OF CARDIOTHORACIC AND VASCULAR ANESTHESIA. 2019;33(10):2695-2702.	Not available	doi:10.1053/j.jvca.2019.03.023	Non-ML models
4561	Aiyegbusi O, Witham MD, Lim M, Gauld G, Bell S. Impact of introducing electronic acute kidney injury alerts in primary care. CLINICAL KIDNEY JOURNAL. 2019;12(2):253-257.	Not available	doi:10.1093/ckj/sfy083	Non-ML models
4574	Cole N, Selby NM, Prowle JR, et al. Risk prediction for acute kidney injury in acute medical admissions in the UK. QJM-AN INTERNATIONAL JOURNAL OF MEDICINE. 2019;112(3):197-205.	Not available	doi:10.1093/qjmed/hcy277	Non-ML models
4635	Weisenthal SJ, Quill C, Farooq S, Kautz H, Zand MS. Predicting acute kidney injury at hospital re-entry using high-dimensional electronic health record data. PLOS ONE. 2018;13(11):e0204920.	Not available	doi:10.1371/journal.pone.0204920	Non-ML models
4654	Beaubien-Souligny W, Benkreira A, Robillard P, et al. Alterations in Portal Vein Flow and Intrarenal Venous Flow Are Associated With Acute Kidney Injury After Cardiac Surgery: A Prospective Observational Cohort Study. JOURNAL OF THE AMERICAN HEART ASSOCIATION. 2018;7(19):e009961.	Not available	doi:10.1161/JAHA.118.009961	Non-ML models
4670	Motwani SS, Curhan GC. Risk Prediction Model for Cisplatin-Associated Acute Kidney Injury Reply. JOURNAL OF CLINICAL ONCOLOGY. 2018;36(23):2454-+.	Not available	doi:10.1200/JCO.2018.79.0832	Non-ML models
4735	Li Y, Yao L, Mao C, Srivastava A, Jiang X, Luo Y. Early Prediction of Acute Kidney Injury in Critical Care Setting Using Clinical Notes. 2018.	Not available	Not available	Non-ML models
4736	Burckhardt P, Nagin D, Vijayasarathy VPR, Padman R. Multi-Trajectory Modeling to Predict Acute Kidney Injury in Chronic Kidney Disease Patients. AMIA ... Annual Symposium proceedings. AMIA Symposium. 2018;2018:1196-1205.	30815162	Not available	Non-ML models
4795	Johnson M, Hounkpatin H, Fraser S, Culliford D, Uniacke M, Roderick P. Using a linked database for epidemiology across the primary and secondary care divide: acute kidney injury. BMC MEDICAL INFORMATICS AND DECISION MAKING. 2017;17:106.	Not available	doi:10.1186/s12911-017-0503-8	Non-ML models
4802	Schutz C, Boulware DR, Huppler-Hullsiek K, et al. Acute Kidney Injury and Urinary Biomarkers in Human Immunodeficiency Virus-Associated Cryptococcal Meningitis. OPEN FORUM INFECTIOUS DISEASES. 2017;4(3):ofx127.	Not available	doi:10.1093/ofid/ofx127	Non-ML models
4803	Duan C, Cao Y, Liu Y, et al. A New Preprocedure Risk Score for Predicting Contrast-Induced Acute Kidney Injury. CANADIAN JOURNAL OF CARDIOLOGY. 2017;33(6):714-723.	Not available	doi:10.1016/j.cjca.2017.01.015	Non-ML models

Record Number	Full reference in JMIR/AMA style	PMID	DOI or URL	Reason for exclusion
4808	Azzalini L, Garcia-Moll X. On Contrast-Induced Acute Kidney Injury, Risk Prediction, and the Future of Predictive Model Development. CANADIAN JOURNAL OF CARDIOLOGY. 2017;33(6):711-713.	Not available	doi:10.1016/j.cjca.2017.03.008	Non-ML models
4810	Leem AY, Park MS, Park BH, et al. Value of Serum Cystatin C Measurement in the Diagnosis of Sepsis-Induced Kidney Injury and Prediction of Renal Function Recovery. YONSEI MEDICAL JOURNAL. 2017;58(3):604-612.	Not available	doi:10.3349/ymj.2017.58.3.604	Non-ML models
4818	Kumar AB, Andrews W, Shi Y, et al. Fluid resuscitation mediates the association between inhalational burn injury and acute kidney injury in the major burn population. JOURNAL OF CRITICAL CARE. 2017;38:62-67.	Not available	doi:10.1016/j.jcrc.2016.10.008	Non-ML models
4834	Mita N, Kawahito S, Soga T, et al. Strict blood glucose control by an artificial endocrine pancreas during hepatectomy may prevent postoperative acute kidney injury. JOURNAL OF ARTIFICIAL ORGANS. 2017;20(1):76-83.	Not available	doi:10.1007/s10047-016-0925-6	Non-ML models
4849	Sun DQ, Zheng CF, Liu WY, et al. AKI-CLIF-SOFA: a novel prognostic score for critically ill cirrhotic patients with acute kidney injury. AGING-US. 2017;9(1):286-296.	Not available	doi:10.18632/aging.101161	Non-ML models
4860	Connell A, Montgomery H, Morris S, et al. Service evaluation of the implementation of a digitally-enabled care pathway for the recognition and management of acute kidney injury. F1000Research. 2017;6:1033-1033.	28751970	doi:10.12688/f1000research.11637.2	Non-ML models
4866	Wang Z, Ma S, Zappitelli M, Parikh C, Wang CY, Devarajan P. Penalized count data regression with application to hospital stay after pediatric cardiac surgery. STATISTICAL METHODS IN MEDICAL RESEARCH. 2016;25(6):2685-2703.	Not available	doi:10.1177/0962280214530608	Non-ML models
4880	van Wolfswinkel ME, Koopmans LC, Hesselink DA, et al. Neutrophil gelatinase-associated lipocalin (NGAL) predicts the occurrence of malaria-induced acute kidney injury. MALARIA JOURNAL. 2016;15:464.	Not available	doi:10.1186/s12936-016-1516-y	Non-ML models
4953	Basu RK, Gist K, Wheeler DS. Improving acute kidney injury diagnostics using predictive analytics. CURRENT OPINION IN CRITICAL CARE. 2015;21(6):473-478.	Not available	doi:10.1097/MCC.0000000000000257	Non-ML models
4977	Park MH, Shim HS, Kim WH, et al. Clinical Risk Scoring Models for Prediction of Acute Kidney Injury after Living Donor Liver Transplantation: A Retrospective Observational Study. PLOS ONE. 2015;10(8):e0136230.	Not available	doi:10.1371/journal.pone.0136230	Non-ML models
5010	Latus J, Schwab M, Tacconelli E, et al. Acute kidney injury and tools for risk-stratification in 456 patients with hantavirus-induced nephropathia epidemica. NEPHROLOGY DIALYSIS TRANSPLANTATION. 2015;30(2):245-251.	Not available	doi:10.1093/ndt/gfu319	Non-ML models
5031	Bang JY, Lee JB, Yoon Y, Seo HS, Song JG, Hwang GS. Acute kidney injury after infrarenal abdominal aortic aneurysm surgery: a comparison of AKIN and RIFLE criteria for risk prediction. BRITISH JOURNAL OF ANAESTHESIA. 2014;113(6):993-1000.	Not available	doi:10.1093/bja/aeu320	Non-ML models
5043	Wang X, Che M, Xie B, Xue S, Preoperative serum cystatin C combined with dipstick proteinuria predicts acute kidney injury after cardiac surgery YYAN. RENAL FAILURE. 2014. 36;10(1497-1503):10.3109/0886022X.2014.949759.	Not available	Not available	Non-ML models
5048	Najafi M. Serum creatinine role in predicting outcome after cardiac surgery beyond acute kidney injury. World journal of cardiology. 2014;6(9):1006-21.	25276301	doi:10.4330/wjc.v6.i9.1006	Non-ML models
5082	Wallace K, Mallard AS, Stratton JD, Johnstoh PA, Dickinson S, Parry RG. Use of an electronic alert to identify patients with acute kidney injury. CLINICAL MEDICINE. 2014;14(1):22-26.	Not available	doi:10.7861/clinmedicine.14-1-22	Non-ML models
5085	Jose Alcaraz A, Augusta Gil-Ruiz M, Castillo A, et al. Postoperative Neutrophil Gelatinase-Associated Lipocalin Predicts Acute Kidney Injury After Pediatric Cardiac Surgery. PEDIATRIC CRITICAL CARE MEDICINE. 2014;15(2):121-130.	Not available	doi:10.1097/PCC.0000000000000034	Non-ML models
5138	Bignami E, Casamassima N, Frati E, et al. Preoperative Endogenous Ouabain Predicts Acute Kidney Injury in Cardiac Surgery Patients. CRITICAL CARE MEDICINE. 2013;41(3):744-755.	Not available	doi:10.1097/CCM.0b013e3182741599	Non-ML models
5163	Zappitelli M, Coca SG, Garg AX, et al. The Association of Albumin/Creatinine Ratio with Postoperative AKI in Children Undergoing Cardiac Surgery. CLINICAL JOURNAL OF THE AMERICAN SOCIETY OF NEPHROLOGY. 2012;7(11):1761-1769.	Not available	doi:10.2215/CJN.12751211	Non-ML models
5192	Coca SG, Jammalamadaka D, Sint K, et al. Preoperative proteinuria predicts acute kidney injury in patients undergoing cardiac surgery. JOURNAL OF THORACIC AND CARDIOVASCULAR SURGERY. 2012;143(2):495-502.	Not available	doi:10.1016/j.jtcvs.2011.09.023	Non-ML models
5193	Ho J, Reslerova M, Gali B, et al. Serum Creatinine Measurement Immediately After Cardiac Surgery and Prediction of Acute Kidney Injury. AMERICAN JOURNAL OF KIDNEY DISEASES. 2012;59(2):196-201.	Not available	doi:10.1053/j.ajkd.2011.08.023	Non-ML models
5232	Celi LAG, Tang RJ, Villarroel MC, Davidzon GA, Lester WT, Chueh HC. A Clinical Database-Driven Approach to Decision Support: Predicting Mortality Among Patients with Acute Kidney Injury. JOURNAL OF HEALTHCARE ENGINEERING. 2011;2(1):97-109.	Not available	doi:10.1260/2040-2295.2.1.97	Non-ML models
5249	Matheny ME, Miller RA, Ikizler TA, et al. Development of Inpatient Risk Stratification Models of Acute Kidney Injury for Use in Electronic Health Records. MEDICAL DECISION MAKING. 2010;30(6):639-650.	Not available	doi:10.1177/0272989X10364246	Non-ML models
5250	Englberger L, Suri RM, Li Z, et al. Validation of Clinical Scores Predicting Severe Acute Kidney Injury After Cardiac Surgery. AMERICAN JOURNAL OF KIDNEY DISEASES. 2010;56(4):623-631.	Not available	doi:10.1053/j.ajkd.2010.04.017	Non-ML models
5263	Varghese SA, Powell TB, Janech MG, et al. Identification of Diagnostic Urinary Biomarkers for Acute Kidney Injury. JOURNAL OF INVESTIGATIVE MEDICINE. 2010;58(4):612-620.	Not available	Not available	Non-ML models

Record Number	Full reference in JMIR/AMA style	PMID	DOI or URL	Reason for exclusion
5271	Maccariello E, Valente C, Nogueira L, et al. SAPS 3 scores at the start of renal replacement therapy predict mortality in critically ill patients with acute kidney injury. KIDNEY INTERNATIONAL. 2010;77(1):51-56.	Not available	doi:10.1038/ki.2009.385	Non-ML models
5507	Matsuura R, Iwagami M, Moriya H, et al. A Simple Scoring Method for Predicting the Low Risk of Persistent Acute Kidney Injury in Critically Ill Adult Patients. Scientific reports. 2020;10(1):5726.	Not available	doi:10.1038/s41598-020-62479-w	Non-ML models
5641	Wan YP, Wang AJ, Zhang W, Zhang H, Peng GH, Zhu X. Development and validation of a nomogram for predicting overall survival in cirrhotic patients with acute kidney injury. World journal of gastroenterology. 2022;28(30):4133-4151.	Not available	doi:10.3748/wjg.v28.i30.4133	Non-ML models
5649	Liao T, Lu Y, Su T, et al. Development and validation of prognostic nomogram for cirrhotic patients with acute kidney injury upon ICU admission. Internal and emergency medicine. 2024;19(1):49-58.	Not available	doi:10.1007/s11739-023-03436-z	Non-ML models
5731	Hu J, Francesco Z, Edward AB, Chong L, Rezoagli E, Lorenzo B. Free hemoglobin ratio as a novel predictive biomarker of acute kidney injury after cardiac surgery: secondary analysis of a randomized controlled trial. Intensive care medicine experimental. 2020;8(SUPPL 2).	Not available	doi:10.1186/s40635-020-00354-8	Non-ML models
5884	Johansen KL, Smith MW, Unruh ML, Siroka AM, O'Connor TZ, Palevsky PM. Predictors of health utility among 60-day survivors of acute kidney injury in the Veterans Affairs/National Institutes of Health Acute Renal Failure Trial Network Study. Clinical journal of the American Society of Nephrology. 2010;5(8):1366-1372.	Not available	doi:10.2215/CJN.02570310	Non-ML models
5890	Khatua CR, Panigrahi S, Mishra D, et al. Presence of Acute kidney injury (AKI) at admission predicts early mortality in patients with acute on chronic liver failure (ACLF): a single centre experience. Hepatology international. 2018;12(2):S206-S207.	Not available	doi:10.1007/s12072-018-9852-3	Non-ML models
5901	Meneguz-Moreno RA, Ramos AI, Siqueira D, et al. Prognostic value of renal function in patients with aortic stenosis treated with transcatheter aortic valve replacement. Catheterization and cardiovascular interventions. 2017;89(3):452-459.	Not available	doi:10.1002/ccd.26693	Non-ML models
6010	Abosaif NY, Tolba YA, Heap M, Russell J, El Nahas AM. The outcome of acute renal failure in the intensive care unit according to RIFLE: model application, sensitivity, and predictability. American journal of kidney diseases. 2005;46(6):1038-1048.	Not available	doi:10.1053/j.ajkd.2005.08.033	Non-ML models

6. Outcome indicators non-compliant (n=128)

Record Number	Full reference in JMIR/AMA style	PMID	DOI or URL	Reason for exclusion
6	Yang YQ, Da JJ, Nie S, et al. A prediction model for moderate to severe acute kidney injury in people with heart failure. Mil Med Res. 2024;11(1):57.	39164767	doi:10.1186/s40779-024-00558-z	Outcome indicators non-compliant
135	Liang Q, Xu Y, Zhou Y, Chen X, Chen J, Huang M. Severe acute kidney injury predicting model based on transcontinental databases: a single-centre prospective study. BMJ Open. 2022;12(3):e054092.	35241466	doi:10.1136/bmjopen-2021-054092	Outcome indicators non-compliant
179	Lee BJ, Hsu CY, Parikh R, et al. Predicting Renal Recovery After Dialysis-Requiring Acute Kidney Injury. Kidney Int Rep. 2019;4(4):571-581.	30993232	doi:10.1016/j.ekir.2019.01.015	Outcome indicators non-compliant
198	Peng C, Yang F, Li L, et al. A Machine Learning Approach for the Prediction of Severe Acute Kidney Injury Following Traumatic Brain Injury. Neurocrit Care. 2023;38(2):335-344.	36195818	doi:10.1007/s12028-022-01606-z	Outcome indicators non-compliant
257	Jiang M, Pan CQ, Li J, Xu LG, Li CL. Explainable machine learning model for predicting furosemide responsiveness in patients with oliguric acute kidney injury. Ren Fail. 2023;45(1):2151468.	36645039	doi:10.1080/0886022x.2022.2151468	Outcome indicators non-compliant
266	Wang L, Peng F, Li ZH, et al. Identification of AKI signatures and classification patterns in ccRCC based on machine learning. Front Med (Lausanne). 2023;10:1195678.	37293297	doi:10.3389/fmed.2023.1195678	Outcome indicators non-compliant
279	He J, Lin J, Duan M. Application of Machine Learning to Predict Acute Kidney Disease in Patients With Sepsis Associated Acute Kidney Injury. Front Med (Lausanne). 2021;8:792974.	34957162	doi:10.3389/fmed.2021.792974	Outcome indicators non-compliant
288	Musiał K, Stojanowski J, Miśkiewicz-Bujna J, Kałwak K, Ussowicz M. KIM-1, IL-18, and NGAL, in the Machine Learning Prediction of Kidney Injury among Children Undergoing Hematopoietic Stem Cell Transplantation-A Pilot Study. Int J Mol Sci. 2023;24(21).	37958774	doi:10.3390/ijms242115791	Outcome indicators non-compliant
296	Feng Y, Li Q, Finfer S, et al. A Novel Risk Prediction Model for Severe Acute Kidney Injury in Intensive Care Unit Patients Receiving Fluid Resuscitation. Front Cardiovasc Med. 2022;9:840611.	35509279	doi:10.3389/fcvm.2022.840611	Outcome indicators non-compliant
312	Pannu N, Graham M, Klarenbach S, et al. A new model to predict acute kidney injury requiring renal replacement therapy after cardiac surgery. Cmaj. 2016;188(15):1076-1083.	27297813	doi:10.1503/cmaj.151447	Outcome indicators non-compliant
313	Mohamadlou H, Lynn-Palevsky A, Barton C, et al. Prediction of Acute Kidney Injury With a Machine Learning Algorithm Using Electronic Health Record Data. Can J Kidney Health Dis. 2018;5:2054358118776326.	30094049	doi:10.1177/2054358118776326	Outcome indicators non-compliant
315	Cox M, Panagides JC, Di Capua J, et al. An interpretable machine learning model for the prevention of contrast-induced nephropathy in patients undergoing lower extremity endovascular interventions for peripheral arterial disease. Clin Imaging. 2023;101:46029.	37247523	doi:10.1016/j.clinimag.2023.05.011	Outcome indicators non-compliant
320	Jiang X, Hu Y, Guo S, Du C, Cheng X. Prediction of persistent acute kidney injury in postoperative intensive care unit patients using integrated machine learning: a retrospective cohort study. Sci Rep. 2022;12(1):17134.	36224308	doi:10.1038/s41598-022-21428-5	Outcome indicators non-compliant
322	Yang D, Kang J, Li Y, et al. Development of a predictive nomogram for acute respiratory distress syndrome in patients with acute pancreatitis complicated with acute kidney injury. Ren Fail. 2023;45(2):2251591.	37724533	doi:10.1080/0886022x.2023.2251591	Outcome indicators non-compliant
328	Huang S, Teng Y, Du J, Zhou X, Duan F, Feng C. Internal and external validation of machine learning-assisted prediction models for mechanical ventilation-associated severe acute kidney injury. Aust Crit Care. 2023;36(4):604-612.	35842332	doi:10.1016/j.aucc.2022.06.001	Outcome indicators non-compliant
343	Mo M, Pan L, Huang Z, Liang Y, Liao Y, Xia N. Development and Validation of a Prediction Model for Survival in Diabetic Patients With Acute Kidney Injury. Front Endocrinol (Lausanne). 2021;12:737996.	35002952	doi:10.3389/fendo.2021.737996	Outcome indicators non-compliant
354	Persson I, Grünwald A, Morvan L, Becedas D, Arlbrandt M. A Machine Learning Algorithm Predicting Acute Kidney Injury in Intensive Care Unit Patients (NAVOY Acute Kidney Injury): Proof-of-Concept Study. JMIR Form Res. 2023;7:e45979.	38096015	doi:10.2196/45979	Outcome indicators non-compliant
360	Tsai PH, Wang JS, Shen CH. Validation of a Prediction Model for Acute Kidney Injury after Cardiac Surgery in a Retrospective Asian Cohort. J Clin Med. 2024;13(10).	38792279	doi:10.3390/jcm13102740	Outcome indicators non-compliant
364	Liu X, Fang M, Wang K, et al. Machine learning-based model to predict severe acute kidney injury after total aortic arch replacement for acute type A aortic dissection. Heliyon. 2024;10(13):e34171.	39071670	doi:10.1016/j.heliyon.2024.e34171	Outcome indicators non-compliant
373	Sun M, Baron J, Dighe A, et al. Early Prediction of Acute Kidney Injury in Critical Care Setting Using Clinical Notes and Structured Multivariate Physiological Measurements. Stud Health Technol Inform. 2019;264:368-372.	31437947	doi:10.3233/shti190245	Outcome indicators non-compliant
383	Du W, Wang X, Zhang D, Chen W, Zuo X, Li P. A genotype-guided prediction model for the incidence of persistent acute kidney injury following lung transplantation. BMC Nephrol. 2024;25(1):458.	39696008	doi:10.1186/s12882-024-03871-w	Outcome indicators non-compliant
385	Alfieri F, Ancona A, Tripepi G, et al. External validation of a deep-learning model to predict severe acute kidney injury based on urine output changes in critically ill patients. J Nephrol. 2022;35(8):2047-2056.	35554875	doi:10.1007/s40620-022-01335-8	Outcome indicators non-compliant
386	Wei W, Cai Z, Chen L, Yuan W, Fan Y, Rong S. Short-term prognostic models for severe acute kidney injury patients receiving prolonged intermittent renal replacement therapy based on machine learning. BMC Med Inform Decis Mak. 2023;23(1):133.	37488514	doi:10.1186/s12911-023-02231-2	Outcome indicators non-compliant
422	Litake O, Park BH, Tully JL, Gabriel RA. Constructing synthetic datasets with generative artificial intelligence to train large language models to classify acute renal failure from clinical notes. J Am Med Inform Assoc. 2024;31(6):1404-1410.	38622901	doi:10.1093/jamia/ocae081	Outcome indicators non-compliant

Record Number	Full reference in JMIR/AMA style	PMID	DOI or URL	Reason for exclusion
429	Zhao H, Liang L, Pan S, et al. Diabetes Mellitus as a Risk Factor for Progression from Acute Kidney Injury to Acute Kidney Disease: A Specific Prediction Model. Diabetes Metab Syndr Obes. 2021;14:2367-2379.	34079315	doi:10.2147/dmso.S307776	Outcome indicators non-compliant
442	Oh W, Takkavatakarn K, Kittrell H, et al. Development and Validation of a Policy Tree Approach for Optimizing Intravenous Fluids in Critically Ill Patients with Sepsis and Acute Kidney Injury. medRxiv. 2024.	39148835	doi:10.1101/2024.08.06.24311556	Outcome indicators non-compliant
452	Wang TH, Kao CC, Chang TH. Ensemble Machine Learning for Predicting 90-Day Outcomes and Analyzing Risk Factors in Acute Kidney Injury Requiring Dialysis. J Multidiscip Healthc. 2024;17:1589-1602.	38628614	doi:10.2147/jmdh.S448004	Outcome indicators non-compliant
521	Jia HM, Zheng Y, Huang LF, et al. Derivation and validation of plasma endostatin for predicting renal recovery from acute kidney injury: a prospective validation study. Crit Care. 2018;22(1):305.	30445971	doi:10.1186/s13054-018-2232-5	Outcome indicators non-compliant
527	Ying F, Chen S, Pan G, He Z. Artificial Intelligence Pulse Coupled Neural Network Algorithm in the Diagnosis and Treatment of Severe Sepsis Complicated with Acute Kidney Injury under Ultrasound Image. J Healthc Eng. 2021;2021:6761364.	34336164	doi:10.1155/2021/6761364	Outcome indicators non-compliant
541	Sun S, Ma F, Li Q, et al. Risk model for deaths and renal replacement therapy dependence in patients with acute kidney injury after cardiac surgery. Interact Cardiovasc Thorac Surg. 2017;25(4):548-554.	28655154	doi:10.1093/icvts/ivx210	Outcome indicators non-compliant
547	Jiang W, Zhang C, Yu J, Shao J, Zheng R. Development and validation of a nomogram for predicting in-hospital mortality of elderly patients with persistent sepsis-associated acute kidney injury in intensive care units: a retrospective cohort study using the MIMIC-IV database. BMJ Open. 2023;13(3):e069824.	36972970	doi:10.1136/bmjopen-2022-069824	Outcome indicators non-compliant
564	Flechet M, Falini S, Bonetti C, et al. Machine learning versus physicians' prediction of acute kidney injury in critically ill adults: a prospective evaluation of the AKIpredictor. Crit Care. 2019;23(1):282.	31420056	doi:10.1186/s13054-019-2563-x	Outcome indicators non-compliant
585	Wang YS, Chen DX, Yan M, et al. Prediction of the severity of acute kidney injury after on-pump cardiac surgery. J Clin Anesth. 2022;78:110664.	35086051	doi:10.1016/j.jclinane.2022.110664	Outcome indicators non-compliant
600	Slankamenac K, Beck-Schimmer B, Breitenstein S, Puhan MA, Clavien PA. Novel prediction score including pre- and intraoperative parameters best predicts acute kidney injury after liver surgery. World J Surg. 2013;37(11):2618-28.	23959337	doi:10.1007/s00268-013-2159-6	Outcome indicators non-compliant
607	McCallion S, McLarnon T, Cooper E, et al. Senescence Biomarkers CKAP4 and PTX3 Stratify Severe Kidney Disease Patients. Cells. 2024;13(19).	39404377	doi:10.3390/cells13191613	Outcome indicators non-compliant
617	Jia L, Bi X, Jia M, et al. [Risk factors analysis for severe acute kidney injury in septic patients and establishment and validation of an hour-specific prediction model]. Zhonghua Wei Zhong Bing Ji Jiu Yi Xue. 2024;36(9):910-916.	39380510	doi:10.3760/cma.j.cn121430-20240111-0003 8	Outcome indicators non-compliant
641	Alfieri F, Ancona A, Tripepi G, et al. A deep-learning model to continuously predict severe acute kidney injury based on urine output changes in critically ill patients. J Nephrol. 2021;34(6):1875-1886.	33900581	doi:10.1007/s40620-021-01046-6	Outcome indicators non-compliant
644	Wang X, Lin X, Xie B, et al. Early serum cystatin C-enhanced risk prediction for acute kidney injury post cardiac surgery: a prospective, observational, cohort study. Biomarkers. 2020;25(1):20-26.	31686541	doi:10.1080/1354750x.2019.1688865	Outcome indicators non-compliant
657	Wu L, Hu Y, Zhang X, et al. Changing relative risk of clinical factors for hospital-acquired acute kidney injury across age groups: a retrospective cohort study. BMC Nephrol. 2020;21(1):321.	32741377	doi:10.1186/s12882-020-01980-w	Outcome indicators non-compliant
662	Sun F, Wang H, Zhang D, Han F, Ye S. One-year renal outcome in lupus nephritis patients with acute kidney injury: a nomogram model. Rheumatology (Oxford). 2022;61(7):2886-2893.	34726692	doi:10.1093/rheumatology/keab818	Outcome indicators non-compliant
668	Liu L, Liu J, Lei L, et al. A prediction model of contrast-associated acute kidney injury in patients with hypoalbuminemia undergoing coronary angiography. BMC Cardiovasc Disord. 2020;20(1):399.	32867690	doi:10.1186/s12872-020-01689-6	Outcome indicators non-compliant
677	Kalisvaart M, Schlegel A, Umbro I, et al. The AKI Prediction Score: a new prediction model for acute kidney injury after liver transplantation. HPB (Oxford). 2019;21(12):1707-1717.	31153834	doi:10.1016/j.hpb.2019.04.008	Outcome indicators non-compliant
680	Luo M, Yang Y, Xu J, et al. A new scoring model for the prediction of mortality in patients with acute kidney injury. Sci Rep. 2017;7(1):7862.	28801674	doi:10.1038/s41598-017-08440-w	Outcome indicators non-compliant
683	Zhu S, Qiao W, Wang Y, et al. A contemporary simple risk score for prediction of severe acute kidney injury after heart transplantation. ESC Heart Fail. 2024.	39535931	doi:10.1002/ehf2.15108	Outcome indicators non-compliant
689	Bao P, Qiu P, Li T, et al. Prognostic value of preoperative nutritional status for postoperative moderate to severe acute kidney injury among older patients undergoing coronary artery bypass graft surgery: a retrospective study based on the MIMIC-IV database. Ren Fail. 2024;46(2):2429683.	39618077	doi:10.1080/0886022x.2024.2429683	Outcome indicators non-compliant
719	Luo CC, Zhong YL, Qiao ZY, et al. Development and validation of a nomogram for postoperative severe acute kidney injury in acute type A aortic dissection. J Geriatr Cardiol. 2022;19(10):734-742.	36338280	doi:10.11909/j.issn.1671-5411.2022.10.003	Outcome indicators non-compliant
722	Patidar KR, Xu C, Shamseddeen H, et al. Development and Validation of a Model to Predict Acute Kidney Injury in Hospitalized Patients With Cirrhosis. Clin Transl Gastroenterol. 2019;10(9):e00075.	31478958	doi:10.14309/ctg.0000000000000075	Outcome indicators non-compliant
791	Alfieri F, Ancona A, Tripepi G, et al. Continuous and early prediction of future moderate and severe Acute Kidney Injury in critically ill patients: Development and multi-centric, multi-national external validation of a machine-learning model. PLoS One. 2023;18(7):e0287398.	37490482	doi:10.1371/journal.pone.0287398	Outcome indicators non-compliant

Record Number	Full reference in JMIR/AMA style	PMID	DOI or URL	Reason for exclusion
802	Parikh CR, Coca SG, Thiessen-Philbrook H, et al. Postoperative biomarkers predict acute kidney injury and poor outcomes after adult cardiac surgery. J Am Soc Nephrol. 2011;22(9):1748-57.	21836143	doi:10.1681/asn.2010121302	Outcome indicators non-compliant
809	Echeverri J, Martins R, Harenski K, et al. Exploring the Cost-Utility of a Biomarker Predicting Persistent Severe Acute Kidney Injury: The Case of C-C Motif Chemokine Ligand 14 (CCL14). Clinicoecon Outcomes Res. 2024;16:46034.	38235419	doi:10.2147/ceor.S434971	Outcome indicators non-compliant
832	Demirjian S, Bashour CA, Shaw A, et al. Predictive Accuracy of a Perioperative Laboratory Test-Based Prediction Model for Moderate to Severe Acute Kidney Injury After Cardiac Surgery. Jama. 2022;327(10):956-964.	35258532	doi:10.1001/jama.2022.1751	Outcome indicators non-compliant
862	Song L, Li Y, Nie S, et al. Using machine learning to predict adverse events in acute coronary syndrome: A retrospective study. Clin Cardiol. 2023;46(12):1594-1602.	37654030	doi:10.1002/clc.24127	Outcome indicators non-compliant
879	Xu L, Wang X, Pu P, Li S, Shao Y, Li Y. Ultrasonic Image Features under the Intelligent Algorithm in the Diagnosis of Severe Sepsis Complicated with Renal Injury. Comput Math Methods Med. 2022;2022:2310014.	35991127	doi:10.1155/2022/2310014	Outcome indicators non-compliant
880	Yonekawa KE, Zhou C, Haaland WL, Wright DR. Nephrotoxin-Related Acute Kidney Injury and Predicting High-Risk Medication Combinations in the Hospitalized Child. J Hosp Med. 2019;14(8):462-467.	30986180	doi:10.12788/jhm.3196	Outcome indicators non-compliant
906	Husain-Syed F, Ferrari F, Sharma A, et al. Preoperative Renal Functional Reserve Predicts Risk of Acute Kidney Injury After Cardiac Operation. Ann Thorac Surg. 2018;105(4):1094-1101.	29382510	doi:10.1016/j.athoracsur.2017.12.034	Outcome indicators non-compliant
914	Koyner JL, Mackey RH, Rosenthal NA, et al. Health Care Resource Utilization and Costs of Persistent Severe Acute Kidney Injury (PS-AKI) Among Hospitalized Stage 2/3 AKI Patients. Kidney360. 2023;4(3):316-325.	36996299	doi:10.34067/kid.0005552022	Outcome indicators non-compliant
915	Luo X, Liu D, Li C, et al. The predictive value of the serum creatinine-to-albumin ratio (sCAR) and lactate dehydrogenase-to-albumin ratio (LAR) in sepsis-related persistent severe acute kidney injury. Eur J Med Res. 2025;30(1):25.	39800746	doi:10.1186/s40001-024-02269-6	Outcome indicators non-compliant
957	Koyner JL, Mackey RH, Rosenthal NA, et al. Clinical Outcomes of Persistent Severe Acute Kidney Injury among Patients with Kidney Disease Improving Global Outcomes Stage 2 or 3 Acute Kidney Injury. Am J Nephrol. 2022;53(46338):816-825.	36450235	doi:10.1159/000528158	Outcome indicators non-compliant
962	Ozrazgat-Baslanti T, Ren Y, Adiyeke E, et al. Development and validation of a race-agnostic computable phenotype for kidney health in adult hospitalized patients. PLoS One. 2024;19(4):e0299332.	38652731	doi:10.1371/journal.pone.0299332	Outcome indicators non-compliant
1005	Jiang W, Song L, Gong W, et al. Low HDL-C can be a biomarker to predict persistent severe AKI in septic patients? A retrospective cohort study. Eur J Med Res. 2023;28(1):567.	38053125	doi:10.1186/s40001-023-01513-9	Outcome indicators non-compliant
1036	Gupta S, Glezerman IG, Hirsch JS, et al. Derivation and external validation of a simple risk score for predicting severe acute kidney injury after intravenous cisplatin: cohort study. Bmj. 2024;384:e077169.	38538012	doi:10.1136/bmj-2023-077169	Outcome indicators non-compliant
1047	Chen Z, Pan L, Zhang J, et al. Development and validation of a nomogram for circuit lifespan of regional citrate anticoagulation-continuous renal replacement therapy in intensive care patients with acute kidney injury. Nurs Crit Care. 2024.	39511929	doi:10.1111/nicc.13196	Outcome indicators non-compliant
1137	Farmakis C. NOT YOUR AVERAGE FLU: A CASE OF SEVERE RHABDOMYOLYSIS AND KIDNEY FAILURE INDUCED BY INFLUENZA VIRUS. 2025;10:588-589.	Not available	doi:10.1016/j.ekir.2024.11.1052	Outcome indicators non-compliant
1138	Theodoropoulou E, Christofilidis G, Papadaki M, et al. SURPRISINGLY GOOD RESPONSE OF A SEVERE ANCA-NEGATIVE CRESCENTIC PAUCI-IMMUNE GLOMERULONEPHRITIS SUCCESSFULLY TREATED WITH CORTICOSTEROIDS, CYCLOPHOSPHAMIDE AND RITUXIMAB. 2025;10:36-37.	Not available	doi:10.1016/j.ekir.2024.11.135	Outcome indicators non-compliant
1154	Harsha vardhan KS, Yadla M, Kumar Bakka V, Burri S, Srinivas P. A STUDY OF ACUTE KIDNEY INJURY IN SEVERE ACUTE PANCREATITIS IN A TERTIARY CARE HOSPITAL. 2025;10:45-46.	Not available	doi:10.1016/j.ekir.2024.11.145	Outcome indicators non-compliant
1178	Benali D, mohammed B, imane M, intissar H, yassamine B. PREDICTIVE FOCTORS FOR THE USE OF HEMODIALYSIS DURING SEVERE ACUTE KIDNEY INJURY FOLLOWING PRE-ECLAMPSIA. 2025;10:604.	Not available	doi:10.1016/j.ekir.2024.11.1078	Outcome indicators non-compliant
1202	Nagarkar A, Sarvi RAVI. A RARE PRESENTATION OF ECTOPIC MEDIASTINAL PARATHYROID ADENOMA PRESENTING AS SEVERE HYPERCALCEMIA WITH ACUTE KIDNEY INJURY. 2025;10:684-685.	Not available	doi:10.1016/j.ekir.2024.11.1211	Outcome indicators non-compliant
1263	Onuigbo M, Breidenstein M, Onyia C, et al. Severe acute kidney injury with suspected bilateral renal artery stenosis on renal Duplex while on concurrent ACE inhibition – A case for carbon dioxide renal angiography. 2025;10:606-607.	Not available	doi:10.1016/j.ekir.2024.11.1081	Outcome indicators non-compliant
1294	Samatha T, Yadla M, Kumar V, B S, V S. CLINICAL CHARACTERISTICS AND OUTCOMES OF SEVERE ACUTE KIDNEY INJURY IN A TERTIARY CARE HOSPITAL- A TEN YEAR RETROSPECTIVE STUDY. 2025;10:607-609.	Not available	doi:10.1016/j.ekir.2024.11.1082	Outcome indicators non-compliant
1356	Chen Y, Jiang Y, Zhu X, et al. Construction and evaluation of dynamic nomogram model prediction model for early acute renal injury risk after heart transplantation. Chinese Journal of Clinical Pharmacology and Therapeutics. 2024;29(11):1272-1279.	Not available	doi:10.12092/j.issn.1009-2501.2024.11.009	Outcome indicators non-compliant
1485	Pimenta G, Marques RC, Sala I, et al. WCN24-2342 SEVERE ACUTE KIDNEY INJURY IN HOSPITALIZED CANCER PATIENTS: PREDICTING RENAL REPLACEMENT THERAPY AND MORTALITY. Kidney International Reports. 2024;9(4):S626.	Not available	doi:10.1016/j.ekir.2024.02.1317	Outcome indicators non-compliant
1488	Rivero Martínez JA, Sanntiago del Angel E. WCN24-1483 APPLYING ARTIFICIAL INTELLIGENCE TO IDENTIFY SUB-PHENOTYPES OF ACUTE KIDNEY INJURY IN MEXICAN PATIENTS WITH SEVERE COVID-19. Kidney International Reports. 2024;9(4):S49-S50.	Not available	doi:10.1016/j.ekir.2024.02.1408	Outcome indicators non-compliant

Record Number	Full reference in JMIR/AMA style	PMID	DOI or URL	Reason for exclusion
1553	Shekhar S, Sinha P, Singh WP. Evaluating the Prognostic Accuracy of Two Severity Scoring Systems in Acute Kidney Failure. International Journal of Pharmaceutical and Clinical Research. 2024;16(5):2803-2806.	Not available	https://www.embase.com/search/results?subaction=viewrecord&id=L2031992138&from=export	Outcome indicators non-compliant
1593	Koyner JL, Martin J, Carey K, et al. Multicenter Development and Validation of a Multimodal Deep Learning Model to Predict Severe AKI. Journal of the American Society of Nephrology. 2024;35:7.	Not available	doi:10.1681/asn.0000000000000259	Outcome indicators non-compliant
1595	Kashani K, Alfieri F, Ancona A, et al. Prospective and External Evaluation of an Artificial Intelligence Model for Continuous and Early Prediction of Moderate and Severe AKI in Critically Ill Patients. Journal of the American Society of Nephrology. 2024;35:58.	Not available	https://www.embase.com/search/results?subaction=viewrecord&id=L645709473&from=export	Outcome indicators non-compliant
1605	Yao J, Patel SK, Noel S, Cahan P, Rabb H. Single-Cell RNA Sequencing of Kidney-Gut Axis during Microbiome Manipulation of Severe AKI to CKD Progression. Journal of the American Society of Nephrology. 2024;35:862.	Not available	https://www.embase.com/search/results?subaction=viewrecord&id=L645710986&from=export	Outcome indicators non-compliant
1714	Anandakrishnan N, Yi Z, Liu T, et al. PREDICTION OF COVID-19 SEVERITY IN HOSPITALIZED PATIENTS USING URINARY PROTEOMICS AND MACHINE LEARNING. Nephrology Dialysis Transplantation. 2023;38:i101-i103.	Not available	doi:10.1093/ndt/gfad063a_3120	Outcome indicators non-compliant
1843	Menez S, Kerr KF, Cheng S, et al. Prediction of Postdischarge Kidney Disease Progression Among Patients with Hospitalized AKI: The ASSESS-AKI Study. Journal of the American Society of Nephrology. 2023;34:62.	Not available	https://www.embase.com/search/results?subaction=viewrecord&id=L642700814&from=export	Outcome indicators non-compliant
1846	Anandakrishnan N, Yi Z, Liu T, et al. Machine Learning-Based Multi-Omics Analyses Predict Disease Severity and Identify Molecular Mechanisms of COVID-Associated Kidney Injury. Journal of the American Society of Nephrology. 2023;34:65-66.	Not available	https://www.embase.com/search/results?subaction=viewrecord&id=L642701328&from=export	Outcome indicators non-compliant
2047	Chandler KE, Sweeney VH, Manganaro K, et al. Detecting and diagnosing severe maternal morbidity. American Journal of Obstetrics and Gynecology. 2022;226(1):S406.	Not available	doi:10.1016/j.ajog.2021.11.678	Outcome indicators non-compliant
2917	Sayan M, Altinisik HB, Sayan O. Neutrophil-platelet ratio as a predictor of acute kidney injury in severe COVID-19. MEDICINE. 2024;103(41):e40053.	Not available	doi:10.1097/MD.0000000000040053	Outcome indicators non-compliant
3318	Chen Y, Mo Z, Chu H, et al. A model for predicting postoperative persistent acute kidney injury (AKI) in AKI after cardiac surgery patients with normal baseline renal function. CLINICAL CARDIOLOGY. 2024;47(1).	Not available	doi:10.1002/clc.24168	Outcome indicators non-compliant
3363	Kusirisin P, Thanapongsatorn P, Chaikomon K, et al. The Role of Erythropoietin Levels in Predicting Long-Term Outcomes following Severe Acute Kidney Injury. BLOOD PURIFICATION. 2023;52(46275):793-801.	Not available	doi:10.1159/000531954	Outcome indicators non-compliant
3400	Peng JC, Wu Y, Xing SP, Zhu ML, Gao Y, Li W. Development and validation of a nomogram to predict the risk of renal replacement therapy among acute kidney injury patients in intensive care unit. CLINICAL AND EXPERIMENTAL NEPHROLOGY. 2023;27(11):951-960.	Not available	doi:10.1007/s10157-023-02383-5	Outcome indicators non-compliant
3509	Osuna-Padilla IA, Rodriguez-Moguel N, Lopez-Orduna LA, et al. Feeding intolerance during prolonged prone position in overweight and obese patients with severe COVID-19. NUTRICION HOSPITALARIA. 2023;40(2):250-256.	Not available	doi:10.20960/nh.04553	Outcome indicators non-compliant
3623	Koyner JL, Mackey RH, Rosenthal NA, et al. Clinical outcomes of persistent severe acute kidney injury among patients with KDIGO stage 2 or 3 AKI. AMERICAN JOURNAL OF NEPHROLOGY. 2023;53(46338):816-825.	Not available	doi:10.1159/000528158	Outcome indicators non-compliant
3675	Phulkerd T, Lertussavavivat T, Limothai U, et al. Circulating and urinary microRNAs profile for predicting renal recovery from severe acute kidney injury. JOURNAL OF INTENSIVE CARE. 2022;10(1):45.	Not available	doi:10.1186/s40560-022-00637-0	Outcome indicators non-compliant
3710	Xu L, Wang X, Pu P, Li S, Shao Y, Li Y. Ultrasonic Image Features under the Intelligent Algorithm in the Diagnosis of Severe Sepsis Complicated with Renal Injury (Retracted Article). COMPUTATIONAL AND MATHEMATICAL METHODS IN MEDICINE. 2022;2022:2310014.	Not available	doi:10.1155/2022/2310014	Outcome indicators non-compliant
3768	Huang T, He W, Xie Y, et al. A LASSO-derived clinical score to predict severe acute kidney injury in the cardiac surgery recovery unit: a large retrospective cohort study using the MIMIC database. BMJ OPEN. 2022;12(6):e060258.	Not available	doi:10.1136/bmjopen-2021-060258	Outcome indicators non-compliant
3811	Chen YW, Wu MY, Mao CH, et al. Severe acute kidney disease is associated with worse kidney outcome among acute kidney injury patients. SCIENTIFIC REPORTS. 2022;12(1):6492.	Not available	doi:10.1038/s41598-022-09599-7	Outcome indicators non-compliant
4008	Liu Y, Xiao J, Duan X, et al. The multivariable prognostic models for severe complications after heart valve surgery. BMC CARDIOVASCULAR DISORDERS. 2021;21(1):491.	Not available	doi:10.1186/s12872-021-02268-z	Outcome indicators non-compliant
4211	Liu LJ, Ortiz-Soriano V, Neyra JA, Chen J, Assoc Comp M. KGDAL: Knowledge Graph Guided Double Attention LSTM for Rolling Mortality Prediction for AKI-D Patients. 2021.	Not available	doi:10.1145/3459930.3469513	Outcome indicators non-compliant
4326	Wang W, Li Z, Chen Y, Wu H, Zhang S, Chen X. Prediction Value of Serum NGAL in the Diagnosis and Prognosis of Experimental Acute and Chronic Kidney Injuries. BIOMOLECULES. 2020;10(7):981.	Not available	doi:10.3390/biom10070981	Outcome indicators non-compliant
4348	Xu L, Li C, Zhao L, et al. Acute kidney injury after nephrectomy: a new nomogram to predict postoperative renal function. BMC NEPHROLOGY. 2020;21(1):181.	Not available	doi:10.1186/s12882-020-01839-0	Outcome indicators non-compliant
4362	Zhang X, Liu B, Liu Y, Ma L, Zeng H. Efficacy of the quick sequential organ failure assessment for predicting clinical outcomes among community-acquired pneumonia patients presenting in the emergency department. BMC INFECTIOUS DISEASES. 2020;20(1):316.	Not available	doi:10.1186/s12879-020-05044-0	Outcome indicators non-compliant

Record Number	Full reference in JMIR/AMA style	PMID	DOI or URL	Reason for exclusion
4391	Nishtala PS, Chyou TY. Identifying drug combinations associated with acute kidney injury using association rules method. PHARMACOEPIDEMIOLOGY AND DRUG SAFETY. 2020;29(4):467-473.	Not available	doi:10.1002/pds.4960	Outcome indicators non-compliant
4397	Naruse H, Ishii J, Takahashi H, et al. Urinary Liver-Type Fatty-Acid-Binding Protein Predicts Long-Term Adverse Outcomes in Medical Cardiac Intensive Care Units. JOURNAL OF CLINICAL MEDICINE. 2020;9(2):482.	Not available	doi:10.3390/jcm9020482	Outcome indicators non-compliant
4398	Wolff G, Lin Y, Quade J, et al. Validation of National Cardiovascular Data Registry risk models for mortality, bleeding and acute kidney injury in interventional cardiology at a German Heart Center. CLINICAL RESEARCH IN CARDIOLOGY. 2020;109(2):235-245.	Not available	doi:10.1007/s00392-019-01506-x	Outcome indicators non-compliant
4438	Howitt SH, Oakley J, Caiado C, et al. A Novel Patient-Specific Model for Predicting Severe Oliguria; Development and Comparison With Kidney Disease: Improving Global Outcomes Acute Kidney Injury Classification. CRITICAL CARE MEDICINE. 2020;48(1):E18-E25.	Not available	doi:10.1097/CCM.0000000000004074	Outcome indicators non-compliant
4440	Nguyen M, Hasan SM, Quach S, et al. Comparison of the Performance of the NCDR CathPCI Score and Mehran Score to Predict Acute Kidney Injury Post-Percutaneous Coronary Intervention. HCA healthcare journal of medicine. 2020;1(5):305-314.	37426612	doi:10.36518/2689-0216.1110	Outcome indicators non-compliant
4448	Lei VJ, ThaiBinh L, Shan E, et al. Risk Stratification for Postoperative Acute Kidney Injury in Major Noncardiac Surgery Using Preoperative and Intraoperative Data. JAMA NETWORK OPEN. 2019;2(12):e1916921.	Not available	doi:10.1001/jamanetworkopen.2019.16921	Outcome indicators non-compliant
4454	Cosentino N, Genovese S, Campodonico J, et al. High-Sensitivity C-Reactive Protein and Acute Kidney Injury in Patients with Acute Myocardial Infarction: A Prospective Observational Study. JOURNAL OF CLINICAL MEDICINE. 2019;8(12):2192.	Not available	doi:10.3390/jcm8122192	Outcome indicators non-compliant
4493	Fiorentino M, Tohme FA, Murugan R, Kellum JA. Plasma Biomarkers in Predicting Renal Recovery from Acute Kidney Injury in Critically Ill Patients. BLOOD PURIFICATION. 2019;48(3):253-261.	Not available	doi:10.1159/000500423	Outcome indicators non-compliant
4587	Low S, Vathsala A, Murali TM, et al. Electronic health records accurately predict renal replacement therapy in acute kidney injury. BMC NEPHROLOGY. 2019;20:32.	Not available	doi:10.1186/s12882-019-1206-4	Outcome indicators non-compliant
4609	McMahon BA, Galligan M, Redahan L, et al. Biomarker Predictors of Adverse Acute Kidney Injury Outcomes in Critically Ill Patients: The Dublin Acute Biomarker Group Evaluation Study. AMERICAN JOURNAL OF NEPHROLOGY. 2019;50(1):19-28.	Not available	doi:10.1159/000500231	Outcome indicators non-compliant
4624	Martini A, Cumarasamy S, Beksac AT, et al. A Nomogram to Predict Significant Estimated Glomerular Filtration Rate Reduction After Robotic Partial Nephrectomy. EUROPEAN UROLOGY. 2018;74(6):833-839.	Not available	doi:10.1016/j.eururo.2018.08.037	Outcome indicators non-compliant
4625	Ranucci M, Aloisio T, Cazzaniga A, Di Dedda U, Gallazzi C, Pistuddi V. Validation of renal-risk models for the prediction of non-renal replacement therapy cardiac surgery-associated acute kidney injury. INTERNATIONAL JOURNAL OF CARDIOLOGY. 2018;272:49-53.	Not available	doi:10.1016/j.ijcard.2018.07.114	Outcome indicators non-compliant
4658	Hinson JS, Martinez DA, Grams MS, Levin S. Prediction of Acute Kidney Injury in the Emergency Department Using Electronic Health Record Data and Machine Learning Methods. ANNALS OF EMERGENCY MEDICINE. 2018;72(4):S154-S154.	Not available	doi:10.1016/j.annemergmed.2018.08.398	Outcome indicators non-compliant
4760	James MT, Pannu N, Hemmelgarn BR, et al. Derivation and External Validation of Prediction Models for Advanced Chronic Kidney Disease Following Acute Kidney Injury. JAMA-JOURNAL OF THE AMERICAN MEDICAL ASSOCIATION. 2017;318(18):1787-1797.	Not available	doi:10.1001/jama.2017.16326	Outcome indicators non-compliant
4771	Mikacenic C, Price BL, Harju-Baker S, et al. A Two Biomarker Model Predicts Mortality in the Critically Ill with Sepsis. AMERICAN JOURNAL OF RESPIRATORY AND CRITICAL CARE MEDICINE. 2017;196(8).	Not available	doi:10.1164/rccm.201611-2307OC	Outcome indicators non-compliant
4801	Pereira M, Rodrigues N, Godinho I, et al. Acute kidney injury in patients with severe sepsis or septic shock: a comparison between the 'Risk, Injury, Failure, Loss of kidney function, End-stage kidney disease' (RIFLE), Acute Kidney Injury Network (AKIN) and Kidney Disease: Improving Global Outcomes (KDIGO) classifications. CLINICAL KIDNEY JOURNAL. 2017;10(3):332-340.	Not available	doi:10.1093/ckj/sfw107	Outcome indicators non-compliant
4831	Hu X, Zhuang XD, Li Y, et al. A Nomogram to Predict Contrast Induced Nephropathy in Patients Undergoing Percutaneous Coronary Intervention. INTERNATIONAL HEART JOURNAL. 2017;58(2):191-196.	Not available	doi:10.1536/ihj.16-213	Outcome indicators non-compliant
4837	Smith LE, Smith DK, Blume JD, Siew ED, Billings FT. Latent variable modeling improves AKI risk factor identification and AKI prediction compared to traditional methods. BMC NEPHROLOGY. 2017;18:55.	Not available	doi:10.1186/s12882-017-0465-1	Outcome indicators non-compliant
4856	Jia Q, Wang YR, He P, et al. Prediction model of in-hospital mortality in elderly patients with acute heart failure based on retrospective study. JOURNAL OF GERIATRIC CARDIOLOGY. 2017;14(11):669-678.	Not available	doi:10.11909/j.issn.1671-5411.2017.11.002	Outcome indicators non-compliant
4865	Chen J, Zhang G, Wang C, et al. Predicting renal replacement therapy after cardiac valve surgery: external validation and comparison of two clinical scores. INTERACTIVE CARDIOVASCULAR AND THORACIC SURGERY. 2016;23(6):869-875.	Not available	doi:10.1093/icvts/ivw271	Outcome indicators non-compliant
4895	de Melo Bezerra Cavalcante CT, Castelo Branco KM, Pinto Junior VC, et al. Syndecan-1 improves severe acute kidney injury prediction after pediatric cardiac surgery. JOURNAL OF THORACIC AND CARDIOVASCULAR SURGERY. 2016;152(1):178-+.	Not available	doi:10.1016/j.jtcvs.2016.03.079	Outcome indicators non-compliant
4959	Rewa O, Wald R, Adhikari NKJ, et al. Whole-blood neutrophil gelatinase-associated lipocalin to predict adverse events in acute kidney injury: A prospective observational cohort study. JOURNAL OF CRITICAL CARE. 2015;30(6):1359-1364.	Not available	doi:10.1016/j.jcrc.2015.08.019	Outcome indicators non-compliant
4976	Kristovic D, Horvatic I, Husedzinovic I, et al. Cardiac surgery-associated acute kidney injury: risk factors analysis and comparison of prediction models. INTERACTIVE CARDIOVASCULAR AND THORACIC SURGERY. 2015;21(3):366-373.	Not available	doi:10.1093/icvts/ivv162	Outcome indicators non-compliant
5072	Cruz DN, Ferrer-Nadal A, Piccinni P, et al. Utilization of Small Changes in Serum Creatinine with Clinical Risk Factors to Assess the Risk of AKI in Critically Ill Adults. CLINICAL JOURNAL OF THE AMERICAN SOCIETY OF NEPHROLOGY. 2014;9(4):663-672.	Not available	doi:10.2215/CJN.05190513	Outcome indicators non-compliant

Record Number	Full reference in JMIR/AMA style	PMID	DOI or URL	Reason for exclusion
5077	Malov AA, Borisov AS, Lomivorotov VV, et al. Mortality Prediction in Patients with Dialysis-dependent Acute Kidney Injury after Cardiac Surgery with Cardiopulmonary Bypass. HEART LUNG AND CIRCULATION. 2014;23(4):325-331.	Not available	doi:10.1016/j.hlc.2013.10.082	Outcome indicators non-compliant
5090	Birnie K, Verheyden V, Pagano D, et al. Predictive models for kidney disease: improving global outcomes (KDIGO) defined acute kidney injury in UK cardiac surgery. CRITICAL CARE. 2014;18(6):606.	Not available	doi:10.1186/s13054-014-0606-x	Outcome indicators non-compliant
5099	Alge JL, Karakala N, Neely BA, et al. Association of Elevated Urinary Concentration of Renin-Angiotensin System Components and Severe AKI. CLINICAL JOURNAL OF THE AMERICAN SOCIETY OF NEPHROLOGY. 2013;8(12):2043-2052.	Not available	doi:10.2215/CJN.03510413	Outcome indicators non-compliant
5139	Kiers HD, van den Boogaard M, Schoenmakers MCJ, et al. Comparison and clinical suitability of eight prediction models for cardiac surgery-related acute kidney injury. NEPHROLOGY DIALYSIS TRANSPLANTATION. 2013;28(2):345-351.	Not available	doi:10.1093/ndt/gfs518	Outcome indicators non-compliant
5187	Demirjian S, Schold JD, Navia J, et al. Predictive Models for Acute Kidney Injury Following Cardiac Surgery. AMERICAN JOURNAL OF KIDNEY DISEASES. 2012;59(3):382-389.	Not available	doi:10.1053/j.ajkd.2011.10.046	Outcome indicators non-compliant
5194	Wu VC, Huang TM, Wu PC, et al. Preoperative Proteinuria Is Associated with Long-Term Progression to Chronic Dialysis and Mortality after Coronary Artery Bypass Grafting Surgery. PLOS ONE. 2012;7(1):e27687.	Not available	doi:10.1371/journal.pone.0027687	Outcome indicators non-compliant
5217	Chawla LS, Amdur RL, Amodeo S, Kimmel PL, Palant CE. The severity of acute kidney injury predicts progression to chronic kidney disease. KIDNEY INTERNATIONAL. 2011;79(12):1361-1369.	Not available	doi:10.1038/ki.2011.42	Outcome indicators non-compliant
5586	Phulkerd T, Limothai U, Pisitkun T, et al. Circulating and urinary microtranscriptome reveal micrornas profile for predicting renal recovery from severe acute kidney injury. Nephrology (Carlton, Vic.). 2021;26(SUPPL 1):5.	Not available	doi:10.1111/nep.13943	Outcome indicators non-compliant
5592	Yan W, Sun XY, Wang M, Zhao FF, Zhou QT. Clinical Evaluation of a Vancomycin Dosage Strategy Based on a Serum Trough Concentration Model in Elderly Patients with Severe Pneumonia. Biomedical and environmental sciences. 2023;36(5):397-405.	Not available	doi:10.3967/bes2023.049	Outcome indicators non-compliant

7. Non-predictive model studies (n=35)

Record Number	Full reference in JMIR/AMA style	PMID	DOI or URL	Reason for exclusion
816	Hu Q, Shi Y, Hua ZY, et al. A Prediction Nomogram for Acute Kidney Injury in Very-Low-Birth-Weight Infants: A Retrospective Study. Front Pediatr. 2020;8:575097.	33520885	doi:10.3389/fped.2020.575097	Non-predictive model studies
838	Mårtensson J, Vaara ST, Pettilä V, et al. Assessment of plasma endostatin to predict acute kidney injury in critically ill patients. Acta Anaesthesiol Scand. 2017;61(10):1286-1295.	28857121	doi:10.1111/aas.12988	Non-predictive model studies
839	Brown JR, MacKenzie TA, Maddox TM, et al. Acute Kidney Injury Risk Prediction in Patients Undergoing Coronary Angiography in a National Veterans Health Administration Cohort With External Validation. J Am Heart Assoc. 2015;4(12).	26656858	doi:10.1161/jaha.115.002136	Non-predictive model studies
963	Churpek MM, Carey KA, Edelson DP, et al. Internal and External Validation of a Machine Learning Risk Score for Acute Kidney Injury. JAMA Netw Open. 2020;3(8):e2012892.	32780123	doi:10.1001/jamanetworkopen.2020.12892	Non-predictive model studies
980	Jarai R, Dangas G, Huber K, et al. B-type natriuretic peptide and risk of contrast-induced acute kidney injury in acute ST-segment-elevation myocardial infarction: a substudy from the HORIZONS-AMI trial. Circ Cardiovasc Interv. 2012;5(6):813-20.	23192919	doi:10.1161/circinterventions.112.972356	Non-predictive model studies
1091	Vanmassenhove J, Lameire N, Dhondt A, Vanholder R, Van Biesen W. Prognostic robustness of serum creatinine based AKI definitions in patients with sepsis: a prospective cohort study. BMC Nephrol. 2015;16:112.	26199072	doi:10.1186/s12882-015-0107-4	Non-predictive model studies
4308	Oshita T, Hiraoka A, Nakajima K, et al. A Better Predictor of Acute Kidney Injury After Cardiac Surgery: The Largest Area Under the Curve Below the Oxygen Delivery Threshold During Cardiopulmonary Bypass. JOURNAL OF THE AMERICAN HEART ASSOCIATION. 2020;9(15):e015566.	Not available	doi:10.1161/JAHA.119.015566	Non-predictive model studies
4434	Deng Y, Ma J, Hou Y, et al. Combining Serum Cystatin C and Urinary N-Acetyl-Beta-D-Glucosaminidase Improves the Precision for Acute Kidney Injury Diagnosis after Resection of Intracranial Space-Occupying Lesions. KIDNEY & BLOOD PRESSURE RESEARCH. 2020;45(1):142-156.	Not available	doi:10.1159/000504599	Non-predictive model studies
4443	Dedemoglu M, Tuysuz ME. Risk estimation model for acute kidney injury defined by KDIGO classification after heart valve replacement surgery. GENERAL THORACIC AND CARDIOVASCULAR SURGERY. 2020;68(9):922-931.	Not available	doi:10.1007/s11748-019-01278-1	Non-predictive model studies
4447	Flechet M, Guiza F, Scharlaeken I, et al. Near-Infrared-Based Cerebral Oximetry for Prediction of Severe Acute Kidney Injury in Critically Ill Children After Cardiac Surgery. Critical care explorations. 2019;1(12):e0063-e0063.	32166244	doi:10.1097/CCE.0000000000000063	Non-predictive model studies
4458	Guan C, Li C, Xu L, et al. Risk factors of cardiac surgery-associated acute kidney injury: development and validation of a perioperative predictive nomogram. JOURNAL OF NEPHROLOGY. 2019;32(6):937-945.	Not available	doi:10.1007/s40620-019-00624-z	Non-predictive model studies
4463	Seker YT, Cukurova Z, Bilgi DO, Hergunsel O. Prognostic Impact of Early Versus Late Initiation of Renal Replacement Therapy Based on Early Warning Algorithm in Critical Care Patients With Acute Kidney Injury. THERAPEUTIC APHERESIS AND DIALYSIS. 2020;24(4):445-452.	Not available	doi:10.1111/1744-9987.13449	Non-predictive model studies
4465	Yeh HC, Ting IW, Huang HC, Chiang HY, Kuo CC. Acute Kidney Injury in the Outpatient Setting Associates with Risk of End-Stage Renal Disease and Death in Patients with CKD. SCIENTIFIC REPORTS. 2019;9:17658.	Not available	doi:10.1038/s41598-019-54227-6	Non-predictive model studies
4472	McBride WT, Kurth MJ, McLean G, et al. Stratifying risk of acute kidney injury in pre and post cardiac surgery patients using a novel biomarker-based algorithm and clinical risk score. SCIENTIFIC REPORTS. 2019;9:16963.	Not available	doi:10.1038/s41598-019-53349-1	Non-predictive model studies
4516	Neyra JA, Hu MC, Minhajuddin A, et al. Kidney Tubular Damage and Functional Biomarkers in Acute Kidney Injury Following Cardiac Surgery. KIDNEY INTERNATIONAL REPORTS. 2019;4(8):1131-1142.	Not available	doi:10.1016/j.ekir.2019.05.005	Non-predictive model studies
4531	Huelin P, Sola E, Elia C, et al. Neutrophil Gelatinase-Associated Lipocalin for Assessment of Acute Kidney Injury in Cirrhosis: A Prospective Study. HEPATOLOGY. 2019;70(1):319-333.	Not available	doi:10.1002/hep.30592	Non-predictive model studies
4536	Martini A, Sfakianos JP, Paulucci DJ, et al. Predicting acute kidney injury after robot-assisted partial nephrectomy: Implications for patient selection and postoperative management. UROLOGIC ONCOLOGY-SEMINARS AND ORIGINAL INVESTIGATIONS. 2019;37(7):445-451.	Not available	doi:10.1016/j.urolonc.2019.04.018	Non-predictive model studies
4621	Harrois A, Soyer B, Gauss T, et al. Prevalence and risk factors for acute kidney injury among trauma patients: a multicenter cohort study. CRITICAL CARE. 2018;22:344.	Not available	doi:10.1186/s13054-018-2265-9	Non-predictive model studies
4660	Lee CC, Chang CH, Chen SW, et al. Preoperative risk assessment improves biomarker detection for predicting acute kidney injury after cardiac surgery. PLOS ONE. 2018;13(9):e0203447.	Not available	doi:10.1371/journal.pone.0203447	Non-predictive model studies
4676	Wu VC, Shiao CC, Chi NH, et al. Outcome Prediction of Acute Kidney Injury Biomarkers at Initiation of Dialysis in Critical Units. JOURNAL OF CLINICAL MEDICINE. 2018;7(8):202.	Not available	doi:10.3390/jcm7080202	Non-predictive model studies
4794	Zambetti BR, Thomas F, Hwang I, et al. A web-based tool to predict acute kidney injury in patients with ST-elevation myocardial infarction: Development, internal validation and comparison. PLOS ONE. 2017;12(7):e0181658.	Not available	doi:10.1371/journal.pone.0181658	Non-predictive model studies
4799	Hunsicker O, Feldheiser A, Weimann A, et al. Diagnostic value of plasma NGAL and intraoperative diuresis for AKI after major gynecological surgery in patients treated within an intraoperative goal-directed hemodynamic algorithm A substudy of a randomized controlled trial. MEDICINE. 2017;96(28):e7357.	Not available	doi:10.1097/MD.0000000000007357	Non-predictive model studies
4830	Crosina J, Lerner J, Ho J, et al. Improving the Prediction of Cardiac Surgery-Associated Acute Kidney Injury. KIDNEY INTERNATIONAL REPORTS. 2017;2(2):172-179.	Not available	doi:10.1016/j.ekir.2016.10.003	Non-predictive model studies
4841	Wang W, Pan Y, Tang X, et al. Serum prealbumin and its changes over time are associated with mortality in acute kidney injury. SCIENTIFIC REPORTS. 2017;7:41493.	Not available	doi:10.1038/srep41493	Non-predictive model studies

Record Number	Full reference in JMIR/AMA style	PMID	DOI or URL	Reason for exclusion
4850	Jiang W, Xu J, Shen B, Wang C, Teng J, Ding X. Validation of Four Prediction Scores for Cardiac Surgery-Associated Acute Kidney Injury in Chinese Patients. BRAZILIAN JOURNAL OF CARDIOVASCULAR SURGERY. 2017;32(6):481-486.	Not available	doi:10.21470/1678-9741-2017-0116	Non-predictive model studies
4903	Metzger J, Mullen W, Husi H, et al. Acute kidney injury prediction in cardiac surgery patients by a urinary peptide pattern: a case-control validation study. CRITICAL CARE. 2016;20:157.	Not available	doi:10.1186/s13054-016-1344-z	Non-predictive model studies
4995	Kane-Gill SL, Sileanu FE, Murugan R, Trietley GS, Handler SM, Kellum JA. Risk Factors for Acute Kidney Injury in Older Adults With Critical Illness: A Retrospective Cohort Study. AMERICAN JOURNAL OF KIDNEY DISEASES. 2015;65(6):860-869.	Not available	doi:10.1053/j.ajkd.2014.10.018	Non-predictive model studies
5033	Shacham Y, Leshem-Rubinow E, Ziv-Baran T, et al. Incidence and mortality of acute kidney injury in acute myocardial infarction patients: a comparison between AKIN and RIFLE criteria. INTERNATIONAL UROLOGY AND NEPHROLOGY. 2014;46(12):2371-2377.	Not available	doi:10.1007/s11255-014-0827-6	Non-predictive model studies
5044	Cho E, Kim SC, Kim MG, Jo SK, Cho WY, Kim HK. The incidence and risk factors of acute kidney injury after hepatobiliary surgery: a prospective observational study. BMC NEPHROLOGY. 2014;15:169.	Not available	doi:10.1186/1471-2369-15-169	Non-predictive model studies
5050	Simonini M, Lanzani C, Bignami E, et al. A new clinical multivariable model that predicts postoperative acute kidney injury: impact of endogenous ouabain. NEPHROLOGY DIALYSIS TRANSPLANTATION. 2014;29(9):1696-1701.	Not available	doi:10.1093/ndt/gfu200	Non-predictive model studies
5188	Breidthardt T, Christ-Crain M, Stolz D, et al. A Combined Cardiorenal Assessment for the Prediction of Acute Kidney Injury in Lower Respiratory Tract Infections. AMERICAN JOURNAL OF MEDICINE. 2012;125(2):168-175.	Not available	doi:10.1016/j.amjmed.2011.07.010	Non-predictive model studies
5207	Krawczeski CD, Goldstein SL, Woo JG, et al. Temporal Relationship and Predictive Value of Urinary Acute Kidney Injury Biomarkers After Pediatric Cardiopulmonary Bypass. JOURNAL OF THE AMERICAN COLLEGE OF CARDIOLOGY. 2011;58(22):2301-2309.	Not available	doi:10.1016/j.jacc.2011.08.017	Non-predictive model studies
5211	Parikh CR, Devarajan P, Zappitelli M, et al. Postoperative Biomarkers Predict Acute Kidney Injury and Poor Outcomes after Pediatric Cardiac Surgery. JOURNAL OF THE AMERICAN SOCIETY OF NEPHROLOGY. 2011;22(9):1737-1747.	Not available	doi:10.1681/ASN.2010111163	Non-predictive model studies
5242	Kim MY, Jang HR, Huh W, et al. Incidence, Risk Factors, and Prediction of Acute Kidney Injury After Off-Pump Coronary Artery Bypass Grafting. RENAL FAILURE. 2011;33(3):316-322.	Not available	doi:10.3109/0886022X.2011.560406	Non-predictive model studies
5820	Demirjian S, Chertow GM, Zhang JH, et al. Model to predict mortality in critically ill adults with acute kidney injury. Clinical journal of the American Society of Nephrology. 2011;6(9):2114-2120.	Not available	doi:10.2215/CJN.02900311	Non-predictive model studies

8. No AUC (n=25)

Record Number	Full reference in JMIR/AMA style	PMID	DOI or URL	Reason for exclusion
273	Yue Z, Yan-Meng G, Ji-Zhuang L. Prediction model for acute kidney injury after coronary artery bypass grafting: a retrospective study. Int Urol Nephrol. 2019;51(9):1605-1611.	31161519	doi:10.1007/s11255-019-02173-7	No AUC
292	Koyner JL, Carey KA, Edelson DP, Churpek MM. The Development of a Machine Learning Inpatient Acute Kidney Injury Prediction Model. Crit Care Med. 2018;46(7):1070-1077.	29596073	doi:10.1097/ccm.0000000000003123	No AUC
506	Inohara T, Kohsaka S, Miyata H, et al. Performance and Validation of the U.S. NCDR Acute Kidney Injury Prediction Model in Japan. J Am Coll Cardiol. 2016;67(14):1715-22.	27056778	doi:10.1016/j.jacc.2016.01.049	No AUC
507	Ip IK, Silveira PC, Alper EC, Benson CB, Khorasani R. External validation of risk stratification strategy in the use of renal ultrasonography in the evaluation of acute kidney injury. J Hosp Med. 2016;11(11):763-767.	27186959	doi:10.1002/jhm.2598	No AUC
555	Kim Y, Park N, Kim J, et al. Development of a new mortality scoring system for acute kidney injury with continuous renal replacement therapy. Nephrology (Carlton). 2019;24(12):1233-1240.	31487094	doi:10.1111/nep.13661	No AUC
841	Haines RW, Lin SP, Hewson R, et al. Acute Kidney Injury in Trauma Patients Admitted to Critical Care: Development and Validation of a Diagnostic Prediction Model. Sci Rep. 2018;8(1):3665.	29483607	doi:10.1038/s41598-018-21929-2	No AUC
1006	Motwani SS, McMahon GM, Humphreys BD, Partridge AH, Waikar SS, Curhan GC. Development and Validation of a Risk Prediction Model for Acute Kidney Injury After the First Course of Cisplatin. J Clin Oncol. 2018;36(7):682-688.	29320311	doi:10.1200/jco.2017.75.7161	No AUC
4357	Serif L, Chalikias G, Didagelos M, et al. Application of 17 Contrast-Induced Acute Kidney Injury Risk Prediction Models. CARDIORENAL MEDICINE. 2020;10(3):162-174.	Not available	doi:10.1159/000506379	No AUC
4426	Wang H, Kang X, Shi Y, et al. SOFA score is superior to APACHE-II score in predicting the prognosis of critically ill patients with acute kidney injury undergoing continuous renal replacement therapy. RENAL FAILURE. 2020;42(1):638-645.	Not available	doi:10.1080/0886022X.2020.1788581	No AUC
4485	Ma B, Allen DW, Graham MM, et al. Comparative Performance of Prediction Models for Contrast-Associated Acute Kidney Injury After Percutaneous Coronary Intervention. CIRCULATION-CARDIOVASCULAR QUALITY AND OUTCOMES. 2019;12(11):e005854.	Not available	doi:10.1161/CIRCOUTCOMES.119.005854	No AUC
4529	Simonov M, Ugwuowo U, Moreira E, et al. A simple real-time model for predicting acute kidney injury in hospitalized patients in the US: A descriptive modeling study. PLOS MEDICINE. 2019;16(7):e1002861.	Not available	doi:10.1371/journal.pmed.1002861	No AUC
4537	Ferrari F, Puci MV, Ferraro OE, et al. Development and validation of quick Acute Kidney Injury-score (q-AKI) to predict acute kidney injury at admission to a multidisciplinary intensive care unit. PLOS ONE. 2019;14(6):e0217424.	Not available	doi:10.1371/journal.pone.0217424	No AUC
4667	Naruse H, Ishii J, Takahashi H, et al. Predicting acute kidney injury using urinary liver-type fatty-acid binding protein and serum N-terminal pro-B-type natriuretic peptide levels in patients treated at medical cardiac intensive care units. CRITICAL CARE. 2018;22:197.	Not available	doi:10.1186/s13054-018-2120-z	No AUC
4671	Hodgson LE, Roderick PJ, Venn RM, Yao GL, Dimitrov BD, Forni LG. The ICE-AKI study: Impact analysis of a Clinical prediction rule and Electronic AKI alert in general medical patients. PLOS ONE. 2018;13(8):e0200584.	Not available	doi:10.1371/journal.pone.0200584	No AUC
4683	Elhosseiny S, Akel T, Mroue J, Tathineni P, El Sayegh S, Lafferty J. The Value of Adding Red Cell Distribution Width to Mehran Risk Score to Predict Contrast-induced Acute Kidney Injury in Patients with Acute Coronary Syndrome. Cureus. 2018;10(7):e2911-e2911.	30186716	doi:10.7759/cureus.2911	No AUC
4776	Guo BL, Ouyang FS, Yang SM, et al. Development of a preprocedure nomogram for predicting contrast-induced acute kidney injury after coronary angiography or percutaneous coronary intervention. ONCOTARGET. 2017;8(43):75087-75093.	Not available	doi:10.18632/oncotarget.20519	No AUC
4885	Soliman IW, Frencken JF, Peelen LM, et al. The predictive value of early acute kidney injury for long-term survival and quality of life of critically ill patients. CRITICAL CARE. 2016;20:242.	Not available	doi:10.1186/s13054-016-1416-0	No AUC
4968	Ahmed A, Vairavan S, Akhoundi A, et al. Development and validation of electronic surveillance tool for acute kidney injury: A retrospective analysis. JOURNAL OF CRITICAL CARE. 2015;30(5):988-993.	Not available	doi:10.1016/j.jcrc.2015.05.007	No AUC
5051	Elmistekawy E, McDonald B, Hudson C, et al. Clinical Impact of Mild Acute Kidney Injury After Cardiac Surgery. ANNALS OF THORACIC SURGERY. 2014;98(3):815-822.	Not available	doi:10.1016/j.athoracsur.2014.05.008	No AUC
5062	Hornik CP, Krawczeski CD, Zappitelli M, et al. Serum Brain Natriuretic Peptide and Risk of Acute Kidney Injury After Cardiac Operations in Children. ANNALS OF THORACIC SURGERY. 2014;97(6):2142-2147.	Not available	doi:10.1016/j.athoracsur.2014.02.035	No AUC
5113	Piano S, Rosi S, Maresio G, et al. Evaluation of the Acute Kidney Injury Network criteria in hospitalized patients with cirrhosis and ascites. JOURNAL OF HEPATOLOGY. 2013;59(3):482-489.	Not available	doi:10.1016/j.jhep.2013.03.039	No AUC
5185	Schneider DF, Dobrowolsky A, Shakir IA, Sinacore JM, Mosier MJ, Gamelli RL. Predicting Acute Kidney Injury Among Burn Patients in the 21st Century: A Classification and Regression Tree Analysis. JOURNAL OF BURN CARE & RESEARCH. 2012;33(2):242-251.	Not available	doi:10.1097/BCR.0b013e318239cc24	No AUC
5189	Brown JR, Kramer RS, MacKenzie TA, Coca SG, Sint K, Parikh CR. Determinants of Acute Kidney Injury Duration After Cardiac Surgery: An Externally Validated Tool. ANNALS OF THORACIC SURGERY. 2012;93(2):570-576.	Not available	doi:10.1016/j.athoracsur.2011.11.004	No AUC
5505	Zhou J, Bai Y, Wang X, et al. A simple risk score for prediction of sepsis associated-acute kidney injury in critically ill patients. Journal of nephrology. 2019;32(6):947-956.	Not available	doi:10.1007/s40620-019-00625-y	No AUC

Record Number	Full reference in JMIR/AMA style	PMID	DOI or URL	Reason for exclusion
5522	Masewu A, Makulo JR, Lepira F, et al. Acute kidney injury is a powerful independent predictor of mortality in critically ill patients: a multicenter prospective cohort study from Kinshasa, the Democratic Republic of Congo. BMC nephrology. 2016;17(1):118.	Not available	doi:10.1186/s12882-016-0333-4	No AUC

Supplementary Table S1. Literature Search Strategy

Table S1.a. Search Strategies (PubMed)

Number	Search Terms	Search Hits
1	((((((((((acute kidney injury[Title/Abstract]) OR (acute kidney injuries[Title/Abstract])) OR (acute renal injury[Title/Abstract])) OR (acute renal injuries[Title/Abstract])) OR (acute kidney failures[Title/Abstract])) OR (acute kidney failure[Title/Abstract])) OR (acute renal failure[Title/Abstract])) OR (acute renal failures[Title/Abstract])) OR (acute renal insufficiencies[Title/Abstract])) OR (acute kidney insufficiency[Title/Abstract])) OR (acute renal insufficiency[Title/Abstract])) OR (acute kidney insufficiencies[Title/Abstract])	73261
2	((((((Algorithms[Title/Abstract]) OR (artificial intelligence[Title/Abstract])) OR (AI[Title/Abstract])) OR (machine intelligence[Title/Abstract])) OR (prediction model[Title/Abstract])) OR (machine learning[Title/Abstract])) OR (computing methodologies[Title/Abstract])	387688
3	("acute kidney injury"[Title/Abstract] OR "acute kidney injuries"[Title/Abstract] OR "acute renal injury"[Title/Abstract] OR "acute renal injuries"[Title/Abstract] OR " acute kidney failures"[Title/Abstract] OR "acute kidney failure"[Title/Abstract] OR "acute renal failure"[Title/Abstract] OR "acute renal failures"[Title/Abstract] OR " acute renal insufficiencies"[Title/Abstract] OR "acute kidney insufficiency"[Title/Abstract] OR "acute renal insufficiency"[Title/Abstract] OR "acute kidney insufficiencies"[Title/Abstract]) AND ("Algorithms"[Title/Abstract] OR "artificial intelligence"[Title/Abstract] OR "AI"[Title/Abstract] OR "machine intelligence"[Title/Abstract] OR "prediction model"[Title/Abstract] OR "machine learning"[Title/Abstract] OR "computing methodologies"[Title/Abstract])	1113

Table S1.b. Search Strategies (Embase)

Number	Search Terms	Search Hits
1	algorithms:ab,ti OR 'artificial intelligence':ab,ti OR 'ai':ab,ti OR 'machine intelligence':ab,ti OR 'prediction model':ab,ti OR 'machine learning':ab,ti OR 'computing methodologies':ab,ti	448534
2	'acute kidney injury':ab,ti OR 'acute kidney injuries':ab,ti OR 'acute renal injury':ab,ti OR 'acute renal injuries':ab,ti OR 'acute kidney failures':ab,ti OR 'acute kidney failure':ab,ti OR 'acute renal failure':ab,ti OR 'acute renal failures':ab,ti OR 'acute renal insufficiencies':ab,ti OR 'acute kidney insufficiency':ab,ti OR 'acute renal insufficiency':ab,ti OR 'acute kidney insufficiencies':ab,ti	113487
3	#1 AND #2	1674

Table S1.c. Search Strategies (Cochrane)

Number	Search Terms	Search Hits
1	(acute kidney injury):ti,ab,kw OR (acute kidney injuries):ti,ab,kw OR (acute renal injury):ti,ab,kw OR (acute renal injuries):ti,ab,kw OR (acute kidney failures):ti,ab,kw (Word variations have been searched)	10908
2	(acute kidney failure):ti,ab,kw OR (acute renal failure):ti,ab,kw OR (acute renal failures):ti,ab,kw OR (acute renal insufficiencies):ti,ab,kw OR (acute kidney insufficiency):ti,ab,kw (Word variations have been searched)	10575
3	(acute renal insufficiency):ti,ab,kw OR (acute kidney insufficiencies):ti,ab,kw (Word variations have been searched)	1664
4	(Algorithms):ti,ab,kw OR (artificial intelligence):ti,ab,kw OR (AI):ti,ab,kw OR (machine intelligence):ti,ab,kw OR (prediction model):ti,ab,kw (Word variations have been searched)	57694
5	(machine learning):ti,ab,kw OR (computing methodologies):ti,ab,kw (Word variations have been searched)	5930
6	#1 OR #2 OR #3	13142
7	#4 OR #5	61010
8	#6 AND #7	603

Table S1.d. Search Strategies (Web of Science)

Number	Search Terms	Search Hits
1	(((((((((((((TI=(Algorithms)) OR TI=(artificial intelligence)) OR TI=(AI)) OR TI=(machine intelligence)) OR TI=(prediction model)) OR TI=(machine learning)) OR TI=(computing methodologies)) OR AB=(Algorithms)) OR AB=(artificial intelligence)) OR AB=(AI)) OR AB=(machine intelligence)) OR AB=(prediction model)) OR AB=(machine learning)) OR AB=(computing methodologies) and Preprint Citation Index	2811059
2	(((((((((((((((((((((((TI=(acute kidney injury)) OR TI=(acute kidney injuries)) OR TI=(acute renal injury)) OR TI=(acute renal injuries)) OR TI=(acute kidney failures)) OR TI=(acute kidney failure)) OR TI=(acute renal failure)) OR TI=(acute renal failures)) OR TI=(acute renal insufficiencies)) OR TI=(acute kidney insufficiency)) OR TI=(acute renal insufficiency)) OR TI=(acute kidney insufficiencies)) OR AB=(acute kidney injury)) OR AB=(acute kidney injuries)) OR AB=(acute renal injury)) OR AB=(acute renal injuries)) OR AB=(acute kidney failures)) OR AB=(acute kidney failure)) OR AB=(acute renal failure)) OR AB=(acute renal failures)) OR AB=(acute renal insufficiencies)) OR AB=(acute kidney insufficiency)) OR AB=(acute renal insufficiency)) OR AB=(acute kidney insufficiencies) and Preprint Citation Index	115341
3	#2 AND #1 and Preprint Citation Index	2675

Supplementary Table S2. Details of Extracted Variables

Serial number	Variable	Description
1	Study title	As reported
2	First author	As reported
3	Year	Year of publication
4	Type of study	e.g., retrospective cohort, prospective cohort
5	Country	Country/region where the study was conducted
6	Data source	e.g., electronic health records, in-hospital database, MIMIC-III database
7	Criteria for AKI	e.g., KDIGO, RIFLE, AKIN, or other study-defined criteria
8	Patient population	e.g., all patients, pediatric patients, adult patients
9	Cohort classification method	Method used to divide the included population into training, test, validation, or other datasets
10	Cohorts	e.g., training set, test set, validation set, external validation set
11	Primary outcome	e.g., AKI onset, mortality
12	Sub-outcome	e.g., sepsis, cardiac surgery-associated AKI (CS-AKI), contrast-induced acute kidney injury (CI-AKI)
13	Total sample size	Total number of patients/encounters included in the study
14	Total onset/mortality count	Number of patients/encounters meeting the primary outcome (AKI and/or death, as defined by the study)
15	Sample size	Number of patients/encounters included in the analyzed cohort or final model development/validation dataset
16	Number without outcome	Number of patients/encounters in the analyzed sample who did not experience the primary outcome (AKI and/or death)
17	Number with outcome	Number of patients/encounters in the analyzed sample who experienced the primary outcome (AKI and/or death)
18	Median age (AKI/death)	Median age of patients with the outcome (AKI and/or death), if reported
19	AI model	Type of prediction model or machine-learning model (e.g., LR, RF, XGBoost, NN)
20	Factors	Predictor domains or feature elements incorporated into the model
21	Number of factors	Number of distinct predictor domains or factor groups

Serial number	Variable	Description
22	Number of features	Number of individual data features/variables extracted from the included population
23	Area under the curve (AUC)	Reported area under the receiver operating characteristic curve
24	SD	Standard deviation of AUC (if reported)
25	Lower 95% CI	Lower limit of the 95% confidence interval
26	Upper 95% CI	Upper limit of the 95% confidence interval
27	Accuracy	The proportion of all predictions, both positive and negative, that are correct. Formula: (TP + TN) / (TP + TN + FP + FN)
28	Precision	The proportion of predicted positive cases that are true positives. Formula: TP / (TP + FP); equivalent to PPV in binary classification when the same positive class is used.
29	Sensitivity	The proportion of actual positive cases that are correctly predicted as positive. Formula: TP / (TP + FN)
30	Specificity	The proportion of actual negative cases that are correctly predicted as negative. Formula: TN / (TN + FP)
31	PPV	The probability that a subject with a positive test result truly has the condition. Formula: TP / (TP + FP); mathematically equivalent to Precision in binary classification when the same positive class is used.
32	NPV	The proportion of predicted negative cases that are true negatives. Formula: TN / (TN + FN)
33	F1-score	The harmonic mean of Precision and Sensitivity (Recall), providing a single metric that balances both. Formula: 2 x (Precision x Sensitivity) / (Precision + Sensitivity)

Note: TP = true positive; TN = true negative; FP = false positive; FN = false negative; LR = logistic regression; RF = random forest; NN = neural network.

Clinical application scenario classification

Early risk stratification
Models designed to identify patients at increased risk of AKI or post-AKI mortality before or early during hospitalization using admission characteristics, baseline clinical information, or routinely available electronic health record data.

Perioperative monitoring
Models developed for surgical or procedural populations using preoperative, intraoperative, or early postoperative information to support perioperative risk assessment.

ICU real-time alerting
Models designed for continuous or near-real-time prediction among critically ill patients using dynamic electronic health record data, laboratory trajectories, vital signs, urine output, or other time-updated variables.

Specialty-specific prediction
Models developed for specific disease populations, procedures, or specialties that do not primarily fit the above categories and aim to provide targeted risk prediction within a defined clinical context

Supplementary Table S3. PROBAST+AI Signaling Questions for Risk-of-Bias Assessment

Participants and data sources	1.1	Were appropriate data sources used?
	1.2	Was an appropriate study design used?
	1.3	Did the inclusions and exclusions of study participants result in a representative dataset?
	Risk of bias	Risk of bias introduced by the selection of participants and data sources
	Applicability	Concern that the data of the included participants do not match the review question or the assessor's intended use of the prediction model
Predictors	2.1	Were predictors deffned and assessed in a similar way for all participants?
	2.2	Was any preprocessing of predictors similar for all participants?
	2.3	Were predictor assessments made without knowledge of outcome data?
	2.4	Were the predictors included in the model available at the time the model was intended to be used?
	Risk of bias	Risk of bias introduced by the predictors or their assessment
	Applicability	Concern that the deffnition, preprocessing, assessment, or timing of assessment of the predictors in the model do not match the review question or the assessor's intended use
Outcome	3.1	Were outcomes deffned and assessed appropriately?
	3.2	Were outcomes deffned and assessed in a similar way for all participants?
	3.3	Were outcome assessments made without use or knowledge of predictor data?
	3.4	Was the time interval between predictor assessment and outcome assessment appropriate?
	Risk of bias	Risk of bias introduced by the outcome or its determination
	Applicability	Concern that the outcome, its deffnition, assessment, or timing of assessment do not match the review question or the assessor's intended use
Analysis	4.1	Was model evaluation based on only apparent performance avoided?
	4.2	Was there evidence that the sample size was reasonable?
	4.3	Were participants with missing or censored data handled appropriately in the analysis?
	4.4	If methods to address class imbalance were used, was the evaluation done in a dataset without correction for imbalance?
	4.5	If data splitting was done to create training and test dataset idence that data leakage was avoided?
	4.6	If resampling methods were used to evaluate model performance, were all model development steps replicated in the resampling process?
	4.7	Was the predictive performance of the model evaluated appropriately—for example, calibration, discrimination, and net benefft?
	Risk of bias	Risk of bias introduced by the analysis

Supplementary Table S4. PROBAST+AI Signaling Questions for Reporting Quality Assessment

Participants and data sources	1.1	Were appropriate data sources used?
	1.2	Was an appropriate study design used?
	1.3	Did the inclusions and exclusions of study participants result in a representative dataset?
	Quality	Concern regarding quality of selection of participants and data sources
	Applicability	Concern that the data of the included participants do not match the review question or the assessor's intended use of the prediction model
Predictors	2.1	Were predictors deffned and assessed in a similar way for all participants?
	2.2	Was any preprocessing of predictors similar for all participants?
	2.3	Were predictor assessments made without knowledge of outcome data?
	2.4	Were the predictors included in the model available at the time the model was intended to be used?
	Quality	Concern regarding the quality of the predictors or their assessment
	Applicability	Concern that the deffnition, preprocessing, assessment, or timing of assessment of the predictors in the model do not match the review question or the assessor's intended use
Outcomes	3.1	Were outcomes deffned and assessed appropriately?
	3.2	Were outcomes deffned and assessed in a similar way for all participants?
	3.3	Were outcome assessments made without use or knowledge of predictor data?
	3.4	Was the time interval between predictor assessment and outcome assessment appropriate?
	Quality	Concern regarding quality of the outcome or its determination
	Applicability	Concern that the outcome, its deffnition, assessment, or timing of assessment do not match the review question or the assessor's intende
Analyses	4.1	Was there evidence that the sample size was reaso
	4.2	Were continuous and categorical predictors handled appropriately?
	4.3	Were participants with missing or censored data handled appropriately in the analysis?
	4.4	If methods to address class imbalance were used, was the model or the model predictions recalibrated?
	4.5	Were methods used to address potential model overfftting?
	Quality	Concern regarding quality of the analysis

Supplementary Table S5. Study-Level Characteristics of Included Studies and AKI Prediction Models

ID	Author (Year)	Study Type	Country	Patient Population	Outcome	Clinical application scenario	Disease group	Data Source	Total Sample Size	Datasets	No. of AI Model	Top Factor	Max AUC
1	Tao Sun (2025)	Prospective	China	Adults(> 18 years)	Incidence	Early Risk Stratification	Other/mixed	In hospital	581	Training cohort, Internal validation cohort	1	mALB,IgG,TRF,α1MG,NAG,NGAL,KIM-1,L-FABP,TIMP2,IGFBP7,CAF22 , IL-18	0.844
2	Kang Liu (2025)	Retrospective	USA	Adults(> 18 years)	Incidence	Early Risk Stratification	Other/mixed	In hospital	401062	Test cohort	2		0.859
3	Y Zhang (2025)	Retrospective	China	Adults(> 18 years)	Incidence	Early Risk Stratification	Other/mixed	In hospital	161876	Training cohort, Internal validation cohort, External validation cohort	1	Latest changing rate of Scr,Use of diuretics,Percentage of lymphocyte,Admission to ICU,Use of human albumin,Lowest Scr,NE percentage/albumin ratio,Scr,Lowest BUN,Heart rate,Cardiac troponin I ,Highest NE percentage,BUN,Procalcitonin,Baseline red blood cell count,Lowest D-dimer,Blood platelet count,Respiratory rate,Lactic dehydrogenase,Cardiovascular surgery	0.920
4	Ming Ye (2025)	Prospective	China	Adults(> 18 years)	Incidence	ICU Real-time Alert	Cardiac and cardiac surgery- related	In hospital	54429	Training cohort, Internal validation cohort	1	eGFR,Age,CKD,Diabetes,Dyslipidemia,Heart failure,Hypertension,Admited AMI,Hemoglobin,Total cholesterol, Prothrombin, WBC count,Mean platelet volume, LVEF, Male, Admitted to the surgical department	0.867
5	Luyan Zheng (2025)	Retrospective	China	All	Mortality	Specialty-specific	Liver disease- related	In hospital	1049	Training cohort, Internal validation cohort, External validation cohort	2	red blood cell count (RBC), total bilirubin, high-density lipoprotein (HDL), thrombin time (TT), bicarbonate (HCO3), total cholesterol, red blood cell distribution width (RDW), hepatic encephalopathy (HE), bile acid, total protein, mean corpuscular hemoglobin concentration (MCHC), international normalized ratio (INR), cholinesterase, hematocrit (HCT), BUN	0.837
6	Rima S Zahr (2025)	Retrospective	USA	Adults(> 18 years)	Incidence	Early Risk Stratification	Hematologic/malignancy- related	In hospital	1178	Internal validation cohort	4	SBP_minimum,DBP__maximum,MAP__maximum,HR_maximum,MAP__median,Pulse_minimum,SBP__quantile_q_0.2,Pulse_maximum,SBP_binned_entropy,SBP__maximum,sBP_quantile_q_0.8,SBP_fft_centroid,SBP_quantile_q_0.6,sBP_quantile_q_0.4,MAP_quantile_q.0.6,sBP_mean_abs_change,HR_minimum,MAP__minimum,DBP_minimum,MAP__quantile__q_0.4,	0.940
7	Xiaofei Zhang (2025)	Retrospective	China	All	Incidence	Early Risk Stratification	Hypoperfusion/shock-related	Public databases	2615	Training cohort, Internal validation cohort, External validation cohort	6		0.920
8	Kaori Ambe	Retrospective	Japan	Adults(> 18 years)	Incidence	Specialty-specific	Nephrotoxic/drug- related	In hospital	1253	Internal validation cohort, Test	1	BMI, volume of transfusion, white blood cell (WBC) count, red blood cell count, hemoglobin level, mean corpuscular volume, mean corpuscular hemoglobin (MCH), MCH concentration,	0.820

	(2025)									cohort		neutrophil, lymphocyte, monocyte, total protein, albumin (Alb), C-reactive protein, alanine transaminase, lactate dehydrogenase (LDH), glutamyl transpeptidase, SCr, blood urea nitrogen, glucose (Glu), potassium, chlorine, total bilirubin, eGFR, magnesium (Mg) supplementation, loop diuretic, total dosage, maximum dosage, and cisplatin only.	
9	Li Zhang (2025)	Retrospec tive	China	Adults(> 18 years)	Incidence	Early Risk Stratification	Other/mixed	Public databases	18186	Training cohort, External validation cohort	5	Gender,Admission age,Admission weight,BMI,First heart rate,First diastolic blood pressure,Minimum diastolic blood pressure,Maximum diastolic blood pressure,Mean diastolic blood pressure,First respiratory rate,Minimum respiratory rate,Maximum respiratory rate,Mean respiratory rate,First time temperature,Mininum temperature,Maximum temperature,Mean temperature,Minimum heart rate,Maximum heart rate,Mean heart rate,Maximum systolic blood pressure,Blood oxygen partial pressure minimum,Blood oxygen partial pressure maximum,Blood urea nitrogen,Minimum blood urea nitrogen,Maximum blood urea nitrogen,Average blood urea nitrogen,International standardized rate,Minimum international standardized rate,Maximum international standardized rate,Average international standardized rate,Partial thromboplastin time,Minimum partial thromboplastin time,Maximum partial thromboplastin time,Average partial thromboplastin time,Hematocrit,Minimum hematocrit,Average hematocrit,Hemoglobin,minimum hemoglobin,Maximum hemoglobin,Average hemoglobin,White blood cell,Minimum white blood cell,Maximum white blood cell,Average white blood cell,Alkaline phosphatase,Minimum alkaline phosphatase,Maximum alkaline phosphatase,Average alkaline phosphatase,Maximum lactate,Average lactate,Absolute neutrophil count,Minimum absolute neutrophil count,Maximum absolute neutrophil count,Average absolute neutrophil count,Neutrophil count,Minimum neutrophil count,Maximum neutrophil count,Average neutrophil count,Glucose,Blood potassium,Maximum blood potassium,Average blood potassium,Estimated glomerular filtration rate,Minimum PH,Maximum PH,Average PH,Minimum chloride,Maximum blood sodium,Antibiotic use,Diuretic use,Vasopressor use,Renal replacement therapy ,Mechanical ventilation,Congestive heart failure,Diabetes,Chronic pulmonary disease,Mild live disease,Malignant cancer,Renal disease,Hypertension	0.880
10	Pierre Tran (2025)	Retrospec tive	France	Adults(> 18 years)	Incidence	Perioperative Monitoring	Trauma/non- cardiac surgery- related	In hospital	774		1	body mass index, age, presence of diabetes, duration of surgery, lowest MAP, Charlson score, smoking and glomerular filtration rate	0.980
11	Mengqing	Retrospec	China	Adults(>	Incidence	Early Risk	Sepsis/infection- relat	In hospital	6371	Internal validation	5	sex, temperature, breathing rate, diastolic blood pressure, CRP, albumin, white blood cell count,	0.890

	Ma (2024)	tive		18 years)		Stratification	ed			cohort, External validation cohort		platelet count, hemoglobin, neutrophil count, and BUN	
12	Huilan Tu (2024)	Retrospective	China	Adults(> 18 years)	Incidence	Specialty-specific	Liver disease- related	Public databases	1254	Training cohort, Internal validation cohort	1	MAP,WBC,TB,GCS,Cr,Alb,HR,Plt	0.797
13	Wanyue Zhang (2024)	Retrospective	China	All	Incidence	ICU Real-time Alert	Severe acute pancreatitis (SAP)	In hospital	772	Training cohort, Internal validation cohort	5	Sequential Organ Failure Assessment (SOFA), Acute Physiology and Chronic Health Evaluation (APACHE II), platelet count (PLT), blood urea nitrogen (BUN), blood uric acid (UA), triglyceride (TG), blood amylase (AMY), alanine aminotransferase (ALT), lactate (Lac), and potassium (K+)	0.950
14	Hai-Xia Liu (2024)	Retrospective	China	Adults(> 18 years)	Incidence	Perioperative Monitoring	Liver disease- related	In hospital	1089	Test cohort, Internal validation cohort	5	preoperative indirect bilirubin, intraoperative UO, anesthesia time, preoperative platelets and graft steatosis	0.760
15	Xiang Li (2024)	Retrospective	Korea	Geriatric	Mortality	ICU Real-time Alert	Other/mixed	In hospital	606	Training cohort, Internal validation cohort	1	age, phosphorus (P), CCI, SBP, creatinine (Cr), albumin (Alb), APACHE II score, SOFA score	0.809
16	Junbo Shen (2024)	Retrospective	USA	All	Incidence	Perioperative Monitoring	Cardiac and cardiac surgery- related	In hospital	6502		13	Creatinine,Pulse,Blood urea nitrogen,Chloride,Hemoglobin,Platelet,Glucose,Age,Height,Hematocrit	0.864
17	Xiran Peng (2024)	Prospective	China	Geriatric	Incidence	Perioperative Monitoring	Trauma/non- cardiac surgery- related	In hospital	10561	Test cohort, Internal validation cohort, Training cohort	2	Operation site,Serum cystain Clevel,Hypertension,International normalized ratio,RDW-CV,Urine protein,Operation time,ASA classification,Diabetes mellitus,Emergency surgery	0.845
18	Tao Sun (2024)	Prospective	China	Adults(> 18 years)	Incidence	Early Risk Stratification	Other/mixed	In hospital	588	Training cohort, Internal validation cohort, External validation cohort	6	sCysC,IL-6,NT-proBNP,NGAL,L-FABP,KIM-1,IGFBP7,CAF22,IL-18	0.902
19	Emma Schwager (2024)	Retrospective	USA	Adults(> 18 years)	Incidence	Early Risk Stratification	Other/mixed	In hospital	50342	Training cohort, Test cohort, Internal validation cohort	1	Average urine output,Creatinine (mg/dL),Fluid balance,vent on (1=yes),Weight(kg),medications_diureticsIV(count),scores_sofa_SOFA Score (calculated),vent duration (hrs),Respiratory Rate (mean),Fi02 (%)	0.756
20	Lingyu	Retrospec	China	Pediatric	Incidence	Early Risk	Other/mixed	In hospital	1685	Test cohort	8	Scr,WBC,Neutrophil count,LDH,Hypnotics and sedatives,Prothrombin	0.813

	Xu (2024)	tive				Stratification						activity,MCHC,Phosphorus,Total protein,RBC,Potassium,Glucose,Age,Procalcitonin,Prothrombin time,	
21	Xiaochi Lu (2024)	Retrospective	China	Adults(> 18 years)	Incidence	Specialty-specific	Nephrotoxic/drug- related	In hospital	3920	Training cohort, Test cohort	9	Age,Lengthofstay,Fibrinogen,Bloodplatelet,Glomerularfiltrationrate,Serumcreatinine,Serumkalium,Ureanitrogen,Lowdensitylipoprotein,Totalcholesterol,NIHSSscore,Drinking,Diabetes,Hemiplegia,Whitebloodcell,Glucose,Respiratoryrate,Heartrate,BMI,	1.000
22	Jun Wei (2024)	Retrospective	China	Adults(> 18 years)	Incidence	Early Risk Stratification	Hypoperfusion/shock- related	In hospital	6891	Training cohort, Test cohort, External validation cohort	12	estimated Glomerular Filtration Rate,CreatinineBlood Urea Nitrogen,Cardiogenic shock,Creatine kinase isoenzymes MB,Troponin TGlucose,HemoglobinAgeHeart RatePhosphate	1.000
23	Rufa Zhang (2024)	Retrospective	China	All	Incidence	ICU Real-time Alert	Severe acute pancreatitis (SAP)	In hospital	437	Internal validation cohort	4		0.830
24	Ruoran Wang (2024)	Retrospective	China	All	Incidence	Early Risk Stratification	Trauma/non- cardiac surgery- related	In hospital	376		2	Glucose,SUA,CystatinC,Scr,Cholesterol,Cl,PT,Phosphorus,GCS,Monocyte	0.892
25	Jiaxin Li (2024)	Prospective	China	Adults(> 18 years)	Incidence	Perioperative Monitoring	Cardiac and cardiac surgery- related	In hospital	1002	Training cohort, Internal validation cohort	1	age, hypertension, baseline sCr, preoperative albumin, preoperative hemoglobin, postoperative NT-proBNP, postoperative cTnI, CABG combined with valve surgery, CPB duration, sCysC, uNAG	0.840
26	Yongbin Wang (2024)	Retrospective	China	Adults(> 18 years)	Mortality	ICU Real-time Alert	Other/mixed	In hospital	1395	Training cohort, External validation cohort	9	age, platelet count, serum creatinine, prothrombin time, anion gap, potassium, cardiac arrest, septic shock, administration of epinephrine, and receipt of mechanical ventilation	0.954
27	XinPei Liu (2024)	Retrospective	China	All	Incidence	Perioperative Monitoring	Trauma/non- cardiac surgery- related	In hospital	527	Training cohort, Internal validation cohort	6	ValveReplacement,PreOp_HTN,PreOp_LargeVeg,PreOp_NYHA,PreOp_Achl,Age,PostOp_LCOS,PreOp_TyG,PreOp_Ccr,CPBtime,IntraOp_RBC,IntraOp_Ur,PreOp_Hgb,SurgeryTiming,Sex,PreOp_CNSC,PreOp_LHR,PreOp_SII,BMI,PostOp_Reexploration,PreOp_CRP,PreOp_SIRI,PreOp_CTD,PreOp_AISI,PreOp_KidneyEmbolism	1.000
28	Shanbing Hou (2024)	Retrospective	China	Adults(> 18 years)	Incidence	ICU Real-time Alert	Hypoperfusion/shock- related	In hospital	347		1	post-resuscitation shock, CRP, LDH, ALP	0.713
29	Meicheng Yang	Retrospective	China	Adults(> 18 years)	Incidence	Early Risk Stratification	Other/mixed	In hospital	26813	Internal validation cohort, External	4	age, gender, weight, height, chronic kidney disease, diabetes, chronic pulmonary disease, congestive heart failure, moderate/severe liver disease, current ICU length of stay, heart rate,	0.799

	(2024)									validation cohort		temperature, systolic blood pressure, mean arterial pressure, diastolic blood pressure, respiration rate, SpO2, pH, pO2, pCO2, FiO2, base excess, lactate, glucose, hematocrit, hemoglobin, white blood cells, platelet, albumin, anion gap, bicarbonate, blood urea nitrogen, creatinine, calcium, chloride, sodium, potassium, international normalized ratio, prothrombin time, alanine aminotransferase, alkaline phosphatase, aspartate aminotransferase, total bilirubin, total 12-hour urine output, PO2/FiO2 ratio, blood urea nitrogen/creatinine ratio, body mass index, lowest serum creatinine value in the last 48 hours, total 12-hour urine output/weight/12 h, binary indicator for missingness of each vital sign and laboratory value, difference between current and previous value of each vital sign and laboratory value, maximum of each vital sign in 24-hour sliding window, minimum of each vital sign in 24-hour sliding window, median of each vital sign in 24-hour sliding window, standard deviation of each vital sign in 24-hour sliding window, differential standard deviation of each vital sign in 24-hour sliding window	
30	Lei Dong (2024)	Retrospective	China	Adults(> 18 years)	Mortality	Early Risk Stratification	Sepsis/infection-related	In hospital	7544	Training cohort, Internal validation cohort, External validation cohort	5	gcs_min,urineoutput,lactate max gas,anchor age,ptt max,temperature maX,aniongap_max,pt max,heart rate maX,spo2 min	0.936
31	Mee Yeon Lee (2024)	Retrospective	Korea	Adults(> 18 years)	Incidence	Early Risk Stratification	Nephrotoxic/drug-related	In hospital	2272257	Training cohort, Internal validation cohort	1	age, male sex, history of acute kidney injury, cerebrovascular disease, dementia, urinary tract infection, beta blockers, aminoglycosides, low osmolar ionic contrast agents, high osmolar ionic contrast agents, proton-pump inhibitors, combination of renin-angiotensin-aldosterone system inhibitors with diuretics or nonsteroidal anti-inflammatory drugs, combination among different types of diuretics	0.728
32	Caifeng Li (2024)	Retrospective	China	Adults(> 18 years)	Mortality	Early Risk Stratification	Sepsis/infection-related	Public databases	1663	Training cohort, Test cohort	3	age, MAP, RR, lactate, Cr, PT-INR, TBIL and CVP	0.790
33	Xiao Yue (2024)	Retrospective	China	Adults(> 18 years)	Mortality	Early Risk Stratification	Sepsis/infection-related	Public databases	11334	Training cohort	1	LODS, Charlson comorbidity index, urine output, INR, respiratory support mode, blood urea nitrogen, age	0.810
34	Xiang Li (2024)	Retrospective	USA	Adults(> 18 years)	Incidence	Specialty-specific	Liver disease-related	Public databases	886		1	age, gender (male), cerebrovascular disease, heart rate, respiratory rate, body temperature, SpO2, hemoglobin, BUN, serum creatinine, INR, bilirubin, urine output, SOFA score	0.811
35	Yufeng Jiang	Retrospective	China	All	Incidence	Early Risk Stratification	Other/mixed	In hospital	1016	Training cohort, Internal validation	1	age, fever, diabetes, hyperuricemia, bilateral calculi, functional solitary kidney, self-medication, prehospital delay.	0.818

	(2024)									cohort			
36	Chen Lin (2024)	Retrospective	China	Adults(> 18 years)	Incidence	Early Risk Stratification	Sepsis/infection-related	In hospital	391	Training cohort, Internal validation cohort	1	age, mean arterial pressure, sedative, mechanical ventilation, vasopressors, hypertension, septic shock, C-reactive protein, procalcitonin (PCT), platelets (PLT), D-dimer, prothrombin (PT), and activated partial thromboplastin time (APTT)	0.870
37	Buqing Ma (2024)	Retrospective	China	Adults(> 18 years)	Incidence	Early Risk Stratification	Cerebrovascular-related	In hospital	2089	Training cohort, Internal validation cohort	1	weight, prior congestive heart failure, GCS score, urine output, heart rate, blood glucose level, WBC, blood calcium concentration, vasoactive drugs, furosemide, invasive mechanical ventilation, supplemental oxygen	0.848
38	Rao Sun (2024)	Retrospective	china	Adults(> 18 years)	Incidence	Perioperative Monitoring	Trauma/non-cardiac surgery-related	In hospital	88367	Test cohort, External validation cohort	4	age, duration of surgery, preoperative serum GGT ， creatinine, ASA physical status III	0.853
39	Wenjuan Zhang (2024)	Retrospective	China	All	Incidence	Perioperative Monitoring	Trauma/non-cardiac surgery-related	In hospital	710	Training cohort, Internal validation cohort	9	gender, age, weight, height, diabetes, hypertension, type of operation, baseline hemoglobin, white blood cell count, platelet, alanine aminotransferase (ALT), aspartate aminotransferase (AST), albumin, total bilirubin, creatinine, blood urea nitrogen (BUN), uric acid (UA), medical history before admission to ICU	1.000
40	Lei Yuan (2024)	Retrospective	China	All	Incidence	ICU Real-time Alert	Severe acute pancreatitis (SAP)	In hospital	672	Training cohort, Internal validation cohort, External validation cohort	1	PA_WV_LHL_GLDM_GLV,PA_WV_LLL_NGTDM_Complexity,PA_WV_LHH_GLSZM_SZN,PA_WV_LHL_GLRLM_RV,EP_WV_LHH_GLSZM_SZN EP_WV_LLH_ GLRLM_LRE	0.900
41	Enci Cai (2024)	Retrospective	USA	Adults(> 18 years)	Incidence	Specialty-specific	Cardiac and cardiac surgery-related	In hospital	8636	Test cohort	3	age, hypertension, diabetes mellitus, anemia, severe frailty, heart failure, chronic kidney disease (CKD) stage, cardiac arrest (responsive and unresponsive), cardiovascular instability, concomitant procedures, PCI status, ST-elevation myocardial infarction (MI), non-ST-elevation MI/unstable angina, mechanical ventricular support, PCI of the proximal left anterior descending (LAD) artery.maximum history systolic blood pressure, minimum history systolic blood pressure, mean history systolic blood pressure, history systolic blood pressure fluctuation, maximum history diastolic blood pressure, minimum history diastolic blood pressure, mean history diastolic blood pressure, history diastolic blood pressure fluctuation, admission date systolic blood pressure, admission date diastolic blood pressure, procedure date systolic blood pressure, procedure date diastolic blood pressure, number of history blood pressure measurements, number of previous hospitalizations, number of previous elective admissions, number of previous emergency admissions, pre-procedure elective admission status.	0.859

42	Chi, Cheng (2024)	Prospective	China	Adults(> 18 years)	Incidence	ICU Real-time Alert	Severe acute pancreatitis (SAP)	In hospital	258	Training cohort	1	age, triglyceride glucose index (TyG), procalcitonin (PCT)	0.856
43	Junlong Hu (2024)	Prospective	China	Pediatric	Incidence	Early Risk Stratification	Other/mixed	In hospital	1823	Internal validation cohort, External validation cohort	11	eGFR,UO,PRISM III,BUN,MAP min,TBil,Body weight,APTT,PCT,INR,PaCO2 max,Lac max,PLT_min,ALT,CRP,PaO2/FiO2_min,Glucose,LDH,pH,HCO3-,	0.940
44	Shengtao Li (2024)	Retrospective	China	Adults(> 18 years)	Mortality	Early Risk Stratification	Other/mixed	In hospital	1079		1	age , anemia , hypotension , organ failure, admission Scr level	0.828
45	Sang-Wook Lee (2024)	Retrospective	Korea	Adults(> 18 years)	Incidence	Perioperative Monitoring	Trauma/non- cardiac surgery- related	In hospital	81544	Internal validation cohort, External validation cohort	4	Age, years Sex BMI (kg/m2) ASA physical status ,White blood cell, 103/μL Hemoglobin, g/dL Sodium, mmol/L Platelet, 103/μL Potassium, mmol/L Chloride, mmol/L Total Bilirubin, mg/dL Albumin, g/dL AST, IU/L ALT, IU/L Hematocrit, % eGFR, ml/min/1.73 m2 Glucose, mg/dL PT, INR aPTT, sec BUN, mg/dL Creatinine, mg/dL CRP, mg/dL ,Maximum, SBP Minimum, SBP Mean, SBP Standard deviation, SBP Sum of delta blood pressure, SBP Maximum, DBP Minimum, DBP Mean, DBP Standard deviation, DBP Sum of delta blood pressure, DBP Maximum, MBP Minimum, MBP Mean, MBP Standard deviation, MBP Sum of delta blood pressure, MBP , Maximum, SBP Minimum, SBP Mean, SBP Standard deviation, SBP Sum of delta blood pressure, SBP Maximum, DBP Minimum, DBP Mean, DBP Standard deviation, DBP Sum of delta blood pressure, DBP Maximum, MBP Minimum, MBP Mean, MBP Standard deviation, MBP Sum of delta blood pressure, MBP , Estimated blood loss Surgery time Anesthetic time	0.868
46	Xunliang Li (2024)	Retrospective	China	Adults(> 18 years)	Incidence	Early Risk Stratification	Other/mixed	Public databases	53150	Training cohort	6	SOFA score,Weight,Mechanical ventilation,SAPS II score,Sepsis,eGFR,WBC,Sp02,BUN,Creatinine,Serum potassium,PTT,Congestive heart failure,Age,Platelets,PT,Glucose,Heart rate,dialysis,Body temperature,	0.816
47	Minoru Sakuragi (2024)	Retrospective	Japan	Adults(> 18 years)	Mortality	Specialty-specific	Hematologic/malignancy- related	In hospital	616	Test cohort	1	CRP(-1wk),lungLDH(-4wk),CRP(-2wk),RBC(-3wk),med_ppi(-1wk),WBC(-2wk),ALB(-2Wk),basal SCrNeutrophil(-2wk),Lymphocyte(-1wk),LDH(-2wk),eGFR(-1wk),TG(-1wk),eGFR(-3wk),skinLDH(-1wk),ACTH(-4wk),ALB(-4wk),MCHC(-1wk),	0.880
48	Changho Han (2024)	Retrospective	South Korea	Adults(> 18 years)	Incidence	Perioperative Monitoring	Cardiac and cardiac surgery- related	In hospital	2003	Internal validation cohort	1	age, sex, blood pressures, DM, HTN, chronic kidney disease, congestive heart failure, EuroSCORE II, etc (skipped for brevity), WBC, Hb, PLT, Na+, K+, BUN, eGFR, albumin, etc (skipped), beta blocker, calcium channel blocker, ACEi, ARB, PO nitrate, diuretics, warfarin, heparinization, etc (skipped),anesthesia/operation time, cardiopulmonary bypass/aortic cross	0.840

												clamp/total circulatory arrest duration, FiO2 after induction/after cardiopulmonary bypass weaning/during sternum closure, norepinephrine, vasopressin, primacor, dobutamine, crystalloid, colloid, packed RBC, fresh frozen plasma, platelet concentrate, cryoprecipitate, urine output, cellsaver, hemofiltration, Katz atheroma grade,BUN, creatinine, eGFR, Hb, PO2, albumin, Na+, K+, tCO2, packed RBC, fresh frozen plasma, platelet concentrate, cryoprecipitate,Baseline regional oxygen saturation (rSO2), Average MAP, CV/ARV of MAP, Duration/AUC of MAP < 65mmHg, Duration/AUC of MAP > 100mmHg, Average perfusion,pressure (PP), Duration/AUC of PP < 60mmHg, Average CVP, Duration/AUC of CVP > 12mmHg, Average CI, Duration/AUC of CI < 2 L/min/m2	
49	Kevin Y Heo (2024)	Retrospective	USA	All	Incidence	Perioperative Monitoring	Trauma/non- cardiac surgery- related	In hospital	141697	Test cohort	5	Chronic renal disease,Hypertension,Diabetes w/o complications,Older age,Congestive heart failure	0.750
50	Jianshan Shi (2024)	Retrospective	China	Adults(> 18 years)	Incidence	Early Risk Stratification	Sepsis/infection- related	Public databases	10575	Training cohort	7	First urine output,OASIS,First care unit,Pa02,Glucose,Age,Temperature max,Creatinine,PTT,Chloride ,Calcium,Phosphate,BUN,SBP min,Magnesium,PT,DBP min,Heart rate max,Resp rate max,Potassium,Platelets,Paco2,WBC,Sp02 min,Hematocrit,PH,MBP min,Sodium,HCo3,RBC,	0.801
51	Le Li (2024)	Retrospective	China	Adults(> 18 years)	Mortality	Early Risk Stratification	Sepsis/infection- related	Public databases	14408	Internal validation cohort, External validation cohort	6	Age,LOS_ICU,GCS score,HTN,CKD,HGB,CRE,BUN,AST,UO,	0.813
52	Yamin Liu (2024)	Retrospective	China	Adults(> 18 years)	Mortality	ICU Real-time Alert	Severe acute pancreatitis (SAP)	In hospital	1265	Training cohort, External validation cohort	3	age, neutrophils, RDW, BUN, albumin, SBP, RRT and vasopressor	0.941
53	Tianyun Gao (2024)	Retrospective	China	Adults(> 18 years)	Mortality	Early Risk Stratification	Sepsis/infection- related	Public databases	12196	Training cohort	8	the GCS score, age, temperature, blood sodium level, absolute lymphocyte count, respiratory rate, SAPS II score, urine output, creatinine level, AKI stage, and body mass index (BMI)	0.798
54	Yuezi Song (2024)	Retrospective	China	All	Incidence	Perioperative Monitoring	Cardiac and cardiac surgery- related	In hospital	701	Training cohort	8	Insulin aspart,Acarbose,Spirolactone,Alfentanil,Dezocine,Levosimendan,Clindamycin,MI,Sex,SctO2(L),SctO2(R),Lactate,CK,Troponin,HR,Family history of surgery,Clopidogrel,Age,SVRIrbesartan	0.835
55	Benjamin R Griffin	Retrospective	America	Adults(> 18 years)	Incidence	Early Risk Stratification	Other/mixed	In hospital	18180	Test cohort	1	age, sex, race, diabetes, liver disease, coronary artery disease, congestive heart failure, peripheral vascular disease, malignancy, cerebrovascular disease,white blood cell count, hemoglobin,	0.820

	(2024)											platelets, serum creatinine, blood urea nitrogen,highest temperature, highest daily mean arterial pressure ,lowest daily MAP,admitting service,baseline creatinine; and daily medication administration data for the 57 nephrotoxic medications	
56	Katarzyna Krzanowska (2024)	Retrospective	Poland	Adults(> 18 years)	Incidence	Early Risk Stratification	Respiratory/ARDS-related	In hospital	4691	Test cohort	1	Respiratory support,PCT,CKD,Myoglobin,Post Ktx,Neutrophil count,Circulatory failure,Age,HTN,Prior AKI	0.798
57	Xuejian Hou (2024)	Retrospective	China	All	Incidence	Perioperative Monitoring	Cardiac and cardiac surgery-related	In hospital	1767	Internal validation cohort	5	The ejection fraction (EF), eGFR, age, albumin (Alb), uric acid (UA), lactate dehydrogenase (LDH)	0.834
58	Shengwei Lin (2024)	Retrospective	China	Adults(> 18 years)	Incidence	ICU Real-time Alert	Severe acute pancreatitis (SAP)	Public databases	1235	Training cohort, Test cohort	7	urineoutput,mv_invas,wbc_mean,vaso,heart_rate_mean,resp_rate_mean,creatinine_mean,bun_mean,sbp_mean,dbp_mean,mbp_mean,bicarbonate_mean,aniongap_min,potassium_mean,calcium_mean,glucose_mean,chloride_mean,platelets_mean,hemoglobin_mean,hematocrit_mean,temperature_mean,spo2_mean,admission_age,sepsis3	0.867
59	Amir Hossein Behnoush (2024)	Retrospective	Iran	All	Incidence	Specialty-specific	Nephrotoxic/drug-related	In hospital	4592	Test cohort	5		0.775
60	Jie Tang (2024)	Retrospective	China	Geriatric	Mortality	Early Risk Stratification	Sepsis/infection-related	Public databases	8426	Training cohort, Internal validation cohort	11	AKI stage, partial pressure of oxygen (PaO2), lactate, urine output, rate of norepinephrine, blood urea nitrogen (BUN) level, invasive mechanical ventilation (IMV), base excess, and anion gap (AP)	0.987
61	Menglei Gu (2024)	Retrospective	China	Adults(> 18 years)	Mortality	ICU Real-time Alert	Other/mixed	Public databases	1535	Internal validation cohort	9	SOFA-ORRT-Oh,WBC,SBP,Phosphorous,BUN,BMI,eGFR,UO2,Bicarbonate,DBP,Hb,Alb,Potassium,Age,HTN,CHF,MV-CRRT,Sex,DM,AKIN,Cerevascular-dls,MI,COP,PVD,Dementia	0.800
62	Shengqiang Chi (2023)	Retrospective	China	Adults(> 18 years)	Incidence	Specialty-specific	Hypoperfusion/shock-related	Public databases	1415	Test cohort	7	Angiography,PCI,Antiplatelet,Anticoagulant,Beta-blocker,Positive inotropic drugs,Vasodilator,ACEI/ARB,Calcium Channel,Blockers,Diabetes,Valvular Heart Disease,Cardiomyopathy,Coronary Heart,DiseaseStroke,Auricular Fibrillation,Acute Heart Failure,Sex,malePotassium, mmol/L,Hemoglobin, g/L,Age.year,DBP, mmHg,SBP, mmHg,Triglyceride, mmol/LALT, IU/L,Total Bilirubin, umolI,Urea, mmol/L,AST, IU/LLDL-C, mmol/L,eGFR,NT-proBNP,	0.791
63	Mengqing Ma	Prospective,Retros	China	All	Incidence	Specialty-specific	Nephrotoxic/drug-related	In hospital	3514	Internal validation cohort, External	6	ACS, Urine protein level, Diuretics, left ventricular ejection fraction (LVEF)(%), Hemoglobin, CHF, Stable Angina, Uric acid, Preoperative DBP, Contrast Volumes, Albumin, Baseline	0.819

	(2023)	pective								validation cohort		creatinine, Vessels of coronary artery disease, Glucose and Diabetes history	
64	Yuchen Gao (2023)	Retrospective	China	Adults(> 18 years)	Incidence	Perioperative Monitoring	Cardiac and cardiac surgery- related	In hospital	15880	Training cohort, Internal validation cohort	2	Postoperative eGFRPostoperative CreatininePreoperative eGFRPostperative NT proBNPSurgical timeCPB timePreoperative AlbuminAgePreoperative ClPreoperative HsCRPIntra operative PlasmaCPBPreoperative PLTPreoperative NaHypertension	0.850
65	Liu Li (2023)	Retrospective	China	Adults(> 18 years)	Incidence	Perioperative Monitoring	Hematologic/malignancy- related	In hospital	389	Training cohort	1	preoperative combined hypertension, preoperative anemia, inadequate intraoperative crystalloid infusion, a low intraoperative minimum MAP and a moderate to severe postoperative decline in Hb levels	0.776
66	Hanlim Choi (2023)	Retrospective	South Korea	Adults(> 18 years)	Incidence	Early Risk Stratification	Trauma/non- cardiac surgery- related	In hospital	400	Test cohort	2	use of vasopressor,cumulative fluid balance 24h, pRBC transfusion 4h, lactate, Age, Hypotension, Mean corpuscular volume, Hemothorax, Intestinal injury ISS, AIS-Chest	0.912
67	Xinyi Cao (2023)	Retrospective	China	Adults(> 18 years)	Incidence	Early Risk Stratification	Sepsis/infection- related	Public databases	3278	Training cohort, Internal validation cohort	1	congestive heart failure (CHF), hypertension, diabetes, transcutaneous oxygen saturation (SpO2), 24-h urine output, white blood cells (WBC), serum creatinine (Scr), prothrombin time (PT), potential of hydrogen (PH), vasopressor use, mechanical ventilation (MV) use	0.770
68	Ziming Jiang (2023)	Retrospective	China	Adults(> 18 years)	Incidence	ICU Real-time Alert	Severe acute pancreatitis (SAP)	Public databases	963	Training cohort, Internal validation cohort	1	weight, SOFA score, white blood cell count, albumin, chronic heart failure, sepsis	0.820
69	Zhiyan Fan (2023)	Retrospective	China	Adults(> 18 years)	Mortality	Early Risk Stratification	Sepsis/infection- related	In hospital	2599	Training cohort, External validation cohort	5	sofa,urineoutput,lactate_min,Sp02_mean,resp_rate_mean,inr_max,meanbp_mean,aniongap_min,bicarbonate_min,inr_min,heart_rate_max,Sp02_min,admission_age,weight,temperature_mean,creatinine_min,lactate_max,temperature_min,meanbp_max,chloride _min,glucose_min,sodium_max,aniongap_max,bun_min,potassium_max,resp_rate_min,hematocrit_min,wbc_min,meanbp_min,glucose_mean,heart_rate_mean,heart_rate_min,bicarbonate_max,hematocrit_max,resp_rate_max,creatinine_max,hemoglobin_max,platelets_min,bun_max,platelets_max,	0.910
70	Fangfang Zhou (2023)	Retrospective	China	Adults(> 18 years)	Incidence	Specialty-specific	Nephrotoxic/drug- related	In hospital	2230		5	Cardiacfailure,FPG,Urine protein,β-receptor inhibior,Loop diuretic,Biguanide,NLR,Scr,Spirolactone,CHD,Hb,GFR,	0.774
71	Luyan Zheng (2023)	Retrospective	China	All	Incidence	Specialty-specific	Liver disease- related	Public databases	1938	Internal validation cohort, External validation cohort	8	sCr,shock index(SI),prothrombin time(PT),mean corpuscular hemoglobin(MCH) ,Total bilirubin,magnesium,Gender,Lymphocyte	0.765
72	Hayato	Retrospec	Japan	Adults(>	Incidence	Early Risk	Nephrotoxic/drug- rel	In hospital	65667	Training cohort	6	Age,Male,CephalosporinsHypertension [MH],Heart failure [MH],NSAIDs,Surugadai	0.827

	Akimoto (2023)	tive		18 years)		Stratification	ated					[HOS),Sepsis [MH],Itabashi [HOS],Anemia [MH],Loop diuretics,Diabetes [MH],Glycopeptides,PPIs,Aldosterone antagonists,Platinum-based agents,Statins,H2 blockers,Cephalosporins * H2 blockers,ARBs,Hikarigaoka [HOS],Penicillins,Calcium channel blockers,Chronic liver disease [MH],SMXTMP,Statins * ARBs,Carbapenems,Penicillins NSAIDs,Loop diuretics * H2 blockers,Cephalosporins* PPIs	
73	Yan Luo (2023)	Retrospective	China	Adults(> 18 years)	Incidence	Early Risk Stratification	Other/mixed	Public databases	15218	Test cohort	7	frequency, TVU, Scr_baseline, BUN, K, PO2, GCS, PH	0.849
74	Qian Li (2023)	Retrospective	China	Adults(> 18 years)	Incidence	Perioperative Monitoring	Cardiac and cardiac surgery- related	In hospital	6495	Training cohort, External validation cohort	12	BUN,PT,PLT,SCr,TBil,ALB,age,SBP,weighthistory_valvularhistory_congenitalHRDBP,history ph,height,surgery_valvularASTsurgery_congenitalALTHgb	0.846
75	Sheng Shi (2023)	Retrospective	China	Pediatric	Incidence	Perioperative Monitoring	Cardiac and cardiac surgery- related	In hospital	1137	Training cohort, Internal validation cohort	1	age at operation, cyanosis, CPB duration >120 min, cross-clamp time, baseline albumin, baseline creatinine	0.755
76	Ling Pan (2023)	Retrospective	China	Adults(> 18 years)	Incidence	Perioperative Monitoring	Cardiac and cardiac surgery- related	In hospital	1159	Training cohort, Internal validation cohort	1	age, hemoglobin, fibrinogen, serum uric acid, cystatin C, bicarbonate, and cardiopulmonary bypass time	0.777
77	Ping Yan (2023)	Retrospective	China	Adults(> 18 years)	Incidence	Specialty-specific	Nephrotoxic/drug- related	In hospital	4218	Training cohort, Internal validation cohort, External validation cohort	2	neutrophil count, eGFR, platelet count, serum calcium, serum sodium, blood urea nitrogen, diuretics, proteinuria, renal CTA, mechanical ventilation, serum albumin, total protein, serum total bilirubin and antimicrobial drugs	0.992
78	Lu-Huai Feng (2023)	Retrospective	USA	Adults(> 18 years)	Incidence	Specialty-specific	Liver disease- related	Public databases	4375	Training cohort, Internal validation cohort	1	ascites, CKD, shock, sepsis, diuretic drugs, hepatic encephalopathy, bacterial infections, vasoactive drugs, admission age, total bilirubin, BUN	0.786
79	Simin Wu (2023)	Retrospective	China	Adults(> 18 years)	Incidence	ICU Real-time Alert	Severe acute pancreatitis (SAP)	Public databases	799	Training cohort, Internal validation cohort	1	age, race, total bilirubin, activated partial thromboplastin time (APTT), need for mechanical ventilation, use of vasoactive drugs, sepsis	0.795
80	Siavash Zamirpour (2023)	Retrospective	USA	Adults(> 18 years)	Incidence	Perioperative Monitoring	Trauma/non- cardiac surgery- related	In hospital	10623	Internal validation cohort, Training cohort	3	last preoperative serum creatinine, minimum serum creatinine within 90 days before the procedure, minutes with urine output ≤ 31.29 mL/h, minutes with heart rate > 85, minutes with perfusion index ≤ 0.8%, and total estimated blood loss	0.810
81	Yen-Yu	Retrospec	China	Adults(>	Incidence	Specialty-specific	Nephrotoxic/drug- rel	In hospital	18895	Training cohort	2	Inotropic agentsHypertensionDMsexACEV/ARBICU admissionAminoglycosidesHeart	0.769

	Chen (2023)	tive		18 years)			ated					failureeGFRCirrhosisHRRepeated contrast examAge,HbDBP	
82	Jiakang Shao (2023)	Retrospective	China	Adults(> 18 years)	Incidence	Perioperative Monitoring	Cardiac and cardiac surgery- related	In hospital	2108	External validation cohort	6	IVST,Baseline ACT,Left atrial diameter,LVEF,DBP,QTc interval,Neutrophil counts,INR,Neutralizated ACT,Right atrial diameter,Hgb,Body weight,Ascending aorta diameter,Weight of the intervention,Creatinine clearance,P-R interval,HCT,Scr,CI,QRS duration	0.820
83	Dawei Chen (2023)	Retrospective	China	Adults(> 18 years)	Incidence	Early Risk Stratification	Sepsis/infection- related	In hospital	2883	Training cohort, Test cohort, Internal validation cohort	1	albumin, acute respiratory failure, CURB-65 score, Cystatin C, white cell count	0.878
84	Huanhuan Shi (2023)	Retrospective	China	Adults(> 18 years)	Incidence	Early Risk Stratification	Sepsis/infection- related	Public databases	12511	Training cohort, Internal validation cohort, External validation cohort	3	Bilirubin_max,Bicarbonate_min,RRT,Mechanical ventilation,First_time_bilirubin,First_time_albumin,Albumin_min,Postassium_max,Bilirubin_min, Hemoglobin_min,First_time_bicarbonate,CKD,Albumin_max,MAP_min,Glucose_max,	0.890
85	Muying Wu (2023)	Retrospective	China	Adults(> 18 years)	Incidence	Perioperative Monitoring	Trauma/non- cardiac surgery- related	In hospital	582	Test cohort	5		0.845
86	Jijun Yang (2023)	Retrospective	China	Adults(> 18 years)	Mortality	Early Risk Stratification	Sepsis/infection- related	Public databases	9158	Training cohort	4	log_urine_output,Spo2,Temperature,Age,Ph,log_Glucose,X1.sqrt_BUN,Sodium,cerebrovascular_disease.1,log_WBC,X1.Paco2,metastatic_solid_tumor.1,sqrt_Bicarbonate,Chloride,X1.Potassium,X1.sqrt_Anion_gap,MBP,Heartrate,X1.sqrt_Creatinine,Hemoglobin,X1.sqrt_Lactate,X1.sqrt_Pao2,log_Neutrophils,severe_liver_disease.1,DBP,SBP,log_Pao2Fio2_ratio,mild_liver_disease.1,X1.sqrt_BMI,log_NLR,Albumin,X1.Respiration_rate,log_LMR,log_ROX_HR,dementia.1	0.873
87	Hsin-Hung Liu (2023)	Retrospective	China	All	Incidence	Early Risk Stratification	Other/mixed	Public databases	12702		3	serum creatinine, serum chloride, serum magnesium, serum phosphorus	0.857
88	Chun-Te Huang (2023)	Retrospective	China	Adults(> 18 years)	Incidence	Early Risk Stratification	Other/mixed	In hospital	55156	Internal validation cohort, External validation cohort	5	Age,SBP, DBP, Pulse pressure, SpO2, Respiratory rate, pulse rate, Temperate, urine 8/16/24 hours,WBC count, Neutrophil, Hemoglobulin, Platelet count, BUN, Serum creatinine, Total Bilirubin,Renin-angiotensin system inhibitors, diuretics, PPI, H2 receptor antagonists, NSAID-COX I inhibitors, NSAID COX II inhibitors, vasopressin, Norepinephrine, dopamine, epinephrine, dobutamine, vancomycin, Bactrim, Gentamicin, Amikacin, Colistin, Amphotericin B, Total medication,Renin-angiotensin system inhibitors, diuretics, PPI, H2 receptor antagonists,	0.928

												NSAID-COX I inhibitors COX II inhibitors, vasopressin, Norepinephrine, dopamine, epinephrine, dobutamine, vancomycin, Bactrim, Gentamicin, Amikacin, Colistin, Amphotericin B	
89	Anran Dai (2023)	Retrospective	China	All	Incidence	Perioperative Monitoring	Cardiac and cardiac surgery- related	In hospital	265	Training cohort, Test cohort	4		0.862
90	Jicheng Jiang (2023)	Retrospective	China	Adults(> 18 years)	Incidence	Perioperative Monitoring	Cardiac and cardiac surgery- related	In hospital	2310	Training cohort	6	Preoperative Scr(mg/dl),Assisted ventilation time(hr),Lipid-lowering agents,Intravenous nitroglycerin,Hyperlipidemia,NYHA H-V,ACC,IABP,CCS II-IV,CPB,Intraoperative BPT,CA,β -blockers,ACEi/ARB,CABG+valve/other surgery,CCF	0.860
91	Yang Zhou (2023)	Retrospective	China	Adults(> 18 years)	Incidence	ICU Real-time Alert	Respiratory/ARDS- related	Public databases	1085	Training cohort, Internal validation cohort	4	creatinine, BUN, urine output, Pao2/Fio2, bilirubin, hemoglobin, potassium, lactate, positive end-expiratory pressure (PEEP), hypertension, congestive heart failure, and diabetes	0.860
92	Christopher T Ryan (2023)	Retrospective	USA	All	Incidence	Perioperative Monitoring	Cardiac and cardiac surgery- related	Public databases	4267	Test cohort	1		0.870
93	Xiao-Qin Luo (2023)	Prospective	China	Pediatric	Incidence	Perioperative Monitoring	Cardiac and cardiac surgery- related	In hospital	3863	Training cohort, External validation cohort	7	Baseline creatinine,Body length,Preoperative length of stay,Cyanotic heart disease,Platelets,Baseline eGFR,Potassium,Age,Total bilirubin,Calciun,Weigh,ASA Physical Status,Blood urea nitrogen,AST,Albumin,Hemoglobin,Sodium,Chloride,Previous cardiac surgery,ALT,RDW,lodinated contrast media,Pulmonary infection,Diuretics,Preoperative intensive care,	0.912
94	Xunliang Li (2023)	Retrospective	China	Adults(> 18 years)	Mortality	Early Risk Stratification	Sepsis/infection- related	Public databases	8129	Internal validation cohort	6	SOFA,Respiratory rate,SAPS II,Age,Cerebrovascular disease,Body temperature ,Urine output,Serum sodium,Serum chloride,Weight,INR,Hemoglobin,Heart rate,Cancer,Serum creatinine,Liver disease ,PTT,Platelets ,Anion gap ,BUN,	0.794
95	Amir Kamel Rahimi (2023)	Retrospective	Australia	Adults(> 18 years)	Incidence	Early Risk Stratification	Other/mixed	Public databases	31586	Test cohort	4	creatinine min,eGFR,creatinine max,Prothrombin time,invasive ventilation,potassium_max,platelets min,congestive heart failure,sodium max,hematocrit min,hemoglobin min,weight_max,chloride max,diabetes Type2,calcium min,age,temperature_max,oxygen saturation min,chronic kidney disease,white blood count max,white blood count min,diastolic blood pressure max,hemoglobin_max,aniongap max,urine output,female,supplemental Oxygen,heart rate max,blood urine nitrogen min,oxygen saturation max,bicarbonate min,calcium max,hypertension,respiratory rate max,systolic blood pressure max,aniongap min,myocardial infarction,WHITE,bicarbonate max,peripheral_vascular_disease	0.760
96	Hongshan	Retrospec	China	Adults(>	Mortality	Early Risk	Sepsis/infection- relat	Public	16154	Training cohort,	11	urine output, maximum blood urea nitrogen (BUN), rate of injection of norepinephrine, maximum	0.830

	Zhou (2023)	tive		18 years)		Stratification	ed	databases		Internal validation cohort		anion gap, maximum creatinine, maximum red blood cell volume distribution width (RDW), minimum international normalized ratio (INR), maximum heart rate, maximum temperature, maximum respiratory rate, minimum fraction of inspired O2 (FiO2), minimum creatinine, minimum GCS score, and diagnosis of diabetes and stroke	
97	Rui Fan (2023)	Retrospective	China	Adults(> 18 years)	Incidence	Perioperative Monitoring	Cardiac and cardiac surgery- related	In hospital	778	Internal validation cohort	3	sST2, NT-proBNP, H-FABP, ALT, AST, LDH, alkaline phosphatase, UA, total cholesterol, triglyceride, HDL, LDL, apolipoprotein A1, apolipoprotein B, lipoprotein(a), serum albumin, total bilirubin, TBA, PCO2, PO2, intubated PO2/FiO2 ratio, serum sodium, serum potassium, serum calcium, serum magnesium, lactic acid, HCO3-, WBC count, lymphocyte count, monocyte count, neutrophil count, RBC count, hemoglobin, hematocrit, RDW, platelet count, MPV, PDW, PT, INR, APTT, fibrinogen, D-dimer	0.856
98	Shuxing Wei (2023)	Retrospective	China	Adults(> 18 years)	Incidence	ICU Real-time Alert	Respiratory/ARDS-related	Public databases	1581	Training cohort, Internal validation cohort	12	CreatininePO2SepsisBUNLactateALBUoSpo2WBCTBILRDWASTDiabetesDBPChloridionHRGlucoseBEPlateletsUTI	0.865
99	Tianchen Jia (2023)	Retrospective	China	Adults(> 18 years)	Incidence	Perioperative Monitoring	Cardiac and cardiac surgery- related	In hospital	4831	Test cohort, External validation cohort	4		0.882
100	Jiaqi Lu (2023)	Retrospective	China	Adults(> 18 years)	Incidence	Early Risk Stratification	Other/mixed	In hospital	446	Training cohort, Internal validation cohort	1	hypernatremia, heart failure, sepsis, APACHE II score, initial creatinine, initial BE (base excess)	0.795
101	Yun Yan (2023)	Retrospective	China	Adults(> 18 years)	Incidence	Perioperative Monitoring	Cardiac and cardiac surgery- related	In hospital	3392	Training cohort, Test cohort	2	age, gender, hemoglobin, pulmonary hypertension, arrhythmia, hypertension, duration of CPB, the volume of autologous blood transfusion, pre-operative diagnosis, the highest level of lactate during the operation, albumin, BMI, vasoactive inotropic score ,ejection fraction	0.730
102	Changwei Wu (2023)	Retrospective	China	Adults(> 18 years)	Mortality	Early Risk Stratification	Other/mixed	In hospital	137084	Training cohort, Internal validation cohort, External validation cohort	1		0.951
103	Tingting Fan (2023)	Retrospective	China	All	Incidence	Early Risk Stratification	Other/mixed	Public databases	1322	Training cohort, Internal validation cohort	8	BUN,Urune output,Weight admit,Age,PLT,Infusion volume,BG	0.835
104	Lijuan	Retrospec	USA	Adults(>	Incidence	Early Risk	Other/mixed	Public	48780	Test cohort	1	age, gender, ethnicity, admission type, admission location, first care unit, time from hospital to	0.840

	Wu (2023)	tive		18 years)		Stratification		databases				ICU admission, heart rate, blood pressure, respiratory rate, temperature, height, weight, albumin, creatinine, white blood cell count, blood urea nitrogen, vasopressor, ventilation, expressed as cumulative exposure frequency,urine volume after ICU admission	
105	Yefeng Tong (2023)	Retrospective	China	Adults(> 18 years)	Incidence	Perioperative Monitoring	Cardiac and cardiac surgery- related	In hospital	1264	Training cohort, Test cohort	6	CRC,UOPH,AGE,CPBt,SB,SCR,RBC,PBL,ALB,HLB,IVST,PSBP,INR,CPS,AD,CCB,EA,HTN, ASA,EM	0.998
106	Suhua She (2023)	Retrospective	China	Adults(> 18 years)	Incidence	Early Risk Stratification	Cerebrovascular- related	Public databases	1213	Training cohort, Internal validation cohort	6	Platelet,Creatinine,Vancomycin,Hemoglobin,Hematocrit,HR,BUN,RBC,Glucose,DBP,RDW,Chloride,SBP,age,BT,HBC,Potassium,Sodium,Sp02,GCs	1.000
107	Wenxing Peng (2023)	Retrospective	China	Geriatric	Incidence	Perioperative Monitoring	Cardiac and cardiac surgery- related	In hospital	2155	Training cohort, Internal validation cohort	1	T2DM, diabetes mellitus type intraoperative use of intra-aortic ballon pump (IABP), cardiopulmonary bypass (CPB), epinephrine, isoprenaline	0.754
108	Jia Tian (2023)	Retrospective	China	Adults(> 18 years)	Incidence	Specialty-specific	Liver disease- related	Public databases	950	Training cohort, Internal validation cohort	5	Mechanical ventilation, vasopressor, international normalized ratio (INR), bilirubin, Charlson comorbidity index (CCI), prothrombin time (PT), estimated glomerular filtration rate (EGFR), partial thromboplastin time (PTT), and heart rate	0.833
109	Yixin Tian (2023)	Retrospective	China	Adults(> 18 years)	Incidence	Early Risk Stratification	Cerebrovascular- related	In hospital	9649	Training cohort, Internal validation cohort, External validation cohort	1	sex, systolic blood pressure, diabetes, Glasgow coma scale, mannitol infusion,serum creatinine, albumin, uric acid, neutrophils-to-lymphocyte ratio	0.821
110	Pan Pan (2023)	Retrospective	China	Adults(> 18 years)	Incidence	Early Risk Stratification	Other/mixed	Public databases	2260		4	Scr,platelet,k,age,WBC,sysbp,bicabonate,gender,bun,height	0.892
111	Yue Hu (2022)	Retrospective	China	Adults(> 18 years)	Incidence	Specialty-specific	Nephrotoxic/drug- related	In hospital	1073	Training cohort, Internal validation cohort	1	subtypes of ACS, age >75 years, multivessel coronary artery disease, hyperuricemia, LDL-C, TyG index, eGFR	0.811
112	Xi Peng (2022)	Retrospective	China	Adults(> 18 years)	Incidence	Specialty-specific	Cardiac and cardiac surgery- related	Public databases	18329	Training cohort, Internal validation cohort, External validation cohort	13	CRE,BUN,PO2,UO,Calcium,Temperature,Lactate,HR,WBC,Age,Weight,PCO2,MAP,RBC,Hematocrit,PLT,CK,Glucose,	0.816
113	Jin Wang (2022)	Retrospective	China	All	Incidence	Perioperative Monitoring	Cardiac and cardiac surgery- related	In hospital	211		1	Age,Body mass index (kg·m−2),Renal dysfunction,Atrial arrhythmia,Central venous pressure,Cardiac index	0.780

114	Qi Xin (2022)	Retrospective	China	Adults(> 18 years)	Incidence	Early Risk Stratification	Sepsis/infection- related	In hospital	1051	Training cohort, Internal validation cohort	1	cardiovascular disease, WBC, MAP, PLT, PCT, PTA, TT	0.888
115	Yirui Hu (2022)	Retrospective	USA	Adults(> 18 years)	Incidence	Early Risk Stratification	Other/mixed	In hospital	209330	Training cohort	2	Serum Creatinine,eGFR,Mean arterial pressure,CKD,Diuretic use,White Blood Cell,Prothrombin time (INR),Serum sodium,Platelet count,Hemoglobin,Antidiabetic meds,Body Mass Index,Thrombocytopenia,Hypercalcemia,Respiratory failure	0.870
116	Yuxi Li (2022)	Retrospective	China	Adults(> 18 years)	Incidence	Specialty-specific	Nephrotoxic/drug- related	In hospital	2560	Test cohort	7	age, sex, anemia, diabetes, heart failure, hypotension, myocardial infarction history, hypercholesterolemia, hypertension, glomerular filtration rate (GFR), high-density lipoprotein cholesterol (HDL-C), pre-procedural peak creatinine, left ventricular ejection fraction (LVEF), urgent percutaneous coronary intervention (PCI), intra-aortic balloon pump (IABP) use, contrast volume	0.820
117	Xian Zeng (2022)	Retrospective	China	Pediatric	Incidence	Perioperative Monitoring	Cardiac and cardiac surgery- related	In hospital	3386	Internal validation cohort	7	C-reactive protein ,Autologous blood transfusion ,Mechanical ventilation time,Lymphocytes,Platelet count,Age,FFP transfusion,Aortic cross-clamp time , Cardiopulmonary bypass time,Neutrophils,Hemoglobin,Preoperative LOs,Monocytes, Platelet distribution width,Operation time,Height,Pressure of oxygen,Anion gap,Absolute neutrophil count,Eosinophils,	0.887
118	Sunho Ko (2022)	Retrospective	Korea	Geriatric	Incidence	Perioperative Monitoring	Trauma/non- cardiac surgery- related	In hospital	5757	Training cohort, External validation cohort	1	higher preoperative levels of creatinine in serum, the use of general anesthesia, male sex, a higher ASA class (> 3), use of a renin–angiotensin–aldosterone system inhibitor, ,no use of tranexamic acid	0.890
119	Qiuchong Chen (2022)	Retrospective	China	Geriatric	Incidence	Perioperative Monitoring	Trauma/non- cardiac surgery- related	In hospital	1000	Training cohort, Test cohort	9	age, BMI, diabetes, hypertension, hypoproteinemia, duration of low mean arterial pressure, anemia, transfusion, ASA, mean arterial pressure	1.000
120	Chang Hu (2022)	Retrospective	China	Pediatric	Mortality	Early Risk Stratification	Other/mixed	Public databases	22360	Internal validation cohort	7	Age,Body weight,Hypertension,Diabetes,Congestive heart failure,Cerebrovascular disease,Liver disease,Tumor,Heart rate,Systolic blood pressure,Mean arterial pressure,Respiratory rate,Body temperature,SpO2,White blood cell,Anion gap,Bicarbonate,Blood urea nitrogen,Chloride,Creatinine,Sodium,International normalized ratio,Prothrombin time,Partial thromboplastin time,Antibiotics,Mechanical ventilation,Vasopressors,Urine output,GCS	0.890
121	Zhi Xiang Du (2022)	Retrospective	China	Adults(> 18 years)	Incidence	Early Risk Stratification	Nephrotoxic/drug- related	In hospital	315		1	microalbuminuria (UM), hematuria (URBC), cystatin C (CYS-C), albumin (ALB), CA-125	0.967
122	Jurij	Retrospec	Germany	All	Incidence	Perioperative	Cardiac and cardiac	In hospital	7507		2	2nd AeGFR preop. /6-12h [%],eGFR <12 h postop. [mL/min/1.73m2],Age [years],Drainage output	0.880

	Matija Kalisnik (2022)	tive				Monitoring	surgery-related					(0-12h) [mL],eGFR ICU arrival [mL/min/1.73m2],1st AeGFR preop./ICU arrival [%],Hb postop. (0-6h) [g/dL],Ejection Fraction preop. [%],Hb preop. [g/dL],Cross clamp time [min.],eGFR preop. [mL/min/1.73m2],Transfusion of pRBCs intraop. [number of units],Revision,History of atrial fibrillation,Chronic obstructive lung disease,Administration of diuretics preop.,Previous cardiac surgery,IABP postop.,Peripheral vascular disease,Infection preop.,Emergent Op,	
123	Suru Yue (2022)	Retrospective	China	Adults(> 18 years)	Incidence	Early Risk Stratification	Sepsis/infection-related	Public databases	2415	Training cohort, Internal validation cohort	1	gender, ethnicity, congestive heart failure, diabetes, obesity, Simplified Acute Physiology Score II (SAPS II), angiotensin-converting enzyme inhibitors (ACEI) or angiotensin receptor blockers (ARBs), bilirubin, creatinine, blood urea nitrogen (BUN), mechanical ventilation	0.760
124	Qi Xin (2022)	Retrospective	China	Geriatric	Incidence	Early Risk Stratification	Sepsis/infection-related	In hospital	849	Training cohort, Internal validation cohort	1	MAP（0.979,0.969–0.989）,AGR（0.277,0.161–0.477）,PTA（0.977,0.968–0.986）,PCT（1.019,1.009–1.028）,Cr（1.012,1.008–1.016）和 PLT（0.994,0.992–0.996）	0.858
125	Sasha Pierre-Paul (2022)	Retrospective	USA	Adults(> 18 years)	Incidence	Early Risk Stratification	Other/mixed	In hospital	200	Training cohort, Test cohort	1	Sex,age,Vanco,Ampho,NSAID,ACEI/ARB,Acyclovir,AMG,DM,Infection,HTN,HLD,IIIicit,Cigarette,Surgery,BKF,BMI,AKI	0.860
126	Okke Nikkinen (2022)	Retrospective	Finland	Adults(> 18 years)	Incidence	Perioperative Monitoring	Trauma/non-cardiac surgery-related	In hospital	648		3	UOP, postop. day 1, ml/kg/h, UOP, operation day, ml/kg/h, UOP, postop. day 1, ml/kg/h, UOP, postop. day 1, ml/kg/h, Type of surgery, operation codes, UOP, operation day, ml/kg/h, UOP, postop. day 1, ml/kg/h, Infused fluids, postop. day 1, ml, Age, UOP, intraop., ml/kg/h, Infused fluids, intraop., ml/h, BMI, grouped, ASA, Sex, BMI, eGFR, preop., sCr preop., Infused fluids, operation day, after surgery, ml, UOP, operation day, after surgery, ml, Emergency hip arthroplasty, Infused fluids, postop. day 1, ml/kg/h, UOP, operation day, ml, UOP, intraop, ml, Duration of operation + recovery room, Infused fluids, intraop., ml/kg/h, Contrast medium within 5 yr., ACE inhibitor at home, Sartan at home, Diabetes, Hypertension.	0.980
127	Luming Zhang (2022)	Retrospective	China	Adults(> 18 years)	Incidence	Early Risk Stratification	Sepsis/infection-related	Public databases	45895	Training cohort	5	[age, anion gap, creatinine, hemoglobin, mean cell hemoglobin con centration (MCHC), phosphate, international normalized ratio (INR), platelet, total bilirubin, potassium, pH,lactate, PaO2, PaCO2, heart rate, temperature and SpO2	0.995
128	Xiao-Qin Luo (2022)	Retrospective	China	Adults(> 18 years)	Mortality	Early Risk Stratification	Sepsis/infection-related	Public databases	15603	Internal validation cohort, External validation cohort	3	Age,Mechanical ventilation,Blood urea nitrogen,GCS score,Urine output,Respiratory rate,Hours from admission,Platelets,Lactate,Partial thromboplastin time,Serum total bilirubin,INR,Body temperature,Baseline serum creatinine,Vasopressors,Arterial pH,Heart rate,Pa02,Ethnicity_Other/Unknown,White blood cells,Serum bicarbonate,Hemoglobin,Oxygen saturation,Serum albumin,Serum sodium,Diastolic blood pressure,Systolic blood	0.984

												pressure,PaCO12,Serum chloride,Sex,Ethnicity_Black,Serum creatinine,RRT,Serum potassium,Ethnicity_Hispanic,Loop diuretics,Ethnicity_Asian,	
129	Kang Liu (2022)	Retrospective	China	All	Incidence	Early Risk Stratification	Other/mixed	In hospital	76957		1		0.780
130	Kai Ma (2022)	Retrospective	China	Adults(> 18 years)	Incidence	Specialty-specific	Nephrotoxic/drug- related	In hospital	1156	Training cohort, Internal validation cohort	1	age >75 years, diabetes mellitus, LVEF, FAR, hsCRP, lymphocyte count	0.835
131	Yu Zhang (2022)	Retrospective	China	Adults(> 18 years)	Incidence	Specialty-specific	Cardiac and cardiac surgery- related	In hospital	1731	Training cohort, Internal validation cohort	1	age, emergency surgery, abnormal BCr, CKD, nephrotoxic drugs, diuretic use, SOFA score	0.725
132	Congjie Wang (2022)	Retrospective	China	Adults(> 18 years)	Incidence	Early Risk Stratification	Respiratory/ARDS- related	In hospital	480	Training cohort, Internal validation cohort	1	coronary artery disease (CAD), arterial oxygen saturation (SaO2), procalcitonin (PCT), C-reactive protein (CRP), glomerular filtration rate (GFR)	0.831
133	Dongliang Yang (2022)	Retrospective	China	Adults(> 18 years)	Incidence	ICU Real-time Alert	Severe acute pancreatitis (SAP)	In hospital	996	Training cohort, Internal validation cohort	1	intra-abdominal pressure (IAP), C-reactive protein (CRP), cystatin C (CysC)	1.000
134	Danfeng Zha (2022)	Retrospective	China	All	Mortality	ICU Real-time Alert	Other/mixed	Public databases	270	Training cohort, Test cohort	2	SAPS-II,Age,SOFA,Respiratory,MAP,MELD,Peripheral vascular,Diabetes uncomplicated,Coagulopathy,Cardiac arrhythmias,Liver disease	0.870
135	Charat Thongprayoon (2022)	Retrospective	USA	Adults(> 18 years)	Incidence	Perioperative Monitoring	Cardiac and cardiac surgery- related	In hospital	13158	Test cohort	6	Baseline.eGFR,Cardiac.surgery.type,Coagulopathy,Peripheral.vascular.dis orders , Hypertension.with.complications,Insulin,RVSP,BUN,BMI,SBPHemoglobin,Arrhythmia,Male,Inotropes,Serum.albumin , Antiarrhythmic.drugs , Serum.sodium , Serum.lactate,Age , p02,	0.790
136	Xiang Yu (2022)	Retrospective	China	Adults(> 18 years)	Incidence	Specialty-specific	Hematologic/malignancy- related	In hospital	248	Training cohort, Test cohort	9	age,nivolumab,NSAIDS, antibiotics, PPI, ACEI/ARB, diuretics, and chemotherapeutic agents,ALb, Hb, eGFR, and D-dimer,hepatobiliary cancer, genitourinary cancer, gastrointestinal tract cancer, and lung cancer	0.832
137	Ira S Hofer (2022)	Retrospective	USA	Adults(> 18 years)	Incidence/ Mortality	Perioperative Monitoring	Trauma/non- cardiac surgery- related	In hospital	101070		1	(all sets)	0.943
138	Justin Y	Retrospec	USA	All	Incidence	Early Risk	Respiratory/ARDS- r	In hospital	5868	Internal validation	1	egfr,ldh,ast,alt,crp,ddimer,wbc,lymph,albumin	0.965

	Lu (2022)	tive				Stratification	elated			cohort			
139	Yi Yang (2022)	Retrospective	China	Adults(> 18 years)	Incidence	ICU Real-time Alert	Severe acute pancreatitis (SAP)	In hospital	424	Training cohort, Test cohort	5	Scr, CRP, PLR, NLR, NAR, CysC	0.913
140	Xiaohong Zhang (2022)	Retrospective	China	All	Incidence	Early Risk Stratification	Cerebrovascular-related	In hospital	3434	Internal validation cohort, External validation cohort	5	Creatitine,Age,Hemoglobin,White blood cell count,Length of stay in ICU,Bicarbonate,Blood urea nitrogen,Sodium,Albumin,Platelet count,Mechanical ventilation,Potassiu,NSAIDS,Diuretics,Hypertension,Aminoglycosides,Vasoactive drugs,Sex,Diabetes,CRRTColloid bolus,Mannitol	0.880
141	Yisong Cheng (2022)	Retrospective	China	All	Incidence	ICU Real-time Alert	Severe acute pancreatitis (SAP)	In hospital	488	Training cohort	4	Platelet, CRP,Procalcitonin,Ln BNP,Uric acid,Creatinine,APTT,Thrombin time	0.828
142	Suru Yue (2022)	Retrospective	China	All	Incidence	Early Risk Stratification	Sepsis/infection-related	Public databases	3176		7	Urine output,Mechanical ventilation,BMI,eGFR,Creatinine min,PTT max,BUN min,TempC_min,Cardiac arrhythmias,Bilirubin_min,Age,Potassium max,PTT_min,TempC_max,Chloride max,Chloride min,Platelet min,PT max,SysBP_min,Vasopressor,Creatinine_max,Sodium max,DiasBP min,Platelet max,Sodium min,Bilirubin max,Lactate max,BUN_max,Anion gap_max,Lactate_min,Anion gap_min,PT min,INR max,Liver disease,INR min,	0.817
143	Toshiki Kuno (2022)	Retrospective	Japanese	All	Incidence	Specialty-specific	Cardiac and cardiac surgery-related	In hospital	19222	Training cohort, Test cohort	2	STEMI,eGFR,Age,IABP,Preprocedural Hb,HF at admission,UA/NSTEMI,DM,CS,CPA,Prior Stroke,Prior HF,	0.797
144	Hsin-Hsiung Chang (2022)	Retrospective	China	Adults(> 18 years)	Mortality	ICU Real-time Alert	Other/mixed	Public databases	3412	Training cohort, Test cohort	4	CreatininePlateletFiO2Anion gapGCSAgeMAPVasopressorRRHCO3	0.823
145	Teddy Lazebnik (2022)	Retrospective	UK	Adults(> 18 years)	Incidence	Perioperative Monitoring	Other/mixed	In hospital	723	Training cohort	1	tumor size (in centimeters), RENAL score, patient age (in years), baseline hemoglobin (in g/dL), ischemia time (in minutes), patient weight (in kilograms), patient height (in centimeters), and baseline preoperative creatinine (in mg/dL)	0.750
146	Dabei Cai (2022)	Retrospective	China	Adults(> 18 years)	Incidence	Early Risk Stratification	Hypoperfusion/shock-related	Public databases	3882	Training cohort, Test cohort, External validation cohort	6	Creatinine,Activated partial thromboplastin time,Glucose,Platelet ,Count	1.000
147	Huan Jing	Retrospec	China	Adults(>	Incidence	Perioperative	Cardiac and cardiac	In hospital	1082	Training cohort,	1	gender (male), age, hypertension, CPB duration, intraoperative RBC transfusion, intraoperative 5%	0.725

	(2022)	tive		18 years)		Monitoring	surgery-related			Internal validation cohort		sodium bicarbonate solution (SBS), urine output	
148	Li Xinsai (2022)	Retrospective	China	All	Incidence	Perioperative Monitoring	Cardiac and cardiac surgery-related	In hospital	456	Training cohort	5	baseline SCR, BUN, UA, MVT, LOS in ICU, WBC, PLT, D dimer, plasma transfusion, CPB duration	0.760
149	Takaya Okawa (2022)	Retrospective	Japanese	All	Incidence	Specialty-specific	Nephrotoxic/drug-related	In hospital	1240	Internal validation cohort	1	Male sex,Age,BSA,Serum creatinine,Serum albumin,History of DM,History of CVD,Maximum daily cisplatin dose	0.770
150	Azar Ejmalian (2022)	Retrospective	Iran	All	Incidence/ Mortality	Perioperative Monitoring	Cardiac and cardiac surgery-related	In hospital	1435	Training cohort	7	Cr,Crystalloid,Surgical Setting,Alb,CCB,Diuretics,NSAIDs,CPB time,Colloid	0.810
151	Yuhui Zhang (2022)	Retrospective	China	Adults(> 18 years)	Incidence	Perioperative Monitoring	Cardiac and cardiac surgery-related	In hospital	224		1	iliac artery involvement, creatinine, D-dimer, autotransfusion, platelet-rich plasma reinfusion, drainage, mechanical ventilation	0.848
152	Mei Wang (2022)	Retrospective	China	All	Mortality	ICU Real-time Alert	Hypoperfusion/shock-related	In hospital	1836	Training cohort, Internal validation cohort	1	Hepatorenal syndrome, shock, central nervous system failure, Charlson comorbidity index (≥2 points), mechanical ventilation, renal function at discharge	0.878
153	Hang Zhang (2022)	Retrospective	China	Adults(> 18 years)	Incidence	Perioperative Monitoring	Cardiac and cardiac surgery-related	In hospital	1457	Internal validation cohort	3	CVP_T4, hemoglobin,serum potassium,ultrafiltration volume,LDH,urine output,neutrophil to lymphocyte ratio [NLR],CVP_T3,perfusion flow_T3,Scr	0.881
154	Wenpeng Gao (2022)	Retrospective	China	All	Incidence	Early Risk Stratification	Other/mixed	Public databases	30020	Test cohort	5	The length of stay,levels of serum creatinine, diuretic, albumin, bicarbonate, sepsis, chloride, white blood cell count (WBC), blood urea nitrogen (BUN) and glucose (GLU)	0.923
155	Jun Liu (2022)	Retrospective	China	Adults(> 18 years)	Incidence	Perioperative Monitoring	Trauma/non-cardiac surgery-related	Public databases	1596	Training cohort, Internal validation cohort	10	Minimum BUN (mEq/L),Blood transfusion,Time in iCU (days),Minimum RBC (10^12/L),Minimum RDW (%),Time to surgery (hours),RDW (%),Mean RDW (%),Minimum Hemoglobin (g/dL),Minimum SCr (mg/dL),WBC (10^9/L),SCr (mg/dL),Mean AG (mmolL),Maximum WBC (10^9/L),Minimum Calcium (mg/dL),Maximum Calcium (mg/dL),BUN (mEq/L),Calcium (mg/dL),Glucose (mg/dL),Minimum Sodium (mEq/L)	0.930
156	Yelena Petrosyan (2022)	Retrospective	Canada	Adults(> 18 years)	Incidence	Perioperative Monitoring	Cardiac and cardiac surgery-related	In hospital	6522	Training cohort	3	CARE score , BMI, hypertension, atrial fibrillation, NYHA Class 3, left ventricle ejection fraction < 35%, anemia, emergent operative status, redo sternotomy, combined CABG/valve surgery, former smoker, and preoperative intra-aortic balloon pump use	0.750

157	Jiaqi Wang (2022)	Retrospective	China	Adults(> 18 years)	Incidence	Perioperative Monitoring	Trauma/non- cardiac surgery- related	In hospital	3314		1	sex, ASA physical status, coronary heart disease, congestive heart failure, hypertension, the use of RAAS inhibitors,the duration of surgery, proteinuria, NT-proBNP concentrations . LVEF levels	0.800
158	Hui Zhao (2022)	Retrospective	China	Adults(> 18 years)	Incidence	Early Risk Stratification	Hypoperfusion/shock - related	In hospital	452	Training cohort, External validation cohort	1	ejection fraction of no more than 40%, age > 72 years, creatinine > 102.7 mmol/L, RDW > 13.15, middistal segment culprit lesions	0.731
159	Wen Tao Liu (2022)	Retrospective	China	Adults(> 18 years)	Incidence	Specialty-specific	Hypoperfusion/shock - related	Public databases	2678		5	Age(year),Male (%),Height (cm),Weight (kg),Respiratory rate(bpm),Body temperature(C),Heart rate (bpm),Systolic bloodpressure (mmHg),Diastolic bloodpressure (mmHg),SOFA score,Ventilation (%) ,Diabetes (%), CHD(%) ,Hypertension (%) ,Atrial flutter or atrialfbrillation (%,)COPD(%),Scr (mg/dL),eGFR(mL/min/1.73 m2),Urea nitrogen(mg/dL),WBC(K/ML),Hemoglobin (g/dL),Platelet(K/uL),PT (S),INR,PH,PaO2 (mmHg),PaCO2(mmHg),Blood lactic acid(mmol/L),Serum bicarbonate(mEq/L),Serum potassium(mEq/L),Serum sodium(mEq/L),Serum calcium(mg/dL),Serum magnesium(mg/dL),Serum phosphate (mg/dL),Blood glucose(mg/dL),RAS inhibitor(%),Diuretics (%),Digoxin(%),B-receptor blocker(%),	0.960
160	Yuhei Goriki (2021)	Retrospective	Japan	All	Incidence	Early Risk Stratification	Hypoperfusion/shock - related	In hospital	908	Training cohort, Internal validation cohort	1	BS (blood sugar) ≥ 200 mg/dL,hsTnI (high-sensitivity troponin I) > 1.6 ng/mL,albumin ≤ 3.5 mg/dL,eGFR < 45 mL/min/1.73 m²	0.755
161	Jianchao Ma (2021)	Retrospective	China	Adults(> 18 years)	Incidence	Early Risk Stratification	Sepsis/infection- related	In hospital	358	Training cohort, Internal validation cohort	1	APACHE II score, serum creatinine, vasopressor use, sCysC, uNAG	0.831
162	Kaidi Gong (2021)	Retrospective	China	Adults(> 18 years)	Incidence	Early Risk Stratification	Other/mixed	Public databases	12447	Test cohort	8	diuretics,creatinine_slope,eGFR last,creatinine _last,mechanical_ventilation,tempc_slope,sysbp_slope,vancomycin,ptt_last,crystalloids_ivf,eGFR _slope,ptt_slope,platelet_last,lactate_last,age,rl-ivf,phosphate_slope,potassium_last,tempc_last,nsaid	0.781
163	Yihan Zhang (2021)	Retrospective	China	Adults(> 18 years)	Incidence	Perioperative Monitoring	Liver disease- related	In hospital	975	Internal validation cohort, External validation cohort	5	IBIL (umol/L)UO (ml/(kg-h))Time under GA (min)PLT (10^9/L)Steatosis >1Pre-op LOS (d)EBL (ml)ALB (g/L)Bicarbonate (ml)Colloid (ml)Pre-op HECryo (U)ALT (U/L)Pre-op HM	0.760
164	Zhimeng Chen	Retrospective	China	Adults(> 18 years)	Incidence	Perioperative Monitoring	Liver disease- related	Public databases	46385	Test cohort	6	urine, glucose, creatinine, sodium, blood urea nitrogen (BUN), lactate, prothrombin time (PT), partial thromboplastin time (PTT), non-steroidal anti-inflammatory drugs (NSAIDs), agents acting	0.858

	(2021)											on the renin-angiotensin system, lipid-lowering medication, congestive heart failure, peripheral vascular, hypertension, diabetes, diuretics, drugs used in diabetes, antithrombotic agents, antihypertensives, contrast media, anion gap, potassium, bilirubin, white blood count (WBC), chloride, blood oxygen saturation value, body temperature, albumin, bicarbonate, systolic blood pressure, respiration rate, mean arterial blood pressure, heart rate, diastolic blood pressure, platelet, hemoglobin, hematocrit, international normalized ratio (INR), coronary artery disease (CAD), liver disease, myocardial infarction, cirrhosis, jaundice, sleep apnea, urinary tract infection, gender, age, ethnicity, mass, height, BMI value	
165	Yunlu Zhang (2021)	Retrospective	China	All	Incidence	Perioperative Monitoring	Liver disease-related	In hospital	7968	External validation cohort	2	Phosphorus,SCr,eGFR,Potassium,BMI,Hemoglobin,Glucose,TBiL,LeukocyteAge,AST,Sodium,Treatment,Anion gap,SUA	0.850
166	Lauren A Scanlon (2021)	Retrospective	UK	Adults(>18 years)	Incidence	Specialty-specific	Hematologic/malignancy-related	In hospital	597403	Training cohort, Test cohort	1	Current CREA/median over the past year,Total protein (TP) from blood test,Current CREA/minimum over the past 7 days,Current CREA level—mean over the past year,Platelet count (PLT) from blood test,Hematocrit (HCT) from blood test,Calcium (CA) from blood test,Lymphocytes (LYMPH) from blood test,Current urea—mean over past year,Lactate dehydrogenase (LDH) from blood test,Red cell count (RBC) from blood test,Minimum CREA over the past 7 days,White cell count (WBC) from blood test,Median CREA over the past year,Minimum CREA over the past 2 days,Current CREA—CREA 10 days ago,Creatinine (CREA) from blood test,Current CREA—CREA 20 days ago,Abnormal albumin (ALB) categorical variable,	0.881
167	Haofan Huang (2021)	Retrospective	China	All	Mortality	Early Risk Stratification	Other/mixed	In hospital	4558	Training cohort, External validation cohort	1	CMO,DNR,SAPSII,Urine output,SOFA,Anion gap,BUN,Lactate,SYSBP,Total CO2	0.910
168	Daniela Ponce (2021)	Prospective	Latin American 12 countries	All	Mortality	Early Risk Stratification	Respiratory/ARDS-related	In hospital	870	Training cohort, Internal validation cohort	3	MV, higher age, vasopressors, WBC,severe condition at admission,COCID Infection at Hospital ,AST,Renal Replacement,moderate condition at admission,AKI Etiology Sepsis	0.894
169	Jing-Cheng Peng (2021)	Retrospective	China	Geriatric	Mortality	Early Risk Stratification	Other/mixed	In hospital	842	Test cohort, Internal validation cohort	1	AKI type　AKI,Renal function at discharge。Hypotension,AMI,Liver disease,The number of organ failure,Proteinuria	0.907

170	Wang Xin (2021)	Retrospective	China	Adults(> 18 years)	Incidence	Perioperative Monitoring	Liver disease-related	In hospital	272	Training cohort	2	operation time, intraoperative hypotension time, AST peak, post-transplant infection, post-transplant shock	0.755
171	Yeonhee Lee (2021)	Retrospective	South Korea	Adults(> 18 years)	Incidence	Perioperative Monitoring	Hematologic/malignancy-related	In hospital	4104	Training cohort	5	Radical vs. Partial nephrectomy,Male,Tumor size,Total operation time,eGFR,Age,Creatinine , Hypertension , Hemoglobin , Estimated blood loss	0.810
172	Tingting Fan (2021)	Retrospective	China	All	Incidence	Early Risk Stratification	Other/mixed	Public databases	760	Training cohort, Internal validation cohort	1	T2DM,microangiopathy,preexisting CHF,history of hypertension,RR,urine output,GCS,DBP	0.747
173	Amanda C Filiberto (2021)	Retrospective	USA	Adults(> 18 years)	Incidence	Perioperative Monitoring	Cardiac and cardiac surgery-related	In hospital	1631		1	Type of surgery,Attending surgeon,Urine hemoglobin 0-7 days prior to surgery (minimum),Scheduled surgery room ,Serum urea nitrogen and creatinine ratio 0-7 days prior to surgery (maximum) ,Serum creatinine 0-7 days prior to surgery (maximum),FiO2 (max) ,Age,Serum hemoglobin 0-7 days prior to surgery (average),FiO2 above 48.9 (percentage),Serum glucose 0-7 days prior to surgery (count),Peek inspiratory pressure (average),Diastolic BP 48-78 (count),Heart rate (average),Serum hemoglobin 8-365 days prior to surgery (minimum),Serum creatinine 0-7 days prior to surgery (average),Charlson comorbidity Index,MAC (average),Systolic BP 91-139 (count),Systolic BP (maximum),Serum creatinine 0-7 days prior to surgery (minimum), Serum glucose 0-7 days prior to surgery (maximum),FiO2 above 48.9% (count),Serum calcium 0-7 days prior to surgery (minimum),End tidal CO2 22-28 (percentage),Hemoglobin 0-7 days prior to surgery (count),BUN 0-7 days prior to surgery (maximum),Systolic BP (minimum),End tidal CO2 28-31.0 (count),MAC 0.33-0.475 (percentage),MAC 0.04-0.33 (percentage),WBC 0-7 days prior to surgery (maximum),Diastolic BP 55.5-70.5 (count),Serum glucose 0-7 days prior to surgery (average),Respiratory rate 7-15 (count),MAC 0.04-0.33 (count),MAC 0.33-0.91 (count),Systolic BP 103-127 (count),Serum calcium 0-7 days prior to surgery (average),Systolic BP 91-103 (count),Diastolic BP 48- 55.5 (count),Diastolic BP (minimum),Systolic BP 67-91 (count),MAC 0.33-0.475 (count),Systolic BP 127.0-139 (count),Heart rate 95-112 (count),Diastolic BP 70.5-78 (count),PIP 26-35 (count),MAC (duration, mins),BUN 0-7 days prior to surgery (average),	0.770
174	Hao Cui (2021)	Prospective	China	Adults(> 18 years)	Incidence	Perioperative Monitoring	Cardiac and cardiac surgery-related	In hospital	214	Training cohort, Internal validation cohort, Test	3	gluconic acid, fumaric acid, and pseudouridine,Preoperative creatinine,eGFR）	0.972

										cohort			
175	Xun Wang (2021)	Retrospective	China	Adults(> 18 years)	Incidence	Early Risk Stratification	Hypoperfusion/shock-related	Public databases	1520		1	eGFR, hemoglobin, sodium, bicarbonate, total bilirubin, age, diabetes, heart failure	0.857
176	Jialin Liu (2021)	Retrospective	China	Adults(> 18 years)	Mortality	Early Risk Stratification	Other/mixed	Public databases	7548		4	Serum creatinine_min,Sodium max,Platelets x 1000 min,Bicarbonate_avg,Chloride min,BP (lowest)min,BUN min,RBC max,Age,HR (current) max,Resp (lowest) min,RBC min,Potassium max,Resp (highest)_min,BP (current) max,Platelets x 1000 max,Chloride avg,Serum creatinine max,BP (highest)max,Resp (highest)max,BP (highest)avg,Resp (lowest)_max,BMI,HR (lowest)min,Potassium min	0.792
177	Qing Qian (2021)	Retrospective	China	Adults(> 18 years)	Incidence	Early Risk Stratification	Other/mixed	Public databases	17205	Test cohort	5	age, sex and body mass index (BMI),Blood Urea Nitrogen (mg/dL), International Normalized Ratio, Glucose (mg/dL), Partial Thromboplastin Time (s), Hemoglobin (K/μL), Platelet Count (K/μL), Potassium (mg/dL), White Blood Cell Count (K/μL), Bicarbonate (mg/dL), Calcium (mg/dL), Prothrombin Time (s), Creatinine (mg/dL) and Urine Output (mL), Oxygen saturation (%)	0.905
178	Yu Tian, (2021)	Retrospective	China	Adults(> 18 years)	Incidence	Perioperative Monitoring	Cardiac and cardiac surgery-related	In hospital	58399	Training cohort, Internal validation cohort	1	age, male, obesity, history of cardiac surgery, history of peripheral vascular disease, IABP use, atrial fibrillation, proteinuria, hypertension, type 2 diabetes, NYHA classification IV, blood transfusion products use, hyperuricemia, LVEF, type of surgery, bypass time, hemorrhage volume > 600 mL, intraoperative RBC use, intraoperative platelet use, intraoperative plasma use	0.690
179	Kipyo Kim (2021)	Retrospective	Korea	Adults(> 18 years)	Incidence	Early Risk Stratification	Other/mixed	In hospital	149108	Internal validation cohort, External validation cohort	2	Serum Cr,eGFR at baseline,Sex,BUN,Department of cardiology,Diuretics,Diabetes,Nsaids,Recent NSAIDs,Cancer,PR_mean,Platelet,Other medical departments,Department of hematoncology,Statin,Department of general surgery,Age,Department of gastroenterology,Other surgical departments,Hypertension,PR_max,Department of urology,Bilirubin,Cerebrovascular disease,COPD,Charlson comorbidity index,RAS blockade,Glucose,Weight,ICU admission,Recent diuretics,Department of neurosurgery,Recent calcium channel blocker,Recent statin,WBC,Ischemic heart disease,Recent vasopressor,Heat failure,Department of pulmonology,Surgical time,SBP_mean,vasopressor,BMI,Beta blocker,Major surgery,C02,Hb,PR_min,Recent RAS blockade,BT_max,	0.927
180	Yang Li (2021)	Retrospective	China	Adults(> 18 years)	Incidence	Perioperative Monitoring	Cardiac and cardiac surgery-related	In hospital	1138	Training cohort, Internal validation cohort	1	Age,BMI,eGFR,LVEF,Statins,Adrenaline,RBC transfusion ,Use of IABP,LCOS,Reoperation for bleeding	0.735

181	Jiaming Wang (2021)	Retrospective	China	Adults(> 18 years)	Incidence	Early Risk Stratification	Other/mixed	Public databases	4672	Internal validation cohort, External validation cohort	3	the full dataset	0.932
182	Hao Du (2021)	Retrospective	Singapore	All	Incidence	Early Risk Stratification	Other/mixed	Public databases	36000		3	age, gender, congestive heart failure, cardiac arrhythmias, valvular disease, pulmonary circulation, peripheral vascular, hypertension, paralysis, neurological disorder, chronic pulmonary diseases, diabetes, hypothyroidism, renal failure, liver diseases, peptic ulcer, aids, lymphoma, metastatic cancer, rheumatoid arthritis, coagulopathy, obesity, fluid electrolyte, anemias, alcohol abuse, drug abuse, psychoses, depression, mean arterial blood pressure, heart rate, respiration rate, temperature, bilirubin, BUN, creatinine, glucose, HCO3, HCT, K, lactate, Mg, Na, PaCO2, PaO2, pH, platelets, troponin, WBC, urine output, fluid balance, mechanical ventilation, vasopressor, sedative medications	0.893
183	Donghwan Yun (2021)	Retrospective	Korea	Adults(> 18 years)	Incidence	Specialty-specific	Nephrotoxic/drug- related	In hospital	14185	Test cohort	8	Alb,Time-varying features,Na,Prot,ALP,SBP,K,Ca,Age,eGFR,P,PR,Glu,CO2,BT,Height,DBP,Chol,CI,HCT,	0.755
184	Annunziata Nusca (2021)	Retrospective	Italy	All	Incidence	Specialty-specific	Nephrotoxic/drug- related	In hospital	1344	Training cohort, Internal validation cohort	1	hypotension,intra-aortic balloon pump –IABP- insertion, congestive heart failure,age, anemia,betes mellitus,chronic renal failure,contrast medium volume,FPG ≥124 mg/dl	0.848
185	Lei Lei (2020)	Retrospective	China	Adults(> 18 years)	Incidence	Specialty-specific	Liver disease- related	In hospital	1173	Training cohort, Test cohort	4		0.999
186	R. J. Kate, (2020)	Retrospective	USA	Geriatric	Incidence	Early Risk Stratification	Other/mixed	In hospital	44691		1	Cisplatin,Aminoglycosides,Hypercalcemia,Diuretics,Acyclovir,ACE inhibitors orNSAIDS or Diuretics,ARB or ACEinhibitorsOr NSAIDS OrDiuretics,Respiratory failure,Rhabdomyolysis,K sparing	0.709
187	Yang Li (2020)	Retrospective	China	Adults(> 18 years)	Incidence	Perioperative Monitoring	Cardiac and cardiac surgery- related	In hospital	5533	Training cohort, Internal validation cohort	1	Age, Body mass index (BMI), Serum creatinine (SCr), Serum uric acid (SUA), New York Heart Association (NYHA) grade, Left ventricular ejection fractions (LVEF), Platelet, Direct bilirubin (DBil), Proteinuria, Surgery type, Aortic cross-clamp time (ACCT), Ultrafiltration volume, Postoperative central venous pressure (CVP)	0.845
188	Yang Li (2020)	Retrospective	China	Adults(> 18 years)	Incidence	Specialty-specific	Hematologic/malignancy- related	In hospital	2395		2	Age, Gender, Diabetes, Hypertension, Hematologic malignancy category (Lymphoma, Leukemia, Multiple Myeloma), Admission type (Emergent, Normal), Anti-tumor treatment (Autologous stem cell transplantation, Chemotherapy, Untreated/palliative care), Alanine aminotransferase (ALT), Aspartate aminotransferase (AST), Total bilirubin (TBIL), Serum creatinine (SCr), Estimated	0.835

												glomerular filtration rate (eGFR), Serum uric acid (SUA), Albumin, Hemoglobin, White blood cell (WBC), Serum sodium, Serum potassium	
189	Zeng-Lei He (2020)	Retrospective	China	Adults(> 18 years)	Incidence	Perioperative Monitoring	Liver disease- related	In hospital	493	Training cohort	5	Recipient BMI,Operative time,Donor TBil,Anhepatic phase,Recipient weight,Donor PLT,Graft weight,Donor BUN,Recipient INR,Recipient AFP,Recipient TB,Donor BMI,Donor age,Blood loss,ALB,Plasma transfusion,Infusion volume,Donor AST,Donor Na,Donor Cr,Cold ischemia,Age,Donor ALT,Donor ALB,Recipient Cr,Donor K,Donor Hb,MELD,Donor WBC,Recipient height,Donor weight,Donor height,Warm ischemia,RBC transfusion,Child-Pugh score,PLT transfusion,HE,Recipient hypertension,Recipient ascites,Recipient DM,Recipient tumor,Donor sex,Recipient sex,Donor HBV,Donor hypertension,Recipient HBV,ABOI,Peritonitis,Donor DM,Glucocorticoid,Piggyback,	0.850
190	Toshiki Kuno (2020)	Prospective	Japan	All	Incidence	Specialty-specific	Cardiac and cardiac surgery- related	In hospital	14273		2	age (categorized: <50, 50–59, 60–69, 70–79, 80–89, ≥90),chronic kidney disease (eGFR categories: ≥60, 45–60, 30–45, <30 mL/min/1.73 m²),previous heart failure,diabetes mellitus,cerebrovascular disease,heart failure on admission,cardiogenic shock on admission,cardiopulmonary arrest on admission,use of intra-aortic balloon pump,ST-elevation myocardial infarction,non-ST-elevation myocardial infarction/unstable angina,preprocedural hemoglobin (<10 g/dL),absolute hemoglobin decrease (>3 g/dL) , relative hemoglobin decrease (>20%)	0.826
191	Nina Rank (2020)	Retrospective	German	Adults(> 18 years)	Incidence	Perioperative Monitoring	Cardiac and cardiac surgery- related	In hospital	2574	Test cohort	1	Age, Sex, Weight, Height, Phosphate, Total bilirubin, Baseline creatinine, Creatinine, Baseline urea, Urea, Glomerular filtration rate, Creatine kinase (CK), CK-MB, Red blood count, White blood count, Platelets, C-reactive protein, Gamma-glutamyltransferase, Glutamic oxaloacetic transaminase, Hemoglobin, International normalized ratio, Lactate dehydrogenase, Magnesium, Hematocrit, Prothrombin time, Partial thromboplastin time, Mean corpuscular hemoglobin, Mean corpuscular volume, Mean corpuscular hemoglobin concentration, Aortic cross-clamp time, Cardiopulmonary bypass time, Time in operation theatre, Surgery procedure type 1 probability, Surgery procedure type 2 probability, Surgery procedure type 3 probability, Surgery procedure type 4 probability, Surgery procedure type 5 probability, Surgery procedure type 6 probability, Surgery procedure type 7 probability, Surgery procedure type 8 probability, Surgery procedure type 9 probability, Surgery procedure type 10 probability, Surgery procedure type 11 probability, Surgery procedure type 12 probability, Surgery procedure type 13 probability, Surgery procedure type 14 probability, Surgery procedure type 15 probability, Surgery procedure type 16 probability,	0.893

												Surgery procedure type 17 probability, Systolic arterial pressure, Mean arterial pressure, Diastolic arterial pressure, Central venous pressure, Heart frequency, Pulse, Body temperature, Oxygen saturation, Base excess, Bicarbonate, Glucose, Hemoglobin, Oxygen saturation, Partial pressure of carbon dioxide (PCO2), Partial pressure of oxygen (PO2), Total carbon dioxide, pH level, Potassium, Sodium, Calcium, Lactate, Carboxyhemoglobin, Oxyhemoglobin, Bleeding Rate, Urine flow rate, Allopurinol, Aminoglycosides, Amphotericin B, Antiplatelet agents (clopidogrel, ticlopidine), Benzodiazepines, Cephalosporins, Cyclosporine, Haloperidol, Ketamine, Nonsteroidal anti-inflammatory drugs, Paracetamol, Penicillins, Proton pump inhibitors, Pyrazolone derivatives, Quinolones, Ranitidine, Rifampin, Sulfonamides, Tacrolimus, (Val-)/Ganciclovir, Aciclovir, Vancomycin, Red Blood Cell Transfusions	
192	Ira S. Hofer (2020)	Retrospective	USA	Adults(> 18 years)	Incidence	Perioperative Monitoring	Trauma/non- cardiac surgery- related	In hospital	59981	Internal validation cohort	2	Age, ASA Physical Status Classification, Surgical Specialty (one-hot encoded), Minimum Systolic Blood Pressure, Maximum Systolic Blood Pressure, Minimum Diastolic Blood Pressure, Maximum Diastolic Blood Pressure, Minimum Mean Arterial Pressure, Maximum Mean Arterial Pressure, Minimum Heart Rate, Maximum Heart Rate, Total Crystalloid Volume, Total Colloid Volume, Total Packed Red Blood Cells Transfused, Total Fresh Frozen Plasma Transfused, Total Platelets Transfused, Total Cell Saver Volume, Estimated Blood Loss, Total Phenylephrine Dose, Total Ephedrine Dose, Total Epinephrine Dose, Total Norepinephrine Dose, Total Dopamine Dose, Total Dobutamine Dose, Total Vasopressin Dose, Total Furosemide Dose, Total Mannitol Dose, Total Heparin Dose, Total Protamine Dose, Minimum End-Tidal Carbon Dioxide, Maximum End-Tidal Carbon Dioxide, Minimum Pulse Oximetry, Maximum Pulse Oximetry, Arterial Line Used, Central Venous Catheter Used, Pulmonary Artery Catheter Used, Case Duration, Total Urine Output, Duration of MAP < 40 mmHg, Duration of MAP < 45 mmHg, Duration of MAP < 50 mmHg, Duration of MAP < 55 mmHg, Duration of MAP < 60 mmHg, Duration of MAP < 65 mmHg	0.792
193	Jahan C. Penny-Dimri (2020)	Retrospective	Australia	Adults(> 18 years)	Incidence	Perioperative Monitoring	Cardiac and cardiac surgery- related	In hospital	96653	Training cohort	4	Age, Gender (Female/Male), Body Mass Index (BMI), Indigenous Australian ethnicity, Smoking history, Hypercholesterolemia, Hypertension, Peripheral vascular disease, Heart failure, Diabetes mellitus, Cerebrovascular disease, Chronic lung disease, Previous myocardial infarction, Previous cardiothoracic procedure, Previous cardiac catheterization, Left main coronary artery disease, History of any cardiac arrhythmia, Atrial fibrillation, Ventricular dysrhythmia, Complete heart block, Number of diseased coronary systems, Left ventricular ejection fraction estimate, NYHA	0.780

												functional class, CCS angina class, Diabetes management, Lung disease severity, Preoperative renal function (Serum creatinine), Preoperative hemoglobin/hematocrit, Preoperative white blood cell count, Preoperative platelet count, Preoperative electrolytes, Admission status, Recent immunosuppression therapy, Recent systemic steroids use, Preoperative beta-blocker use, Preoperative ACEI/ARB use, Preoperative statin use, Preoperative antiplatelet therapy, Preoperative anticoagulation therapy, Intraoperative inotrope/vasopressor use, Procedure on aortic valve, Mitral valve procedure, Tricuspid valve procedure, Combined valve surgery, Coronary artery bypass grafting (CABG) alone, Combined CABG + valve surgery, Cardiopulmonary bypass (CPB) time, Aortic cross-clamp time, Off-pump CABG, Minimally invasive cardiac surgery, Redo cardiac surgery, Intraoperative blood transfusion, Intraoperative fluid balance, Intraoperative urine output, Duration of surgery, Postoperative intensive care unit (ICU) admission urgency	
194	Chien-Ning Hsu (2020)	Retrospective	China	Adults(> 18 years)	Incidence	Early Risk Stratification	Other/mixed	In hospital	234867	Training cohort, Internal validation cohort	2	Baseline SCr,Baseline eGFR,Baseline BUN,Baseline Calcium,CKD,Age at admission,RASI/Diuretics ,Baseline Phosphorus,Diabetes without complications,Severe liver disease	0.789
195	C. Qu (2020)	Retrospective	China	Adults(> 18 years)	Incidence	ICU Real-time Alert	Severe acute pancreatitis (SAP)	In hospital	334	Test cohort	5	5	0.919
196	Ling Sun (2020)	Retrospective	China	Adults(> 18 years)	Incidence	Specialty-specific	Nephrotoxic/drug- related	In hospital	1495	Training cohort, Internal validation cohort	3		1.000
197	Khaled Shawwa (2020)	Retrospective	USA	Adults(> 18 years)	Incidence	Early Risk Stratification	Other/mixed	In hospital	117001	Training cohort, Internal validation cohort, External validation cohort	1	Hospital Admit BMI,Chronic kidney disease,Congestive heart failure; nonhypertensive,Coagulation and hemorrhagic disorders,Cardiac dysrhythmias,Age at ICU Admit,,Weight (kg) Hosp AdmitCardiac arrest and ventricular fibrillation,Cancer of brain and nervous system,admitdiagnosis_Heart valve disorders,Baseline creatinine combined,Pulmonary heart disease,admitdiagnosis_Septicemia (except in labor),Chronic ulcer of skin,Acute myocardial infarction,BMI increase,admitdiagnosis_Respiratory failure; insufficiency; arrest (adult),admitdiagnosis_Other and unspecified benign neoplasm,Other nutritional, endocrine, and metabolic disorders,baseline creatinine available,Weight increase,Chronic obstructive pulmonary disease and bronchiectasis,Disorders of lipid metabolism,Nephritis; nephrosis; renal sclerosis,Hypertension with complications and secondary hypertension,Diseases of white blood cells,admitdiagnosis_Other fractures,Other endocrine disorders,Diabetes mellitus with	0.707

												complications,Coronary atherosclerosis and other heart disease,	
198	Yi Du (2020)	Retrospective	China	Adults(> 18 years)	Incidence	Specialty-specific	Cardiac and cardiac surgery- related	In hospital	292	Training cohort, Internal validation cohort	1	operation methods, age, congestive heart failure, BMI, heart rate, WBC count, platelet count, hemoglobin, uric acid, PI kidney cortex, PI kidney medulla	0.967
199	Yang Li (2020)	Retrospective	China	Adults(> 18 years)	Incidence	Perioperative Monitoring	Hematologic/malignancy- related	In hospital	6495	Training cohort, Internal validation cohort	6	diabetes, cancer category, treatment, ALT, SCr, eGFR, SUA, hypoalbuminemia, anemia, abnormal sodium, potassium levels	0.823
200	P. Y. Tseng (2020)	Retrospective	China	All	Incidence	Perioperative Monitoring	Cardiac and cardiac surgery- related	In hospital	671	Training cohort	6		0.843
201	Guiyu Lei (2020)	Retrospective	China	All	Incidence	Perioperative Monitoring	Cardiac and cardiac surgery- related	In hospital	897	Test cohort	4	BMI,body mass index; CABG, coronary artery bypass grafting; CPB, cardiopulmonary bypass; FFP, fresh frozen plasma; Hb, hemoglobin; LVEF, left ventricular ejec tion fraction; PRBC, packed red blood cells; SCr, serum creatinine.	0.800
202	Qi Wang (2019)	Prospective	China	Adults(> 18 years)	Incidence	Early Risk Stratification	Other/mixed	In hospital	936	Training cohort, Test cohort	1	hypertension, chronic kidney disease, acute pancreatitis, cardiac failure, shock, pH≤7.30, CK>1000 U/L, hypoproteinemia, nephrotoxin exposure, and male	0.833
203	Caitlyn Chiofolo (2019)	Retrospective	USA	Adults(> 18 years)	Incidence	Early Risk Stratification	Other/mixed	In hospital	6530	Training cohort, Test cohort	1	Serum urea nitrogen, pH, Age, Noninvasive diastolic blood pressure, Temperature, Noninvasive mean arterial blood pressure, Hematocrit level, Serum sodium concentration, Serum potassium concentration, Estimated glomerular filtration rate (eGFR) based on serum creatinine level, Median urine output at 12 hours, Median urine output at 24 hours, Shock index based on noninvasive diastolic blood pressure, Shock index based on invasive diastolic blood pressure, Pulse pressure, Delivered tidal volume, Partial pressure of arterial oxygen to fraction of inspired oxygen ratio (PaO2/FiO2), Net fluid balance, Cumulative dose of normal saline	0.949
204	JOSHUA PARRECO (2019)	Prospective	USA	Adults(> 18 years)	Incidence	Early Risk Stratification	Other/mixed	Public databases	151098		3	Creatinine, BUN, Glucose, Potassium, Sodium, Chloride, Calcium, Bicarbonate, Anion gap, Magnesium, Phosphate, Hemoglobin, Hematocrit, Platelets, WBC, Bilirubin, AST, ALT, Alkaline phosphatase, PT, PTT, Lactate, Albumin, Prealbumin, Temperature, Respiratory rate, Heart rate, Blood pressure	0.834
205	Catalina Martin-Cleary (2019)	Retrospective	Spain	Adults(> 18 years)	Incidence	Early Risk Stratification	Other/mixed	In hospital	47466		3	Abdominal surgery,Cardiovascular surgery,Urological surgery,Congestive heart failure,Hemiplegia,Renal disease,Rheumatic disease,Liver disease,Malignancy,Cardiovascular disease,Cerebrovascular disease,Anaemia,Diabetes,Surgical admission,Urgent admission,Age (years),Uric acid (mg/dL),Urea (mg/dL),Calcium (mg/dL),Leucocytes (n/L),Sodium	0.818

												(mEq/L),Glucose (mg/dL),(Potassium)² (mEq/L)	
206	Nakyung Jeon (2019)	Retrospective	USA	Adults(> 18 years)	Incidence	Early Risk Stratification	Other/mixed	In hospital	62561		1	Acidosis, Albuminemia, Anemia, Cardiac surgery, Chronic hypertension, Chronic kidney disease, Critically ill, Dehydration, Diabetes, Sex (Male), Heart failure, Hypotension, Increasing trend in serum creatinine (SCRs), Liver injury, Obesity, Oliguria, Prolonged surgery, Stage 1 acute kidney injury (AKI), Systemic inflammatory response, Number of highly nephrotoxic drugs, Number of nephrotoxic drugs, Use of piperacillin/tazobactam (TZP) + vancomycin	0.790
207	Lasith Adhikari (2019)	Retrospective	Italy	Adults(> 18 years)	Mortality	Perioperative Monitoring	Trauma/non- cardiac surgery- related	In hospital	2911	Internal validation cohort	1		0.870
208	Jianqin He, (2019)	Retrospective	USA	Adults(> 18 years)	Incidence	Early Risk Stratification	Other/mixed	In hospital	72846		5		0.734
209	Ke Lin (2019)	Prospective	USA	Adults(> 18 years)	Mortality	Early Risk Stratification	Other/mixed	Public databases	19044		3	urine output, systolic blood pressure, age, serum bicarbonatelevel and heart rate.	0.866
210	Daishan Jiang (2019)	Retrospective	China	Adults(> 18 years)	Incidence	Early Risk Stratification	Sepsis/infection- related	In hospital	442		1	Age,Lactate,CRP,cTnI,NT-proBNP,H-FABP	0.755
211	Hyung-Chul Lee (2018)	Retrospective	Korea	Adults(> 18 years)	Incidence	Perioperative Monitoring	Cardiac and cardiac surgery- related	In hospital	2010	Test cohort	6	Intraop_pRBC,Preoperative hematocrit,Preoperative creatinine,Total_intraop_Fluid_admin,Surgery Time,Preop_left_ventricle_EF,Preop_uric_acid,Intraoperative_FFP,Preop_E_of_e'_more_15,Mean_cardiac_index,Preoperative_glucose,Anesthesia_time,Age,Preoperative_albumin,Body_mass_index,Intraoperative_colloid,Mean_arterial_pressure,Intraoperative_diuretics,Mean_SvO2,Hypertension,Chronic_Kidney_disease,Previous_cardiac_disease,Preoperative_CCB,Diabetes_mellitus,Preoperative_beta_blocker,Preoperative_ARB,GenderPreoperative_statin,Surgery_type_CABG,Cerebrovascular-accident,Three_vessel_disease,Emergency_case,Previous_cardiac_surgeryPrevious_coronary_stent,COPD,Preoperative_diuretics,Preoperative_pul_HTN,Surge-type-aorta,Surgey-type-valve	0.780
212	Chenxi Huang (2018)	Retrospective	USA	Adults(> 18 years)	Incidence	Specialty-specific	Cardiac and cardiac surgery- related	In hospital	970869	Internal validation cohort	2		0.759

213	Kun Zhu (2018)	Retrospective	Korea	All	Incidence	Perioperative Monitoring	Liver disease-related	In hospital	1211	Test cohort	8	Cold ischemic time(min)Mean SvO2(%)Mean Cardiac Index(L/min/m²)Urine output(mL/kg/hr)Preoperative Glucose(mg/dL)Preoperative Creatinine(mg/dL)Preoperative Hb(g/dL)Mean Glucose (mg/dL)Warm ischemic time(min)Preoperative K+(meq/L)MELD scoreAge(year)Preoperative Platelet(10⁹/L)EBLAscites removal(mL)Operation time (hr)BMI(kg/m²)Mean Hb(g/dL)Mean femoral ABP(mmHg)Colloid(mL)	0.900
214	Li Wang (2017)	Retrospective	USA	Pediatric	Incidence	Early Risk Stratification	Other/mixed	In hospital	6009	Internal validation cohort	1	Age at admission,High risk nephrotoxins,Moderate risk nephrotoxins,Total medications,Minimum platelet count,Median RDW,Phosphorus,None checked,Phosphorus,Transaminases,Transaminases,Minimum pH,Hypotension	0.740
215	Yujun Deng (2017)	Prospective	China	Adults(> 18 years)	Incidence	Early Risk Stratification	Other/mixed	In hospital	1084		1	Serum cystatin C (sCysC), Urinary N-acetyl-β-D-glucosaminidase (uNAG), Urinary albumin/creatinine ratio (uACR)	0.863
216	L. Nelson Sanchez-Pinto (2016)	Retrospective	USA	Pediatric	Incidence	Early Risk Stratification	Other/mixed	In hospital	9396	Training cohort, Internal validation cohort	1		0.860
217	Kate, R. J (2016)	Retrospective	USA	Geriatric	Incidence	Early Risk Stratification	Other/mixed	In hospital	25521	Training cohort, Test cohort	5		0.743
218	Shu Yi Ng, (2014)	Prospective	Australia	Adults(> 18 years)	Incidence	Perioperative Monitoring	Cardiac and cardiac surgery-related	In hospital	28422		1	obesity, preoperativecreatinine level, diabetes, IABP use pre-, peri-, and/or post operatively, infective endocarditis, bypass time, cardio genic shock, non-RBC bloodobesity, preoperativecreatinine level, diabetes, IABP use pre-, peri-, and/or post operatively, infective endocarditis, bypass time, cardio genic shock, non-RBC blood	0.810
219	Won Ho Kim (2013)	Retrospective	Korea	Adults(> 18 years)	Incidence	Perioperative Monitoring	Cardiac and cardiac surgery-related	In hospital	737	Training cohort, Internal validation cohort	1	Age older than 60 years, Preoperative GFR <60 mL/min/1.73 m², Preoperative LVEF <55%, Operation time >7 hours, Intraoperative oliguria (<0.5 mL/kg/h), Intraoperative furosemide use	0.740

Supplementary Table S6. Study-Level PROBAST+AI Assessment of Risk of Bias, Reporting Quality, and Applicability

Quality and concerns about applicability for each study using PROBAST+AI Checklist																										
ID	Study	1.1	1.2	1.3	1A	1B	2.1	2.2	2.3	2.4	2A	2B	3.1	3.2	3.3	3.4	3A	3B	4.1	4.2	4.3	4.4	4.5	4A	Overall concern regarding quality of the prediction model development	Overall concern for applicability of the prediction model development
4	Suru Yue, 2022	Y	PY	Y	high	high	Y	Y	Y	Y	high	high	Y	Y	Y	Y	high	high	Y	Y	Y	N/A	Y	high	high	high
8	Zhiyan Fan, 2023	Y	Y	Y	high	high	Y	Y	Y	Y	high	high	Y	Y	Y	Y	high	high	Y	Y	Y	Y	Y	high	high	high
9	R. J. Kate,2020	PY	PY	PY	high	high	Y	Y	PN	Y	high	high	Y	Y	PN	Y	high	high	Y	Y	PY	PN	Y	high	high	high
11	Wanyue Zhang, 2024	Y	PY	Y	high	high	Y	Y	Y	Y	high	high	Y	Y	Y	Y	high	high	Y	Y	Y	N/A	Y	high	high	high
12	P. Y. Tseng,2020	PY	PY	PY	high	high	Y	Y	PY	Y	high	high	Y	Y	PY	Y	high	high	PN	Y	Y	PN	Y	low	low	high
14	Xiao-Qin Luo, 2023	Y	PY	Y	high	high	Y	Y	Y	Y	high	high	Y	Y	PN	Y	high	high	Y	Y	Y	Y	Y	high	high	high
15	Qiuchong Chen, 2022	Y	PY	Y	high	high	Y	Y	Y	Y	high	high	Y	Y	PN	Y	high	high	PN	Y	NI	N/A	Y	low	low	high
18	Yisong Cheng, 2022	PY	PY	Y	high	high	Y	Y	Y	Y	high	high	Y	Y	PN	Y	high	high	Y	Y	NI	N/A	Y	low	low	high
26	Tianyun Gao, 2024	PY	PY	PY	high	high	Y	Y	Y	Y	high	high	Y	Y	PN	Y	high	high	Y	Y	Y	N/A	Y	high	high	high
31	Shengwei Lin, 2024	PY	PY	PY	high	high	Y	Y	Y	Y	high	high	Y	Y	PN	Y	high	high	NI	Y	Y	N/A	Y	low	low	high
48	Shuxing Wei, 2023	PY	PY	PY	high	high	Y	Y	Y	Y	high	high	Y	Y	PN	Y	high	high	Y	Y	Y	Y	Y	high	high	high
77	Rufa Zhang, 2024	PY	PY	PY	high	high	Y	Y	Y	Y	high	high	Y	Y	PN	Y	high	high	PN	Y	Y	N/A	Y	low	low	high
78	Xunliang Li, 2024	PY	PY	PY	high	high	Y	Y	Y	Y	high	high	Y	Y	PN	Y	high	high	Y	Y	Y	N/A	Y	high	high	high
79	Suhua She, 2023	PY	PY	PY	high	high	Y	NI	Y	Y	NI	high	Y	Y	PN	Y	high	high	Y	Y	NI	N/A	PY	low	low	high
83	Jianshan Shi, 2024	PY	PY	PY	high	high	Y	Y	Y	Y	high	high	Y	Y	PN	Y	high	high	Y	Y	Y	PN	Y	high	high	high
87	Yongbin Wang, 2024	PY	PY	PY	high	high	Y	Y	Y	Y	high	high	Y	Y	PN	Y	high	high	Y	Y	Y	N/A	Y	high	high	high
90	Christopher T Ryan, 2023	PY	PY	PY	high	high	Y	Y	Y	Y	high	high	Y	Y	PN	Y	high	high	Y	Y	PY	Y	Y	high	high	high
96	Jurij Matija Kalisnik, 2022	PY	PY	PY	high	high	Y	Y	Y	Y	high	high	Y	Y	PN	Y	high	high	Y	Y	Y	NI	Y	NI	NI	high
98	Jin Wang, 2022	PY	PY	PY	high	high	Y	NI	Y	Y	NI	high	Y	Y	PN	Y	high	high	PY	Y	NI	PN	Y	low	low	high
99	Toshiki Kuno, 2022	Y	Y	Y	high	high	Y	Y	Y	Y	high	high	Y	Y	Y	Y	high	high	Y	Y	Y	NI	Y	NI	NI	high
101	Hao Cui, 2021	Y	Y	Y	high	high	Y	Y	Y	Y	high	high	Y	Y	Y	Y	high	high	PN	Y	Y	NI	PY	low	low	high
102	Hongshan Zhou, 2023	PY	PY	PY	high	high	Y	Y	Y	Y	high	high	Y	Y	PN	Y	high	high	Y	Y	PY	N/A	Y	high	high	high
109	Azar Ejmalian, 2022	PY	PY	PY	high	high	Y	Y	Y	Y	high	high	Y	Y	PN	Y	high	high	Y	Y	Y	Y	Y	high	high	high
136	Luming Zhang, 2022	PY	PY	PY	high	high	Y	Y	Y	Y	high	high	Y	Y	PN	Y	high	high	Y	Y	Y	N/A	Y	high	high	high
137	Chang Hu, 2022	PY	PY	PY	high	high	Y	Y	Y	Y	high	high	Y	Y	PN	Y	high	high	Y	Y	Y	N/A	Y	high	high	high
140	Kaidi Gong, 2021	PY	PY	PY	high	high	Y	Y	Y	Y	high	high	Y	Y	PN	Y	high	high	Y	Y	Y	N/A	Y	high	high	high
141	Yamin Liu, 2024	PY	PY	PY	high	high	Y	Y	Y	Y	high	high	Y	Y	PN	Y	high	high	Y	Y	Y	PN	Y	high	high	high
	Jicheng Jiang	PY	PY	PY	high	high	Y	Y	Y	Y	high	high	Y	Y	PN	Y	high	high	Y	Y	Y	N/A	Y	high	high	high

Quality and concerns about applicability for each study using PROBAST+AI Checklist																										
**ID**	**Study**	**1.1**	**1.2**	**1.3**	**1A**	**1B**	**2.1**	**2.2**	**2.3**	**2.4**	**2A**	**2B**	**3.1**	**3.2**	**3.3**	**3.4**	**3A**	**3B**	**4.1**	**4.2**	**4.3**	**4.4**	**4.5**	**4A**	**Overall concern regarding quality of the prediction model development**	**Overall concern for applicability of the prediction model development**
157	Jun Liu, 2022	PY	PY	PY	high	high	Y	Y	Y	Y	high	high	Y	Y	PN	Y	high	high	PN	Y	Y	N/A	Y	low	low	high
158	Yun Yan, 2023	PY	PY	PY	high	high	Y	Y	Y	Y	high	high	Y	Y	PN	Y	high	high	Y	Y	Y	N/A	Y	high	high	high
163	Tianchen Jia, 2023	PY	PY	PY	high	high	Y	Y	Y	Y	high	high	Y	Y	PN	Y	high	high	Y	Y	NI	N/A	Y	high	high	high
164	Yirui Hu, 2022	PY	PY	PY	high	high	Y	Y	Y	Y	high	high	Y	Y	PN	Y	high	high	Y	Y	Y	N/A	Y	high	high	high
169	Amanda C Filiberto, 2021	PY	PY	PY	high	high	Y	NI	Y	Y	NI	high	Y	Y	PN	Y	high	high	PN	Y	NI	N/A	Y	low	low	high
170	Xi Peng, 2022	PY	PY	PY	high	high	Y	Y	Y	Y	high	high	Y	Y	PN	Y	high	high	Y	Y	Y	N/A	Y	high	high	high
173	Yuchen Gao, 2023	PY	PY	PY	high	high	Y	Y	Y	Y	high	high	Y	Y	PN	Y	high	high	Y	Y	Y	N/A	Y	high	high	high
174	Rao Sun,2024	Y	PY	Y	high	high	Y	Y	Y	Y	high	high	Y	Y	Y	Y	high	high	Y	Y	Y	N/A	Y	high	high	high
175	Lei Dong, 2024	PY	PY	PY	high	high	Y	Y	Y	Y	high	high	Y	Y	PN	Y	high	high	Y	Y	PY	Y	Y	high	high	high
176	Rima S Zahr, 2025	PY	PY	PY	high	high	Y	PY	Y	Y	high	high	Y	Y	PN	Y	high	high	N	Y	PY	Y	Y	low	low	high
178	Sunho Ko, 2022	PY	PY	PY	high	high	Y	Y	Y	Y	high	high	Y	Y	PN	Y	high	high	Y	Y	PN	N/A	Y	high	high	high
181	Yuezi Song, 2024	PY	PY	PY	high	high	Y	Y	Y	Y	high	high	Y	Y	PN	Y	high	high	N	Y	Y	N/A	Y	low	low	high
182	Sasha Pierre-Paul, 2022	PY	PY	PY	high	high	Y	Y	Y	Y	high	high	Y	Y	PN	Y	high	high	PN	PN	NI	N/A	PY	low	low	high
183	Jie Tang, 2024	PY	PY	PY	high	high	Y	Y	Y	Y	high	high	Y	Y	PN	Y	high	high	Y	Y	PY	N/A	Y	high	high	high
185	Xunliang Li, 2023	PY	PY	PY	high	high	Y	Y	Y	Y	high	high	Y	Y	PN	Y	high	high	Y	Y	Y	N/A	Y	high	high	high
186	Yelena Petrosyan, 2022	PY	PY	PY	high	high	Y	Y	Y	Y	high	high	Y	Y	PN	Y	high	high	Y	Y	NI	N/A	Y	high	high	high
187	C. Qu,2020	PY	PY	PY	high	high	Y	Y	Y	Y	high	high	Y	Y	PN	Y	high	high	PN	Y	PN	N/A	Y	low	low	high
188	Lei Yuan, 2024	PY	PY	PY	high	high	Y	Y	Y	Y	high	high	Y	Y	Y	Y	high	high	Y	Y	NI	N	Y	low	low	high
189	Yefeng Tong, 2023	PY	PY	PY	high	high	Y	Y	Y	Y	high	high	Y	Y	PN	Y	high	high	Y	Y	NI	N/A	Y	NI	NI	high
192	Qian Li, 2023	PY	PY	PY	high	high	Y	Y	Y	Y	high	high	Y	Y	PN	Y	high	high	Y	Y	Y	N/A	Y	high	high	high
199	Emma Schwager, 2024	PY	PY	PY	high	high	Y	Y	Y	Y	high	high	Y	Y	PN	Y	high	high	Y	Y	Y	N/A	Y	high	high	high
201	Guiyu Lei,2020	PY	PY	PY	high	high	Y	NI	PN	Y	low	high	Y	Y	PN	Y	high	high	Y	NI	NI	N/A	Y	low	low	high
207	Zhi Xiang Du,2022	PY	PY	PN	high	low	Y	Y	PY	Y	high	high	Y	Y	Y	Y	high	high	N	Y	NI	N/A	Y	low	low	low
211	Rui Fan, 2023	PY	PY	PY	high	high	Y	Y	PY	Y	high	high	Y	Y	PN	Y	high	high	Y	Y	Y	N/A	Y	high	high	high
212	Caitlyn Chiofolo,2019	PY	PY	PY	high	high	Y	NI	PN	Y	low	high	Y	Y	Y	Y	high	high	Y	NI	Y	N/A	Y	low	low	high
216	Charat Thongprayoon, 2022	PY	PY	PY	high	high	Y	Y	Y	Y	high	high	Y	Y	PN	Y	high	high	Y	Y	PN	N/A	Y	high	high	high
217	Yeonhee Lee, 2021	PY	PY	PY	high	high	Y	NI	Y	Y	NI	high	Y	Y	PN	Y	high	high	Y	Y	NI	N/A	Y	NI	NI	high
	Xian Zeng	PY	PY	PY	high	high	Y	Y	Y	Y	high	high	Y	Y	PN	Y	high	high	Y	Y	Y	Y	Y	high	high	high

Quality and concerns about applicability for each study using PROBAST+AI Checklist																										
**ID**	**Study**	**1.1**	**1.2**	**1.3**	**1A**	**1B**	**2.1**	**2.2**	**2.3**	**2.4**	**2A**	**2B**	**3.1**	**3.2**	**3.3**	**3.4**	**3A**	**3B**	**4.1**	**4.2**	**4.3**	**4.4**	**4.5**	**4A**	**Overall concern regarding quality of the prediction model development**	**Overall concern for applicability of the prediction model development**
220	Yi Yang,2022	Y	PY	Y	high	high	Y	Y	Y	Y	high	high	Y	Y	Y	Y	high	high	N	Y	PY	N/A	Y	low	low	high
222	Wen Tao Liu, 2022	PY	PY	PY	high	high	Y	Y	Y	Y	high	high	Y	Y	PN	Y	high	high	Y	Y	PY	N/A	Y	high	high	high
226	Fangfang Zhou, 2023	PY	PY	PY	high	high	Y	Y	Y	Y	high	high	Y	Y	PN	Y	high	high	Y	Y	PY	Y	Y	high	high	high
227	Tingting Fan, 2023	PY	PY	PY	high	high	Y	Y	Y	Y	high	high	Y	Y	PN	Y	high	high	Y	Y	PY	N/A	Y	high	high	high
230	Mengqing Ma, 2023	PY	PY	PY	high	high	Y	Y	Y	Y	high	high	Y	Y	PN	Y	high	high	Y	Y	Y	Y	Y	high	high	high
232	Amir Kamel Rahimi, 2023	PY	PY	PY	high	high	Y	NI	Y	Y	NI	high	Y	Y	PN	Y	high	high	Y	Y	Y	Y	Y	high	high	high
233	Xiaohong Zhang, 2022	PY	PY	PY	high	high	Y	Y	Y	Y	high	high	Y	Y	PN	Y	high	high	Y	Y	Y	N/A	Y	high	high	high
236	Chun-Te Huang, 2023	Y	PY	Y	high	high	Y	Y	Y	Y	high	high	Y	Y	PN	Y	high	high	Y	Y	NI	N/A	Y	high	high	high
237	Jahan C. Penny-Dimri, 2020	PY	PY	PY	high	high	Y	Y	Y	Y	high	high	Y	Y	PN	Y	high	high	Y	Y	N	N/A	Y	low	low	high
239	Yan Luo, 2023	PY	PY	PY	high	high	Y	Y	Y	Y	high	high	Y	Y	PN	Y	high	high	Y	Y	Y	N	Y	low	low	high
240	Takaya Okawa, 2022	PY	PY	PY	high	high	Y	Y	Y	Y	high	high	Y	Y	PN	Y	high	high	Y	Y	N	N	Y	low	low	high
241	Khaled Shawwa, 2020	PY	PY	PY	high	high	Y	PY	Y	Y	high	high	Y	Y	PN	Y	high	high	Y	Y	NI	N/A	Y	low	low	high
245	Hsin-Hsiung Chang, 2022	PY	PY	PY	high	high	Y	Y	Y	Y	high	high	Y	Y	PN	Y	high	high	Y	Y	Y	N/A	Y	high	high	high
246	Dabei Cai, 2022	PY	PY	PY	high	high	Y	PY	Y	Y	high	high	Y	Y	PN	Y	high	high	Y	Y	Y	N	Y	low	low	high
250	Siavash Zamirpour, 2023	PY	PY	PY	high	high	Y	Y	Y	Y	high	high	Y	Y	PN	Y	high	high	Y	Y	PY	N/A	Y	high	high	high
253	Ling Sun,2020	PY	PY	PY	high	high	Y	Y	Y	Y	high	high	Y	Y	PN	Y	high	high	Y	Y	PY	PN	Y	high	high	high
256	Teddy Lazebnik, 2022	PY	PY	PY	high	high	Y	Y	Y	Y	high	high	Y	Y	PN	Y	high	high	Y	Y	PN	N/A	Y	low	low	high
259	Jialin Liu, 2021	PY	PY	PY	high	high	Y	Y	Y	Y	high	high	Y	Y	PN	Y	high	high	Y	Y	PN	N/A	Y	low	low	high
262	Yu Zhang,2022	Y	PY	Y	high	high	Y	Y	PY	Y	high	high	Y	Y	Y	Y	high	high	Y	Y	PN	N/A	Y	low	low	high
263	Huanhuan Shi, 2023	PY	PY	PY	high	high	Y	PY	Y	Y	high	high	Y	Y	PN	Y	high	high	Y	Y	PN	N/A	Y	low	low	high
264	Jun Wei, 2024	PY	PY	PY	high	high	Y	Y	Y	Y	high	high	Y	Y	PN	Y	high	high	Y	Y	Y	N/A	Y	high	high	high
272	Muying Wu, 2023	PY	PY	PN	low	high	Y	Y	Y	Y	high	high	Y	Y	PN	Y	high	high	Y	Y	Y	PN	Y	low	low	high
283	Shanbing Hou,2024	PY	PY	PN	low	high	Y	Y	PY	Y	high	high	Y	Y	Y	Y	high	high	Y	Y	N	N/A	Y	low	low	high
284	Hai-Xia Liu, 2024	PY	PY	PY	high	high	Y	Y	Y	Y	high	high	Y	Y	PN	Y	high	high	Y	Y	Y	N/A	Y	high	high	high
285	Zeng-Lei He, 2020	PY	PY	PN	low	high	Y	Y	Y	Y	high	high	Y	Y	PN	Y	high	high	N	PY	NI	N/A	N	low	low	high
286	Li Xinsai, 2022	PY	PY	PN	low	high	Y	Y	Y	Y	high	high	Y	Y	PN	Y	high	high	N	Y	PN	N/A	Y	low	low	high
289	Yihan Zhang, 2021	PY	PY	PY	high	high	Y	Y	Y	Y	high	high	Y	Y	PN	Y	high	high	Y	Y	Y	N/A	Y	high	high	high
	Le L	PY	PY	PY	high	high	Y	Y	PN	Y	low	high	Y	Y	PN	Y	high	high	Y	Y	Y	N/A	Y	high	low	high

Quality and concerns about applicability for each study using PROBAST+AI Checklist

ID	Study	1.1	1.2	1.3	1A	1B	2.1	2.2	2.3	2.4	2A	2B	3.1	3.2	3.3	3.4	3A	3B	4.1	4.2	4.3	4.4	4.5	4A	Overall concern regarding quality of the prediction model development	Overall concern for applicability of the prediction model development
295	Jijun Yang, 2023	PY	PY	PY	high	high	Y	Y	Y	Y	high	high	Y	Y	PN	Y	high	high	Y	Y	Y	Y	Y	high	high	high
300	Jiakang Shao, 2023	PY	PY	PY	high	high	Y	Y	Y	Y	high	high	Y	Y	PN	Y	high	high	Y	Y	N	Y	Y	low	low	high
302	Kun Zhu,2018	PY	PY	PY	high	high	Y	Y	PN	Y	high	high	Y	Y	PN	Y	high	high	N	Y	PY	N/A	Y	low	low	high
303	Changho Han, 2024	PY	PY	PY	high	high	Y	Y	PN	Y	high	high	Y	Y	PN	Y	high	high	NI	Y	NI	N/A	Y	low	low	high
307	Shengqiang Chi, 2023	PY	PY	PY	high	high	NI	N	PN	Y	low	high	Y	Y	PN	Y	high	high	NI	Y	PN	N/A	Y	low	low	high
308	Dongliang Yang,2022	Y	PY	Y	high	high	Y	NI	PY	Y	NI	high	Y	Y	Y	Y	high	high	Y	Y	N	N/A	Y	low	low	high
309	Anran Dai, 2023	PY	PY	PY	high	high	Y	Y	PY	Y	high	high	Y	Y	PN	Y	high	high	Y	Y	Y	N/A	Y	high	high	high
311	Xiao-Qin Luo, 2022	PY	PY	PY	high	high	Y	Y	PN	Y	high	high	Y	Y	PN	Y	high	high	Y	PY	Y	N/A	Y	high	high	high
321	Yen-Yu Chen, 2023	PY	PY	PN	low	high	Y	Y	PN	Y	high	high	Y	Y	PN	Y	high	high	Y	Y	PN	PN	Y	low	low	high
332	Ling Pan, 2023	PY	PY	PY	high	high	Y	NI	PY	Y	NI	high	Y	Y	PN	Y	high	high	Y	Y	NI	N/A	Y	NI	NI	high
336	Yu Tian,, 2021	PY	PY	Y	high	high	Y	Y	PN	Y	high	high	Y	Y	PN	Y	high	high	Y	Y	PY	N/A	Y	high	high	high
341	Lingyu Xu, 2024	PY	PY	PY	high	high	Y	Y	PN	Y	high	high	Y	Y	PN	Y	high	high	Y	Y	Y	PN	Y	high	high	high
344	Mee Yeon Lee, 2024	PY	PY	Y	high	high	Y	Y	PN	Y	high	high	PY	Y	PN	Y	high	high	Y	Y	PY	N/A	Y	high	high	high
347	Hyung-Chul Lee,2018	PY	PY	PY	high	high	Y	Y	PN	Y	high	high	Y	Y	PN	Y	high	high	Y	Y	Y	PN	Y	high	high	high
351	JOSHUA PARRECO,2019	PY	PY	PN	high	high	Y	NI	PN	Y	low	high	Y	Y	PN	Y	high	high	Y	PY	N	PN	Y	low	low	high
352	Wenjuan Zhang, 2024	PY	PY	PY	high	high	Y	NI	PN	Y	NI	high	Y	Y	PN	Y	high	high	N	NI	NI	N/A	Y	low	low	high
362	Chien-Ning Hsu,2020	PY	PY	PY	high	high	Y	Y	PN	Y	high	high	Y	Y	PN	Y	high	high	Y	Y	PY	N/A	Y	high	high	high
363	Mengqing Ma, 2024	PY	PY	PY	high	high	Y	Y	PN	Y	high	high	Y	Y	PN	Y	high	high	Y	Y	Y	PN	Y	high	high	high
366	Xiran Peng, 2024	Y	Y	PY	high	high	Y	Y	Y	Y	high	high	Y	Y	Y	Y	high	high	Y	Y	NI	N/A	Y	low	low	high
371	Xiang Yu, 2022	PY	PY	PY	high	high	Y	Y	PN	Y	high	high	Y	Y	PN	Y	high	high	PN	Y	Y	Y	PN	low	low	high
379	Justin Y Lu, 2022	PY	PY	PY	high	high	Y	Y	PN	Y	high	high	Y	Y	PN	Y	high	high	Y	Y	N	N/A	Y	low	low	high
384	Lei Lei,2020	PY	PY	PY	high	high	Y	PY	PN	Y	high	high	Y	Y	PN	Y	high	high	PN	Y	PN	N/A	PY	low	low	high
394	Lauren A Scanlon, 2021	PY	PY	PY	high	high	Y	Y	PN	Y	high	high	Y	Y	PN	Y	high	high	Y	Y	Y	Y	Y	high	high	high
395	Liu Li, 2023	PY	PY	PY	high	high	Y	NI	PN	Y	low	high	Y	Y	PN	Y	high	high	N	Y	NI	N/A	Y	low	low	high
398	Jia Tian, 2023	PY	PY	PY	high	high	Y	NI	PN	Y	low	high	Y	Y	PN	Y	high	high	Y	Y	N	N/A	Y	low	low	high
405	Hsin-Hung Liu, 2023	PY	PY	PY	high	high	Y	Y	PN	Y	high	high	Y	Y	PN	Y	high	high	Y	Y	NI	Y	Y	NI	NI	high
407	Xiaochi Lu, 2024	PY	PY	PY	high	high	Y	Y	PN	Y	high	high	Y	Y	PN	Y	high	high	Y	Y	Y	PN	Y	high	high	high
	Chi, Cheng	Y	Y	PY	high	high	Y	NI	Y	Y	NI	high	Y	Y	Y	Y	high	high	N	Y	PN	N/A	Y	low	low	high

Quality and concerns about applicability for each study using PROBAST+AI Checklist																									
**ID**	**Study**	**1.1**	**1.2**	**1.3**	**1A**	**1B**	**2.1**	**2.2**	**2.3**	**2.4**	**2A**	**2B**	**3.1**	**3.2**	**3.3**	**3.4**	**3A**	**3B**	**4.1**	**4.2**	**4.3**	**4.4**	**4.5**	**4A**	**Overall concern regarding quality of the prediction model development**	**Overall concern for applicability of the prediction model development**
409	Meicheng Yang, 2024	PY	PY	PY	high	high	Y	Y	PN	Y	high	high	Y	Y	PN	Y	high	high	Y	Y	Y	Y	Y	high	high	high
411	Donghwan Yun, 2021	PY	PY	PN	low	high	Y	Y	PN	Y	high	high	Y	Y	PN	Y	high	high	Y	Y	PY	N/A	Y	high	high	high
415	Yunlu Zhang, 2021	PY	PY	PY	high	high	Y	Y	PN	Y	high	high	Y	Y	PN	Y	high	high	Y	Y	Y	N/A	Y	high	high	high
417	Jiaxin Li,2024	Y	Y	Y	high	high	Y	Y	Y	Y	high	high	Y	Y	Y	Y	high	high	Y	Y	N	N/A	Y	low	low	high
421	Kipyo Kim, 2021	PY	PY	PY	high	high	Y	Y	PN	Y	high	high	Y	Y	PN	Y	high	high	Y	Y	Y	N/A	Y	high	high	high
424	Menglei Gu, 2024	PY	PY	PY	high	high	Y	Y	PN	Y	high	high	Y	Y	PN	Y	high	high	Y	Y	Y	N/A	Y	high	high	high
427	XinPei Liu, 2024	PY	PY	PY	high	high	Y	Y	PN	Y	high	high	Y	Y	PN	Y	high	high	Y	Y	Y	N/A	Y	high	high	high
430	Xuejian Hou, 2024	PY	PY	PY	high	high	Y	Y	PN	Y	high	high	Y	Y	PN	Y	high	high	Y	Y	NI	N/A	Y	low	low	high
435	Yi Du,2020	Y	PY	Y	high	high	Y	Y	PY	Y	high	high	Y	Y	Y	Y	high	high	N	PY	NI	N/A	Y	low	low	high
443	L. Nelson Sanchez-Pinto,2016	PY	PY	PY	high	high	Y	Y	PN	Y	high	high	Y	Y	PN	Y	high	high	Y	Y	PY	N/A	PY	high	high	high
446	Sang-Wook Lee, 2024	PY	PY	PY	high	high	Y	Y	PN	Y	high	high	Y	Y	PN	Y	high	high	Y	Y	PY	PN	Y	high	high	high
458	Pierre Tran, 2025	PY	PY	PY	high	high	Y	Y	PN	Y	high	high	Y	Y	PN	Y	high	high	PN	Y	Y	PN	Y	low	low	high
465	Li Zhang, 2025	PY	PY	PY	high	high	Y	Y	PN	Y	high	high	Y	Y	PN	Y	high	high	Y	Y	Y	PN	Y	high	high	high
470	Nakyung Jeon,2019	PY	PY	PY	high	high	Y	Y	PN	Y	high	high	Y	Y	PN	Y	high	high	Y	Y	Y	NI	PY	high	high	high
473	Qi Wang,2019	Y	Y	PY	high	high	Y	Y	Y	Y	high	high	Y	Y	Y	Y	high	high	Y	Y	N	N/A	Y	low	low	high
480	Xiaofei Zhang, 2025	PY	PY	PY	high	high	Y	Y	PN	Y	high	high	Y	Y	PN	N	low	high	NI	Y	Y	N/A	Y	high	high	high
485	Y Zhang, 2025	PY	PY	PY	high	high	Y	Y	PN	Y	high	high	Y	Y	PN	Y	high	high	Y	Y	Y	PN	Y	high	high	high
490	Ming Ye, 2025	PY	PY	PY	high	high	Y	Y	PN	Y	high	high	Y	Y	PN	Y	high	high	Y	Y	PY	N/A	PY	high	high	high
503	Chen Lin, 2024	PY	PY	PY	high	high	Y	PY	PN	Y	high	high	Y	Y	PN	Y	high	high	Y	Y	Y	N/A	Y	high	high	high
514	Kaori Ambe, 2025	PY	PY	PY	high	high	Y	Y	PN	Y	high	high	Y	Y	PN	Y	high	high	PN	Y	Y	PN	Y	low	low	high
515	Kate, R. J,2016	PY	PY	PY	high	high	Y	PY	PN	Y	high	high	Y	Y	PN	Y	high	high	Y	PY	PN	PN	PY	low	low	high
517	Danfeng Zha, 2022	PY	PY	PY	high	high	Y	NI	PN	Y	low	high	Y	Y	PN	Y	high	high	PN	Y	PN	N/A	Y	low	low	high
518	Ke Lin,2019	PY	PY	PN	low	high	Y	Y	PN	Y	high	high	Y	Y	PN	Y	high	high	Y	PY	PN	N/A	Y	high	low	high
519	Yang Zhou, 2023	PY	PY	PY	high	high	Y	Y	PN	Y	high	high	Y	Y	PN	Y	high	high	Y	Y	Y	N/A	Y	high	high	high
524	Huan Jing,2022	Y	PY	Y	high	high	Y	NI	PY	Y	NI	high	Y	Y	Y	Y	high	high	Y	Y	NI	N/A	Y	NI	NI	high
525	Yuxi Li, 2022	PY	PY	PY	high	high	Y	Y	PN	Y	high	high	Y	Y	PN	Y	high	high	Y	Y	NI	PN	Y	NI	NI	high
526	Hayato Akimoto, 2023	PY	PY	PY	high	high	Y	Y	PN	Y	high	high	Y	Y	PN	Y	high	high	Y	Y	Y	N/A	Y	high	high	high
	Benjamin R Griffin	PY	PY	PY	high	high	Y	PY	PN	Y	high	high	Y	Y	PN	Y	high	high	Y	Y	Y	N/A	Y	high	high	high

Quality and concerns about applicability for each study using PROBAST+AI Checklist

ID	Study	1.1	1.2	1.3	1A	1B	2.1	2.2	2.3	2.4	2A	2B	3.1	3.2	3.3	3.4	3A	3B	4.1	4.2	4.3	4.4	4.5	4A	Overall concern regarding quality of the prediction model development	Overall concern for applicability of the prediction model development
545	Jianqin He,,2019	PY	PY	PY	high	high	Y	Y	PN	Y	high	high	Y	Y	PN	Y	high	high	Y	PY	PN	N/A	PY	low	low	high
548	Yang Li, 2021	PY	PY	PY	high	high	Y	NI	PN	Y	low	high	Y	Y	PN	Y	high	high	Y	Y	PN	N/A	Y	low	low	high
556	Jiaqi Lu,2023	Y	PY	Y	high	high	Y	Y	PY	Y	high	high	Y	Y	Y	Y	high	high	Y	Y	PY	N/A	PY	high	high	high
557	Yang Li,2020	Y	Y	PY	high	high	Y	Y	PY	Y	high	high	Y	Y	Y	Y	high	high	Y	Y	PN	N/A	Y	low	low	high
562	Luyan Zheng, 2023	PY	PY	PY	high	high	Y	Y	PN	Y	high	high	Y	Y	PN	Y	high	high	Y	Y	Y	N/A	Y	high	high	high
563	Lijuan Wu, 2023	PY	PY	PY	high	high	Y	Y	PN	Y	high	high	Y	Y	PN	Y	high	high	Y	Y	Y	N/A	Y	high	high	high
568	Catalina Martin-Cleary, 2019	PY	PY	PY	high	high	Y	Y	PN	Y	high	high	Y	Y	PN	Y	high	high	Y	Y	PY	N/A	Y	high	high	high
577	Huilan Tu, 2024	PY	PY	PY	high	high	Y	Y	PN	Y	high	high	Y	Y	PN	Y	high	high	Y	Y	PN	N/A	Y	low	low	high
578	Buqing Ma,2024	Y	PY	Y	high	high	Y	Y	PY	Y	high	high	Y	Y	Y	Y	high	high	Y	Y	Y	N/A	Y	high	high	high
590	Mei Wang, 2022	PY	PY	PY	high	high	Y	NI	PN	Y	low	high	Y	Y	PN	Y	high	high	Y	Y	PN	N/A	Y	low	low	high
598	Qing Qian, 2021	PY	PY	PY	high	high	Y	Y	PN	Y	high	high	Y	Y	PN	Y	high	high	Y	Y	Y	Y	Y	high	high	high
601	Haofan Huang, 2021	PY	PY	PY	high	high	Y	Y	PN	Y	high	high	Y	Y	PN	Y	high	high	Y	Y	PN	N/A	Y	high	high	high
603	Amir Hossein Behnoush, 2024	PY	PY	PY	high	high	Y	Y	PN	Y	high	high	Y	Y	PN	Y	high	high	Y	Y	Y	N/A	Y	high	high	high
608	Daishan Jiang,2019	PY	PY	PY	high	high	Y	NI	PN	Y	low	high	Y	Y	PN	Y	high	high	Y	PY	N	N/A	PY	low	low	high
618	Lasith Adhikari,2019	PY	PY	PY	high	high	Y	Y	PN	Y	high	high	Y	Y	PN	Y	high	high	N	Y	PY	N/A	Y	low	low	high
619	Minoru Sakuragi, 2024	PY	PY	PY	high	high	Y	Y	PN	Y	high	high	Y	Y	PN	Y	high	high	N	Y	PY	N/A	N	low	low	high
623	Yang Li,2020	PY	PY	PY	high	high	Y	Y	PN	Y	high	high	Y	Y	PN	Y	high	high	Y	Y	PY	N/A	Y	high	high	high
626	Wenpeng Gao, 2022	PY	PY	PY	high	high	Y	Y	PN	Y	high	high	Y	Y	PN	Y	high	high	Y	Y	PY	N/A	Y	high	high	high
656	Kang Liu, 2025	PY	PY	PY	high	high	Y	Y	PN	Y	high	high	Y	Y	PN	Y	high	high	Y	Y	PN	N/A	Y	high	high	high
660	Simin Wu, 2023	PY	PY	PY	high	high	Y	Y	PN	Y	high	high	Y	Y	PN	Y	high	high	Y	Y	Y	N/A	Y	high	high	high
661	Sheng Shi,2023	Y	PY	Y	high	high	Y	Y	PY	Y	high	high	Y	Y	Y	Y	high	high	PY	Y	N	N/A	Y	low	low	high
665	Daniela Ponce, 2021	Y	Y	Y	high	high	Y	Y	Y	Y	high	high	Y	Y	Y	Y	high	high	Y	Y	Y	N/A	Y	high	high	high
682	Tao Sun, 2024	Y	Y	PY	high	high	Y	Y	Y	Y	high	high	Y	Y	Y	Y	high	high	Y	Y	PY	N/A	Y	high	high	high
687	Okke Nikkinen, 2022	PY	PY	PN	low	high	Y	Y	PN	Y	high	high	Y	Y	PN	Y	high	high	PN	Y	PN	N/A	Y	low	low	high
688	Chenxi Huang,2018	Y	PY	PY	high	high	Y	Y	PN	Y	high	high	Y	Y	PN	Y	high	high	Y	Y	Y	N/A	Y	high	high	high
694	Jiaqi Wang, 2022	PY	PY	PN	high	high	Y	Y	PN	Y	high	high	Y	Y	PN	Y	high	high	Y	Y	N	N/A	N	low	low	high
706	Kevin Y Heo, 2024	PY	PY	PY	high	high	Y	Y	PN	Y	high	high	Y	Y	PN	Y	high	high	Y	Y	Y	PN	Y	high	high	high
	Xinyi Cao	PY	PY	PY	high	high	Y	Y	PN	Y	high	high	Y	Y	PN	Y	high	high	Y	Y	Y	N/A	Y	high	high	high

Quality and concerns about applicability for each study using PROBAST+AI Checklist																									
**ID**	**Study**	**1.1**	**1.2**	**1.3**	**1A**	**1B**	**2.1**	**2.2**	**2.3**	**2.4**	**2A**	**2B**	**3.1**	**3.2**	**3.3**	**3.4**	**3A**	**3B**	**4.1**	**4.2**	**4.3**	**4.4**	**4.5**	**4A**	**Overall concern regarding quality of the prediction model development**	**Overall concern for applicability of the prediction model development**
724	Luyan Zheng, 2025	PY	PY	PY	high	high	Y	Y	PN	Y	high	high	Y	Y	PN	Y	high	high	PY	Y	Y	N/A	Y	high	high	high
728	Junbo Shen, 2024	PY	PY	PY	high	high	Y	Y	PN	Y	high	high	Y	Y	PN	Y	high	high	Y	Y	PN	PN	Y	low	low	high
736	Pan Pan, 2023	PY	PY	PY	high	high	Y	Y	PN	Y	high	high	Y	Y	PN	Y	high	high	Y	Y	Y	N/A	Y	high	high	high
741	Li Wang,2017	PY	PY	PY	high	high	Y	Y	PN	Y	high	high	Y	Y	PN	Y	high	high	Y	Y	PY	N/A	Y	high	high	high
750	Wenxing Peng, 2023	PY	PY	PY	high	high	Y	Y	PN	Y	high	high	Y	Y	PN	Y	high	high	Y	Y	PY	N/A	Y	high	high	high
754	Qi Xin, 2022	PY	PY	PY	high	high	Y	Y	PN	Y	high	high	Y	Y	PN	Y	high	high	Y	Y	PN	N/A	Y	low	low	high
762	Yuhei Goriki, 2021	PY	PY	PY	high	high	Y	Y	PN	Y	high	high	Y	Y	PN	Y	high	high	Y	Y	PN	N/A	Y	low	low	high
794	Hanlim Choi, 2023	PY	PY	PY	high	high	Y	Y	PN	Y	high	high	Y	Y	PN	Y	high	high	PN	Y	PN	PN	Y	low	low	high
835	Dawei Chen,2023	Y	PY	Y	high	high	Y	Y	PN	Y	high	high	Y	Y	Y	Y	high	high	Y	Y	PN	N/A	Y	low	low	high
852	Ruoran Wang, 2024	PY	PY	PY	high	high	Y	Y	PN	Y	high	high	Y	Y	PN	Y	high	high	N	Y	PN	N/A	Y	low	low	high
853	Toshiki Kuno, 2020	Y	Y	Y	high	high	Y	Y	Y	Y	high	high	Y	Y	Y	Y	high	high	Y	Y	PN	N/A	Y	low	low	high
873	Xiang Li,2024	Y	PY	Y	high	high	Y	Y	PY	Y	high	high	Y	Y	Y	Y	high	high	Y	Y	Y	N/A	Y	high	high	high
874	Ziming Jiang, 2023	PY	PY	Y	high	high	Y	Y	PN	Y	high	high	Y	Y	PN	Y	high	high	Y	Y	Y	N/A	Y	high	high	high
894	Ira S Hofer, 2022	PY	PY	PY	high	high	Y	Y	PN	Y	high	high	Y	Y	PN	Y	high	high	Y	Y	PN	N/A	Y	low	low	high
898	Hang Zhang, 2022	PY	PY	Y	high	high	Y	Y	PN	Y	high	high	Y	Y	PN	Y	high	high	Y	Y	Y	N/A	Y	high	high	high
905	Suru Yue, 2022	PY	PY	PY	high	high	Y	Y	PN	Y	high	high	Y	Y	PN	Y	high	high	Y	Y	Y	N/A	Y	high	high	high
920	Xiao Yue,2024	PY	PY	PY	high	high	PY	NI	PY	Y	high	high	Y	Y	Y	Y	high	high	Y	NI	NI	NI	NI	NI	NI	high
924	Katarzyna Krzanowska,2024	PY	PY	PY	high	high	Y	Y	PN	Y	high	high	Y	Y	PN	Y	high	high	Y	Y	Y	N/A	Y	high	high	high
932	Ping Yan,2023	PY	PY	Y	high	high	Y	Y	PN	Y	high	high	Y	Y	PN	Y	high	high	Y	Y	Y	Y	Y	high	high	high
947	Congjie Wang,2022	Y	PY	Y	high	high	Y	Y	PY	Y	high	high	Y	Y	Y	Y	high	high	PY	Y	NI	N/A	Y	low	low	high
949	Qi Xin, 2022	PY	PY	PY	high	high	Y	Y	PN	Y	high	high	Y	Y	PN	Y	high	high	Y	Y	PN	N/A	Y	low	low	high
991	Xun Wang,2021	PY	PN	PY	low	high	Y	PN	PY	Y	low	high	Y	Y	Y	Y	high	high	PY	PN	NI	N/A	Y	low	low	high
992	Jing-Cheng Peng,2021	PY	PY	Y	high	high	Y	Y	PN	Y	high	high	Y	Y	PN	Y	high	high	Y	Y	Y	N/A	PN	high	high	high
1027	Ira S. Hofer,2020	PY	PY	PY	high	high	Y	Y	PN	Y	high	high	Y	Y	PN	Y	high	high	Y	Y	PN	N/A	Y	low	low	high
1069	Kang Liu, 2025	PY	PY	Y	high	high	Y	Y	PN	Y	high	high	Y	Y	PN	Y	high	high	Y	Y	PN	N/A	Y	low	low	high
1108	Caifeng Li, 2022	PY	PY	Y	high	high	Y	Y	PN	Y	high	high	Y	Y	PN	Y	high	high	Y	Y	Y	N/A	PN	high	high	high
2814	Yufeng Jiang,2024	PY	PY	PY	high	high	Y	Y	PN	Y	high	high	Y	Y	PN	Y	high	high	Y	Y	PY	N/A	Y	high	high	high
	Shengtao Li,2024	PY	PY	PY	high	high	Y	Y	PN	Y	high	high	Y	Y	PN	Y	high	high	Y	Y	PN	N/A	Y	low	low	high

Quality and concerns about applicability for each study using PROBAST+AI Checklist																										
**ID**	**Study**	**1.1**	**1.2**	**1.3**	**1A**	**1B**	**2.1**	**2.2**	**2.3**	**2.4**	**2A**	**2B**	**3.1**	**3.2**	**3.3**	**3.4**	**3A**	**3B**	**4.1**	**4.2**	**4.3**	**4.4**	**4.5**	**4A**	**Overall concern regarding quality of the prediction model development**	**Overall concern for applicability of the prediction model development**
2956	Tao Sun, 2025	Y	Y	PY	high	high	Y	Y	Y	Y	high	high	Y	Y	Y	Y	high	high	Y	Y	PN	N/A	Y	low	low	high
3072	Xiang Li,2024	PY	PY	Y	high	high	Y	Y	PN	Y	high	high	Y	Y	Y	Y	high	high	Y	Y	Y	N/A	Y	high	high	high
3198	Junlong Hu, 2024	Y	Y	Y	high	high	Y	Y	Y	Y	high	high	Y	Y	Y	Y	high	high	Y	Y	PY	N/A	Y	high	high	high
3222	Enci Cai,2024	PY	PY	PY	high	high	Y	Y	PN	Y	high	high	Y	Y	PN	Y	high	high	Y	Y	PY	N/A	Y	high	high	high
3415	Yixin Tian,2023	PY	PY	PY	high	high	Y	Y	PN	Y	high	high	Y	Y	PN	Y	high	high	Y	Y	Y	N/A	Y	high	high	high
3417	Changwei Wu ,2023	PY	PY	PY	high	high	Y	Y	PN	Y	high	high	Y	Y	PN	Y	high	high	Y	Y	PN	PN	Y	high	high	high
3533	Lu-Huai Feng,2023	PY	PN	PY	low	high	Y	Y	PN	Y	low	high	Y	Y	Y	PN	high	high	Y	Y	PY	N/A	Y	high	low	high
3595	Hui Zhao,2022	PY	PY	PY	high	high	Y	Y	PN	Y	high	high	Y	Y	PN	Y	high	high	Y	Y	PN	N/A	Y	low	low	high
3652	Yue Hu,2022	Y	PY	Y	high	high	Y	Y	PN	Y	high	high	Y	Y	Y	Y	high	high	Y	Y	PN	N/A	Y	low	low	high
3695	Yuhui Zhang,2022	PY	PY	PY	low	high	Y	Y	PN	PN	low	low	Y	Y	Y	PN	high	high	N	Y	NI	N/A	PY	low	low	low
3942	Kai Ma,2022	Y	PY	Y	high	high	Y	Y	PN	Y	low	high	Y	Y	Y	Y	high	high	Y	Y	N	N/A	Y	low	low	high
4047	Annunziata Nusca,2021	PY	PN	PY	low	high	Y	Y	PN	Y	high	high	PY	PN	PN	Y	low	high	PN	Y	Y	N/A	Y	low	low	high
4098	Zhimeng Chen,2021	PY	PY	PY	high	high	Y	Y	PN	Y	high	high	Y	Y	PN	Y	high	high	Y	Y	PY	N/A	Y	high	high	high
4105	Jiaming Wang,2021	PY	PY	PY	high	high	Y	Y	PN	Y	high	high	Y	Y	PN	Y	high	high	Y	Y	PY	PN	Y	high	high	high
4116	Jianchao Ma,2021	Y	Y	Y	high	high	Y	Y	Y	Y	high	high	Y	Y	Y	Y	high	high	Y	Y	PN	N/A	Y	low	low	high
4160	Tingting Fan,2021	PY	PY	Y	high	high	Y	Y	PN	Y	high	high	Y	Y	PY	Y	high	high	N	Y	PY	N/A	Y	low	low	high
4228	Wang Xin ,2021	Y	Y	PY	high	high	Y	Y	PN	Y	high	high	Y	Y	PN	Y	high	high	PY	Y	PN	N/A	Y	low	low	high
4244	Hao Du,2021	PY	PY	PY	high	high	Y	Y	PN	Y	high	high	Y	Y	PN	Y	high	high	Y	Y	Y	N/A	Y	high	high	high
4277	Nina Rank,2020	PY	PY	PY	high	high	Y	Y	PN	Y	high	high	Y	Y	PN	Y	high	high	Y	Y	Y	N/A	Y	high	high	high
4352	Yang Li,2020	PY	PY	PY	high	high	Y	Y	PN	Y	high	high	Y	Y	PN	Y	high	high	Y	Y	PN	N/A	Y	low	low	high
4827	Yujun Deng,2017	Y	Y	Y	high	high	Y	PY	Y	Y	high	high	Y	Y	Y	Y	high	high	Y	Y	PN	N/A	Y	low	low	high
5064	Shu Yi Ng,,2014	PY	PY	PY	high	high	Y	NI	PN	Y	low	high	Y	Y	PN	Y	high	high	Y	PY	N	N/A	Y	low	low	high
5103	Won Ho Kim,2013	PY	PY	PY	high	high	Y	Y	PN	Y	high	high	Y	Y	PN	Y	high	high	Y	Y	N	N/A	PY	low	low	high

Note: Signalling questions are again rated as yes (Y),
probably yes (PY), probably no (PN), no (N), no information (NI), and in some cases not applicable (NA,
items marked with an asterisk)*

risk of bias of PROBAST+AI																																								
**ID**	**Study**	**1.1**	**1.2**	**1.3**	**1.A**	**1.B**	**2.1**	**2.2**	**2.3**	**2.4**	**2.A**	**2.B**	**3.1**	**3.2**	**3.3**	**3.4**	**3.A**	**3.B**	**4.1**	**4.2.A**	**4.2.I**	**4.2.E**	**4.3.A**	**4.3.I**	**4.3.E**	**4.4.A**	**4.4.I**	**4.4.E**	**4.5.A**	**4.5.I**	**4.5.E**	**4.6.A**	**4.6.I**	**4.6.E**	**4.7.A**	**4.7.I**	**4.7.E**	**4.A**	**Overall risk of bias of the prediction model evaluation**	**Overall concern for applicability of the prediction model evaluation**
4	Suru Yue, 2022	Y	PY	Y	low	high	Y	Y	Y	Y	low	high	Y	Y	Y	Y	low	high	Y	Y	Y	NA	Y	Y	NA	N/A	N/A	NA	NA	Y	NA	NA	Y	NA	NI	Y	NA	low	low	high
8	Zhiyan Fan, 2023	Y	Y	Y	low	high	Y	Y	Y	Y	low	high	Y	Y	Y	Y	low	high	Y	Y	Y	Y	Y	Y	Y	NI	PN	NA	NA	Y	NA	NA	Y	NA	NI	Y	Y	low	low	high
9	R. J. Kate,2020	PY	PY	PY	low	high	Y	Y	PN	Y	low	high	Y	Y	PN	Y	low	high	Y	Y	Y	NA	PY	PY	NA	PN	PN	NA	NA	Y	NA	NA	Y	NA	PN	PN	NA	low	low	high
11	Wanyue Zhang, 2024	Y	PY	Y	low	high	Y	Y	Y	Y	low	high	Y	Y	Y	Y	low	high	Y	Y	Y	Y	Y	Y	NI	NI	NI	NI	NA	Y	NA	NA	Y	NA	NI	Y	Y	low	low	high
12	P. Y. Tseng,2020	PY	PY	PY	low	high	Y	Y	PY	Y	low	high	Y	Y	PY	Y	low	high	Y	PN	PN	NA	Y	Y	NA	PN	PN	NA	NA	Y	NA	NA	NI	NA	PN	PN	NA	high	high	high
14	Xiao-Qin Luo, 2023	Y	PY	Y	low	high	Y	Y	Y	Y	low	high	Y	Y	PN	Y	low	high	Y	Y	Y	Y	Y	Y	Y	NI	Y	Y	NA	PN	NA	NA	PN	NA	NI	Y	Y	low	low	high
15	Qiuchong Chen, 2022	Y	PY	Y	low	high	Y	Y	Y	Y	low	high	Y	Y	PN	Y	low	high	Y	PN	PN	NA	NI	NI	NA	N/A	N/A	NA	NA	PN	NA	NA	PN	NA	NI	Y	NA	high	high	high
18	Yisong Cheng, 2022	PY	PY	Y	low	high	Y	Y	Y	Y	low	high	Y	Y	PN	Y	low	high	Y	Y	Y	NA	NI	NI	NA	N/A	N/A	NA	NA	PN	NA	NA	PN	NA	NI	Y	NA	high	high	high
26	Tianyun Gao, 2024	PY	PY	PY	low	high	Y	Y	Y	Y	low	high	Y	Y	PN	Y	low	high	Y	Y	Y	NA	Y	Y	NA	N/A	N/A	NA	NA	PN	NA	NA	PN	NA	NI	Y	NA	low	low	high
31	Shengwei Lin, 2024	PY	PY	PY	low	high	Y	Y	Y	Y	low	high	Y	Y	PN	Y	low	high	Y	NI	NI	NA	Y	Y	NA	N/A	N/A	NA	NA	PN	NA	NA	PN	NA	NI	Y	NA	high	high	high
48	Shuxing Wei, 2023	PY	PY	PY	low	high	Y	Y	Y	Y	low	high	Y	Y	PN	Y	low	high	Y	Y	Y	Y	Y	Y	Y	NI	PN	PN	NA	PN	NA	NA	PN	NA	NI	Y	Y	low	low	high
77	Rufa Zhang, 2024	PY	PY	PY	low	high	Y	Y	Y	Y	low	high	Y	Y	PN	Y	low	high	Y	PN	PN	NA	Y	Y	NA	N/A	N/A	NA	NA	Y	NA	NA	NA	NA	NI	Y	NA	high	high	high
78	Xunliang Li, 2024	PY	PY	PY	low	high	Y	Y	Y	Y	low	high	Y	Y	PN	Y	low	high	Y	Y	Y	NA	Y	Y	NA	N/A	N/A	NA	NA	Y	NA	NA	NA	NA	NI	Y	NA	low	low	high
79	Suhua She, 2023	PY	PY	PY	low	high	Y	NI	Y	Y	low	high	Y	Y	PN	Y	low	high	Y	Y	Y	NA	NI	NI	NA	N/A	N/A	NA	NA	Y	NA	NA	Y	NA	NA	Y	NA	high	high	high
83	Jianshan Shi, 2024	PY	PY	PY	low	high	Y	Y	Y	Y	low	high	Y	Y	PN	Y	low	high	Y	Y	Y	NA	Y	Y	NA	PN	PN	NA	NA	Y	NA	NA	Y	NA	NA	Y	NA	low	low	high
87	Yongbin Wang, 2024	PY	PY	PY	low	high	Y	Y	Y	Y	low	high	Y	Y	PN	Y	low	high	Y	Y	Y	Y	Y	Y	Y	NA	NA	NA	NA	Y	NA	NA	Y	NA	NA	Y	Y	low	low	high
90	Christopher T Ryan, 2023	PY	PY	PY	low	high	Y	Y	Y	Y	low	high	Y	Y	PN	Y	low	high	Y	Y	Y	NA	PY	PY	NA	Y	Y	NA	NA	Y	NA	NA	Y	NA	NA	Y	NA	low	low	high
96	Jurij Matija Kalisnik, 2022	PY	PY	PY	low	high	Y	Y	Y	Y	low	high	Y	Y	PN	Y	low	high	Y	Y	Y	NA	Y	Y	NA	NI	NI	NA	NA	Y	NA	NA	Y	NA	NA	Y	NA	NI	NI	high
98	Jin Wang, 2022	PY	PY	PY	low	high	Y	NI	Y	Y	NI	high	Y	Y	PN	Y	low	high	Y	PY	PY	NA	NI	NI	NA	PN	PN	NA	NA	Y	NA	NA	Y	NA	NA	Y	NA	low	NI	high
99	Toshiki Kuno, 2022	Y	Y	Y	low	high	Y	Y	Y	Y	low	high	Y	Y	Y	Y	low	high	Y	Y	Y	NA	Y	Y	NA	NI	NI	NA	NA	PN	NA	NA	Y	NA	NA	Y	NA	high	high	high
101	Hao Cui, 2021	Y	Y	Y	low	high	Y	Y	Y	Y	low	high	Y	Y	Y	Y	low	high	Y	PN	PN	NA	Y	Y	NA	NI	NI	NA	NA	Y	NA	NA	Y	NA	NA	Y	NA	high	high	high
102	Hongshan Zhou, 2023	PY	PY	PY	low	high	Y	Y	Y	Y	low	high	Y	Y	PN	Y	low	high	Y	Y	Y	Y	PY	PY	PY	NA	NA	NA	NA	Y	NA	NA	NA	NA	NA	Y	Y	low	low	high
109	Azar Ejmalian, 2022	PY	PY	PY	low	high	Y	Y	Y	Y	low	high	Y	Y	PN	Y	low	high	Y	Y	Y	NA	Y	Y	NA	Y	Y	NA	NA	PN	NA	NA	Y	NA	NA	Y	NA	high	high	high
136	Luming Zhang, 2022	PY	PY	PY	low	high	Y	Y	Y	Y	low	high	Y	Y	PN	Y	low	high	Y	Y	Y	Y	Y	Y	N	NA	NA	NA	NA	Y	NA	NA	Y	NA	NA	Y	Y	low	low	high
137	Chang Hu, 2022	PY	PY	PY	low	high	Y	Y	Y	Y	low	high	Y	Y	PN	Y	low	high	Y	Y	Y	NA	Y	Y	NA	N/A	N/A	NA	NA	Y	NA	NA	NA	NA	NA	Y	NA	low	low	high
140	Kaidi Gong, 2021	PY	PY	PY	low	high	Y	Y	Y	Y	low	high	Y	Y	PN	Y	low	high	Y	Y	Y	NA	Y	Y	NA	N/A	N/A	NA	NA	Y	NA	NA	Y	NA	NA	Y	NA	low	low	high
141	Yamin Liu, 2024	PY	PY	PY	low	high	Y	Y	Y	Y	low	high	Y	Y	PN	Y	low	high	Y	Y	Y	Y	Y	Y	Y	NA	NA	NA	NA	PN	NA	NA	Y	NA	NA	Y	Y	low	low	high
153	Jicheng Jiang, 2023	PY	PY	PY	low	high	Y	Y	Y	Y	low	high	Y	Y	PN	Y	low	high	Y	Y	Y	NA	Y	Y	NA	N/A	N/A	NA	NA	PN	NA	NA	NA	NA	NA	Y	NA	low	low	high
157	Jun Liu, 2022	PY	PY	PY	low	high	Y	Y	Y	Y	low	high	Y	Y	PN	Y	low	high	Y	PN	PN	NA	Y	Y	NA	N/A	N/A	NA	NA	PN	NA	NA	PN	NA	NA	Y	NA	high	high	high
158	Yun Yan, 2023	PY	PY	PY	low	high	Y	Y	Y	Y	low	high	Y	Y	PN	Y	low	high	Y	Y	Y	NA	Y	Y	NA	N/A	N/A	NA	NA	Y	NA	NA	Y	NA	NA	Y	NA	low	low	high
163	Tianchen Jia, 2023	PY	PY	PY	low	high	Y	Y	Y	Y	low	high	Y	Y	PN	Y	low	high	Y	Y	Y	Y	NI	NI	NI	NA	Y	NA	NA	Y	NA	NA	Y	NA	NA	Y	Y	low	low	high
164	Yirui Hu, 2022	PY	PY	PY	low	high	Y	Y	Y	Y	low	high	Y	Y	PN	Y	low	high	Y	Y	Y	NA	Y	Y	NA	N/A	N/A	NA	NA	Y	NA	NA	NA	NA	NA	Y	NA	low	low	high
169	Amanda C Filiberto, 2021	PY	PY	PY	low	high	Y	NI	Y	Y	low	high	Y	Y	PN	Y	low	high	Y	PN	PN	NA	NI	NI	NA	N/A	N/A	NA	NA	PN	NA	NA	NA	NA	NA	Y	NA	high	high	high
170	Xi Peng, 2022	PY	PY	PY	low	high	Y	Y	Y	Y	low	high	Y	Y	PN	Y	low	high	Y	Y	Y	Y	Y	Y	Y	NA	NA	NA	NA	PN	NA	NA	NA	NA	NA	Y	Y	low	low	high
173	Yuchen Gao, 2023	PY	PY	PY	low	high	Y	Y	Y	Y	low	high	Y	Y	PN	Y	low	high	Y	Y	Y	NA	Y	Y	NA	N/A	N/A	NA	NA	PN	NA	NA	NA	NA	NA	Y	NA	low	low	high
174	Rao Sun,2024	Y	PY	Y	low	high	Y	Y	Y	Y	low	high	Y	Y	Y	Y	low	high	Y	Y	Y	Y	Y	Y	Y	NA	NA	NA	NA	Y	NA	NA	Y	NA	NA	Y	Y	low	low	high
175	Lei Dong, 2024	PY	PY	PY	low	high	Y	Y	Y	Y	low	high	Y	Y	PN	Y	low	high	Y	Y	Y	Y	PY	PY	NA	NA	NA	NA	NA	PN	NA	NA	NA	NA	NA	Y	Y	low	low	high
176	Rima S Zahr, 2025	PY	PY	PY	low	high	Y	PY	Y	Y	low	high	Y	Y	PN	Y	low	high	Y	N	N	NA	PY	PY	NA	Y	Y	NA	NA	Y	NA	NA	Y	NA	NA	Y	NA	high	high	high
178	Sunho Ko, 2022	PY	PY	PY	low	high	Y	Y	Y	Y	low	high	Y	Y	PN	Y	low	high	Y	Y	Y	Y	PN	PN	PN	NA	NA	NA	NA	NA	NA	NA	NA	NA	Y	Y	Y	low	low	high
181	Yuezi Song, 2024	PY	PY	PY	low	high	Y	Y	Y	Y	low	high	Y	Y	PN	Y	low	high	Y	N	N	NA	Y	Y	NA	N/A	N/A	NA	NA	Y	NA	NA	Y	NA	Y	Y	NA	high	high	high
182	Sasha Pierre-Paul, 2022	PY	PY	PY	low	high	Y	Y	Y	Y	low	high	Y	Y	PN	Y	low	high	Y	PN	PN	NA	NI	NI	NA	N/A	N/A	NA	NA	Y	NA	NA	Y	NA	Y	Y	NA	high	high	high
	Jie Tang	PY	PY	PY	low	high	Y	Y	Y	Y	low	high	Y	Y	PN	Y	low	high	Y	Y	Y	NA	PY	PY	NA	N/A	N/A	NA	NA	Y	NA	NA	NA	NA	Y	Y	NA	low	low	high

risk of bias of PROBAST+AI																																									
ID	Study	1.1	1.2	1.3	1.A	1.B	2.1	2.2	2.3	2.4	2.A	2.B	3.1	3.2	3.3	3.4	3.A	3.B	4.1	4.2.A	4.2.I	4.2.E	4.3.A	4.3.I	4.3.E	4.4.A	4.4.I	4.4.E	4.5.A	4.5.I	4.5.E	4.6.A	4.6.I	4.6.E	4.7.A	4.7.I	4.7.E	4.A	Overall risk of bias of the prediction model evaluation	Overall concern for applicability of the prediction model evaluation
185	Xunliang Li, 2023	PY	PY	PY	low	high	Y	Y	Y	Y	low	high	Y	Y	PN	Y	low	high	Y	Y	Y	NA	Y	Y	NA	N/A	N/A	NA	NA	Y	NA	NA	NA	NA	Y	Y	NA	low	low	high
186	Yelena Petrosyan, 2022	PY	PY	PY	low	high	Y	Y	Y	Y	low	high	Y	Y	PN	Y	low	high	Y	Y	Y	NA	NI	NI	NA	N/A	N/A	NA	NA	Y	NA	NA	NA	NA	Y	Y	NA	low	low	high
187	C. Qu,2020	PY	PY	PY	low	high	Y	Y	Y	Y	low	high	Y	Y	PN	Y	low	high	Y	PN	PN	NA	PN	PN	NA	N/A	N/A	NA	NA	Y	NA	NA	Y	NA	PN	PN	NA	high	high	high
188	Lei Yuan, 2024	PY	PY	PY	low	high	Y	Y	Y	Y	low	high	Y	Y	Y	Y	low	high	Y	Y	Y	Y	NI	NI	NI	Y	Y	Y	NA	Y	NA	NA	Y	NA	NA	NA	NA	high	high	high
189	Yefeng Tong, 2023	PY	PY	PY	low	high	Y	Y	Y	Y	low	high	Y	Y	PN	Y	low	high	Y	Y	Y	NA	NI	NI	NA	N/A	N/A	NA	NA	Y	NA	NA	Y	NA	Y	Y	NA	low	low	high
192	Qian Li, 2023	PY	PY	PY	low	high	Y	Y	Y	Y	low	high	Y	Y	PN	Y	low	high	Y	Y	Y	Y	Y	Y	Y	NI	NI	NI	NA	Y	NA	NA	Y	NA	Y	Y	Y	low	low	high
199	Emma Schwager, 2024	PY	PY	PY	low	high	Y	Y	Y	Y	low	high	Y	Y	PN	Y	low	high	Y	Y	Y	NA	Y	Y	NA	N/A	N/A	NA	NA	Y	NA	NA	Y	NA	Y	Y	NA	low	low	high
201	Guiyu Lei,2020	PY	PY	PY	low	high	Y	NI	PN	Y	high	high	Y	Y	PN	Y	low	high	Y	Y	Y	NA	NI	NI	NA	N/A	N/A	NA	NA	Y	NA	NA	Y	NA	PN	PN	NA	high	high	high
207	Zhi Xiang Du,2022	PY	PY	PN	low	low	Y	Y	PY	Y	low	high	Y	Y	Y	Y	low	high	Y	N	N	NA	NI	NI	NA	N/A	N/A	NA	NA	NA	NA	NA	PY	NA	NA	PY	NA	high	high	high
211	Rui Fan, 2023	PY	PY	PY	low	high	Y	Y	PY	Y	low	high	Y	Y	PN	Y	low	high	Y	Y	Y	Y	Y	Y	Y	NI	NI	NI	NA	NA	NA	NA	Y	NA	Y	Y	Y	low	low	high
212	Caitlyn Chiofolo,2019	PY	PY	PY	low	high	Y	NI	PN	Y	high	high	Y	Y	Y	Y	low	high	Y	Y	Y	NA	N	N	NA	N/A	N/A	NA	NA	Y	NA	NA	Y	NA	Y	Y	NA	high	high	high
216	Charat Thongprayoon, 2022	PY	PY	PY	low	high	Y	Y	Y	Y	low	high	Y	Y	PN	Y	low	high	Y	Y	Y	NA	PN	PN	NA	N/A	N/A	NA	NA	Y	NA	NA	Y	NA	Y	Y	NA	low	low	high
217	Yeonhee Lee, 2021	PY	PY	PY	low	high	Y	NI	Y	Y	NI	high	Y	Y	PN	Y	low	high	Y	Y	Y	NA	NI	NI	NA	N/A	N/A	NA	NA	Y	NA	NA	Y	NA	Y	Y	NA	low	NI	high
219	Xian Zeng, 2022	PY	PY	PY	low	high	Y	Y	Y	Y	low	high	Y	Y	PN	Y	low	high	Y	Y	Y	NA	Y	Y	NA	Y	Y	NA	NA	Y	NA	NA	Y	NA	Y	Y	NA	low	low	high
220	Yi Yang,2022	Y	PY	Y	low	high	Y	Y	Y	Y	low	high	Y	Y	Y	Y	low	high	Y	N	N	NA	PY	PY	NA	N/A	N/A	NA	NA	Y	NA	NA	NA	NA	NA	PY	NA	high	high	high
222	Wen Tao Liu, 2022	PY	PY	PY	low	high	Y	Y	Y	Y	low	high	Y	Y	PN	Y	low	high	Y	Y	Y	NA	PY	PY	NA	N/A	N/A	NA	NA	Y	NA	NA	Y	NA	Y	Y	NA	low	low	high
226	Fangfang Zhou, 2023	PY	PY	PY	low	high	Y	Y	Y	Y	low	high	Y	Y	PN	Y	low	high	Y	Y	Y	NA	PY	PY	NA	Y	Y	NA	NA	Y	NA	NA	Y	NA	Y	Y	NA	low	low	high
227	Tingting Fan, 2023	PY	PY	PY	low	high	Y	Y	Y	Y	low	high	Y	Y	PN	Y	low	high	Y	Y	Y	NA	PY	PY	NA	N/A	N/A	NA	NA	Y	NA	NA	Y	NA	Y	Y	NA	low	low	high
230	Mengqing Ma, 2023	PY	PY	PY	low	high	Y	Y	Y	Y	low	high	Y	Y	PN	Y	low	high	Y	Y	Y	Y	Y	Y	Y	Y	Y	Y	NA	Y	NA	NA	Y	NA	Y	Y	Y	low	low	high
232	Amir Kamel Rahimi, 2023	PY	PY	PY	low	high	Y	NI	Y	Y	NI	high	Y	Y	PN	Y	low	high	Y	Y	Y	NA	Y	Y	NA	Y	Y	NA	NA	Y	NA	NA	Y	NA	Y	Y	NA	low	NI	high
233	Xiaohong Zhang, 2022	PY	PY	PY	low	high	Y	Y	Y	Y	low	high	Y	Y	PN	Y	low	high	Y	Y	Y	Y	Y	Y	Y	NI	NI	NI	NA	Y	NA	NA	NI	NA	Y	Y	Y	low	low	high
236	Chun-Te Huang, 2023	Y	PY	Y	low	high	Y	Y	Y	Y	low	high	Y	Y	PN	Y	low	high	Y	Y	Y	Y	NI	NI	NI	NI	NI	NI	NA	Y	NA	NA	Y	NA	PN	Y	Y	low	low	high
237	Jahan C. Penny-Dimri, 2020	PY	PY	PY	low	high	Y	Y	Y	Y	low	high	Y	Y	PN	Y	low	high	Y	Y	Y	NA	N	N	NA	N/A	N/A	NA	NA	Y	NA	NA	Y	NA	Y	Y	NA	high	high	high
239	Yan Luo, 2023	PY	PY	PY	low	high	Y	Y	Y	Y	low	high	Y	Y	PN	Y	low	high	Y	Y	Y	NA	Y	Y	NA	N	N	NA	NA	Y	NA	NA	Y	NA	Y	Y	NA	high	high	high
240	Takaya Okawa, 2022	PY	PY	PY	low	high	Y	Y	Y	Y	low	high	Y	Y	PN	Y	low	high	Y	Y	Y	NA	N	N	NA	N	N	NA	NA	Y	NA	NA	Y	NA	Y	Y	NA	high	high	high
241	Khaled Shawwa, 2020	PY	PY	PY	low	high	Y	PY	Y	Y	low	high	Y	Y	PN	Y	low	high	Y	Y	Y	Y	NI	NI	NI	NI	NI	NI	NA	Y	NA	NA	Y	NA	Y	Y	Y	low	low	high
245	Hsin-Hsiung Chang, 2022	PY	PY	PY	low	high	Y	Y	Y	Y	low	high	Y	Y	PN	Y	low	high	Y	Y	Y	Y	Y	Y	Y	NI	NI	NI	NA	Y	NA	NA	Y	NA	Y	Y	Y	low	low	high
246	Dabei Cai, 2022	PY	PY	PY	low	high	Y	PY	Y	Y	low	high	Y	Y	PN	Y	low	high	Y	Y	Y	Y	Y	Y	Y	Y	Y	Y	NA	Y	NA	NA	Y	NA	Y	Y	Y	low	low	high
250	Siavash Zamirpour, 2023	PY	PY	PY	low	high	Y	Y	Y	Y	low	high	Y	Y	PN	Y	low	high	Y	Y	Y	NA	PY	PY	NA	N/A	N/A	NA	NA	Y	NA	NA	Y	NA	Y	Y	NA	low	low	high
253	Ling Sun,2020	PY	PY	PY	low	high	Y	Y	Y	Y	low	high	Y	Y	PN	Y	low	high	Y	Y	Y	NA	PY	PY	NA	PN	PN	NA	NA	Y	NA	NA	NI	NA	PN	PN	NA	high	high	high
256	Teddy Lazebnik, 2022	PY	PY	PY	low	high	Y	Y	Y	Y	low	high	Y	Y	PN	Y	low	high	Y	Y	Y	NA	PN	PN	NA	N/A	N/A	NA	NA	Y	NA	NA	Y	NA	Y	Y	NA	high	high	high
259	Jialin Liu, 2021	PY	PY	PY	low	high	Y	Y	Y	Y	low	high	Y	Y	PN	Y	low	high	Y	Y	Y	NA	PN	PN	NA	N/A	N/A	NA	NA	NA	NA	NA	Y	NA	Y	Y	NA	high	high	high
262	Yu Zhang,2022	Y	PY	Y	low	high	Y	Y	PY	Y	low	high	Y	Y	Y	Y	low	high	Y	Y	Y	NA	PN	PN	NA	N/A	N/A	NA	NA	Y	NA	NA	PY	NA	NA	Y	NA	high	high	high
263	Huanhuan Shi, 2023	PY	PY	PY	low	high	Y	PY	Y	Y	low	high	Y	Y	PN	Y	low	high	Y	Y	Y	Y	PN	PN	PN	NI	NI	NI	NA	Y	NA	NA	Y	NA	Y	Y	Y	high	high	high
264	Jun Wei, 2024	PY	PY	PY	low	high	Y	Y	Y	Y	low	high	Y	Y	PN	Y	low	high	Y	Y	Y	Y	Y	Y	Y	Y	Y	Y	NA	Y	NA	NA	Y	NA	Y	Y	Y	low	low	high
272	Muying Wu, 2023	PY	PY	PN	high	high	Y	Y	Y	Y	low	high	Y	Y	PN	Y	low	high	Y	Y	Y	NA	Y	Y	NA	PN	PN	NA	NA	Y	NA	NA	Y	NA	Y	Y	NA	high	high	high
283	Shanbing Hou,2024	PY	PY	PN	high	high	Y	Y	PY	Y	low	high	Y	Y	Y	Y	low	high	Y	Y	Y	NA	N	N	NA	N/A	N/A	NA	NA	NA	NA	NA	PY	NA	NA	Y	NA	high	high	high
284	Hai-Xia Liu, 2024	PY	PY	PY	low	high	Y	Y	Y	Y	low	high	Y	Y	PN	Y	low	high	Y	Y	Y	Y	Y	Y	Y	NI	NI	NI	NA	Y	NA	NA	Y	NA	Y	Y	Y	low	low	high
285	Zeng-Lei He, 2020	PY	PY	PN	high	high	Y	Y	Y	Y	low	high	Y	Y	PN	Y	low	high	Y	N	N	NA	NI	NI	NA	N/A	N/A	NA	NA	Y	NA	NA	Y	NA	Y	Y	NA	high	high	high
286	Li Xinsai, 2022	PY	PY	PN	high	high	Y	Y	Y	Y	low	high	Y	Y	PN	Y	low	high	Y	N	N	NA	PN	PN	NA	N/A	N/A	NA	NA	Y	NA	NA	Y	NA	Y	Y	NA	high	high	high
289	Yihan Zhang, 2021	PY	PY	PY	low	high	Y	Y	Y	Y	low	high	Y	Y	PN	Y	low	high	Y	Y	Y	Y	Y	Y	Y	NI	NI	NI	NA	Y	NA	NA	Y	NA	Y	Y	NA	low	low	high
	Le Li	PY	PY	PY	low	high	Y	Y	PN	Y	low	high	Y	Y	PN	Y	low	high	Y	Y	Y	Y	Y	Y	Y	NI	NI	NI	NA	Y	NA	NA	Y	NA	Y	Y	Y	low	low	high

risk of bias of PROBAST+AI																																								
ID	Study	1.1	1.2	1.3	1.A	1.B	2.1	2.2	2.3	2.4	2.A	2.B	3.1	3.2	3.3	3.4	3.A	3.B	4.1	4.2.A	4.2.I	4.2.E	4.3.A	4.3.I	4.3.E	4.4.A	4.4.I	4.4.E	4.5.A	4.5.I	4.5.E	4.6.A	4.6.I	4.6.E	4.7.A	4.7.I	4.7.E	4.A	Overall risk of bias of the prediction model evaluation	Overall concern for applicability of the prediction model evaluation
295	Jijun Yang, 2023	PY	PY	PY	low	high	Y	Y	Y	Y	low	high	Y	Y	PN	Y	low	high	Y	Y	Y	NA	Y	Y	NA	Y	Y	NA	NA	Y	NA	NA	Y	NA	Y	Y	NA	low	low	high
300	Jiakang Shao, 2023	PY	PY	PY	low	high	Y	Y	Y	Y	low	high	Y	Y	PN	Y	low	high	Y	Y	Y	Y	N	N	N	Y	Y	Y	NA	NA	NA	NA	Y	NA	Y	Y	Y	high	high	high
302	Kun Zhu,2018	PY	PY	PY	low	high	Y	Y	PN	Y	low	high	Y	Y	PN	Y	low	high	Y	N	N	NA	PY	PY	NA	N/A	N/A	NA	NA	Y	NA	NA	Y	NA	PN	PN	NA	high	high	high
303	Changho Han, 2024	PY	PY	PY	low	high	Y	Y	PN	Y	low	high	Y	Y	PN	Y	low	high	Y	NI	NI	NA	NI	NI	NA	N/A	N/A	NA	NA	Y	NA	NA	Y	NA	Y	Y	NA	high	high	high
307	Shengqiang Chi, 2023	PY	PY	PY	low	high	NI	N	PN	Y	high	high	Y	Y	PN	Y	low	high	Y	NI	NI	NA	PN	PN	NA	N/A	N/A	NA	NA	Y	NA	NA	Y	NA	Y	Y	NA	low	low	high
308	Dongliang Yang,2022	Y	PY	Y	low	high	Y	NI	PY	Y	NI	high	Y	Y	Y	Y	low	high	Y	Y	Y	NA	N	N	NA	N/A	N/A	NA	NA	Y	NA	NA	NI	NA	Y	Y	NA	low	NI	high
309	Anran Dai, 2023	PY	PY	PY	low	high	Y	Y	PY	Y	low	high	Y	Y	PN	Y	low	high	Y	Y	Y	NA	Y	Y	NA	N/A	N/A	NA	NA	Y	NA	NA	Y	NA	Y	Y	NA	low	low	high
311	Xiao-Qin Luo, 2022	PY	PY	PY	low	high	Y	Y	PN	Y	low	high	Y	Y	PN	Y	low	high	Y	Y	Y	Y	Y	Y	Y	NI	NI	NI	NA	Y	NA	NA	Y	NA	Y	Y	Y	low	low	high
321	Yen-Yu Chen, 2023	PY	PY	PN	high	high	Y	Y	PN	Y	low	high	Y	Y	PN	Y	low	high	Y	Y	Y	NA	PN	PN	NA	PN	PN	NA	NA	Y	NA	NA	Y	NA	Y	Y	NA	high	high	high
332	Ling Pan, 2023	PY	PY	PY	low	high	Y	NI	PY	Y	NI	high	Y	Y	PN	Y	low	high	Y	Y	Y	NA	NI	NI	NA	N/A	N/A	NA	NA	Y	NA	NA	Y	NA	Y	Y	NA	low	NI	high
336	Yu Tian,, 2021	PY	PY	Y	low	high	Y	Y	PN	Y	low	high	Y	Y	PN	Y	low	high	Y	Y	Y	NA	PY	PY	NA	N/A	N/A	NA	NA	Y	NA	NA	NA	NA	Y	Y	NA	low	low	high
341	Lingyu Xu, 2024	PY	PY	PY	low	high	Y	Y	PN	Y	low	high	Y	Y	PN	Y	low	high	Y	Y	Y	NA	Y	Y	NA	PN	PN	NA	NA	Y	NA	NA	Y	NA	Y	Y	NA	low	low	high
344	Mee Yeon Lee, 2024	PY	PY	Y	low	high	Y	Y	PN	Y	low	high	PY	Y	PN	Y	low	high	Y	Y	Y	NA	PY	PY	NA	N/A	N/A	NA	NA	Y	NA	NA	Y	NA	Y	Y	NA	low	low	high
347	Hyung-Chul Lee,2018	PY	PY	PY	low	high	Y	Y	PN	Y	low	high	Y	Y	PN	Y	low	high	Y	Y	Y	NA	Y	Y	NA	PN	PN	NA	NA	Y	NA	NA	Y	NA	PN	PN	NA	low	low	high
351	JOSHUA PARRECO,2019	PY	PY	PN	low	high	Y	NI	PN	Y	high	high	Y	Y	PN	Y	low	high	Y	Y	Y	NA	N	N	NA	PN	PN	NA	NA	Y	NA	NA	Y	NA	PN	PN	NA	high	high	high
352	Wenjuan Zhang, 2024	PY	PY	PY	low	high	Y	NI	PN	Y	low	high	Y	Y	PN	Y	low	high	Y	N	N	NA	NI	NI	NA	N/A	N/A	NA	NA	Y	NA	NA	Y	NA	Y	Y	NA	high	high	high
362	Chien-Ning Hsu,2020	PY	PY	PY	low	high	Y	Y	PN	Y	low	high	Y	Y	PN	Y	low	high	Y	Y	Y	NA	PY	PY	NA	N/A	N/A	NA	NA	Y	NA	NA	Y	NA	PN	PN	NA	low	low	high
363	Mengqing Ma, 2024	PY	PY	PY	low	high	Y	Y	PN	Y	low	high	Y	Y	PN	Y	low	high	Y	Y	Y	Y	Y	Y	Y	Y	Y	Y	NA	Y	NA	NA	Y	NA	Y	Y	Y	low	low	high
366	Xiran Peng, 2024	Y	Y	PY	low	high	Y	Y	Y	Y	low	high	Y	Y	Y	Y	low	high	Y	Y	Y	NA	NI	NI	NA	N/A	N/A	NA	NA	Y	NA	NA	Y	NA	Y	Y	NA	high	high	high
371	Xiang Yu, 2022	PY	PY	PY	low	high	Y	Y	PN	Y	low	high	Y	Y	PN	Y	low	high	Y	PN	PN	NA	Y	Y	NA	Y	Y	NA	NA	Y	NA	NA	Y	NA	Y	Y	NA	high	high	high
379	Justin Y Lu, 2022	PY	PY	PY	low	high	Y	Y	PN	Y	low	high	Y	Y	PN	Y	low	high	Y	Y	Y	Y	N	N	N	NI	NI	NI	NA	Y	NA	NA	Y	NA	Y	Y	NA	low	low	high
384	Lei Lei,2020	PY	PY	PY	low	high	Y	PY	PN	Y	low	high	Y	Y	PN	Y	low	high	Y	PN	PN	NA	PN	PN	NA	N/A	N/A	NA	NA	Y	NA	NA	Y	NA	PN	PN	NA	high	high	high
394	Lauren A Scanlon, 2021	PY	PY	PY	low	high	Y	Y	PN	Y	low	high	Y	Y	PN	Y	low	high	Y	Y	Y	NA	Y	Y	NA	Y	Y	NA	NA	Y	NA	NA	NI	NA	Y	Y	NA	low	low	high
395	Liu Li, 2023	PY	PY	PY	low	high	Y	NI	PN	Y	high	high	Y	Y	PN	Y	low	high	Y	N	N	NA	NI	NI	NA	N/A	N/A	NA	NA	Y	NA	NA	NA	NA	Y	Y	NA	high	high	high
398	Jia Tian, 2023	PY	PY	PY	low	high	Y	NI	PN	Y	high	high	Y	Y	PN	Y	low	high	Y	Y	Y	NA	N	N	NA	N/A	N/A	NA	NA	NA	NA	NA	Y	NA	Y	Y	NA	low	low	high
405	Hsin-Hung Liu, 2023	PY	PY	PY	low	high	Y	Y	PN	Y	low	high	Y	Y	PN	Y	low	high	Y	Y	Y	NA	NI	NI	NA	Y	Y	NA	NA	NA	NA	NA	Y	NA	Y	Y	NA	low	low	high
407	Xiaochi Lu, 2024	PY	PY	PY	low	high	Y	Y	PN	Y	low	high	Y	Y	PN	Y	low	high	Y	Y	Y	Y	Y	Y	Y	NI	NI	NI	NA	Y	NA	NA	Y	NA	Y	Y	Y	low	low	high
408	Chi, Cheng, 2024	Y	Y	PY	low	high	Y	NI	Y	Y	NI	high	Y	Y	Y	Y	low	high	Y	N	N	NA	PN	PN	NA	N/A	N/A	NA	NA	NA	NA	NA	NA	NA	Y	Y	NA	high	high	high
409	Meicheng Yang, 2024	PY	PY	PY	low	high	Y	Y	PN	Y	low	high	Y	Y	PN	Y	low	high	Y	Y	Y	Y	Y	Y	Y	Y	Y	Y	NA	Y	NA	NA	Y	NA	Y	PY	PY	low	low	high
411	Donghwan Yun, 2021	PY	PY	PN	high	high	Y	Y	PN	Y	low	high	Y	Y	PN	Y	low	high	Y	Y	Y	NA	PY	PY	NA	N/A	N/A	NA	NA	Y	NA	NA	Y	NA	NA	Y	NA	low	low	high
415	Yunlu Zhang, 2021	PY	PY	PY	low	high	Y	Y	PN	Y	low	high	Y	Y	PN	Y	low	high	Y	Y	Y	NA	Y	Y	NA	N/A	N/A	NA	NA	Y	NA	NA	NA	NA	PN	NA	NA	low	low	high
417	Jiaxin Li,2024	Y	Y	Y	low	high	Y	Y	Y	Y	low	high	Y	Y	Y	Y	low	high	Y	Y	Y	Y	N	N	N	NI	NI	NI	NA	NI	NA	NA	NI	NA	Y	Y	Y	low	low	high
421	Kipyo Kim, 2021	PY	PY	PY	low	high	Y	Y	PN	Y	low	high	Y	Y	PN	Y	low	high	Y	Y	Y	Y	Y	Y	Y	Y	Y	Y	NA	Y	NA	NA	Y	NA	Y	Y	Y	low	low	high
424	Menglei Gu, 2024	PY	PY	PY	low	high	Y	Y	PN	Y	low	high	Y	Y	PN	Y	low	high	Y	Y	Y	Y	Y	Y	Y	NI	NI	NI	NA	NI	NA	NA	NI	NA	Y	NI	Y	low	low	high
427	XinPei Liu, 2024	PY	PY	PY	low	high	Y	Y	PN	Y	low	high	Y	Y	PN	Y	low	high	Y	Y	Y	NA	Y	Y	NA	N/A	N/A	NA	NA	Y	NA	NA	NI	NA	Y	Y	NA	low	low	high
430	Xuejian Hou, 2024	PY	PY	PY	low	high	Y	Y	PN	Y	low	high	Y	Y	PN	Y	low	high	Y	Y	Y	NA	NI	NI	NA	N/A	N/A	NA	NA	Y	NA	NA	NI	NA	Y	Y	NA	low	low	high
435	Yi Du,2020	Y	PY	Y	low	high	Y	Y	PY	Y	low	high	Y	Y	Y	Y	low	high	Y	N	N	NA	NI	NI	NA	N/A	N/A	NA	NA	Y	NA	NA	NI	NA	Y	Y	NA	high	high	high
443	L. Nelson Sanchez-Pinto,2016	PY	PY	PY	low	high	Y	Y	PN	Y	low	high	Y	Y	PN	Y	low	high	Y	Y	Y	NA	PY	PY	NA	N/A	N/A	NA	NA	Y	NA	NA	Y	NA	Y	Y	NA	low	low	high
446	Sang-Wook Lee, 2024	PY	PY	PY	low	high	Y	Y	PN	Y	low	high	Y	Y	PN	Y	low	high	Y	Y	Y	Y	PY	PY	Y	Y	Y	Y	NA	Y	NA	NA	Y	NA	Y	Y	Y	low	low	high
458	Pierre Tran, 2025	PY	PY	PY	low	high	Y	Y	PN	Y	low	high	Y	Y	PN	Y	low	high	Y	PN	PN	NA	Y	Y	NA	PN	PN	NA	NA	Y	NA	NA	Y	NA	PN	PN	NA	high	high	high
465	Li Zhang, 2025	PY	PY	PY	low	high	Y	Y	PN	Y	low	high	Y	Y	PN	Y	low	high	Y	Y	Y	Y	Y	Y	Y	Y	Y	Y	NA	NI	NA	NA	Y	NA	Y	Y	Y	low	low	high
	Nakyung Jeon,2019	PY	PY	PY	low	high	Y	Y	PN	Y	low	high	Y	Y	PN	Y	low	high	Y	Y	Y	NA	Y	Y	NA	NI	NI	NA	NA	NI	NA	NA	Y	NA	PN	PN	NA	low	low	high

risk of bias of PROBAST+AI

ID	Study	1.1	1.2	1.3	1.A	1.B	2.1	2.2	2.3	2.4	2.A	2.B	3.1	3.2	3.3	3.4	3.A	3.B	4.1	4.2.A	4.2.I	4.2.E	4.3.A	4.3.I	4.3.E	4.4.A	4.4.I	4.4.E	4.5.A	4.5.I	4.5.E	4.6.A	4.6.I	4.6.E	4.7.A	4.7.I	4.7.E	4.A	Overall risk of bias of the prediction model evaluation	Overall concern for applicability of the prediction model evaluation
473	Qi Wang,2019	Y	Y	PY	low	high	Y	Y	Y	Y	low	high	Y	Y	Y	Y	low	high	Y	Y	Y	NA	N	N	NA	N/A	N/A	NA	NA	Y	NA	NA	Y	NA	Y	Y	NA	low	low	high
480	Xiaofei Zhang, 2025	PY	PY	PY	low	high	Y	Y	PN	Y	low	high	Y	Y	PN	N	high	high	Y	Y	Y	Y	Y	Y	Y	NA	NI	NA	NA	Y	NA	NA	Y	NA	Y	Y	Y	low	low	high
485	Y Zhang, 2025	PY	PY	PY	low	high	Y	Y	PN	Y	low	high	Y	Y	PN	Y	low	high	Y	Y	Y	Y	Y	Y	Y	Y	Y	Y	NA	Y	NA	NA	Y	NA	Y	Y	Y	low	low	high
490	Ming Ye, 2025	PY	PY	PY	low	high	Y	Y	PN	Y	low	high	Y	Y	PN	Y	low	high	Y	Y	Y	NA	PY	PY	NA	N/A	N/A	NA	NA	Y	NA	NA	Y	NA	Y	Y	NA	low	low	high
503	Chen Lin, 2024	PY	PY	PY	low	high	Y	PY	PN	Y	low	high	Y	Y	PN	Y	low	high	Y	Y	Y	NA	Y	Y	NA	N/A	N/A	NA	NA	Y	NA	NA	Y	NA	Y	Y	NA	low	low	high
514	Kaori Ambe, 2025	PY	PY	PY	low	high	Y	Y	PN	Y	low	high	Y	Y	PN	Y	low	high	Y	PN	PN	NA	Y	Y	NA	PN	PN	NA	NA	Y	NA	NA	Y	NA	Y	Y	NA	high	high	high
515	Kate, R. J,2016	PY	PY	PY	low	high	Y	PY	PN	Y	low	high	Y	Y	PN	Y	low	high	Y	Y	Y	NA	PN	PN	NA	PN	PN	NA	NA	NI	NA	NA	Y	NA	PN	PN	NA	low	low	high
517	Danfeng Zha, 2022	PY	PY	PY	low	high	Y	NI	PN	Y	high	high	Y	Y	PN	Y	low	high	Y	PN	PN	NA	PN	PN	NA	N/A	N/A	NA	NA	Y	NA	NA	Y	NA	Y	Y	NA	high	high	high
518	Ke Lin,2019	PY	PY	PN	high	high	Y	Y	PN	Y	low	high	Y	Y	PN	Y	low	high	Y	Y	Y	NA	PN	PN	NA	N/A	N/A	NA	NA	NI	NA	NA	NI	NA	PN	PN	NA	low	high	high
519	Yang Zhou, 2023	PY	PY	PY	low	high	Y	Y	PN	Y	low	high	Y	Y	PN	Y	low	high	Y	Y	Y	NA	Y	Y	NA	N/A	N/A	NA	NA	Y	NA	NA	Y	NA	Y	Y	NA	low	low	high
524	Huan Jing,2022	Y	PY	Y	low	high	Y	NI	PY	Y	NI	high	Y	Y	Y	Y	low	high	Y	Y	Y	N	NI	NI	NI	NI	NI	NI	NA	Y	NA	NA	PN	NA	Y	PN	PN	high	high	high
525	Yuxi Li, 2022	PY	PY	PY	low	high	Y	Y	PN	Y	low	high	Y	Y	PN	Y	low	high	Y	Y	Y	NA	NI	NI	NA	PN	PN	NA	NA	Y	NA	NA	Y	NA	Y	Y	NA	low	low	high
526	Hayato Akimoto, 2023	PY	PY	PY	low	high	Y	Y	PN	Y	low	high	Y	Y	PN	Y	low	high	Y	Y	Y	NA	Y	Y	NA	N/A	N/A	NA	NA	Y	NA	NA	Y	NA	Y	Y	NA	low	low	high
533	Benjamin R Griffin, 2024	PY	PY	PY	low	high	Y	PY	PN	Y	low	high	Y	Y	PN	Y	low	high	Y	Y	Y	NA	Y	Y	NA	N/A	N/A	NA	NA	Y	NA	NA	Y	NA	Y	Y	NA	low	low	high
545	Jianqin He,,2019	PY	PY	PY	low	high	Y	Y	PN	Y	low	high	Y	Y	PN	Y	low	high	Y	Y	Y	NA	PN	PN	NA	N/A	N/A	NA	NA	NI	NA	NA	NI	NA	PN	PN	NA	low	low	high
548	Yang Li, 2021	PY	PY	PY	low	high	Y	NI	PN	Y	high	high	Y	Y	PN	Y	low	high	Y	Y	Y	NA	PN	PN	NA	N/A	N/A	NA	NA	Y	NA	NA	Y	NA	Y	Y	NA	high	high	high
556	Jiaqi Lu,2023	Y	PY	Y	low	high	Y	Y	PY	Y	low	high	Y	Y	Y	Y	low	high	Y	Y	Y	NA	PY	PY	NA	N/A	N/A	NA	NA	Y	NA	NA	NI	NA	PN	Y	NA	low	low	high
557	Yang Li,2020	Y	Y	PY	low	high	Y	Y	PY	Y	low	high	Y	Y	Y	Y	low	high	Y	Y	Y	Y	PN	PN	PN	NI	NI	NA	NA	Y	NA	NA	NI	NA	PN	PN	NA	low	low	high
562	Luyan Zheng, 2023	PY	PY	PY	low	high	Y	Y	PN	Y	low	high	Y	Y	PN	Y	low	high	Y	Y	Y	Y	Y	Y	Y	NA	NA	NA	NA	Y	NA	NA	Y	NA	Y	Y	Y	low	low	high
563	Lijuan Wu, 2023	PY	PY	PY	low	high	Y	Y	PN	Y	low	high	Y	Y	PN	Y	low	high	Y	Y	Y	NA	Y	Y	NA	N/A	N/A	NA	NA	Y	NA	NA	Y	NA	Y	Y	NA	low	low	high
568	Catalina Martin-Cleary, 2019	PY	PY	PY	low	high	Y	Y	PN	Y	low	high	Y	Y	PN	Y	low	high	Y	Y	Y	NA	PY	PY	NA	N/A	N/A	NA	NA	NA	NA	NA	Y	NA	PY	PY	NA	low	low	high
577	Huilan Tu, 2024	PY	PY	PY	low	high	Y	Y	PN	Y	low	high	Y	Y	PN	Y	low	high	Y	Y	Y	NA	PN	PN	NA	N/A	N/A	NA	NA	Y	NA	NA	Y	NA	Y	Y	NA	low	low	high
578	Buqing Ma,2024	Y	PY	Y	low	high	Y	Y	PY	Y	low	high	Y	Y	Y	Y	low	high	Y	Y	Y	NA	Y	Y	NA	N/A	N/A	NA	NA	Y	NA	NA	Y	NA	PN	PN	NA	low	low	high
590	Mei Wang, 2022	PY	PY	PY	low	high	Y	NI	PN	Y	high	high	Y	Y	PN	Y	low	high	Y	Y	Y	NA	PN	PN	NA	N/A	N/A	NA	NA	Y	NA	NA	Y	NA	PY	PY	NA	low	high	high
598	Qing Qian, 2021	PY	PY	PY	low	high	Y	Y	PN	Y	low	high	Y	Y	PN	Y	low	high	Y	Y	Y	NA	Y	Y	NA	Y	Y	NA	NA	Y	NA	NA	NA	NA	PY	PY	NA	low	low	high
601	Haofan Huang, 2021	PY	PY	PY	low	high	Y	Y	PN	Y	low	high	Y	Y	PN	Y	low	high	Y	Y	Y	Y	Y	Y	Y	NA	NA	NA	NA	Y	NA	NA	Y	NA	Y	Y	Y	low	low	high
603	Amir Hossein Behnoush, 2024	PY	PY	PY	low	high	Y	Y	PN	Y	low	high	Y	Y	PN	Y	low	high	Y	Y	Y	NA	Y	Y	NA	N/A	N/A	NA	NA	Y	NA	NA	Y	NA	PY	PY	NA	low	low	high
608	Daishan Jiang,2019	PY	PY	PY	low	high	Y	NI	PN	Y	high	high	Y	Y	PN	Y	low	high	N	Y	Y	NA	N	N	NA	N/A	N/A	NA	NA	NA	NA	NA	NA	NA	PN	NA	NA	high	high	high
618	Lasith Adhikari,2019	PY	PY	PY	low	high	Y	Y	PN	Y	low	high	Y	Y	PN	Y	low	high	Y	N	N	NA	PY	PY	NA	N/A	N/A	NA	NA	Y	NA	NA	NI	NA	PN	PN	NA	high	high	high
619	Minoru Sakuragi, 2024	PY	PY	PY	low	high	Y	Y	PN	Y	low	high	Y	Y	PN	Y	low	high	Y	N	N	NA	PY	PY	NA	N/A	N/A	NA	NA	Y	NA	NA	Y	NA	Y	Y	NA	high	high	high
623	Yang Li,2020	PY	PY	PY	low	high	Y	Y	PN	Y	low	high	Y	Y	PN	Y	low	high	Y	Y	Y	NA	PY	PY	NA	N/A	N/A	NA	NA	Y	NA	NA	NI	NA	PN	PN	NA	low	low	high
626	Wenpeng Gao, 2022	PY	PY	PY	low	high	Y	Y	PN	Y	low	high	Y	Y	PN	Y	low	high	Y	Y	Y	NA	PY	PY	NA	N/A	N/A	NA	NA	Y	NA	NA	Y	NA	Y	Y	NA	low	low	high
656	Kang Liu, 2025	PY	PY	PY	low	high	Y	Y	PN	Y	low	high	Y	Y	PN	Y	low	high	Y	Y	Y	Y	PN	PN	PN	NA	NA	NA	NA	Y	NA	NA	Y	NA	Y	Y	NA	low	low	high
660	Simin Wu, 2023	PY	PY	PY	low	high	Y	Y	PN	Y	low	high	Y	Y	PN	Y	low	high	Y	Y	Y	NA	Y	Y	NA	N/A	N/A	NA	NA	Y	NA	NA	NA	NA	Y	Y	NA	low	low	high
661	Sheng Shi,2023	Y	PY	Y	low	high	Y	Y	PY	Y	low	high	Y	Y	Y	Y	low	high	Y	PY	PY	NA	N	N	NA	N/A	N/A	NA	NA	Y	NA	NA	NA	NA	Y	Y	NA	high	high	high
665	Daniela Ponce, 2021	Y	Y	Y	low	high	Y	Y	Y	Y	low	high	Y	Y	Y	Y	low	high	Y	Y	Y	NA	Y	Y	NA	N/A	N/A	NA	NA	Y	NA	NA	Y	NA	Y	Y	NA	low	low	high
682	Tao Sun, 2024	Y	Y	PY	low	high	Y	Y	Y	Y	low	high	Y	Y	Y	Y	low	high	Y	Y	Y	Y	PY	PY	Y	NI	NI	NA	NA	Y	NA	NA	Y	NA	Y	Y	Y	low	low	high
687	Okke Nikkinen, 2022	PY	PY	PN	high	high	Y	Y	PN	Y	low	high	Y	Y	PN	Y	low	high	Y	PN	PN	NA	PN	PN	NA	N/A	N/A	NA	NA	Y	NA	NA	Y	NA	PY	PY	NA	high	high	high
688	Chenxi Huang,2018	Y	PY	PY	low	high	Y	Y	PN	Y	low	high	Y	Y	PN	Y	low	high	Y	Y	Y	Y	Y	Y	Y	NI	NI	NI	NA	Y	NA	NA	NI	NA	Y	Y	Y	low	low	high
694	Jiaqi Wang, 2022	PY	PY	PN	low	high	Y	Y	PN	Y	low	high	Y	Y	PN	Y	low	high	Y	Y	Y	NA	N	N	NA	N/A	N/A	NA	NA	NI	NA	NA	NI	NA	Y	NI	NA	high	high	high
706	Kevin Y Heo, 2024	PY	PY	PY	low	high	Y	Y	PN	Y	low	high	Y	Y	PN	Y	low	high	Y	Y	Y	NA	Y	Y	NA	PN	PN	NA	NA	Y	NA	NA	Y	NA	N	N	NA	high	high	high
	Xinyi Cac	PY	PY	PY	low	high	Y	Y	PN	Y	low	high	Y	Y	PN	Y	low	high	Y	Y	Y	NA	Y	Y	NA	N/A	N/A	NA	NA	Y	NA	NI	NI	NA	Y	Y	NA	low	low	high

risk of bias of PROBAST+AI																																								
ID	Study	1.1	1.2	1.3	1.A	1.B	2.1	2.2	2.3	2.4	2.A	2.B	3.1	3.2	3.3	3.4	3.A	3.B	4.1	4.2.A	4.2.I	4.2.E	4.3.A	4.3.I	4.3.E	4.4.A	4.4.I	4.4.E	4.5.A	4.5.I	4.5.E	4.6.A	4.6.I	4.6.E	4.7.A	4.7.I	4.7.E	4.A	Overall risk of bias of the prediction model evaluation	Overall concern for applicability of the prediction model evaluation
724	Luyan Zheng, 2025	PY	PY	PY	low	high	Y	Y	PN	Y	low	high	Y	Y	PN	Y	low	high	Y	PY	PY	Y	Y	Y	Y	NI	NI	NI	NA	Y	NA	NA	Y	NA	Y	Y	Y	low	low	high
728	Junbo Shen, 2024	PY	PY	PY	low	high	Y	Y	PN	Y	low	high	Y	Y	PN	Y	low	high	Y	Y	Y	NA	PN	PN	NA	PN	PN	NA	NA	Y	NA	NA	Y	NA	Y	Y	NA	high	high	high
736	Pan Pan, 2023	PY	PY	PY	low	high	Y	Y	PN	Y	low	high	Y	Y	PN	Y	low	high	Y	Y	Y	NA	Y	Y	NA	N/A	N/A	NA	NA	Y	NA	NA	Y	NA	Y	Y	NA	low	low	high
741	Li Wang,2017	PY	PY	PY	low	high	Y	Y	PN	Y	low	high	Y	Y	PN	Y	low	high	Y	Y	Y	Y	PY	PY	PY	NI	NI	NA	NA	Y	NA	NA	NA	NA	PN	PN	NA	low	low	high
750	Wenxing Peng, 2023	PY	PY	PY	low	high	Y	Y	PN	Y	low	high	Y	Y	PN	Y	low	high	Y	Y	Y	NA	PY	PY	NA	N/A	N/A	NA	NA	Y	NA	NA	Y	NA	Y	Y	NA	low	low	high
754	Qi Xin, 2022	PY	PY	PY	low	high	Y	Y	PN	Y	low	high	Y	Y	PN	Y	low	high	Y	Y	Y	NA	PN	PN	NA	N/A	N/A	NA	NA	Y	NA	NA	NI	NA	Y	Y	NA	low	low	high
762	Yuhei Goriki, 2021	PY	PY	PY	low	high	Y	Y	PN	Y	low	high	Y	Y	PN	Y	low	high	Y	Y	Y	N	PN	PN	PN	NI	NI	NA	NA	Y	NA	NA	NI	NA	Y	Y	NA	high	high	high
794	Hanlim Choi, 2023	PY	PY	PY	low	high	Y	Y	PN	Y	low	high	Y	Y	PN	Y	low	high	Y	PN	PN	NA	PN	PN	NA	PN	PN	NA	NA	Y	NA	NA	Y	NA	PY	PY	NA	high	high	high
835	Dawei Chen,2023	Y	PY	Y	low	high	Y	Y	PN	Y	low	high	Y	Y	Y	Y	low	high	Y	Y	Y	NA	PN	PN	NA	N/A	N/A	NA	NA	Y	NA	NA	Y	NA	PN	Y	NA	high	high	high
852	Ruoran Wang, 2024	PY	PY	PY	low	high	Y	Y	PN	Y	low	high	Y	Y	PN	Y	low	high	Y	N	N	NA	PN	PN	NA	N/A	N/A	NA	NA	Y	NA	NA	Y	NA	PY	PY	NA	high	high	high
853	Toshiki Kuno, 2020	Y	Y	Y	low	high	Y	Y	Y	Y	low	high	Y	Y	Y	Y	low	high	Y	Y	Y	NA	PN	PN	NA	N/A	N/A	NA	NA	NI	NA	NA	NI	NA	PY	PY	NA	high	high	high
873	Xiang Li,2024	Y	PY	Y	low	high	Y	Y	PY	Y	low	high	Y	Y	Y	Y	low	high	Y	Y	Y	NA	Y	Y	NA	N/A	N/A	NA	NA	Y	NA	NA	NI	NA	Y	Y	NA	low	low	high
874	Ziming Jiang, 2023	PY	PY	Y	low	high	Y	Y	PN	Y	low	high	Y	Y	PN	Y	low	high	Y	Y	Y	NA	Y	Y	NA	N/A	N/A	NA	NA	Y	NA	NA	NI	NA	Y	Y	NA	low	low	high
894	Ira S Hofer, 2022	PY	PY	PY	low	high	Y	Y	PN	Y	low	high	Y	Y	PN	Y	low	high	Y	Y	Y	NA	PN	PN	NA	N/A	N/A	NA	NA	Y	NA	NA	Y	NA	PY	PY	NA	high	high	high
898	Hang Zhang, 2022	PY	PY	Y	low	high	Y	Y	PN	Y	low	high	Y	Y	PN	Y	low	high	Y	Y	Y	NA	Y	Y	NA	N/A	N/A	NA	NA	Y	NA	NA	Y	NA	Y	Y	NA	low	low	high
905	Suru Yue, 2022	PY	PY	PY	low	high	Y	Y	PN	Y	low	high	Y	Y	PN	Y	low	high	Y	Y	Y	NA	Y	Y	NA	N/A	N/A	NA	NA	Y	NA	NA	NI	NA	Y	Y	NA	low	low	high
920	Xiao Yue,2024	PY	PY	PY	low	high	PY	NI	PY	Y	low	high	Y	Y	Y	Y	low	high	PN	Y	Y	NA	NI	NI	NA	NI	NI	NA	NA	Y	NA	NA	NA	NA	NA	Y	NA	high	high	high
924	Katarzyna Krzanowska,2024	PY	PY	PY	low	high	Y	Y	PN	Y	low	high	Y	Y	PN	Y	low	high	Y	Y	Y	NA	Y	Y	NA	N/A	N/A	NA	NA	Y	NA	NA	Y	NA	PY	PY	NA	low	low	high
932	Ping Yan,2023	PY	PY	Y	low	high	Y	Y	PN	Y	low	high	Y	Y	PN	Y	low	high	Y	Y	Y	Y	Y	Y	Y	Y	Y	Y	NA	Y	NA	NA	NI	NA	Y	Y	Y	low	low	high
947	Congjie Wang,2022	Y	PY	Y	low	high	Y	Y	PY	Y	low	high	Y	Y	Y	Y	low	high	Y	PY	PY	NA	NI	NI	NA	N/A	N/A	NA	NA	Y	NA	NA	NI	NA	Y	Y	NA	high	high	high
949	Qi Xin, 2022	PY	PY	PY	low	high	Y	Y	PN	Y	low	high	Y	Y	PN	Y	low	high	Y	Y	Y	N	PN	PN	PN	NI	NI	NA	NA	Y	NA	NA	NI	NA	PY	PY	NA	high	high	high
991	Xun Wang,2021	PY	PN	PY	high	high	Y	PN	PY	Y	high	high	Y	Y	Y	Y	low	high	Y	PY	PY	NA	NI	NI	NA	N/A	N/A	NA	NA	NI	NA	NA	NI	NA	Y	Y	NA	low	high	high
992	Jing-Cheng Peng,2021	PY	PY	Y	low	high	Y	Y	PN	Y	low	high	Y	Y	PN	Y	low	high	Y	Y	Y	NA	Y	Y	NA	N/A	N/A	NA	NA	Y	NA	NA	NI	NA	PY	PY	NA	low	low	high
1027	Ira S. Hofer,2020	PY	PY	PY	low	high	Y	Y	PN	Y	low	high	Y	Y	PN	Y	low	high	Y	Y	Y	NA	PN	PN	NA	N/A	N/A	NA	NA	Y	NA	NA	NI	NA	PN	PN	NA	high	high	high
1069	Kang Liu, 2025	PY	PY	Y	low	high	Y	Y	PN	Y	low	high	Y	Y	PN	Y	low	high	Y	Y	Y	NA	PN	PN	NA	N/A	N/A	NA	NA	Y	NA	NA	Y	NA	Y	Y	NA	low	low	high
1108	Caifeng Li, 2022	PY	PY	Y	low	high	Y	Y	PN	Y	low	high	Y	Y	PN	Y	low	high	Y	Y	Y	NA	Y	Y	NA	N/A	N/A	NA	NA	Y	NA	NA	Y	NA	Y	Y	NA	low	low	high
2814	Yufeng Jiang,2024	PY	PY	PY	low	high	Y	Y	PN	Y	low	high	Y	Y	PN	Y	low	high	Y	Y	Y	NA	PY	PY	NA	N/A	N/A	NA	NA	Y	NA	NA	Y	NA	Y	Y	NA	low	low	high
2905	Shengtao Li,2024	PY	PY	PY	low	high	Y	Y	PN	Y	low	high	Y	Y	PN	Y	low	high	Y	Y	Y	NA	PN	PN	NA	N/A	N/A	NA	NA	Y	NA	NA	Y	NA	Y	Y	NA	low	low	high
2956	Tao Sun, 2025	Y	Y	PY	low	high	Y	Y	Y	Y	low	high	Y	Y	Y	Y	low	high	Y	Y	Y	Y	PN	PN	PN	NI	NI	NI	NA	NI	NA	NA	NI	NA	PY	PY	PY	low	low	high
3072	Xiang Li,2024	PY	PY	Y	low	high	Y	Y	PN	Y	low	high	Y	Y	Y	Y	low	high	Y	Y	Y	NA	Y	Y	NA	N/A	N/A	NA	NA	NI	NA	NA	NI	NA	Y	Y	NA	low	low	high
3198	Junlong Hu, 2024	Y	Y	Y	low	high	Y	Y	Y	Y	low	high	Y	Y	Y	Y	low	high	Y	Y	Y	Y	PY	PY	PY	NI	NI	NI	NA	Y	NA	NA	Y	NA	Y	Y	Y	low	low	high
3222	Enci Cai,2024	PY	PY	PY	low	high	Y	Y	PN	Y	low	high	Y	Y	PN	Y	low	high	Y	Y	Y	NA	PY	PY	NA	N/A	N/A	NA	NA	Y	NA	NA	Y	NA	PN	PN	NA	low	low	high
3415	Yixin Tian,2023	PY	PY	PY	low	high	Y	Y	PN	Y	low	high	Y	Y	PN	Y	low	high	Y	Y	Y	Y	Y	Y	Y	NI	NI	NI	NA	Y	NA	NA	NI	NA	PN	Y	Y	low	low	high
3417	Changwei Wu ,2023	PY	PY	PY	low	high	Y	Y	PN	Y	low	high	Y	Y	PN	Y	low	high	Y	Y	Y	Y	PN	PN	Y	NI	NI	NI	NA	Y	NA	NA	NI	NA	PN	PN	PN	low	low	high
3533	Lu-Huai Feng,2023	PY	PN	PY	high	high	Y	Y	PN	Y	high	high	Y	Y	Y	PN	low	high	Y	Y	Y	NA	PY	PY	NA	N/A	N/A	NA	NA	Y	NA	NA	NI	NA	Y	Y	NA	low	high	high
3595	Hui Zhao,2022	PY	PY	PY	low	high	Y	Y	PN	Y	low	high	Y	Y	PN	Y	low	high	Y	Y	Y	PN	PN	PN	PN	NI	NI	NI	NI	NI	NI	NA	NI	NA	Y	NA	NA	high	high	high
3652	Yue Hu,2022	Y	PY	Y	low	high	Y	Y	PN	Y	low	high	Y	Y	Y	Y	low	high	Y	Y	Y	NA	PN	PN	NA	N/A	N/A	NA	NA	Y	NA	NA	NI	NA	PN	PN	NA	high	high	high
3695	Yuhui Zhang,2022	PY	PY	PY	high	high	Y	Y	PN	PN	high	low	Y	Y	Y	PN	low	high	N	N	NA	NA	NI	NI	NA	N/A	N/A	NA	NA	NA	NA	NA	NA	NA	PN	NA	NA	high	high	high
3942	Kai Ma,2022	Y	PY	Y	low	high	Y	Y	PN	Y	low	high	Y	Y	Y	Y	low	high	Y	Y	Y	NA	N	N	N	NI	NI	NA	NA	Y	NA	NA	NI	NA	Y	Y	NA	high	high	high
4047	Annunziata Nusca,2021	PY	PN	PY	high	high	Y	Y	PN	Y	low	high	PY	PN	PN	Y	high	high	Y	PN	PN	NA	Y	Y	NA	N/A	N/A	NA	NA	Y	NA	NA	PN	NA	PN	PN	NA	high	high	high
4098	Zhimeng Chen,2021	PY	PY	PY	low	high	Y	Y	PN	Y	low	high	Y	Y	PN	Y	low	high	Y	Y	Y	N	PY	PY	PY	NI	NI	NA	NA	Y	NA	NA	NI	NA	PN	PN	NA	high	high	high
	Jiaming Wang,2021	PY	PY	PY	low	high	Y	Y	PN	Y	low	high	Y	Y	PN	Y	low	high	Y	Y	Y	Y	PY	PY	Y	NI	NI	NI	NA	Y	NA	NA	Y	NA	PN	PN	NA	low	low	high

risk of bias of PROBAST+AI

ID	Study	1.1	1.2	1.3	1.A	1.B	2.1	2.2	2.3	2.4	2.A	2.B	3.1	3.2	3.3	3.4	3.A	3.B	4.1	4.2.A	4.2.I	4.2.E	4.3.A	4.3.I	4.3.E	4.4.A	4.4.I	4.4.E	4.5.A	4.5.I	4.5.E	4.6.A	4.6.I	4.6.E	4.7.A	4.7.I	4.7.E	4.A	Overall risk of bias of the prediction model evaluation	Overall concern for applicability of the prediction model evaluation
4116	Jianchao Ma,2021	Y	Y	Y	low	high	Y	Y	Y	Y	low	high	Y	Y	Y	Y	low	high	Y	Y	Y	N	PN	PN	PN	NA	NA	NA	NA	Y	NA	NA	NI	NA	Y	Y	NA	high	high	high
4160	Tingting Fan,2021	PY	PY	Y	low	high	Y	Y	PN	Y	low	high	Y	Y	PY	Y	low	high	Y	N	N	NA	PY	PY	NA	N/A	N/A	NA	NA	Y	NA	NA	PY	NA	Y	Y	NA	high	high	high
4228	Wang Xin ,2021	Y	Y	PY	low	high	Y	Y	PN	Y	low	high	Y	Y	PN	Y	low	high	Y	PY	PY	N	PN	PN	PN	NI	NA	NA	NA	NA	NA	NA	NI	NA	PN	NA	PN	high	high	high
4244	Hao Du,2021	PY	PY	PY	low	high	Y	Y	PN	Y	low	high	Y	Y	PN	Y	low	high	Y	Y	Y	Y	Y	Y	Y	NI	NI	NI	NA	NA	NA	NA	NI	NA	PN	PN	NA	low	low	high
4277	Nina Rank,2020	PY	PY	PY	low	high	Y	Y	PN	Y	low	high	Y	Y	PN	Y	low	high	Y	Y	Y	NA	Y	Y	NA	N/A	N/A	NA	NA	NA	NA	NA	NI	NA	Y	Y	NA	low	low	high
4352	Yang Li,2020	PY	PY	PY	low	high	Y	Y	PN	Y	low	high	Y	Y	PN	Y	low	high	Y	Y	Y	NA	PN	PN	NA	N/A	N/A	NA	NA	NA	NA	NA	NI	NA	PN	PN	NA	high	high	high
4827	Yujun Deng,2017	Y	Y	Y	low	high	Y	PY	Y	Y	low	high	Y	Y	Y	Y	low	high	Y	Y	Y	NA	PN	PN	NA	N/A	N/A	NA	NA	NA	NA	NA	Y	NA	PN	PN	NA	high	high	high
5064	Shu Yi Ng,,2014	PY	PY	PY	low	high	Y	NI	PN	Y	high	high	Y	Y	PN	Y	low	high	Y	Y	Y	NA	N	N	NA	N/A	N/A	NA	NA	Y	NA	NA	Y	NA	Y	Y	NA	high	high	high
5103	Won Ho Kim,2013	PY	PY	PY	low	high	Y	Y	PN	Y	low	high	Y	Y	PN	Y	low	high	Y	Y	Y	NA	N	N	NA	N/A	N/A	NA	NA	Y	NA	NA	NI	NA	Y	Y	NA	high	high	high

Note: Signalling questions are again rated as yes (Y),
probably yes (PY), probably no (PN), no (N), no information (NI), and in some cases not applicable (NA,
items marked with an asterisk)*

Supplementary Table S7. Complementary Model-Level Meta-analysis of Pooled AUCs by AI Model Application Type

Supplementary Table S7A: Meta−analysis of pooled AUC by ML/AI model class for AKI prediction.

Model	No. of AUC estimates (k)	Pooled AUC (HKSJ-adjusted 95% CI)	I^2 ; Cochran Q P value	Egger small-study effects test P value	HKSJ-adjusted between-group P value
Linear/generalized linear models	153	0.782 (0.769−0.794)	100.0%; P<.001	<.001	
Tree-based and ensemble methods	121	0.800 (0.785−0.814)	100.0%; P<.001	.006	
Deep learning models	48	0.784 (0.754−0.809)	100.0%; P<.001	.269	
Hybrid models and others	82	0.767 (0.750−0.784)	100.0%; P<.001	.523	
**Overall**	**404**	**0.785 (0.777−0.793)**	**100.0%; P<.001**	**<.001**	F(3,400)=2.7829; P=.041

Supplementary Table S7B: Meta−analysis of pooled AUC by ML/AI model class for mortality prediction among AKI patients.

Model	No. of AUC estimates (k)	Pooled AUC (HKSJ-adjusted 95% CI)	I^2 ; Cochran Q P value	Egger small-study effects test P value	HKSJ-adjusted between-group P value
Linear/generalized linear models	23	0.782 (0.759−0.804)	98.7%; P<.001	.220	
Tree-based and ensemble methods	24	0.804 (0.777−0.829)	99.9%; P<.001	.125	
Deep learning models	9	0.764 (0.713−0.808)	99.4%; P<.001	Not estimable	
Hybrid models and others	16	0.770 (0.721−0.813)	99.8%; P<.001	.536	
**Overall**	**72**	**0.785 (0.769−0.800)**	**99.8%; P <.001**	**.054**	F(3,68)=1.2906; P=.285

Supplementary Table S10. Sources of variance information used for AUC estimates included in quantitative pooling

This table summarizes the sources of variance information used to derive standard errors for AUC estimates included in quantitative pooling.

Outcome	Outcome-specific representative AUC records	AUC estimates included in pooling	Derived from reported 95% CI	Hanley-McNeil approximation	Derived from reported SD	Excluded because variance was not derivable
AKI occurrence	190	188	124 (66.0%)	64 (34.0%)	0 (0.0%)	2
Post-AKI mortality	31	31	21 (67.7%)	10 (32.3%)	0 (0.0%)	0
Overall	221	219	145 (66.2%)	74 (33.8%)	0 (0.0%)	2

Note. Values are shown as n (%), unless otherwise indicated. Percentages were calculated using the number of AUC estimates included in quantitative pooling for each outcome as the denominator. Standard errors were derived according to the prespecified hierarchy: reported 95% confidence intervals, followed by the Hanley-McNeil approximation based on event and non-event counts, and then reported standard deviations when available. No AUC estimates ultimately required reported standard deviations as the final variance source. Two AKI occurrence records were excluded because variance information could not be derived. AUC, area under the receiver operating characteristic curve; AKI, acute kidney injury; CI, confidence interval; SD, standard deviation.

Supplementary Figure S1. Risk-of-Bias Assessment Results Using the PROBAST+AI Tool for Included AKI Prediction Model Studies

n = 219

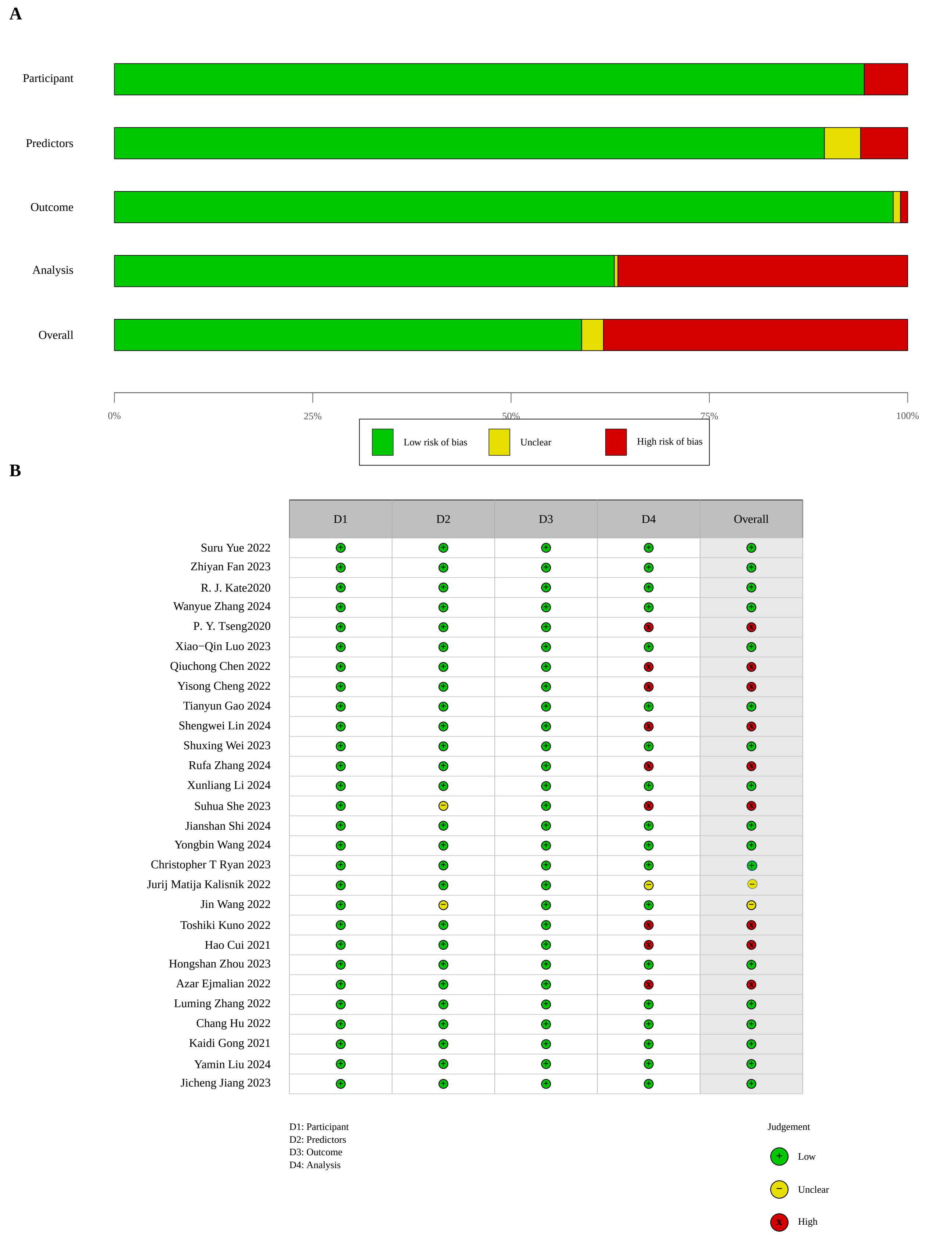

Note: (A) Summary of risk of bias assessment. (B) Traffic light plot of risk of bias assessment.

Supplementary Figure S1. Risk-of-Bias Assessment Results Using the PROBAST+AI Tool for Included AKI Prediction Model Studies

n = 219

B (continued, page 2 of 5)

	D1	D2	D3	D4	Overall
Jun Liu 2022	+	+	+	x	x
Yun Yan 2023	+	+	+	+	+
Tianchen Jia 2023	+	+	+	+	+
Yirui Hu 2022	+	+	+	+	+
Amanda C Filiberto 2021	+	−	+	x	x
Xi Peng 2022	+	+	+	+	+
Yuchen Gao 2023	+	+	+	+	+
Rao Sun2024	+	+	+	+	+
Lei Dong 2024	+	+	+	+	+
Rima S Zahr 2025	+	+	+	x	x
Sunho Ko 2022	+	+	+	+	+
Yuezi Song 2024	+	+	+	x	x
Sasha Pierre−Paul 2022	+	+	+	x	x
Jie Tang 2024	+	+	+	+	+
Xunliang Li 2023	+	+	+	+	+
Yelena Petrosyan 2022	+	+	+	+	+
C. Qu2020	+	+	+	x	x
Lei Yuan 2024	+	+	+	x	x
Yefeng Tong 2023	+	+	+	+	+
Qian Li 2023	+	+	+	+	+
Emma Schwager 2024	+	+	+	+	+
Guiyu Lei2020	+	x	+	x	x
Zhi Xiang Du2022	+	+	+	x	x
Rui Fan 2023	+	+	+	+	+
Caitlyn Chiofolo2019	+	x	+	x	x
Charat Thongprayoon 2022	+	+	+	+	+
Yeonhee Lee 2021	+	+	−	+	−
Xian Zeng 2022	+	+	+	+	+
Yi Yang2022	+	+	+	x	x
Wen Tao Liu 2022	+	+	+	+	+
Fangfang Zhou 2023	+	+	+	+	+
Tingting Fan 2023	+	+	+	+	+
Mengqing Ma 2023	+	+	+	+	+
Amir Kamel Rahimi 2023	+	+	−	+	−
Xiaohong Zhang 2022	+	+	+	+	+
Chun−Te Huang 2023	+	+	+	+	+
Jahan C. Penny−Dimri 2020	+	+	+	x	x
Yan Luo 2023	+	+	+	x	x
Takaya Okawa 2022	+	+	+	x	x
Khaled Shawwa 2020	+	+	+	+	+
Hsin−Hsiung Chang 2022	+	+	+	+	+
Dabei Cai 2022	+	+	+	+	+
Siavash Zamirpour 2023	+	+	+	+	+
Ling Sun2020	+	+	+	x	x
Teddy Lazebnik 2022	+	+	+	x	x
Jialin Liu 2021	+	+	+	x	x
Yu Zhang2022	+	+	+	x	x
Huanhuan Shi 2023	+	+	+	x	x
Jun Wei 2024	+	+	+	+	+
Muying Wu 2023	x	+	+	x	x

D1: Participant
D2: Predictors
D3: Outcome
D4: Analysis

Judgement
+ Low
− Unclear
x High

Note: (A) Summary of risk of bias assessment. (B) Traffic light plot of risk of bias assessment.

Supplementary Figure S1. Risk-of-Bias Assessment Results Using the PROBAST+AI Tool for Included AKI Prediction Model Studies

n = 219

B (continued, page 3 of 5)

	D1	D2	D3	D4	Overall
Shanbing Hou2024	x	+	+	x	x
Hai−Xia Liu 2024	+	+	+	+	+
Zeng−Lei He 2020	x	+	+	x	x
Li Xinsai 2022	x	+	+	x	x
Yihan Zhang 2021	+	+	+	+	+
Le Li 2024	+	+	+	+	+
Jijun Yang 2023	+	+	+	+	+
Jiakang Shao 2023	+	+	+	x	x
Kun Zhu2018	+	+	+	x	x
Changho Han 2024	+	+	+	x	x
Shengqiang Chi 2023	+	x	+	+	+
Dongliang Yang2022	+	−	+	+	−
Anran Dai 2023	+	+	+	+	+
Xiao−Qin Luo 2022	+	+	+	+	+
Yen−Yu Chen 2023	x	+	+	x	x
Ling Pan 2023	+	−	+	+	−
Yu Tian 2021	+	+	+	+	+
Lingyu Xu 2024	+	+	+	+	+
Mee Yeon Lee 2024	+	+	+	+	+
Hyung−Chul Lee2018	+	+	+	+	+
JOSHUA PARRECO2019	+	x	+	x	x
Wenjuan Zhang 2024	+	−	+	x	x
Chien−Ning Hsu2020	+	+	+	+	+
Mengqing Ma 2024	+	+	+	+	+
Xiran Peng 2024	+	+	+	x	x
Xiang Yu 2022	+	+	+	x	x
Justin Y Lu 2022	+	+	+	+	+
Lei Lei2020	+	+	+	x	x
Lauren A Scanlon 2021	+	+	+	+	+
Liu Li 2023	+	x	+	x	x
Jia Tian 2023	+	x	+	+	+
Hsin−Hung Liu 2023	+	+	+	+	+
Xiaochi Lu 2024	+	+	+	+	+
Chi Cheng 2024	+	−	+	x	x
Meicheng Yang 2024	+	+	+	+	+
Donghwan Yun 2021	x	+	+	+	+
Yunlu Zhang 2021	+	+	+	+	+
Jiaxin Li2024	+	+	+	+	+
Kipyo Kim 2021	+	+	+	+	+
Menglei Gu 2024	+	+	+	+	+
XinPei Liu 2024	+	+	+	+	+
Xuejian Hou 2024	+	+	+	+	+
Yi Du2020	+	+	+	x	x
L. Nelson Sanchez−Pinto2016	+	+	+	+	+
Sang−Wook Lee 2024	+	+	+	+	+
Pierre Tran 2025	+	+	+	x	x
Li Zhang 2025	+	+	+	+	+
Nakyung Jeon 2019	+	+	+	+	+
Qi Wang2019	+	+	+	+	+
Xiaofei Zhang 2025	+	+	x	+	+

D1: Participant
D2: Predictors
D3: Outcome
D4: Analysis

Judgement
+ Low
− Unclear
x High

Note: (A) Summary of risk of bias assessment. (B) Traffic light plot of risk of bias assessment.

Supplementary Figure S1. Risk-of-Bias Assessment Results Using the PROBAST+AI Tool for Included AKI Prediction Model Studies

n = 219

B (continued, page 4 of 5)

	D1	D2	D3	D4	Overall
Y Zhang 2025	+	+	+	+	+
Ming Ye 2025	+	+	+	+	+
Chen Lin 2024	+	+	+	+	+
Kaori Ambe 2025	+	+	+	x	x
Kate R. J2016	+	+	+	+	+
Danfeng Zha 2022	+	x	+	x	x
Ke Lin2019	x	+	+	+	x
Yang Zhou 2023	+	+	+	+	+
Huan Jing2022	+	−	+	x	x
Yuxi Li 2022	+	+	+	+	+
Hayato Akimoto 2023	+	+	+	+	+
Benjamin R Griffin 2024	+	+	+	+	+
Jianqin He2019	+	+	+	+	+
Yang Li 2021	+	x	+	x	x
Jiaqi Lu2023	+	+	+	+	+
Yang Li2020	+	+	+	+	+
Luyan Zheng 2023	+	+	+	+	+
Lijuan Wu 2023	+	+	+	+	+
Catalina Martin−Cleary 2019	+	+	+	+	+
Huilan Tu 2024	+	+	+	+	+
Buqing Ma2024	+	+	+	+	+
Mei Wang 2022	+	x	+	+	x
Qing Qian 2021	+	+	+	+	+
Haofan Huang 2021	+	+	+	+	+
Amir Hossein Behnoush 2024	+	+	+	+	+
Daishan Jiang2019	+	x	+	x	x
Lasith Adhikari2019	+	+	+	x	x
Minoru Sakuragi 2024	+	+	+	x	x
Yang Li2020	+	+	+	+	+
Wenpeng Gao 2022	+	+	+	+	+
Kang Liu 2025	+	+	+	+	+
Simin Wu 2023	+	+	+	+	+
Sheng Shi2023	+	+	+	x	x
Daniela Ponce 2021	+	+	+	+	+
Tao Sun 2024	+	+	+	+	+
Okke Nikkinen 2022	x	+	+	x	x
Chenxi Huang2018	+	+	+	+	+
Jiaqi Wang 2022	+	+	+	x	x
Kevin Y Heo 2024	+	+	+	x	x
Xinyi Cao 2023	+	+	+	+	+
Luyan Zheng 2025	+	+	+	+	+
Junbo Shen 2024	+	+	+	x	x
Pan Pan 2023	+	+	+	+	+
Li Wang2017	+	+	+	+	+
Wenxing Peng 2023	+	+	+	+	+
Qi Xin 2022	+	+	+	+	+
Yuhei Goriki 2021	+	+	+	x	x
Hanlim Choi 2023	+	+	+	x	x
Dawei Chen 2023	+	+	+	x	x
Ruoran Wang 2024	+	+	+	x	x

D1: Participant
D2: Predictors
D3: Outcome
D4: Analysis

Judgement
+ Low
− Unclear
x High

Note: (A) Summary of risk of bias assessment. (B) Traffic light plot of risk of bias assessment.

Supplementary Figure S1. Risk-of-Bias Assessment Results Using the PROBAST+AI Tool for Included AKI Prediction Model Studies

n = 219

B (continued, page 5 of 5)

	D1	D2	D3	D4	Overall
Toshiki Kuno 2020	+	+	+	x	x
Xiang Li2024	+	+	+	+	+
Ziming Jiang 2023	+	+	+	+	+
Ira S Hofer 2022	+	+	+	x	x
Hang Zhang 2022	+	+	+	+	+
Suru Yue 2022	+	+	+	+	+
Xiao Yue2024	+	−	+	x	x
Katarzyna Krzanowska2024	+	+	+	+	+
Ping Yan2023	+	+	+	+	+
Congjie Wang2022	+	+	+	x	x
Qi Xin 2022	+	+	+	x	x
Xun Wang2021	x	x	+	+	x
Jing−Cheng Peng2021	+	+	+	+	+
Ira S. Hofer2020	+	+	+	x	x
Kang Liu 2025	+	+	+	+	+
Caifeng Li 2022	+	+	+	+	+
Yufeng Jiang2024	+	+	+	+	+
Shengtao Li2024	+	+	+	+	+
Tao Sun 2025	+	+	+	+	+
Xiang Li2024	+	+	+	+	+
Junlong Hu 2024	+	+	+	+	+
Enci Cai2024	+	+	+	+	+
Yixin Tian2023	+	+	+	+	+
Changwei Wu 2023	+	+	+	+	+
Lu−Huai Feng2023	x	x	+	+	x
Hui Zhao2022	+	+	+	x	x
Yue Hu2022	+	+	+	x	x
Yuhui Zhang2022	x	x	+	x	x
Kai Ma2022	+	+	+	x	x
Annunziata Nusca2021	x	+	x	x	x
Zhimeng Chen2021	+	+	+	x	x
Jiaming Wang2021	+	+	+	+	+
Jianchao Ma2021	+	+	+	x	x
Tingting Fan2021	+	+	+	x	x
Wang Xin 2021	+	+	+	x	x
Hao Du2021	+	+	+	+	+
Nina Rank2020	+	+	+	+	+
Yang Li2020	+	+	+	x	x
Yujun Deng2017	+	+	+	x	x
Shu Yi Ng2014	+	−	+	x	x
Won Ho Kim2013	+	+	+	x	x

D1: Participant
D2: Predictors
D3: Outcome
D4: Analysis

Judgement
+ Low
− Unclear
x High

Note: (A) Summary of risk of bias assessment. (B) Traffic light plot of risk of bias assessment.

Supplementary Figure S1A. Risk-of-Bias Assessment Results Using the PROBAST+AI Tool for Included AKI Prediction Model StudiesOutcome: Onset

n = 190

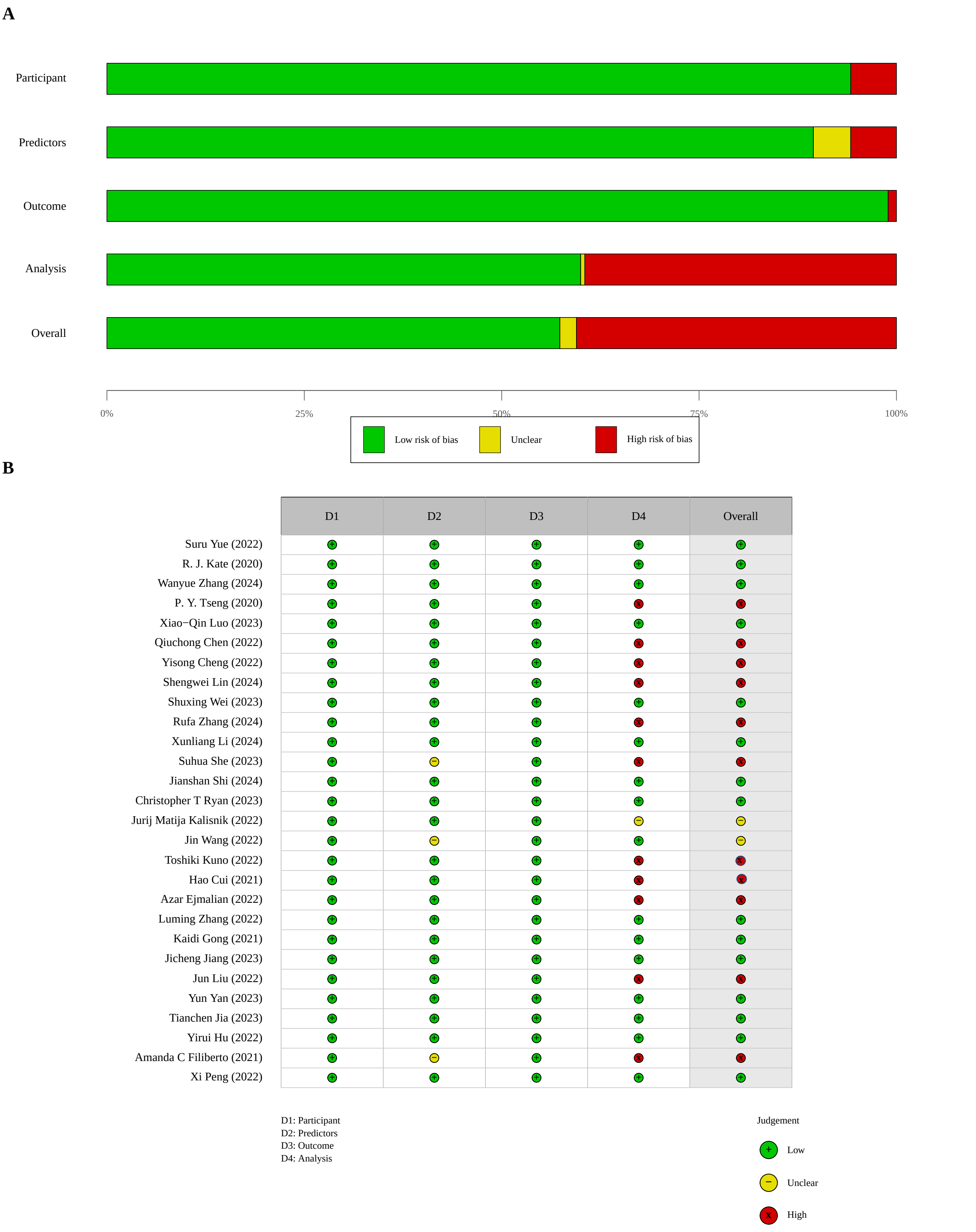

Note: (A) Summary of risk of bias assessment. (B) Traffic light plot of risk of bias assessment.

Supplementary Figure S1A. Risk-of-Bias Assessment Results Using the PROBAST+AI Tool for Included AKI Prediction Model StudiesOutcome: Onset

n = 190

B (continued, page 2 of 5)

	D1	D2	D3	D4	Overall
Yuchen Gao (2023)	+	+	+	+	+
Rao Sun (2024)	+	+	+	+	+
Rima S Zahr (2025)	+	+	+	x	x
Sunho Ko (2022)	+	+	+	+	+
Yuezi Song (2024)	+	+	+	x	x
Sasha Pierre−Paul (2022)	+	+	+	x	x
Yelena Petrosyan (2022)	+	+	+	+	+
C. Qu (2020)	+	+	+	x	x
Lei Yuan (2024)	+	+	+	x	x
Yefeng Tong (2023)	+	+	+	+	+
Qian Li (2023)	+	+	+	+	+
Emma Schwager (2024)	+	+	+	+	+
Guiyu Lei (2020)	+	x	+	x	x
Zhi Xiang Du (2022)	+	+	+	x	x
Rui Fan (2023)	+	+	+	+	+
Caitlyn Chiofolo (2019)	+	x	+	x	x
Charat Thongprayoon (2022)	+	+	+	+	+
Yeonhee Lee (2021)	+	+	+	+	+
Xian Zeng (2022)	+	+	+	+	+
Yi Yang (2022)	+	+	+	x	x
Wen Tao Liu (2022)	+	+	+	+	+
Fangfang Zhou (2023)	+	+	+	+	+
Tingting Fan (2023)	+	+	+	+	+
Mengqing Ma (2023)	+	+	+	+	+
Amir Kamel Rahimi (2023)	+	+	+	+	+
Xiaohong Zhang (2022)	+	+	+	+	+
Chun−Te Huang (2023)	+	+	+	+	+
Jahan C. Penny−Dimri (2020)	+	+	+	x	x
Yan Luo (2023)	+	+	+	x	x
Takaya Okawa (2022)	+	+	+	x	x
Khaled Shawwa (2020)	+	+	+	+	+
Dabei Cai (2022)	+	+	+	+	+
Siavash Zamirpour (2023)	+	+	+	+	+
Ling Sun (2020)	+	+	+	x	x
Teddy Lazebnik (2022)	+	+	+	x	x
Yu Zhang (2022)	+	+	+	x	x
Huanhuan Shi (2023)	+	+	+	x	x
Jun Wei (2024)	+	+	+	+	+
Muying Wu (2023)	x	+	+	x	x
Shanbing Hou (2024)	x	+	+	x	x
Hai−Xia Liu (2024)	+	+	+	+	+
Zeng−Lei He (2020)	x	+	+	x	x
Li Xinsai (2022)	x	+	+	x	x
Yihan Zhang (2021)	+	+	+	+	+
Jiakang Shao (2023)	+	+	+	x	x
Kun Zhu (2018)	+	+	+	x	x
Changho Han (2024)	+	+	+	x	x
Shengqiang Chi (2023)	+	x	+	+	+
Dongliang Yang (2022)	+	−	+	+	−
Anran Dai (2023)	+	+	+	+	+

D1: Participant
D2: Predictors
D3: Outcome
D4: Analysis

Judgement

+ Low

− Unclear

x High

Note: (A) Summary of risk of bias assessment. (B) Traffic light plot of risk of bias assessment.

Supplementary Figure S1A. Risk-of-Bias Assessment Results Using the PROBAST+AI Tool for Included AKI Prediction Model StudiesOutcome: Onset

n = 190

B (continued, page 3 of 5)

	D1	D2	D3	D4	Overall
Yen−Yu Chen (2023)	x	+	+	x	x
Ling Pan (2023)	+	−	+	+	−
Yu Tian (2021)	+	+	+	+	+
Lingyu Xu (2024)	+	+	+	+	+
Mee Yeon Lee (2024)	+	+	+	+	+
Hyung−Chul Lee (2018)	+	+	+	+	+
JOSHUA PARRECO (2019)	+	x	+	x	x
Wenjuan Zhang (2024)	+	−	+	x	x
Chien−Ning Hsu (2020)	+	+	+	+	+
Mengqing Ma (2024)	+	+	+	+	+
Xiran Peng (2024)	+	+	+	x	x
Xiang Yu (2022)	+	+	+	x	x
Justin Y Lu (2022)	+	+	+	+	+
Lei Lei (2020)	+	+	+	x	x
Lauren A Scanlon (2021)	+	+	+	+	+
Liu Li (2023)	+	x	+	x	x
Jia Tian (2023)	+	x	+	+	+
Hsin−Hung Liu (2023)	+	+	+	+	+
Xiaochi Lu (2024)	+	+	+	+	+
Chi Cheng (2024)	+	−	+	x	x
Meicheng Yang (2024)	+	+	+	+	+
Donghwan Yun (2021)	x	+	+	+	+
Yunlu Zhang (2021)	+	+	+	+	+
Jiaxin Li (2024)	+	+	+	+	+
Kipyo Kim (2021)	+	+	+	+	+
XinPei Liu (2024)	+	+	+	+	+
Xuejian Hou (2024)	+	+	+	+	+
Yi Du (2020)	+	+	+	x	x
L. Nelson Sanchez−Pinto (2016)	+	+	+	+	+
Sang−Wook Lee (2024)	+	+	+	+	+
Pierre Tran (2025)	+	+	+	x	x
Li Zhang (2025)	+	+	+	+	+
Nakyung Jeon (2019)	+	+	+	+	+
Qi Wang (2019)	+	+	+	+	+
Xiaofei Zhang (2025)	+	+	x	+	+
Y Zhang (2025)	+	+	+	+	+
Ming Ye (2025)	+	+	+	+	+
Chen Lin (2024)	+	+	+	+	+
Kaori Ambe (2025)	+	+	+	x	x
Kate R. J (2016)	+	+	+	+	+
Yang Zhou (2023)	+	+	+	+	+
Huan Jing (2022)	+	−	+	x	x
Yuxi Li (2022)	+	+	+	+	+
Hayato Akimoto (2023)	+	+	+	+	+
Benjamin R Griffin (2024)	+	+	+	+	+
Jianqin He (2019)	+	+	+	+	+
Yang Li (2021)	+	x	+	x	x
Jiaqi Lu (2023)	+	+	+	+	+
Yang Li (2020)	+	+	+	+	+
Luyan Zheng (2023)	+	+	+	+	+

D1: Participant
D2: Predictors
D3: Outcome
D4: Analysis

Judgement
+ Low
− Unclear
x High

Note: (A) Summary of risk of bias assessment. (B) Traffic light plot of risk of bias assessment.

Supplementary Figure S1A. Risk-of-Bias Assessment Results Using the PROBAST+AI Tool for Included AKI Prediction Model StudiesOutcome: Onset

n = 190

B (continued, page 4 of 5)

	D1	D2	D3	D4	Overall
Lijuan Wu (2023)	+	+	+	+	+
Catalina Martin−Cleary (2019)	+	+	+	+	+
Huilan Tu (2024)	+	+	+	+	+
Buqing Ma (2024)	+	+	+	+	+
Qing Qian (2021)	+	+	+	+	+
Amir Hossein Behnoush (2024)	+	+	+	+	+
Daishan Jiang (2019)	+	x	+	x	x
Yang Li (2020)	+	+	+	+	+
Wenpeng Gao (2022)	+	+	+	+	+
Kang Liu (2025)	+	+	+	+	+
Simin Wu (2023)	+	+	+	+	+
Sheng Shi (2023)	+	+	+	x	x
Tao Sun (2024)	+	+	+	+	+
Okke Nikkinen (2022)	x	+	+	x	x
Chenxi Huang (2018)	+	+	+	+	+
Jiaqi Wang (2022)	+	+	+	x	x
Kevin Y Heo (2024)	+	+	+	x	x
Xinyi Cao (2023)	+	+	+	+	+
Junbo Shen (2024)	+	+	+	x	x
Pan Pan (2023)	+	+	+	+	+
Li Wang (2017)	+	+	+	+	+
Wenxing Peng (2023)	+	+	+	+	+
Qi Xin (2022)	+	+	+	+	+
Yuhei Goriki (2021)	+	+	+	x	x
Hanlim Choi (2023)	+	+	+	x	x
Dawei Chen (2023)	+	+	+	x	x
Ruoran Wang (2024)	+	+	+	x	x
Toshiki Kuno (2020)	+	+	+	x	x
Ziming Jiang (2023)	+	+	+	+	+
Ira S Hofer (2022)	+	+	+	x	x
Hang Zhang (2022)	+	+	+	+	+
Suru Yue (2022)	+	+	+	+	+
Katarzyna Krzanowska (2024)	+	+	+	+	+
Ping Yan (2023)	+	+	+	+	+
Congjie Wang (2022)	+	+	+	x	x
Qi Xin (2022)	+	+	+	x	x
Xun Wang (2021)	x	x	+	+	x
Ira S. Hofer (2020)	+	+	+	x	x
Kang Liu (2022)	+	+	+	+	+
Yufeng Jiang (2024)	+	+	+	+	+
Tao Sun (2025)	+	+	+	+	+
Xiang Li (2024)	+	+	+	+	+
Junlong Hu (2024)	+	+	+	+	+
Enci Cai (2024)	+	+	+	+	+
Yixin Tian (2023)	+	+	+	+	+
Lu−Huai Feng (2023)	x	x	+	+	x
Hui Zhao (2022)	+	+	+	x	x
Yue Hu (2022)	+	+	+	x	x
Yuhui Zhang (2022)	x	x	+	x	x
Kai Ma (2022)	+	+	+	x	x

D1: Participant
D2: Predictors
D3: Outcome
D4: Analysis

Judgement

+ Low

− Unclear

x High

Note: (A) Summary of risk of bias assessment. (B) Traffic light plot of risk of bias assessment.

Supplementary Figure S1A. Risk-of-Bias Assessment Results Using the PROBAST+AI Tool for Included AKI Prediction Model StudiesOutcome: Onset

n = 190

B (continued, page 5 of 5)

	D1	D2	D3	D4	Overall
Annunziata Nusca (2021)	x	+	x	x	x
Zhimeng Chen (2021)	+	+	+	x	x
Jiaming Wang (2021)	+	+	+	+	+
Jianchao Ma (2021)	+	+	+	x	x
Tingting Fan (2021)	+	+	+	x	x
Wang Xin (2021)	+	+	+	x	x
Hao Du (2021)	+	+	+	+	+
Nina Rank (2020)	+	+	+	+	+
Yang Li (2020)	+	+	+	x	x
Yujun Deng (2017)	+	+	+	x	x
Shu Yi Ng (2014)	+	−	+	x	x
Won Ho Kim (2013)	+	+	+	x	x

D1: Participant
D2: Predictors
D3: Outcome
D4: Analysis

Judgement
+ Low
− Unclear
x High

Note: (A) Summary of risk of bias assessment. (B) Traffic light plot of risk of bias assessment.

Supplementary Figure S1B. Risk-of-Bias Assessment Results Using the PROBAST+AI Tool for Included AKI Prediction Model StudiesOutcome: Death

n = 31

A

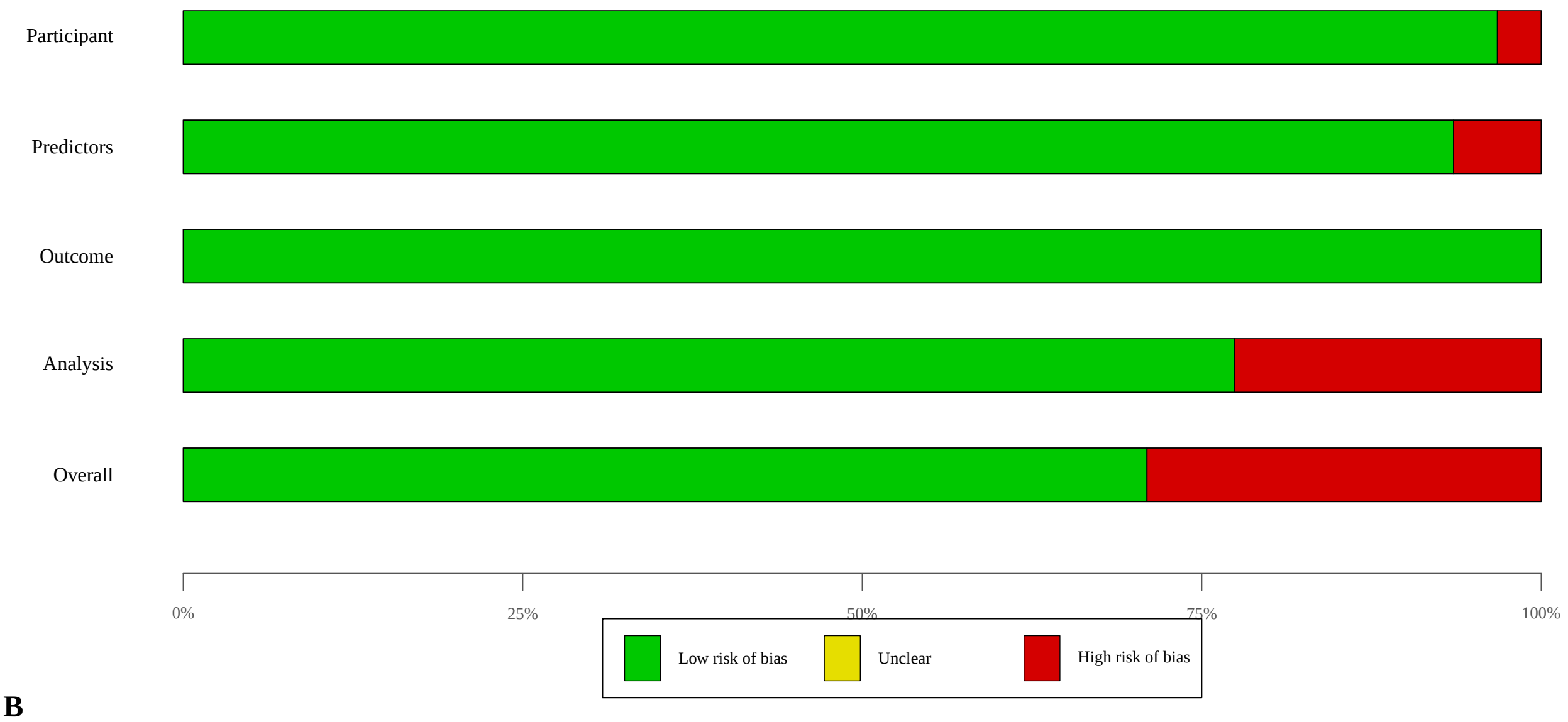

B

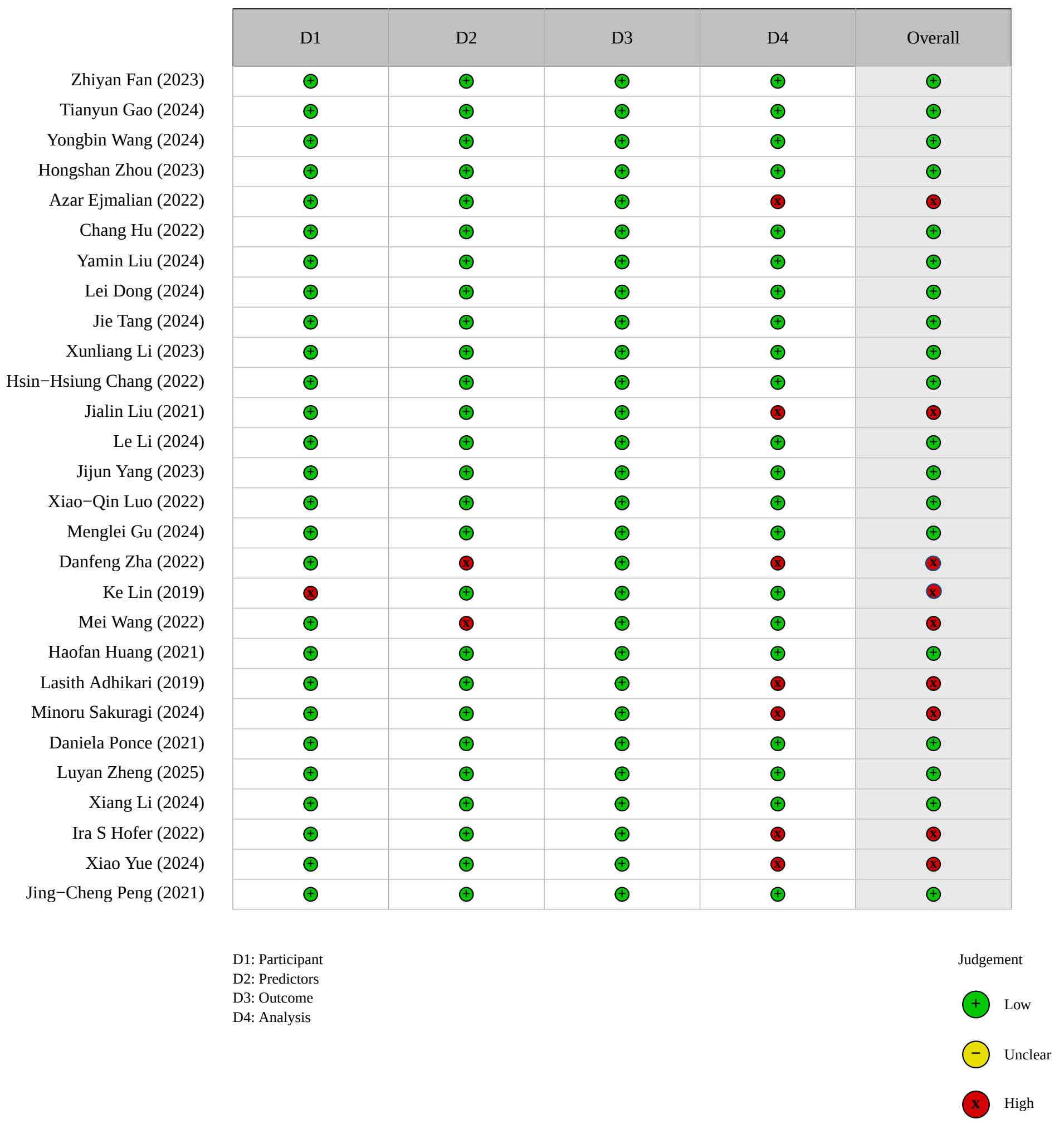

Note: (A) Summary of risk of bias assessment. (B) Traffic light plot of risk of bias assessment.

n = 31

Supplementary Figure S1B. Risk-of-Bias Assessment Results Using the PROBAST+AI Tool for Included AKI Prediction Model StudiesOutcome: Death

B (continued, page 2 of 2)

	D1	D2	D3	D4	Overall
Caifeng Li (2024)	+	+	+	+	+
Shengtao Li (2024)	+	+	+	+	+
Changwei Wu (2023)	+	+	+	+	+

D1: Participant
D2: Predictors
D3: Outcome
D4: Analysis

Judgement

+ Low
− Unclear
x High

Note: (A) Summary of risk of bias assessment. (B) Traffic light plot of risk of bias assessment.

Supplementary Figure S2. Forest Plot for Meta-analysis of AUCs of AKI Occurrence Prediction Models (Page 1 of 6)

Study ID	No. of models	Events/Total	AUC (95% CI)	Weight
Rima S Zahr (2025)	4	82/1178	0.940 (0.924−0.953)	0.6%
Pierre Tran (2025)	1	13/774	0.980 (0.761−0.999)	0.1%
Xiaofei Zhang (2025)	6	1289/2615	0.740 (0.595−0.847)	0.4%
Y Zhang (2025)	1	8721/161876	0.910 (0.905−0.915)	0.6%
Ming Ye (2025)	1	2112/54429	0.866 (0.852−0.879)	0.6%
Kaori Ambe (2025)	1	119/1253	0.820 (0.768−0.862)	0.5%
Kang Liu (2025)	2	51385/401062	0.859 (0.857−0.861)	0.6%
Tao Sun (2025)	1	152/581	0.844 (0.782−0.891)	0.5%
Wanyue Zhang (2024)	5	374/772	0.950 (0.931−0.964)	0.5%
Shengwei Lin (2024)	7	667/1235	0.867 (0.826−0.900)	0.5%
Rufa Zhang (2024)	4	97/437	0.830 (0.696−0.912)	0.4%
Xunliang Li (2024)	6	29551/53150	0.816 (0.808−0.824)	0.6%
Jianshan Shi (2024)	7	8575/10575	0.801 (0.792−0.810)	0.6%
Rao Sun (2024)	4	1359/88367	0.830 (0.806−0.851)	0.6%
Yuezi Song (2024)	8	73/701	0.835 (0.768−0.886)	0.5%
Lei Yuan (2024)	1	138/672	0.820 (0.558−0.943)	0.3%
Emma Schwager (2024)	1	29316/50342	0.756 (0.752−0.760)	0.6%
Jun Wei (2024)	12	1695/6891	0.808 (0.746−0.858)	0.5%
Shanbing Hou (2024)	1	197/347	0.713 (0.657−0.763)	0.5%
Hai−Xia Liu (2024)	5	530/1089	0.750 (0.674−0.813)	0.5%
Changho Han (2024)	1	322/2003	0.840 (0.810−0.866)	0.6%
Lingyu Xu (2024)	8	251/1685	0.813 (0.777−0.844)	0.6%
Mee Yeon Lee (2024)	1	1307/2272257	0.720 (0.691−0.747)	0.6%
Wenjuan Zhang (2024)	9	22/710	0.909 (0.806−0.960)	0.4%
Mengqing Ma (2024)	5	1006/6371	0.870 (0.855−0.884)	0.6%
Xiran Peng (2024)	2	346/10561	0.845 (0.793−0.886)	0.5%
Xiaochi Lu (2024)	9	1775/3920	0.955 (0.947−0.961)	0.6%
Chi, Cheng (2024)	1	79/258	0.856 (0.770−0.913)	0.5%
Meicheng Yang (2024)	4	10136/26813	0.763 (0.755−0.771)	0.6%
Jiaxin Li (2024)	1	325/1002	0.840 (0.790−0.880)	0.5%
XinPei Liu (2024)	6	261/527	0.771 (0.688−0.837)	0.5%
Xuejian Hou (2024)	5	417/1767	0.834 (0.807−0.858)	0.6%
Sang−Wook Lee (2024)	4	2392/81544	0.769 (0.769−0.770)	0.6%
Chen Lin (2024)	1	139/391	0.870 (0.765−0.932)	0.4%
Benjamin R Griffin (2024)	1	NA/18180	0.820 (0.810−0.830)	0.6%
**Overall**			**0.834 (0.821−0.846)**	

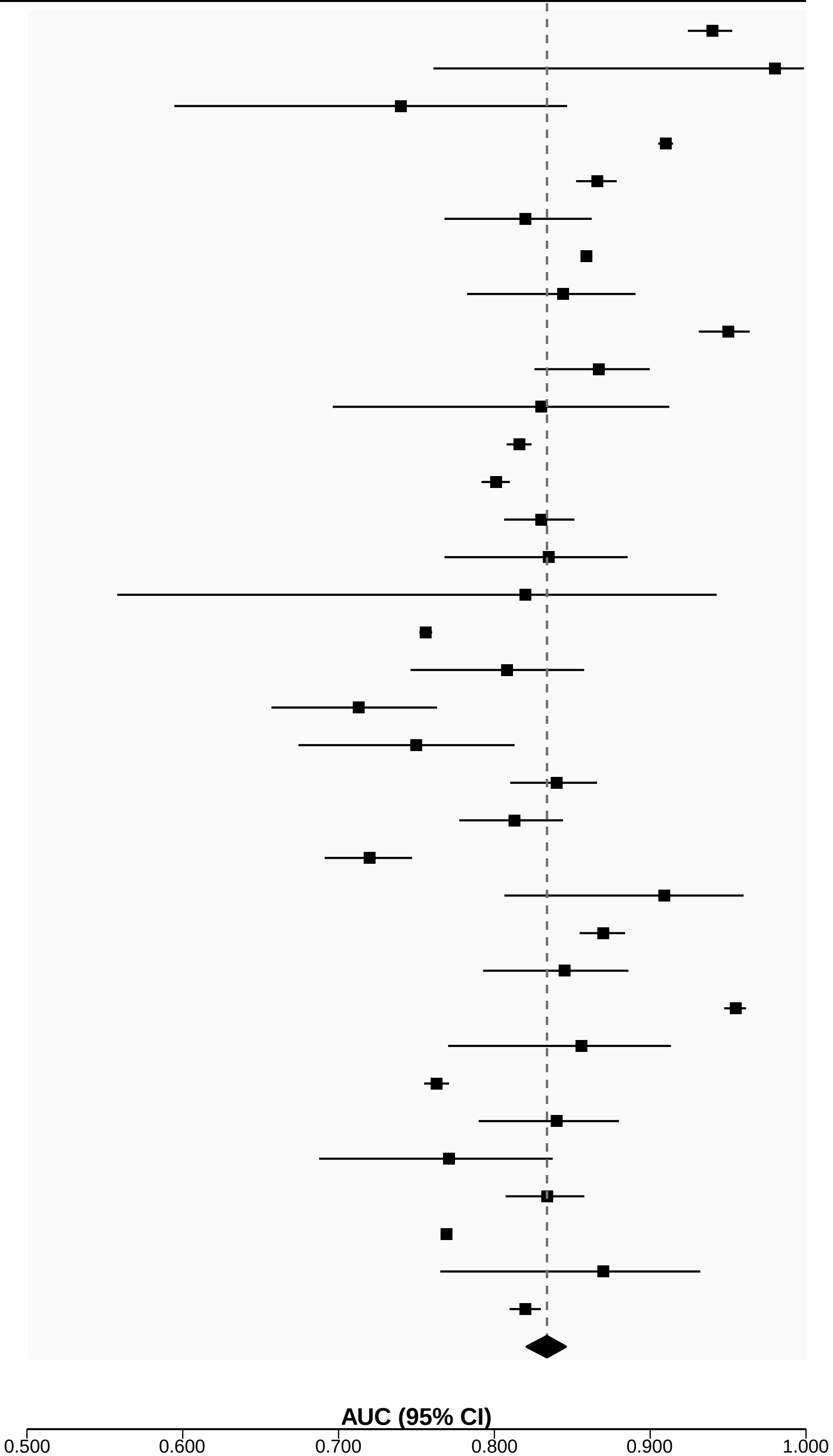

HKSJ-adjusted random-effects model;
Pooled AUC = 0.834 (95% CI 0.821−0.846)
Heterogeneity: Q = 3444324.607, df = 187, P < 0.001; I² = 99.993%; t² = 0.3672

Supplementary Figure S2. Forest Plot for Meta-analysis of AUCs of AKI Occurrence Prediction Models (Page 2 of 6)

Study ID	No. of models	Events/Total	AUC (95% CI)	Weight
Huilan Tu (2024)	1	745/1254	0.750 (0.723−0.775)	0.6%
Buqing Ma (2024)	1	1342/2089	0.848 (0.815−0.876)	0.6%
Amir Hossein Behnoush (2024)	5	646/4592	0.775 (0.728−0.816)	0.5%
Tao Sun (2024)	6	155/588	0.902 (0.863−0.931)	0.5%
Kevin Y Heo (2024)	5	4192/141697	0.750 (0.741−0.759)	0.6%
Junbo Shen (2024)	13	2091/6502	0.864 (0.863−0.864)	0.6%
Ruoran Wang (2024)	2	39/376	0.892 (0.816−0.939)	0.5%
Katarzyna Krzanowska (2024)	1	NA/4691	0.798 (0.791−0.805)	0.6%
Yufeng Jiang (2024)	1	190/1016	0.782 (0.696−0.849)	0.5%
Xiang Li (2024)	1	264/886	0.811 (0.778−0.840)	0.6%
Junlong Hu (2024)	11	449/1823	0.910 (0.889−0.927)	0.6%
Enci Cai (2024)	3	639/8636	0.859 (0.828−0.885)	0.6%
Xiao−Qin Luo (2023)	7	615/3863	0.889 (0.846−0.921)	0.5%
Shuxing Wei (2023)	12	416/1581	0.854 (0.828−0.877)	0.6%
Suhua She (2023)	6	356/1213	0.698 (0.634−0.755)	0.5%
Christopher T Ryan (2023)	1	2636/4267	0.870 (0.859−0.880)	0.6%
Jicheng Jiang (2023)	6	1020/2310	0.860 (0.806−0.901)	0.5%
Yun Yan (2023)	2	1713/3392	0.690 (0.654−0.724)	0.6%
Tianchen Jia (2023)	4	563/4831	0.882 (0.862−0.899)	0.6%
Yuchen Gao (2023)	2	4845/15880	0.849 (0.837−0.861)	0.6%
Yefeng Tong (2023)	6	380/1264	0.781 (0.650−0.872)	0.4%
Qian Li (2023)	12	1819/6495	0.745 (0.730−0.759)	0.6%
Rui Fan (2023)	3	210/778	0.856 (0.806−0.895)	0.5%
Fangfang Zhou (2023)	5	120/2230	0.774 (0.720−0.820)	0.5%
Tingting Fan (2023)	8	497/1322	0.800 (0.797−0.803)	0.6%
Mengqing Ma (2023)	6	634/3514	0.816 (0.766−0.858)	0.5%
Amir Kamel Rahimi (2023)	4	5473/31586	0.760 (0.752−0.768)	0.6%
Chun−Te Huang (2023)	5	18213/55156	0.865 (0.861−0.869)	0.6%
Yan Luo (2023)	7	4902/15218	0.849 (0.842−0.856)	0.6%
Siavash Zamirpour (2023)	3	601/10623	0.810 (0.766−0.847)	0.5%
Huanhuan Shi (2023)	3	2305/12511	0.840 (0.829−0.850)	0.6%
Muying Wu (2023)	5	121/582	0.845 (0.763−0.902)	0.5%
Jiakang Shao (2023)	6	637/2108	0.820 (0.798−0.840)	0.6%
Shengqiang Chi (2023)	7	777/1415	0.791 (0.767−0.813)	0.6%
Anran Dai (2023)	4	191/265	0.800 (0.639−0.901)	0.4%
			**0.834 (0.821−0.846)**	

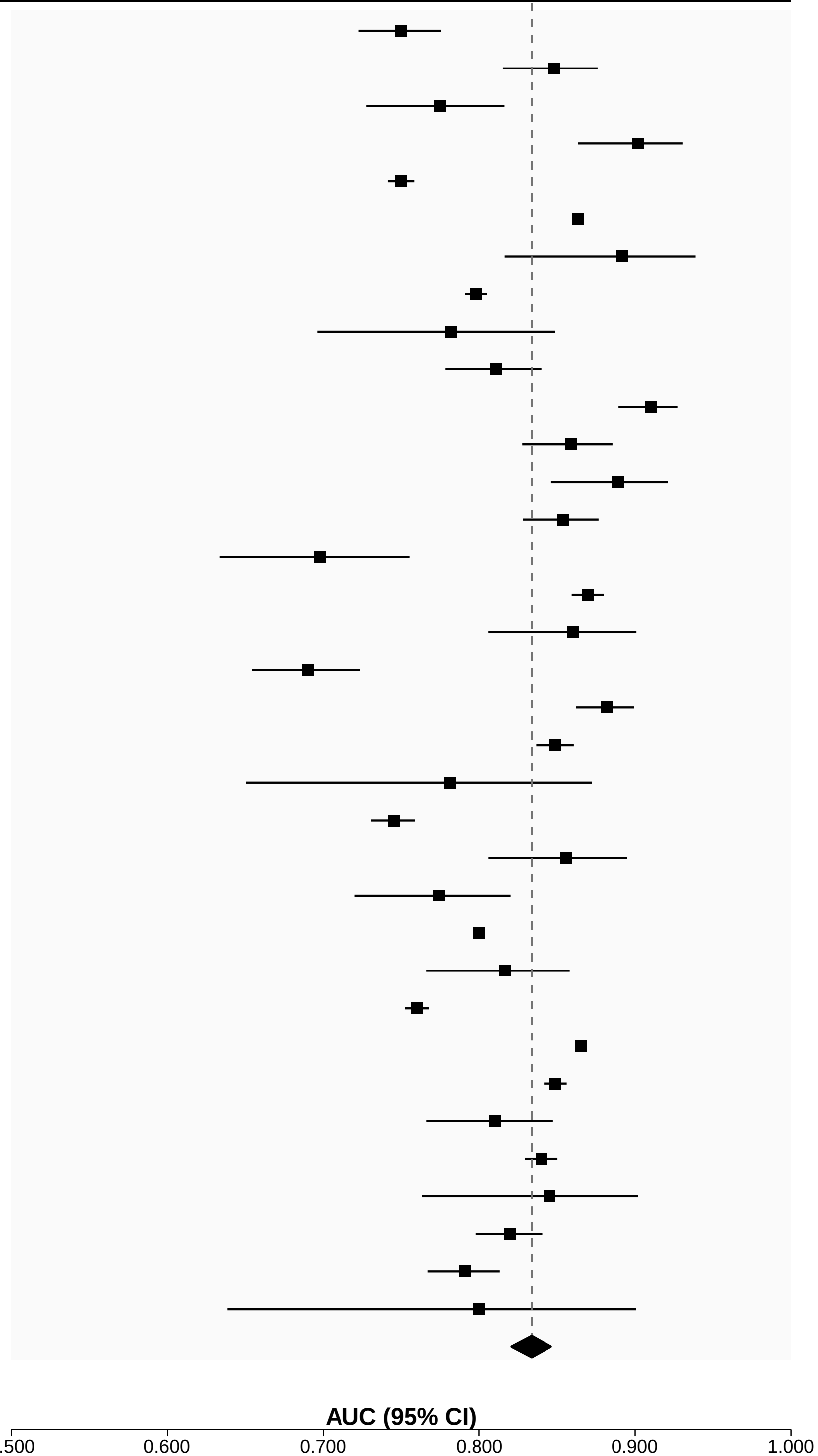

Overall
HKSJ-adjusted random-effects model;
Pooled AUC = 0.834 (95% CI 0.821−0.846)
Heterogeneity: Q = 3444324.607, df = 187, P < .001; I^2 = 100.0%; t^2 = 0.3672

Supplementary Figure S2. Forest Plot for Meta-analysis of AUCs of AKI Occurrence Prediction Models (Page 3 of 6)

Study ID	No. of models	Events/Total	AUC (95% CI)	Weight
Yen−Yu Chen (2023)	2	1264/18895	0.769 (0.753−0.784)	0.6%
Ling Pan (2023)	1	429/1159	0.760 (0.702−0.810)	0.5%
Liu Li (2023)	1	30/389	0.776 (0.661−0.860)	0.5%
Jia Tian (2023)	5	429/950	0.833 (0.805−0.858)	0.6%
Hsin−Hung Liu (2023)	3	550/12702	0.857 (0.836−0.876)	0.6%
Yang Zhou (2023)	4	375/1085	0.859 (0.831−0.883)	0.6%
Hayato Akimoto (2023)	6	5319/65667	0.827 (0.814−0.840)	0.6%
Jiaqi Lu (2023)	1	178/446	0.795 (0.703−0.864)	0.5%
Luyan Zheng (2023)	8	658/1938	0.712 (0.677−0.745)	0.6%
Lijuan Wu (2023)	1	29334/48780	0.840 (0.830−0.850)	0.6%
Simin Wu (2023)	1	499/799	0.772 (0.739−0.802)	0.6%
Sheng Shi (2023)	1	203/1137	0.755 (0.688−0.812)	0.5%
Xinyi Cao (2023)	1	1953/3278	0.724 (0.707−0.741)	0.6%
Pan Pan (2023)	4	1130/2260	0.892 (0.433−0.989)	0.1%
Wenxing Peng (2023)	1	365/2155	0.718 (0.662−0.768)	0.5%
Hanlim Choi (2023)	2	78/400	0.912 (0.856−0.947)	0.5%
Dawei Chen (2023)	1	827/2883	0.877 (0.835−0.909)	0.5%
Ziming Jiang (2023)	1	695/963	0.760 (0.694−0.816)	0.5%
Ping Yan (2023)	2	440/4218	0.940 (0.916−0.958)	0.5%
Yixin Tian (2023)	1	1325/9649	0.821 (0.754−0.873)	0.5%
Lu−Huai Feng (2023)	1	3119/4375	0.774 (0.752−0.794)	0.6%
Suru Yue (2022)	7	2397/3176	0.817 (0.802−0.831)	0.6%
Qiuchong Chen (2022)	9	99/1000	0.821 (0.815−0.827)	0.6%
Yisong Cheng (2022)	4	151/488	0.828 (0.780−0.867)	0.5%
Jurij Matija Kalisnik (2022)	2	1699/7507	0.880 (0.870−0.889)	0.6%
Jin Wang (2022)	1	95/211	0.780 (0.708−0.838)	0.5%
Toshiki Kuno (2022)	2	1800/19222	0.772 (0.759−0.785)	0.6%
Azar Ejmalian (2022)	7	672/1435	0.810 (0.807−0.813)	0.6%
Luming Zhang (2022)	5	22099/45895	0.995 (0.995−0.995)	0.6%
Jun Liu (2022)	10	402/1596	0.930 (0.910−0.946)	0.5%
Yirui Hu (2022)	2	26410/209330	0.870 (0.860−0.880)	0.6%
Xi Peng (2022)	13	4276/18329	0.816 (0.808−0.824)	0.6%
Sunho Ko (2022)	1	553/5757	0.890 (0.871−0.907)	0.6%
Sasha Pierre−Paul (2022)	1	63/200	0.781 (0.698−0.846)	0.5%
Yelena Petrosyan (2022)	3	1760/6522	0.750 (0.735−0.764)	0.6%
**Overall**			**0.834 (0.821−0.846)**	

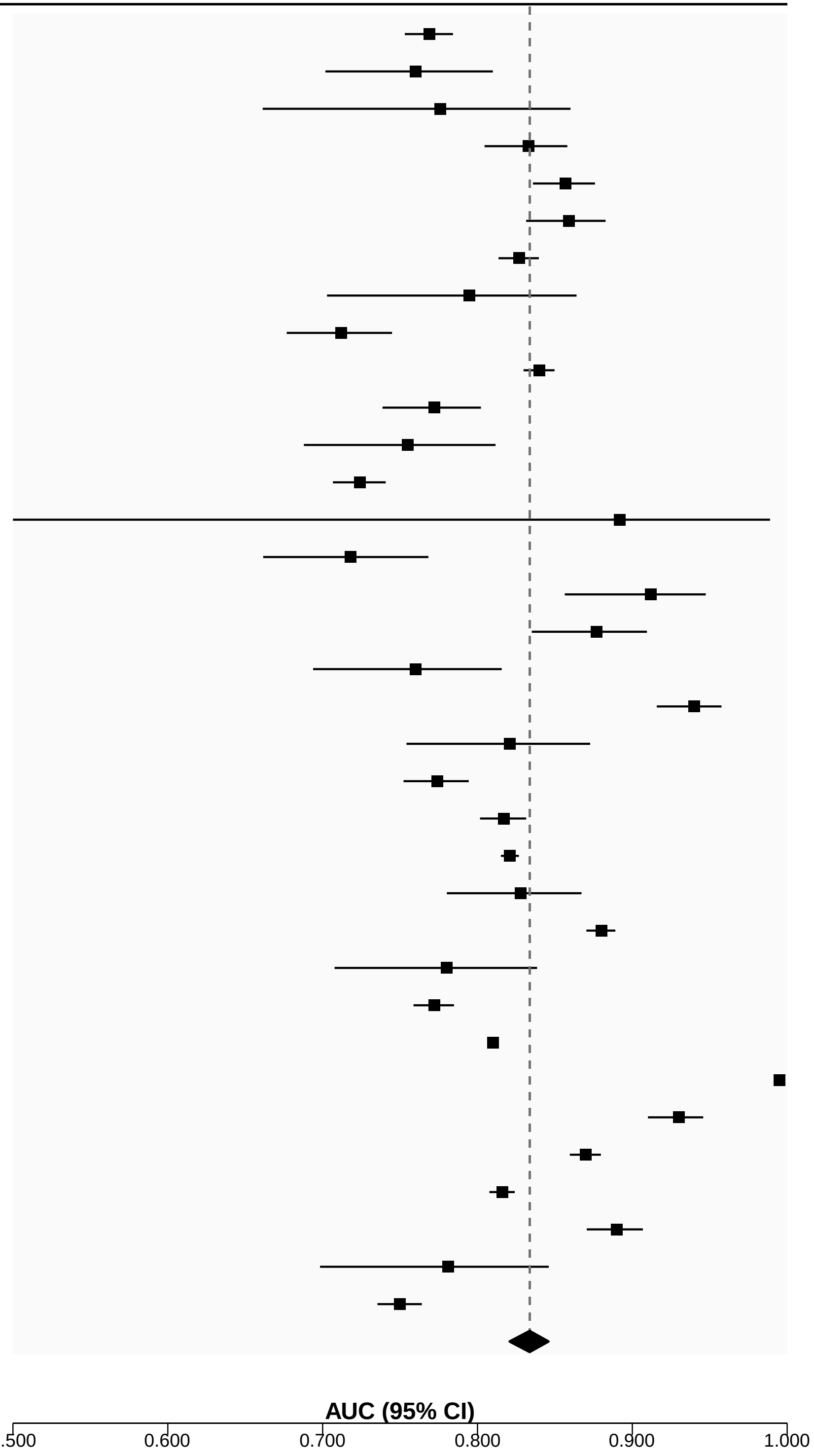

HKSJ-adjusted random-effects model;
Pooled AUC = 0.834 (95% CI 0.821−0.846)
Heterogeneity: Q = 3444324.607, df = 187, P < .001; I^2 = 100.0%; t^2 = 0.3672

Supplementary Figure S2. Forest Plot for Meta-analysis of AUCs of AKI Occurrence Prediction Models (Page 4 of 6)

Study ID	No. of models	Events/Total	AUC (95% CI)	Weight
Zhi Xiang Du (2022)	1	67/315	0.967 (0.937−0.983)	0.4%
Charat Thongprayoon (2022)	6	4745/13158	0.790 (0.769−0.809)	0.6%
Xian Zeng (2022)	7	331/3386	0.887 (0.861−0.909)	0.6%
Yi Yang (2022)	5	67/424	0.913 (0.852−0.950)	0.5%
Wen Tao Liu (2022)	5	919/2678	0.960 (0.950−0.968)	0.6%
Xiaohong Zhang (2022)	5	844/3434	0.790 (0.770−0.809)	0.6%
Takaya Okawa (2022)	1	213/1240	0.770 (0.729−0.807)	0.6%
Dabei Cai (2022)	6	1098/3882	0.781 (0.749−0.810)	0.6%
Teddy Lazebnik (2022)	1	231/723	0.750 (0.707−0.788)	0.6%
Yu Zhang (2022)	1	776/1731	0.706 (0.660−0.748)	0.6%
Li Xinsai (2022)	5	176/456	0.760 (0.603−0.868)	0.4%
Dongliang Yang (2022)	1	202/996	0.993 (0.978−0.998)	0.3%
Xiang Yu (2022)	9	68/248	0.832 (0.758−0.886)	0.5%
Justin Y Lu (2022)	1	2290/5868	0.965 (0.959−0.970)	0.6%
Huan Jing (2022)	1	288/1082	0.709 (0.671−0.745)	0.6%
Yuxi Li (2022)	7	189/2560	0.820 (0.780−0.854)	0.5%
Wenpeng Gao (2022)	5	17222/30020	0.923 (0.920−0.926)	0.6%
Okke Nikkinen (2022)	3	25/648	0.980 (0.978−0.981)	0.6%
Jiaqi Wang (2022)	1	223/3314	0.800 (0.762−0.833)	0.6%
Qi Xin (2022)	1	485/849	0.858 (0.832−0.881)	0.6%
Ira S Hofer (2022)	1	15989/101070	0.925 (0.924−0.925)	0.6%
Hang Zhang (2022)	3	353/1457	0.881 (0.818−0.924)	0.5%
Suru Yue (2022)	1	1566/2415	0.760 (0.741−0.778)	0.6%
Congjie Wang (2022)	1	85/480	0.810 (0.733−0.869)	0.5%
Qi Xin (2022)	1	523/1051	0.888 (0.843−0.921)	0.5%
Kang Liu (2022)	1	7259/76957	0.780 (0.770−0.790)	0.6%
Hui Zhao (2022)	1	NA/452	0.731 (0.606−0.828)	0.5%
Yue Hu (2022)	1	214/1073	0.773 (0.709−0.827)	0.5%
Yuhui Zhang (2022)	1	53/224	0.848 (0.779−0.898)	0.5%
Kai Ma (2022)	1	168/1156	0.767 (0.705−0.820)	0.5%
Hao Cui (2021)	3	122/214	0.972 (0.903−0.992)	0.3%
Kaidi Gong (2021)	8	1739/12447	0.781 (0.766−0.795)	0.6%
Amanda C Filiberto (2021)	1	599/1631	0.770 (0.727−0.808)	0.6%
Yeonhee Lee (2021)	5	1167/4104	0.810 (0.781−0.836)	0.6%
Yihan Zhang (2021)	5	528/975	0.750 (0.674−0.813)	0.5%
**Overall**			**0.834 (0.821−0.846)**	

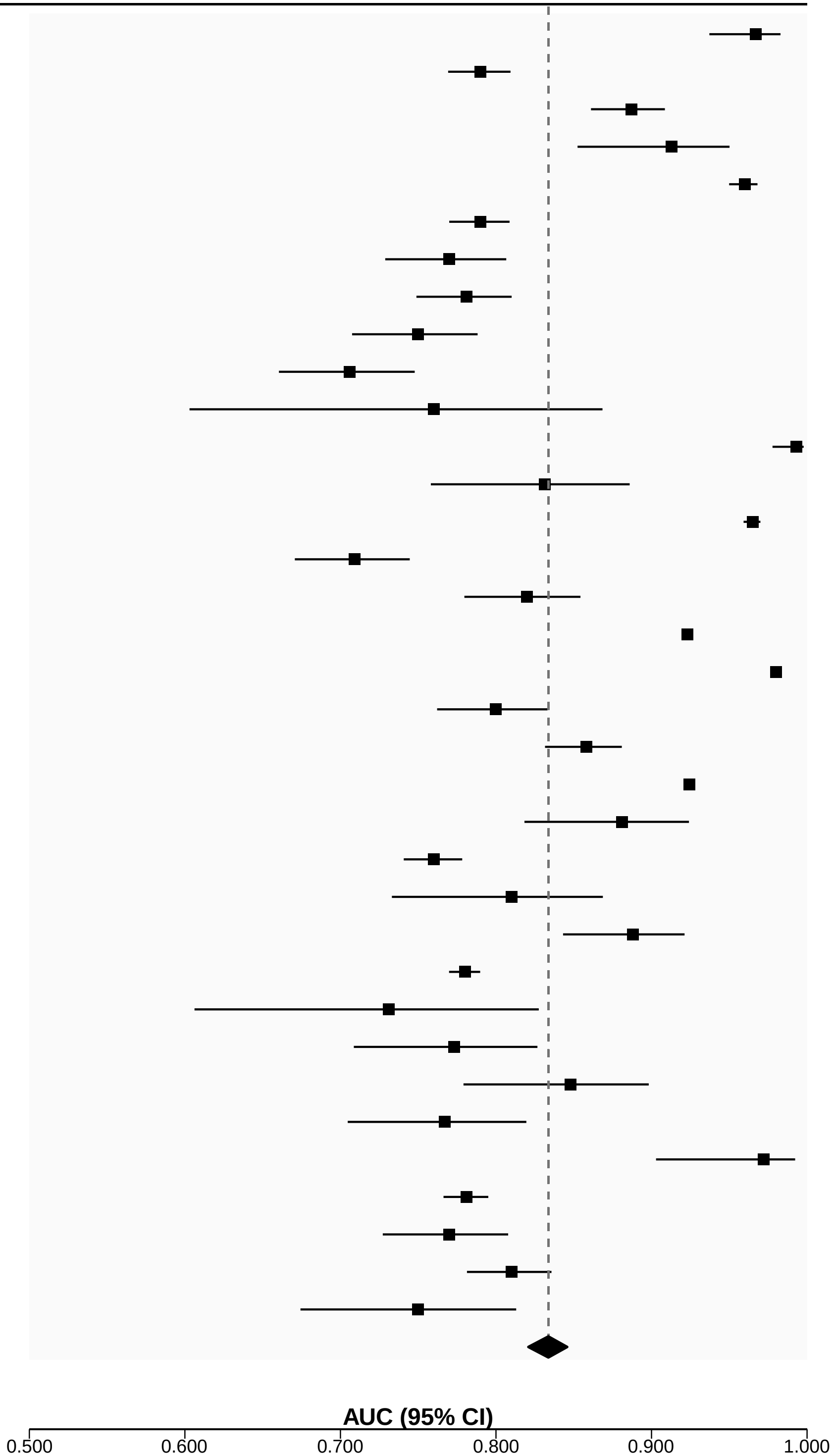

HKSJ-adjusted random-effects model;
Pooled AUC = 0.834 (95% CI 0.821−0.846)
Heterogeneity: Q = 3444324.607, df = 187, P < .001; I^2 =100.0%; τ^2 = 0.3672

Supplementary Figure S2. Forest Plot for Meta-analysis of AUCs of AKI Occurrence Prediction Models (Page 5 of 6)

Study ID	No. of models	Events/Total	AUC (95% CI)	Weight
Yu Tian, (2021)	1	20026/58399	0.690 (0.680−0.700)	0.6%
Lauren A Scanlon (2021)	1	74812/597403	0.654 (0.632−0.676)	0.6%
Donghwan Yun (2021)	8	261/14185	0.755 (0.705−0.799)	0.5%
Yunlu Zhang (2021)	2	NA/589	0.850 (0.756−0.912)	0.5%
Kipyo Kim (2021)	2	7163/149108	0.914 (0.909−0.918)	0.6%
Yang Li (2021)	1	275/1138	0.735 (0.645−0.809)	0.5%
Qing Qian (2021)	5	3929/17205	0.905 (0.898−0.911)	0.6%
Yuhei Goriki (2021)	1	77/908	0.754 (0.644−0.839)	0.5%
Xun Wang (2021)	1	731/1520	0.857 (0.797−0.902)	0.5%
Annunziata Nusca (2021)	1	48/1344	0.848 (0.781−0.897)	0.5%
Zhimeng Chen (2021)	6	13935/46385	0.858 (0.857−0.858)	0.6%
Jiaming Wang (2021)	3	2336/4672	0.926 (0.909−0.940)	0.6%
Jianchao Ma (2021)	1	121/358	0.784 (0.688−0.857)	0.5%
Tingting Fan (2021)	1	314/760	0.712 (0.636−0.778)	0.5%
Wang Xin (2021)	2	131/272	0.755 (0.696−0.806)	0.5%
R. J. Kate, (2020)	1	3786/44691	0.709 (0.690−0.728)	0.6%
P. Y. Tseng (2020)	6	163/671	0.843 (0.771−0.895)	0.5%
C. Qu (2020)	5	80/334	0.919 (0.865−0.953)	0.5%
Guiyu Lei (2020)	4	652/897	0.800 (0.767−0.829)	0.6%
Jahan C. Penny−Dimri (2020)	4	4599/96653	0.780 (0.755−0.803)	0.6%
Khaled Shawwa (2020)	1	47867/117001	0.656 (0.648−0.664)	0.6%
Ling Sun (2020)	3	226/1495	0.820 (0.758−0.869)	0.5%
Zeng−Lei He (2020)	5	176/493	0.850 (0.783−0.899)	0.5%
Chien−Ning Hsu (2020)	2	19448/234867	0.761 (0.757−0.765)	0.6%
Lei Lei (2020)	4	77/1173	0.772 (0.703−0.829)	0.5%
Yi Du (2020)	1	58/292	0.896 (0.826−0.940)	0.5%
Yang Li (2020)	1	1943/5533	0.816 (0.777−0.850)	0.6%
Yang Li (2020)	6	940/6495	0.790 (0.736−0.835)	0.5%
Toshiki Kuno (2020)	2	1499/14273	0.826 (0.809−0.841)	0.6%
Ira S. Hofer (2020)	2	4838/59981	0.792 (0.775−0.808)	0.6%
Nina Rank (2020)	1	1287/2574	0.893 (0.854−0.922)	0.5%
Yang Li (2020)	2	370/2395	0.835 (0.811−0.857)	0.6%
Caitlyn Chiofolo (2019)	1	1935/6530	0.882 (0.866−0.896)	0.6%
JOSHUA PARRECO (2019)	3	8408/151098	0.834 (0.829−0.839)	0.6%
Nakyung Jeon (2019)	1	1212/62561	0.790 (0.772−0.807)	0.6%
**Overall**			**0.834 (0.821−0.846)**	

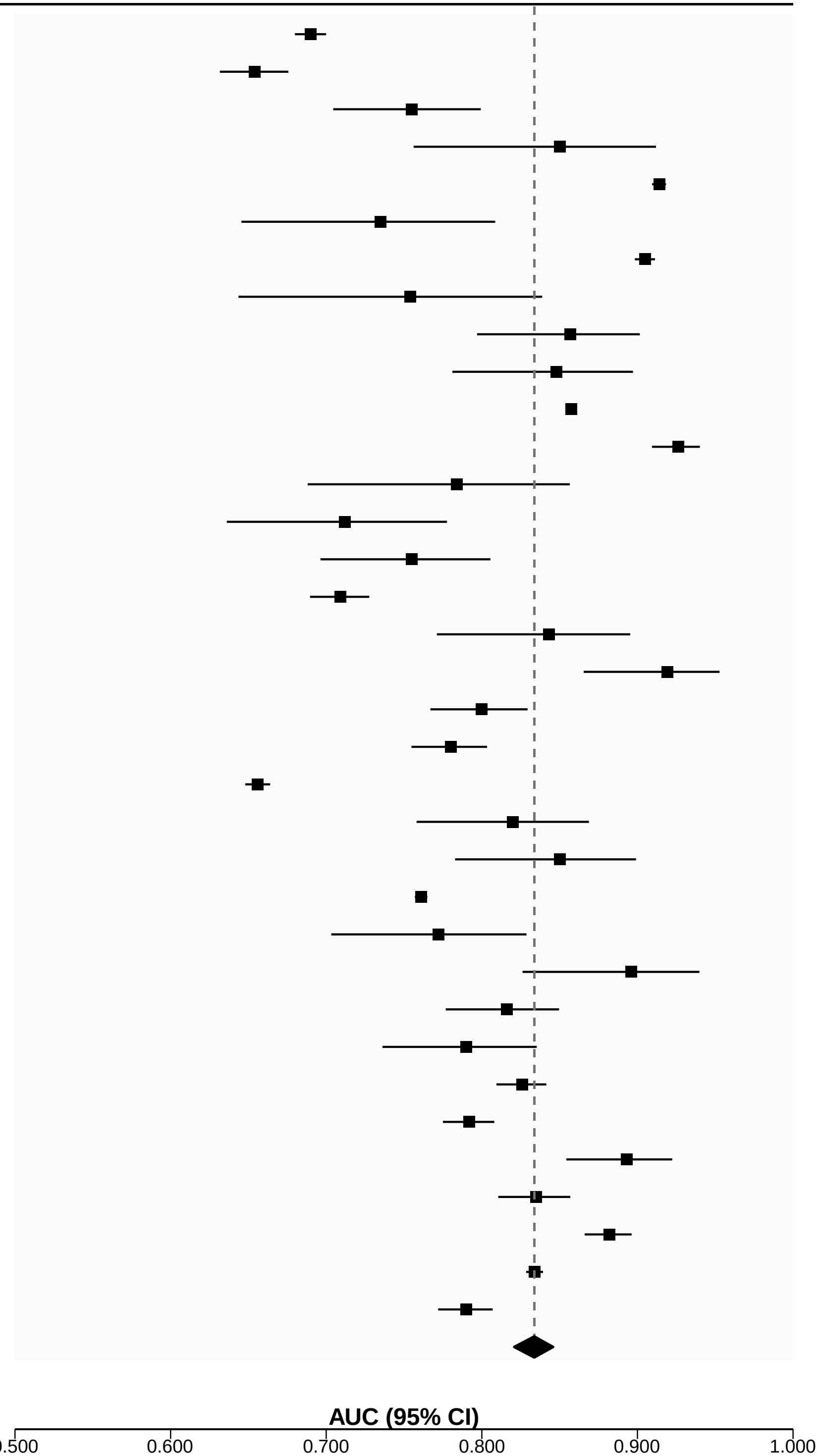

HKSJ-adjusted random-effects model;
Pooled AUC = 0.834 (95% CI 0.821−0.846)
Heterogeneity: Q = 3444324.607, df = 187, P < .001; I^2 = 100.0%; t^2 = 0.3672

Supplementary Figure S2. Forest Plot for Meta-analysis of AUCs of AKI Occurrence Prediction Models (Page 6 of 6)

Study ID	No. of models	Events/Total	AUC (95% CI)	Weight
Qi Wang (2019)	1	468/936	0.783 (0.724−0.832)	0.5%
Jianqin He, (2019)	5	1634/72846	0.734 (0.733−0.735)	0.6%
Catalina Martin−Cleary (2019)	3	2385/47466	0.818 (0.810−0.826)	0.6%
Daishan Jiang (2019)	1	125/442	0.755 (0.712−0.794)	0.6%
Kun Zhu (2018)	8	365/1211	0.900 (0.860−0.930)	0.5%
Hyung−Chul Lee (2018)	6	770/2010	0.780 (0.754−0.804)	0.6%
Chenxi Huang (2018)	2	72954/970869	0.759 (0.756−0.762)	0.6%
Li Wang (2017)	1	2452/6009	0.740 (0.709−0.769)	0.6%
Yujun Deng (2017)	1	326/1084	0.863 (0.821−0.896)	0.5%
L. Nelson Sanchez−Pinto (2016)	1	377/9396	0.860 (0.844−0.875)	0.6%
Kate, R. J (2016)	5	2258/25521	0.743 (0.731−0.754)	0.6%
Shu Yi Ng, (2014)	1	1642/28422	0.810 (0.797−0.823)	0.6%
Won Ho Kim (2013)	1	NA/737	0.740 (0.675−0.796)	0.5%
**Overall**			**0.834 (0.821−0.846)**	

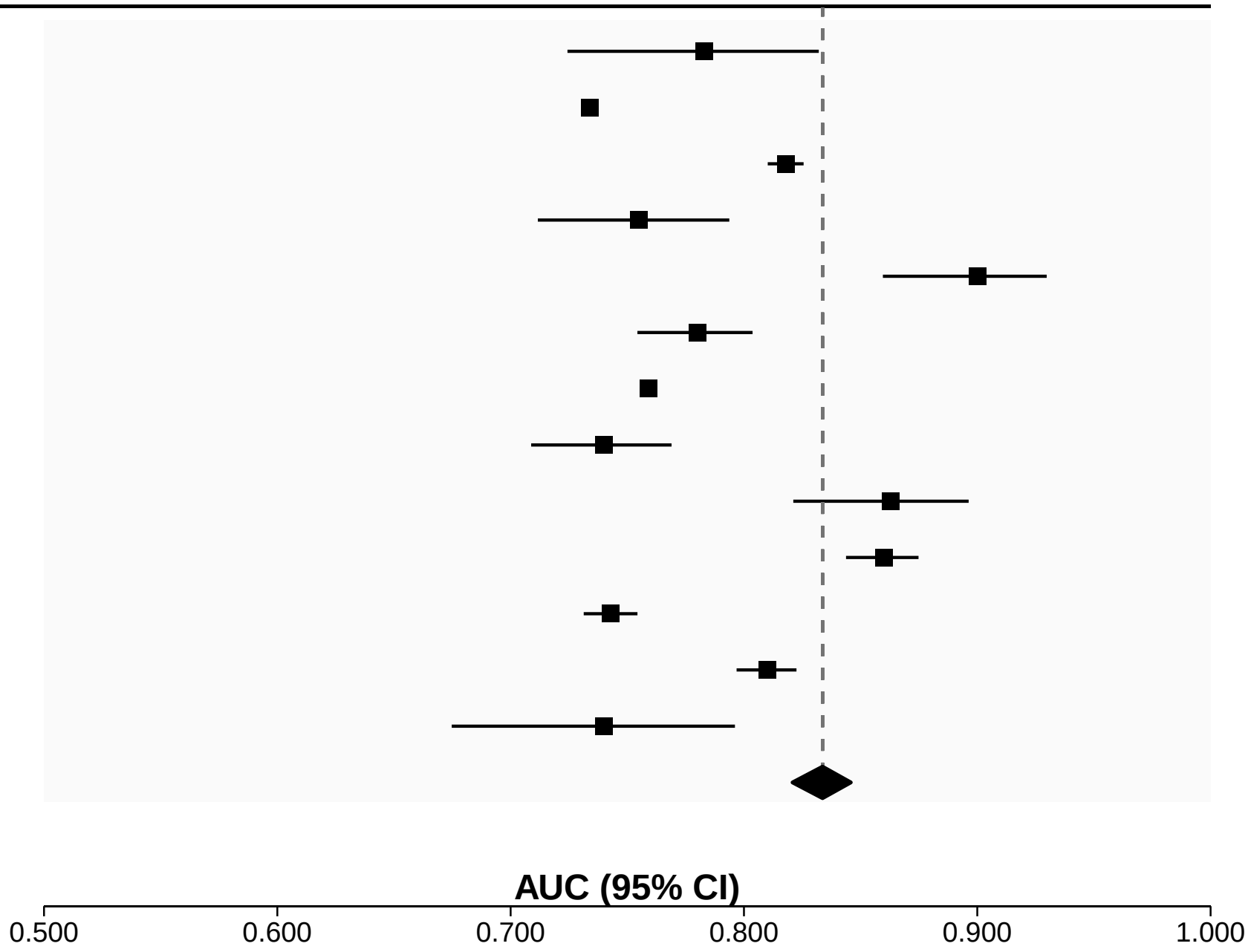

HKSJ-adjusted random-effects model;
Pooled AUC = 0.834 (95% CI 0.821−0.846)
Heterogeneity: Q = 3444324.607, df = 187, P < .001; I² = 100.0%; t² = 0.3672

Supplementary Figure S3. Forest Plot for Meta-analysis of AUCs of Mortality Following AKI Prediction Models(Page 1 of 1)

Study ID	No. of models	Events/Total	AUC (95% CI)	Weight
Luyan Zheng (2025)	2	274/1049	0.787 (0.741−0.827)	3.2%
Tianyun Gao (2024)	8	2352/12196	0.798 (0.773−0.821)	3.4%
Yongbin Wang (2024)	9	670/1395	0.756 (0.698−0.806)	3.1%
Yamin Liu (2024)	3	246/1265	0.724 (0.640−0.795)	2.9%
Lei Dong (2024)	5	1769/7544	0.796 (0.734−0.847)	3.0%
Jie Tang (2024)	11	1813/8426	0.804 (0.791−0.816)	3.5%
Le Li (2024)	6	3249/14408	0.784 (0.774−0.794)	3.5%
Menglei Gu (2024)	9	1080/1535	0.800 (0.777−0.821)	3.4%
Minoru Sakuragi (2024)	1	112/616	0.880 (0.831−0.916)	2.8%
Xiang Li (2024)	1	379/606	0.799 (0.762−0.831)	3.3%
Xiao Yue (2024)	1	2496/11334	0.810 (0.800−0.820)	3.5%
Caifeng Li (2024)	3	681/1663	0.720 (0.678−0.758)	3.3%
Shengtao Li (2024)	1	160/1079	0.828 (0.784−0.865)	3.1%
Zhiyan Fan (2023)	5	NA/2599	0.810 (0.789−0.829)	3.4%
Hongshan Zhou (2023)	11	3318/16154	0.754 (0.744−0.764)	3.5%
Xunliang Li (2023)	6	1629/8129	0.794 (0.759−0.825)	3.3%
Jijun Yang (2023)	4	1940/9158	0.873 (0.859−0.885)	3.4%
Changwei Wu (2023)	1	1864/137084	0.924 (0.916−0.932)	3.4%
Azar Ejmalian (2022)	7	413/1435	0.800 (0.797−0.803)	3.5%
Chang Hu (2022)	7	3484/22360	0.890 (0.881−0.898)	3.5%
Hsin−Hsiung Chang (2022)	4	1235/3412	0.823 (0.789−0.853)	3.3%
Xiao−Qin Luo (2022)	3	2979/15603	0.848 (0.838−0.858)	3.5%
Danfeng Zha (2022)	2	139/270	0.820 (0.702−0.898)	2.1%
Mei Wang (2022)	1	196/1836	0.867 (0.811−0.908)	2.8%
Ira S Hofer (2022)	1	2312/101070	0.943 (0.937−0.949)	3.4%
Jialin Liu (2021)	4	1234/7548	0.792 (0.776−0.807)	3.5%
Haofan Huang (2021)	1	1610/4558	0.820 (0.806−0.833)	3.5%
Daniela Ponce (2021)	3	544/870	0.831 (0.755−0.887)	2.6%
Jing−Cheng Peng (2021)	1	183/842	0.907 (0.851−0.943)	2.5%
Ke Lin (2019)	3	2586/19044	0.866 (0.862−0.870)	3.5%
Lasith Adhikari (2019)	1	1339/2911	0.870 (0.842−0.894)	3.3%
**Overall**			**0.830 (0.808−0.851)**	

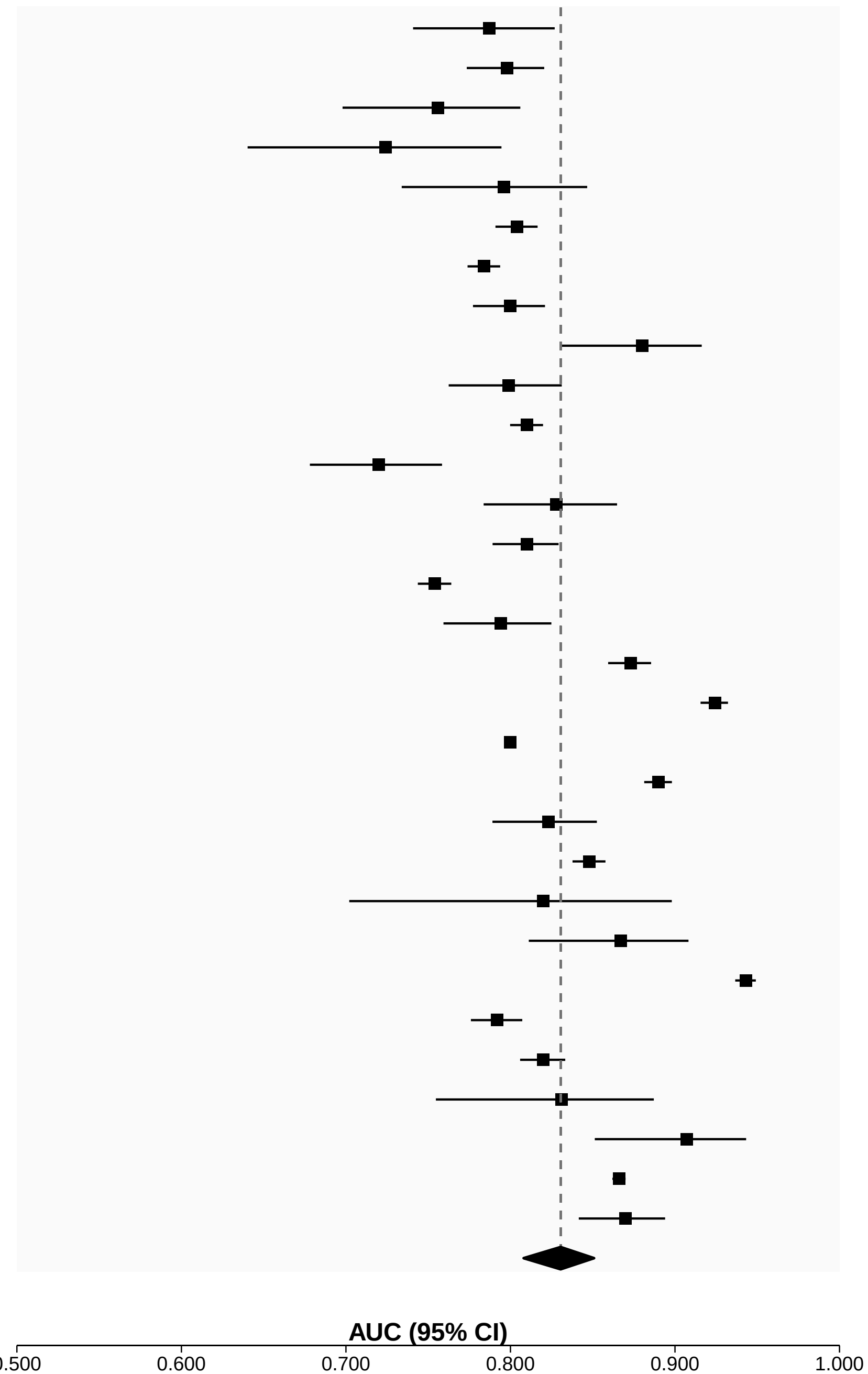

HKSJ-adjusted random-effects model;Pooled AUC = 0.830 (95% CI 0.807−0.851)

Heterogeneity: Q = 1976.606, df = 30, P < .001; I² = 99.0%; τ² = 0.1709

Supplementary Figure S4. Complementary Model-Level Subgroup Meta-analysis of AUCs for AKI Occurrence Prediction by AI Model Application Type

REML random-effects subgroup meta-analysis with HKSJ-adjusted inference; overall pooled AUC = 0.785 (0.777−0.793)

Model class	No.of AUC estimates (k)	AUC (95% CI)	I^2	tau^2
Deep learning models	48	0.783 (0.754−0.809)	100.0%	0.292
Hybrid models and others	82	0.767 (0.750−0.784)	100.0%	0.178
Linear/generalized linear models	153	0.782 (0.769−0.794)	100.0%	0.154
Tree-based and ensemble methods	121	0.800 (0.785−0.814)	100.0%	0.237
**Overall**	**404**	**0.785 (0.777−0.793)**	**100.0%**	**0.206**

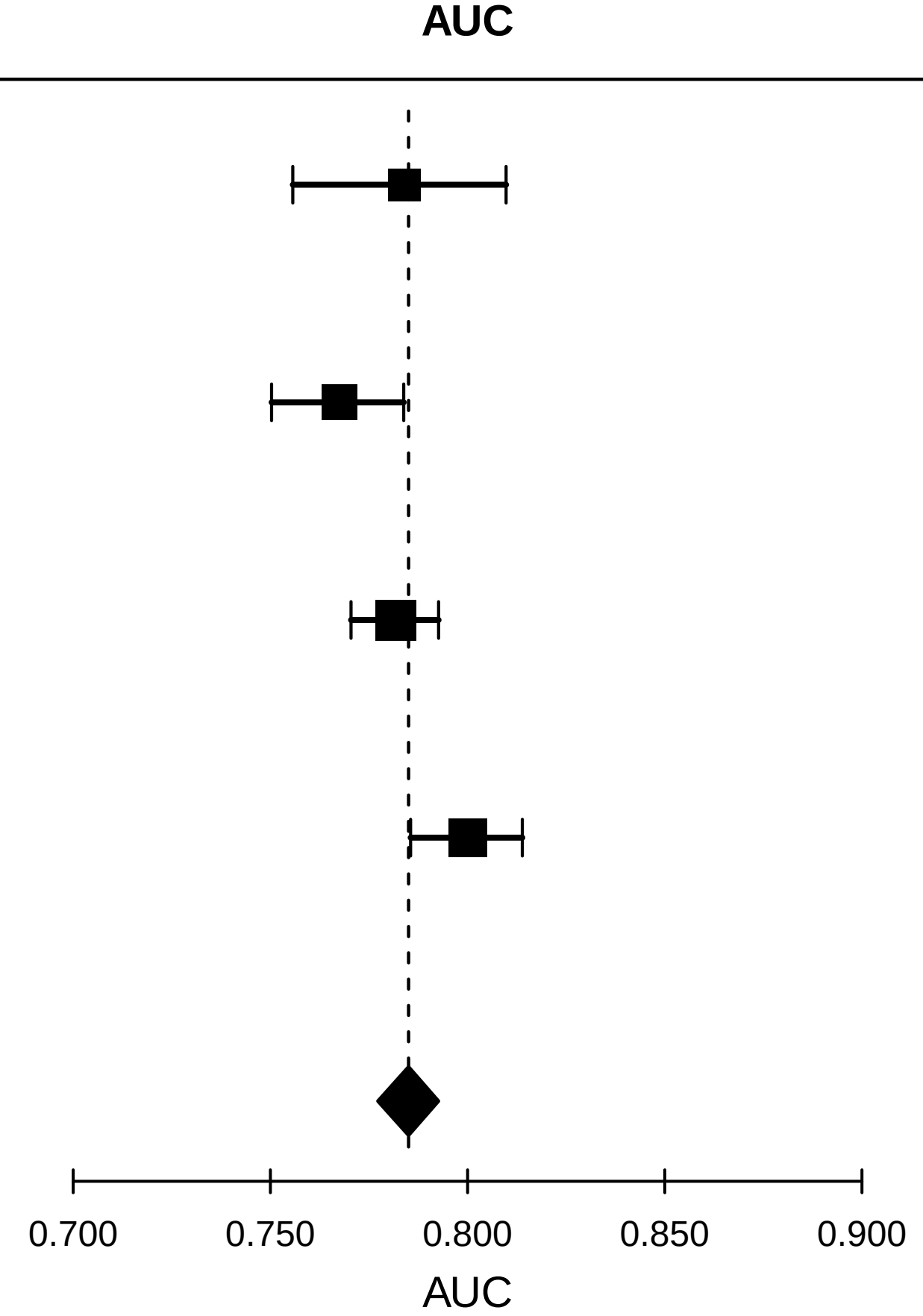

HKSJ-adjusted subgroup difference test: F(3,400) = 2.7829, p = .041. Dashed line marks overall pooled AUC.

Note. We extracted a total of 1,881 records from 219 articles. First, following the priority of four datasets, we selected the record with the largest AUC from each article as the representative model, resulting in 221 records, of which 190 were from surviving cases and 31 were for mortality following AKI. Subsequently, to fully explore and compare model performance, we merged the 1,881 rows of data based on the same article and the same model, yielding 404 records. Pooled AUCs and 95% CIs were estimated on the logit-transformed AUC scale using REML random-effects models with Hartung-Knapp-Sidik-Jonkman adjusted inference and back-transformed to the AUC scale. The dashed line indicates the overall pooled AUC.

Supplementary Figure S5. Complementary Model-Level Subgroup Meta-analysis of AUCs for Mortality Following AKI Prediction by AI Model Application Type

REML random-effects subgroup meta-analysis with HKSJ-adjusted inference; overall pooled AUC = 0.785 (0.769−0.800)

Model class	No.of AUC estimates (k)	AUC (95% CI)	I^2	tau^2
Deep learning models	9	0.764 (0.713−0.808)	99.4%	0.116
Hybrid models and others	16	0.770 (0.721−0.813)	99.8%	0.237
Linear/generalized linear models	23	0.782 (0.759−0.804)	98.7%	0.092
Tree-based and ensemble	24	0.804 (0.777−0.829)	99.9%	0.147
**Overall**	**72**	**0.785 (0.769−0.800)**	**99.8%**	**0.147**

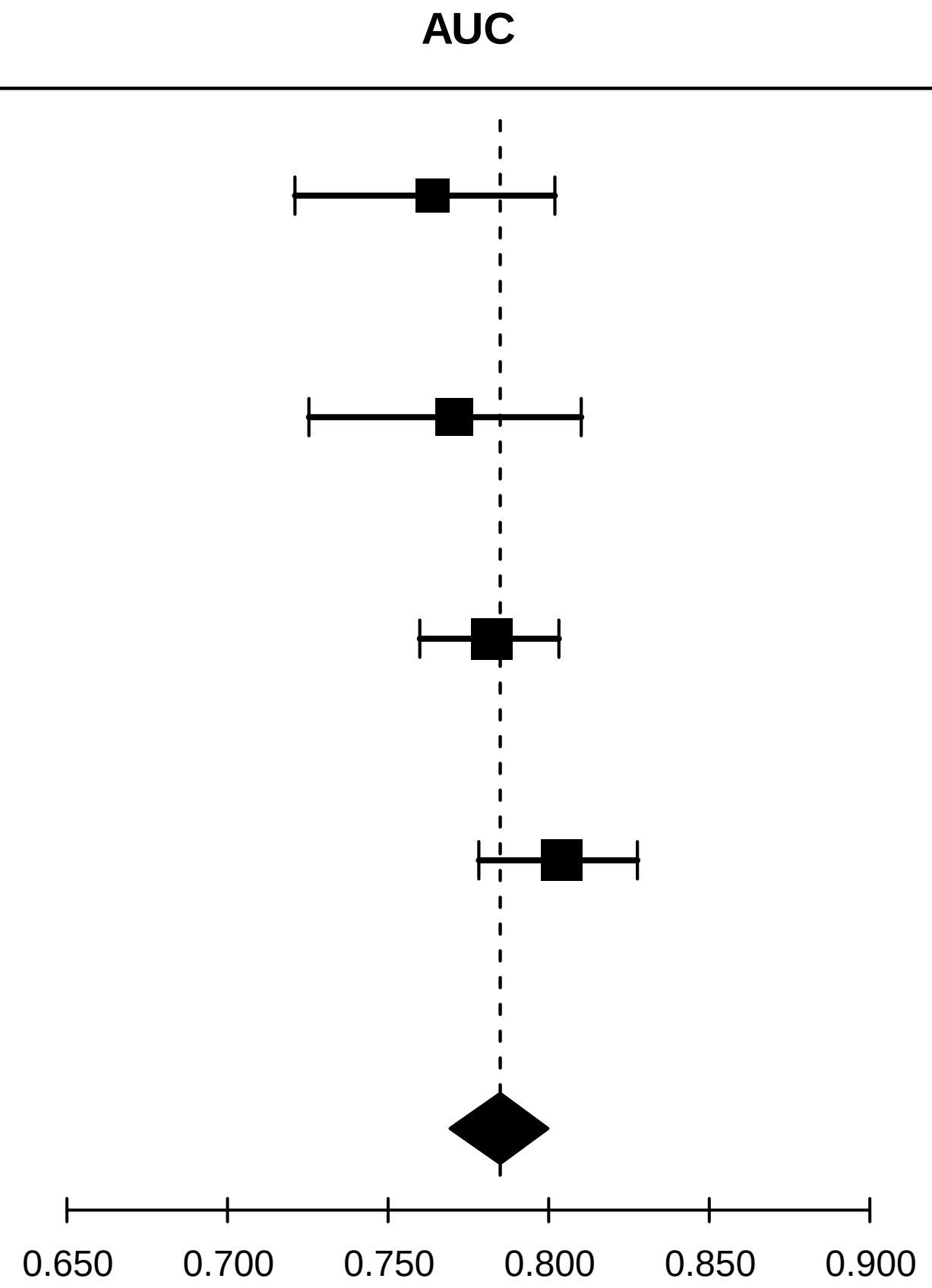

HKSJ-adjusted subgroup difference test: F(3,68)=1.2906; P=.285

Supplementary Figure S6. Multivariable Meta-regression Coefficients for AUCs of AKI Occurrence and Mortality Following AKI Prediction Models

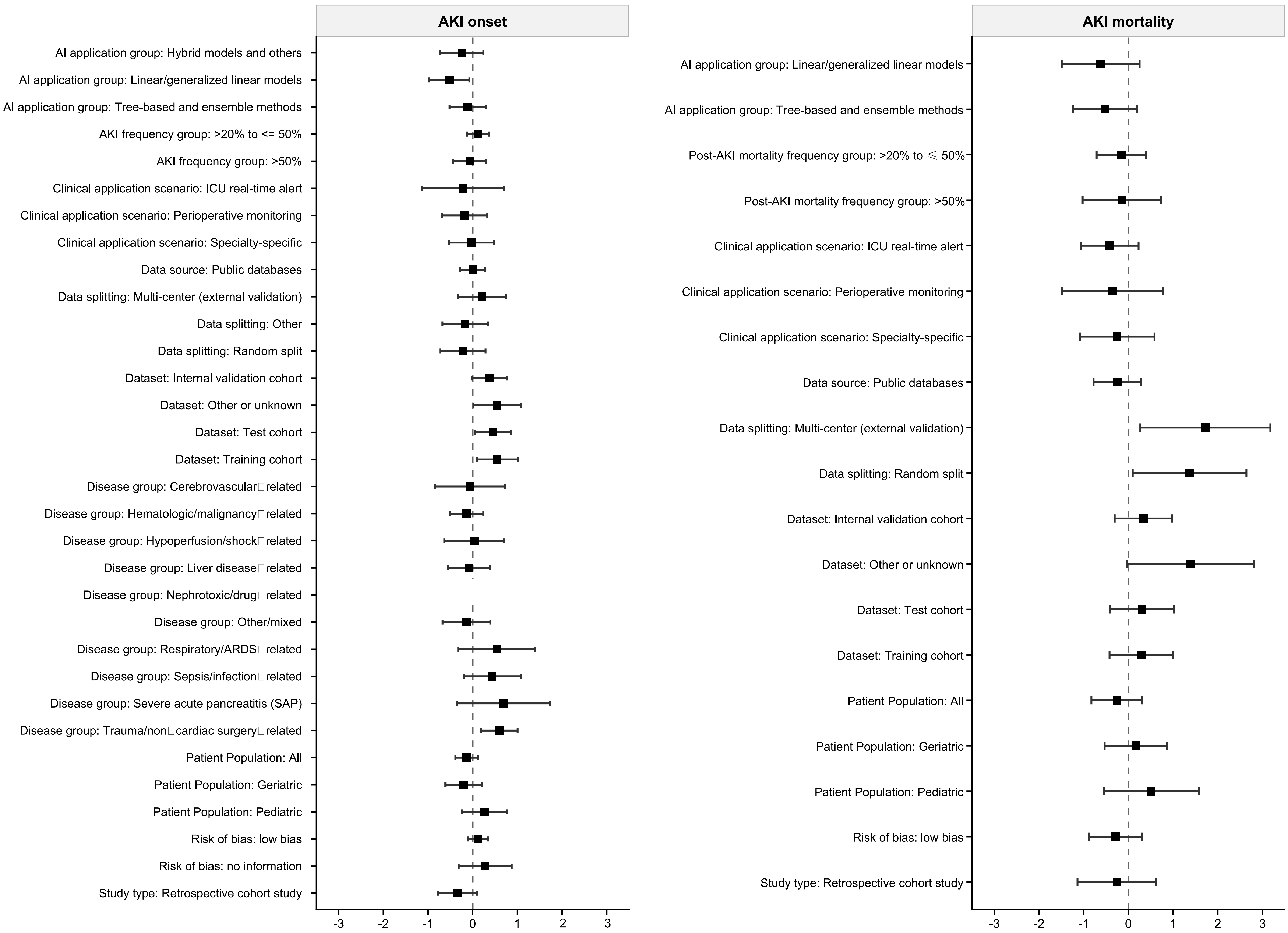

Supplementary Figure S7A. Forest Plot for Low Risk-of-Bias Sensitivity Analyses of AUCs for AKI Occurrence Prediction Models (Page 1 of 3)

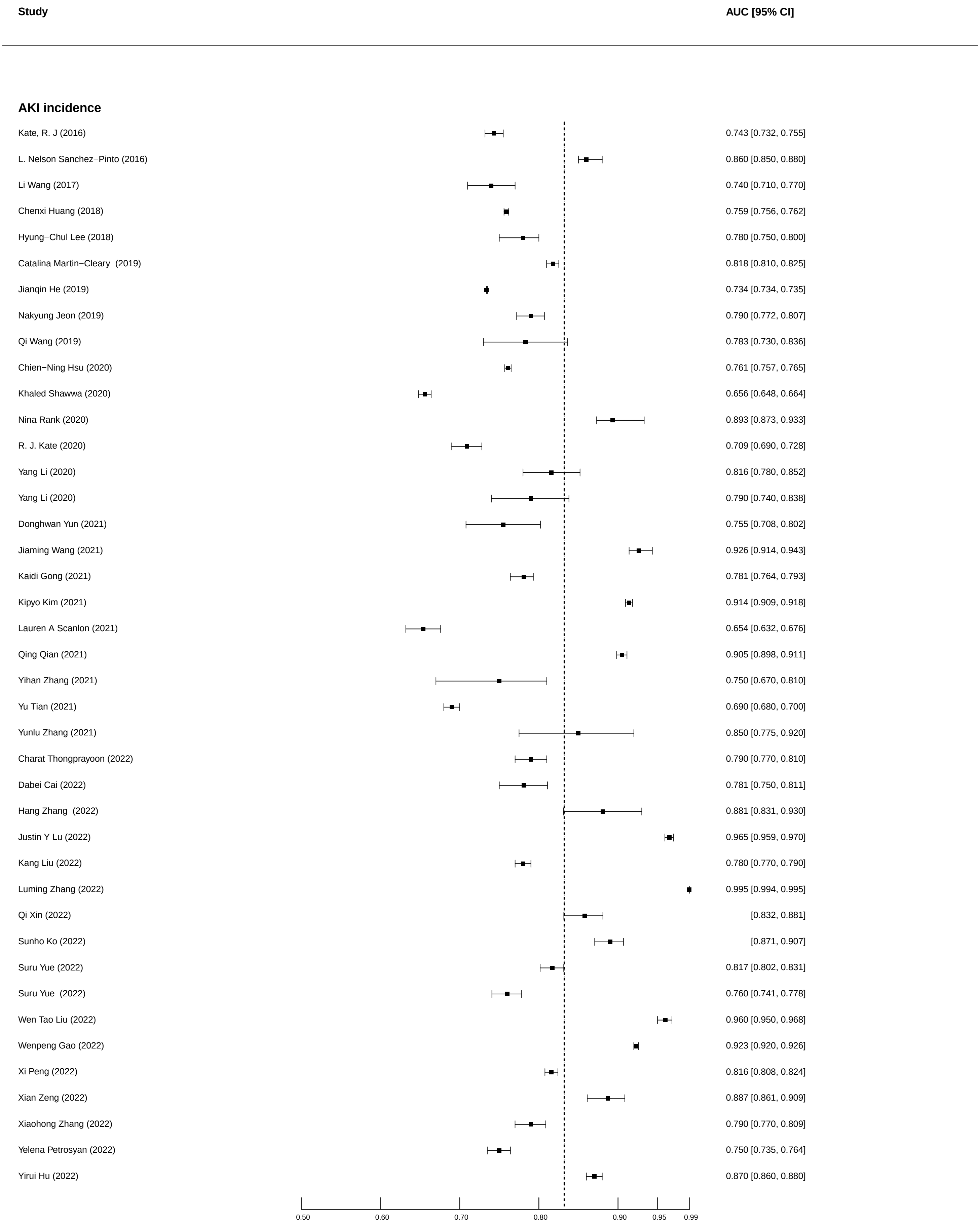

f Supplementary Figure S7A. Forest Plot for Low Risk-of-Bias Sensitivity Analyses of AUCs for AKI Occurrence Prediction Models (Page 2 of 3)

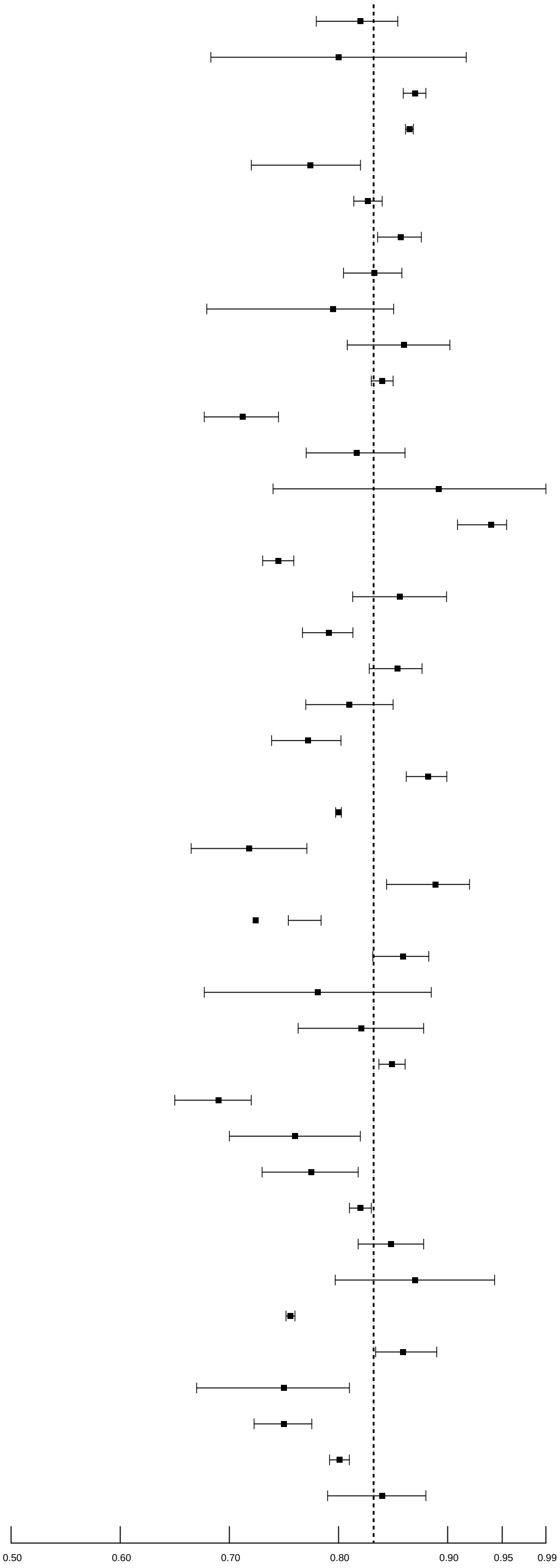

Study	AUC [95% CI]
**AKI incidence (continued)**	
Yuxi Li (2022)	0.820 [0.780, 0.854]
Anran Dai (2023)	0.800 [0.683, 0.917]
Christopher T Ryan (2023)	0.870 [0.859, 0.880]
Chun−Te Huang (2023)	0.865 [0.861, 0.869]
Fangfang Zhou (2023)	0.774 [0.720, 0.820]
Hayato Akimoto (2023)	0.827 [0.814, 0.840]
Hsin−Hung Liu (2023)	0.857 [0.836, 0.876]
Jia Tian (2023)	0.833 [0.805, 0.858]
Jiaqi Lu (2023)	0.795 [0.679, 0.851]
Jicheng Jiang (2023)	0.860 [0.808, 0.902]
Lijuan Wu (2023)	0.840 [0.830, 0.850]
Luyan Zheng (2023)	0.712 [0.677, 0.745]
Mengqing Ma (2023)	0.816 [0.770, 0.861]
Pan Pan (2023)	0.892 [0.740, 0.997]
Ping Yan (2023)	0.940 [0.909, 0.954]
Qian Li (2023)	0.745 [0.730, 0.759]
Rui Fan (2023)	0.856 [0.813, 0.899]
Shengqiang Chi (2023)	0.791 [0.767, 0.813]
Shuxing Wei (2023)	0.854 [0.828, 0.877]
Siavash Zamirpour (2023)	0.810 [0.770, 0.850]
Simin Wu (2023)	0.772 [0.739, 0.802]
Tianchen Jia (2023)	0.882 [0.862, 0.899]
Tingting Fan (2023)	0.800 [0.797, 0.803]
Wenxing Peng (2023)	0.718 [0.665, 0.771]
Xiao−Qin Luo (2023)	0.889 [0.844, 0.920]
Xinyi Cao (2023)	0.724 [0.754, 0.784]
Yang Zhou (2023)	0.859 [0.831, 0.883]
Yefeng Tong (2023)	0.781 [0.677, 0.885]
Yixin Tian (2023)	0.821 [0.763, 0.878]
Yuchen Gao (2023)	0.849 [0.837, 0.861]
Yun Yan (2023)	[0.650, 0.720]
Ziming Jiang (2023)	[0.700, 0.820]
Amir Hossein Behnoush (2024)	0.775 [0.730, 0.818]
Benjamin R Griffin (2024)	0.820 [0.810, 0.830]
Buqing Ma (2024)	0.848 [0.818, 0.878]
Chen Lin (2024)	0.870 [0.797, 0.943]
Emma Schwager (2024)	0.756 [0.752, 0.760]
Enci Cai (2024)	0.859 [0.834, 0.890]
Hai−Xia Liu (2024)	0.750 [0.670, 0.810]
Huilan Tu (2024)	0.750 [0.723, 0.775]
Jianshan Shi (2024)	0.801 [0.792, 0.810]
Jiaxin Li (2024)	0.840 [0.790, 0.880]

Supplementary Figure S7A. Forest Plot for Low Risk-of-Bias Sensitivity Analyses of AUCs for AKI Occurrence Prediction Models (Page 3 of 3)

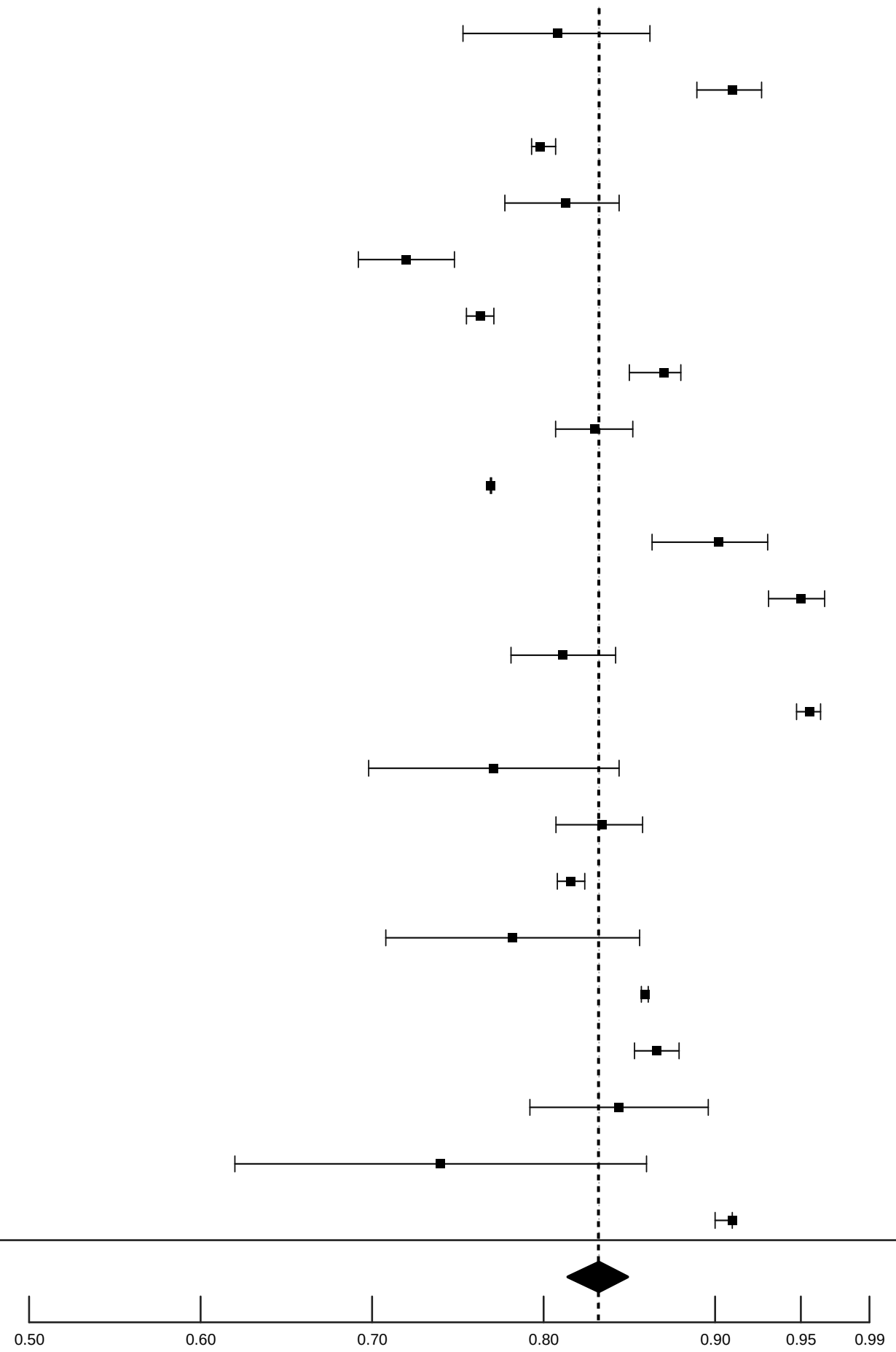

Study	AUC [95% CI]
**AKI incidence (continued)**	
Jun Wei (2024)	0.808 [0.753, 0.862]
Junlong Hu (2024)	0.910 [0.889, 0.927]
Katarzyna Krzanowska (2024)	0.798 [0.793, 0.807]
Lingyu Xu (2024)	0.813 [0.777, 0.844]
Mee Yeon Lee (2024)	0.720 [0.692, 0.748]
Meicheng Yang (2024)	0.763 [0.755, 0.771]
Mengqing Ma (2024)	0.870 [0.850, 0.880]
Rao Sun (2024)	0.830 [0.807, 0.852]
Sang−Wook Lee (2024)	0.769 [0.769, 0.770]
Tao Sun (2024)	0.902 [0.863, 0.931]
Wanyue Zhang (2024)	0.950 [0.931, 0.964]
Xiang Li (2024)	0.811 [0.781, 0.842]
Xiaochi Lu (2024)	0.955 [0.947, 0.961]
XinPei Liu (2024)	0.771 [0.698, 0.844]
Xuejian Hou (2024)	0.834 [0.807, 0.858]
Xunliang Li (2024)	0.816 [0.808, 0.824]
Yufeng Jiang (2024)	0.782 [0.708, 0.856]
Kang Liu (2025)	0.859 [0.857, 0.861]
Ming Ye (2025)	0.866 [0.853, 0.879]
Tao Sun (2025)	0.844 [0.792, 0.896]
Xiaofei Zhang (2025)	0.740 [0.620, 0.860]
Y Zhang (2025)	0.910 [0.900, 0.910]
HKSJ-adjusted random-effects model; I^2 = 100.0%	**0.832 [0.814, 0.849]**

Supplementary Figure S7B. Forest Plot for Low Risk-of-Bias Sensitivity Analyses of AUCs for Mortality Following AKI Prediction Models

Study	AUC [95% CI]
**AKI mortality**	
Daniela Ponce (2021)	0.831 [0.760, 0.890]
Haofan Huang (2021)	0.820 [0.806, 0.833]
Jing−Cheng Peng (2021)	0.907 [0.865, 0.949]
Chang Hu (2022)	0.890 [0.880, 0.897]
Hsin−Hsiung Chang (2022)	0.823 [0.791, 0.854]
Xiao−Qin Luo (2022)	0.848 [0.838, 0.858]
Changwei Wu (2023)	0.924 [0.916, 0.932]
Hongshan Zhou (2023)	0.754 [0.744, 0.764]
Jijun Yang (2023)	0.873 [0.860, 0.886]
Xunliang Li (2023)	0.794 [0.762, 0.827]
Zhiyan Fan (2023)	0.810 [0.790, 0.830]
Caifeng Li (2024)	0.720 [0.680, 0.760]
Jie Tang (2024)	0.804 [0.791, 0.816]
Le Li (2024)	0.784 [0.774, 0.794]
Lei Dong (2024)	0.796 [0.742, 0.852]
Menglei Gu (2024)	0.800 [0.777, 0.821]
Shengtao Li (2024)	0.828 [0.784, 0.865]
Tianyun Gao (2024)	0.798 [0.774, 0.821]
Xiang Li (2024)	0.799 [0.762, 0.831]
Yamin Liu (2024)	0.724 [0.648, 0.800]
Yongbin Wang (2024)	0.756 [0.702, 0.809]
Luyan Zheng (2025)	0.787 [0.745, 0.830]
HKSJ-adjusted random-effects model; I^2 = 97.4%	**0.819 [0.792, 0.843]**

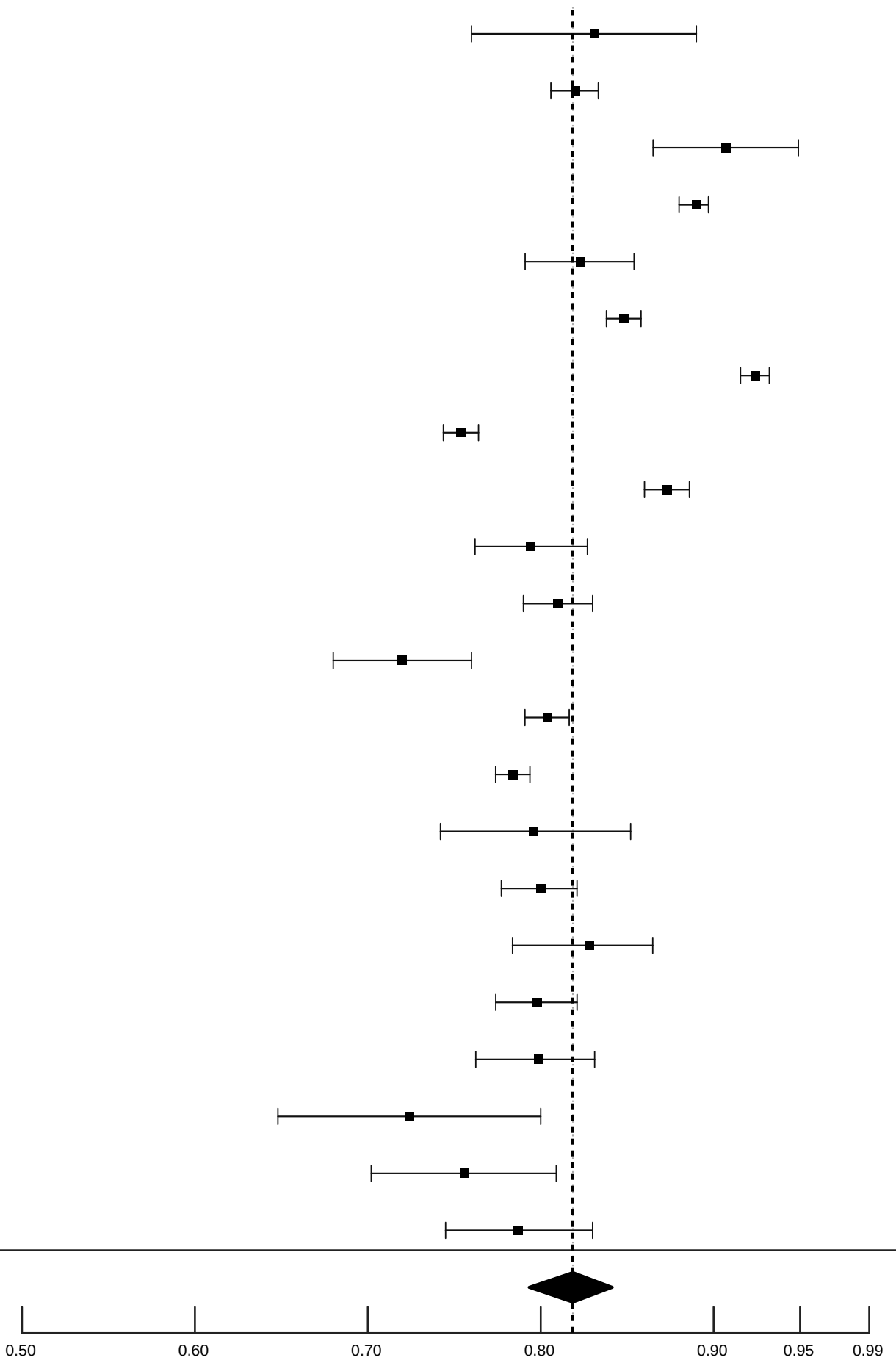

Supplement: Supplementary file 1 [file jmir-v28-e104269-s001.pdf]
